# The phosphorylation of PHF5A by TrkA-ERK1/2-ABL1 cascade regulates centrosome separation

**DOI:** 10.1038/s41419-023-05561-1

**Published:** 2023-02-09

**Authors:** Chen Song, Yu Zhang, Yutong Li, Juntao Bie, Zhe Wang, Xin Yang, Haishuang Li, Liangyi Zhu, Tianzhuo Zhang, Qing Chang, Jianyuan Luo

**Affiliations:** 1 Department of Medical Genetics, Center for Medical Genetics, Peking University Health Science Center, Beijing, 100191 China; 2 Department of Pathology, Peking University School of Basic Medical Science; Peking University Third Hospital, Peking University Health Science Center, Beijing, 100191 China; 3 Department of Biochemistry and Biophysics, Beijing Key Laboratory of Protein Posttranslational Modifications and Cell Function, Center for Medical Genetics, Peking University Health Science Center, Beijing, 100191 China; 4 Present Address: Institute for Cancer Genetics, Columbia University, New York, NY 10032 USA

**Keywords:** Biochemistry, Cell biology, Cancer

## Abstract

During interphase, the newly duplicated pairs of centrosomes are held together by a centrosome linker, and the centrosome separation needs the disruption of this linker to induce the duplicated centrosomes separating into two distinct microtubule organization centers. The mechanism of regulating centrosome separation is however poorly understood. Here, we demonstrated that the phosphorylation of PHF5A at Y36 by the TrkA-ERK1/2-ABL1 cascade plays a critical role in regulating centrosome separation. PHF5A, a well-characterized spliceosome component, is enriched in the centrosome. The pY36-PHF5A promotes the interaction between CEP250 and Nek2A in a spliceosomal-independent manner, which leads to premature centrosome separation. Furthermore, the unmatured centrosome remodels the microtubule and subsequently regulates cell proliferation and migration. Importantly, we found that the phosphorylation cascade of TrkA-ERK1/2-ABL1-PHF5A is hyper-regulated in medulloblastoma. The inhibition of this cascade can induce senescence and restrict the proliferation of medulloblastoma. Our findings on this phosphorylation cascade in regulating centrosome separation could provide a series of potential targets for restricting the progress of medulloblastoma.

## Introduction

The centrosome is the most important microtubule organization center in mammalian cells, which regulates spindle formation and cell division in the mitotic cell cycle and influents cell migration, motility, and cellular organelles organization [1]. During the cell cycle, the older centrosome disengages in the G1 phase and duplicates in the S phase to form a pair of new centrosomes once per cell cycle and moves apart to form the opposing mitotic poles [2]. During the centrosome maturation, the pericentriolar materials are recruited and surrounded by the centrosome [3]. The duplicated mature centrosomes anchor and nucleate microtubule via the gamma-tubulin ring complex in the pericentriolar materials [4]. The opposing centrosomes and the parallel microtubule bundles function as the mitotic spindle and help to align the sister chromatid paired for accurate chromosome segregation [5]. The structure, function, and number of the centrosome are strictly controlled in cells to ensure the cellular process [6]. The numerical centrosome abnormality is the most common dysfunction of the centrosome [6]. The overduplication, mitosis failure, or improper centrosome separation cause the increase of centrosome number which may result in multipolar mitosis and generate chromosomal instability [7, 8].

During the G1 phase, the mother and daughter centrioles are held together by a proteinaceous linker between the proximal ends of the centrioles. To form the mitotic spindle, the centrosome linker is disassembled to facilitate the new pair of centrosome disjunctions [1]. Two important components of centrosome linker are CEP250 and rootletin. CEP250 is located at the proximal ends of the centrioles, and functions as the docking site for rootletin [9]. Rootletin assembles into filaments with other proteins, like CEP68 and LRRC45, which binds to CEP250 for centriole attachment [10, 11]. During the late G2 phase and early M phase, CEP250 and rootletin are phosphorylated by NIMA-related kinase 2 (Nek2), which is activated by the CDK1 cascade or growth factor cascade [10]. After phosphorylation, CEP250 is disassembled from the centrosome which causes centrosome disjunction [12]. The disassembled centrosomes move to the opposing mitotic poles with the force of microtubule at the onset of mitosis [13]. CEP250-Nek2A phosphorylation cascade plays a critical role in the regulation of centrosome separation and the cell cycle process. The kinase-dead Nek2A causes the accumulation of multiple centrosomes due to the centrosome separation failure, which increases the degree of aneuploidy and chromosomal instability [14, 15]. Depletion of CEP250 or overexpression of Nek2 also induces premature centrosome separation independent of the cell cycle phase, which leads to the formation of multipolar mitosis with disengaged centrioles [16–18]. Multipolar mitosis causes cell asymmetric divisions and chromosomal instability [19]. As the microtubule organization center, the centrosome also regulates microtubule nucleation and stabilization in mammalian cells [20]. The abnormal centrosome, especially numerical abnormality, affects microtubule density [21]. The proper density of the microtubule network and microtubule-dependent peri-centrosomal assembly ensure the correct location and morphology of the Golgi complex which determines the cell-polarized migration [21, 22]. Due to the importance of the centrosome, the precise timing of centrosome separation and the correct structure of the mitotic spindle ensure the process of mitosis and microtubule organization.

During pre-mRNA maturation, the spliceosome recognizes and removes intron. PHF5A is a crucial scaffolding component presenting in the spliceosome U2 snRNP complex, and forms the branch point pocket with SF3B1 [23]. The mutation of PHF5A could cause resistance to splicing inhibitors [24]. PHF5A can promote colorectal cancer progression by regulating alternative splicing [25, 26]. Besides functions in splicing, PHF5A also has been found that it could play spliceosome-independent functions. It stabilizes the Paf1 transcriptional complex in pluripotent embryonic stem cells' self-renewal and maintenance of pluripotency [27]. Also, PHF5A stabilized the p400 histone chaperone complex and was involved in DNA repair [16].

TrkA, as a canonical receptor tyrosine kinase, is activated by its ligand NGF and transduces the phosphorylation cascade in regulating neurogenesis, cell differentiation, and proliferation [28]. ABL1 showed a pericentriolar matrix location in a growth factor-dependent signaling mechanism [29]. The crosstalk between NGF-TrkA and ABL1 in the centrosome has not been reported.

Medulloblastoma is the most common primary malignant brain tumor in children over the age of 3–5 years. Medulloblastoma has been categorized into four subgroups named Wnt, Shh, Goup3, and Group4 [30]. In Shh-medulloblastoma, the highly conserved Hedgehog (Hh) pathway is abnormally activated which causes the primary cilium abundance [31]. There are several regulators involved in the Hh pathway, including the Suppressor of fused (Sufu) [32]. Sufu localizes to the centrosome and plays an important role in centrosome duplication, suggesting the correlation between Shh-medulloblastoma and centrosome [33]. Since the metastasis of medulloblastoma remains the major cause of mortality in children, an improved understanding of the molecular events driving metastasis represents a major priority for medulloblastoma research [34].

In this study, we found that the phosphorylated PHF5A at site Y36 locates in the centrosome, and regulates centrosome separation by promoting the interaction between CEP250 and Nek2A. The phosphorylation of PHF5A-Y36 is regulated by the TrkA-ERK1/2-ABL1 cascade with the centrosome translocation of ABL1. The hyperphosphorylation of PHF5A-Y36 is observed in the Shh- medulloblastoma with the upregulation of the TrkA-ABL1 cascade and promotes the proliferation and migration of medulloblastoma cells. The TrkA-ABL1-PHF5A cascade could be a series of potential targets to restrict medulloblastoma progress.

## Materials and methods

### Transgenic mice and cell lines

NeuroD2:SmoA1 mice (#008831) were purchased from Jackson Labs.

Daoy MB cell lines were purchased from the Cell Resource Center, Peking Union Medical College. HeLa and HEK293T were purchased from ATCC.

### Cell culture and transfection

HEK293T, Hela, and Daoy cells were cultured on poly-D-lysine-coated dishes or coverslips with DMEM (GIBCO) supplemented with 10% fetal bovine serum (GIBCO) and 1% penicillin/streptomycin. Cells were transfected with plasmid DNA using PEI according to the manufacturer’s protocol. Cells were transfected with siRNA using Lipofectamine RNAimax according to the manufacturer’s protocol. The following siRNA target sequences were used: si-PHF5A-CDS, 5′-CCAUCGAAGACUGUGUGAAA-3′; si-PHF5A-UTR, 5′-GCCUACUACUACCAGCAGAAA-3′; si-CEP250-1#, 5′-GCTGACTCTATTCGACAACAA-3′; si-CEP250-2#, 5′-CCCTGACTCAAAGTCTGACAT-3′; si-Nek2-UTR, 5′-GCCATGCCTTTCTGTATAGTA-3′.

Before NGF (PeproTech) treatment, the cells were serum-starved for 16 h. The proper final concentration of NGF (50 or 100 ng/ml) was added to the cell culture medium still without serum.

### Plasmid and antibody

PCR-amplified human PHF5A, ABL1, and Nek2A were cloned into pcDNA 3.1 vectors (with -HA, -Flag, or -Myc-His tags). PCR-amplified human CETN2 were cloned into pEGFP-C2 vectors. PcDNA 3.1-Flag-PHF5A (all mutant) and pcDNA 3.1-HA-ABL1 (PP and K290R) were constructed using KOD-plus Mutagenesis Kit (TOYOBO). pLVX-TetOne-Puro-Flag-PHF5A (WT, Y36E, Y36F), pQCXIH-Flag-PHF5A, and pHBLV-CMVIE-IRES-Puro EGFP-CETN2 were constructed using ClonExpress II One Step Cloning Kit (Vazyme). ShRNA was constructed via the ligation of an oligonucleotide into a pLKO.1-Puro vector. The following shRNA target sequences were used: shRNA PHF5A CDS, 5′-CCAUCGGAAGACUGUGUGAAA-3′; shRNA PHF5A UTR, 5′-GCCUACUACUACCAGCAGAAA-3′; shRNA ABL1 UTR, 5′- CCAGCTCTACTACCTACGTTT-3′. The following antibodies were obtained from commercial sources: Anti-Flag (Sigma-Aldric, Cat#F-3165), Anti-GFP (Enogene, Cat#E12-009), Anti-pTyr (PY99) (Santa Cruz Biotechnology, Cat#sc-7020), Anti-GAPDH (Cell Signaling Technology, Cat#5174), Anti-HA (Thermo Fisher Scientific, Cat#26183), Anti-TrkA (Bioss, Cat#ba-0193R), Anti-PHF5A (Proteintech, Cat#15554-1-AP), Anti-ERK1/2 (Abcam, Cat#ab184699 and Zenbio, Cat#201246-4F3), Anti-pERK1/2 (Thr202/Tyr204) (Zenbio, Cat#301245), Anti-β-actin (Santa Cruz Biotechnology, Cat#sc-47778), Anti-AKT (Cell Signaling Technology, Cat#4685), Anti-pAKT (Ser473) (Cell Signaling Technology, Cat#4060), Anti-ABL1 (Cell Signaling Technology, Cat#2862), Anti-pABL1 (Tyr412) (Cell Signaling Technology, Cat#2865), Anti-γ-tubulin (mouse: Proteintech, Cat#66320-I-IG and rabbit: Abcam, Cat#ab179503), Anti-CEP250 (mouse: Santa Cruz Biotechnology, Cat#sc-390540 and rabbit: Proteintech, Cat#14498-1-AP), Donkey anti-rabbit Alexa Fluor 488 (Thermo Fisher Scientific, Cat#A21206), Goat anti-mouse Alexa Fluor 594 (Thermo Fisher Scientific, Cat#A32742), Goat anti-mouse Alexa Fluor 488 (Yeason, Cat# 33206ES60), Donkey anti-rabbit Alexa Fluor 350 (Bioss, Cat#bs-0295D-AF250).

### Immunoprecipitation and co-immunoprecipitation assay

Cell lysates were prepared with BC100 buffer (20 mmol/L Tris-HCl (pH 7.9), 100 mmol/L NaCl, 0.2% NP-40, and 20% glycerol) containing protease inhibitor cocktail (Selleck) and used directly for immunoprecipitation. Anti-Flag, anti-HA, or anti-GFP of protein A/G-Sepharose beads were incubated with cell lysates at 4 °C overnight. After BC100 buffer washing, the precipitants were analyzed by Western blot using the indicated antibodies.

### In vitro phosphorylation assay

Full-length His-PHF5A was purified from the *E.coli* bacteria as the substrate in the phosphorylation assay. HA-ABL1 (WT, PP, K290R) were purified from HEK293T cells [35, 36]. Reactions were carried out at 37 °C for 30 min in a reaction buffer containing 66.7 mM Tris-Cl (pH = 8.0), 100 mM NaCl, 5 nM MnSO_4_, 0.5 mM CaCl_2_, 2 mM DTT, and 0.01 mM ATP. Phosphorylation of target proteins was analyzed by western blot using a pan-tyrosine-phosphorylation specific antibody.

### Cell synchronization

Hela or Daoy cells were plated in dishes or coverslips at 50–70% confluence and treated with 5 μM thymidine for 14 or 20 h. Cells were washed with warm PBS five times and refresh the complete medium. To inactive Eg5, monanstrol was added into the complete medium after thymidine blocking and releasing for 5 h.

### Immunofluorescence and immunohistochemistry staining

Cells were fixed with 4% paraformaldehyde for 20 min and permeabilized using 0.25% Triton X-100 for 20 min at room temperature. Use quick antigen retrieval solution for cell section (Solarbio) according to the manufacturer’s protocol to retrieve antigen fixed with 4% paraformaldehyde before blocking. Additionally, Cells were also fixed with −20 °C pre-cooled methanol for 5 min to obtain a better resolution of microtubule staining. After blocking with 1% BSA supplemented with 10% goat serum, the cells were incubated with the primary antibodies overnight at 4 °C. After washing with TBS, the cells were incubated with secondary antibodies conjugated with Alexa-350, Alexa-488, or Alexa-594 for 2.5 h at room temperature. Finally, the cells were stained with DAPI (Solarbio) to visualize the nuclei. Photos were taken under a confocal microscope (LSM-880 with Airyscan, Zeiss). Super-resolution Photos were taken under SP8 LIGHTNING confocal microscope (Leica). Shh-subtype medulloblastoma samples in OCT were washed with TBS three times and performed with antigen retrieval solution (Solarbio) for 5 min at room temperature according to the manufacturer’s protocol. After blocking with 1% BSA supplemented with 10% goat serum, the slices were incubated with the primary antibodies overnight at 4 °C. After washing with TBS, the slices were incubated with secondary antibodies conjugated with HRP for 2.5 h at room temperature. Finally, Using DAB to detect the signal according to the manufacturer’s protocol.

### Living-cell imaging

Cell migration assay was assessed in living Daoy cells with stable overexpression of EGFP-CETN2. Use UltraVIEW VoX (PerkinElmer) to record the cell orientation every 10 min for 24 h. Cell division assay was assessed in living HeLa cells released after the synchronization of thymidine and stained with Hochest 33342. Use UltraVIEW VoX (PerkinElmer) to record every 5 min for 4 h.

### Identification of phosphorylation site by LC-MS/MS analysis

After SDS-PAGE, the band of PHF5A was excised and subjected to in-gel trypsin digestion. LC-MS analyses of protein digests were carried out on an ion trap mass spectrometer (LTQ Velos Pro, Thermo Scientific) coupled with nanoflow reversed-phase liquid chromatography (EASY-nLC 1000, Thermo Scientific). The capillary column (75 μm × 150 mm) with a laser-pulled electrospray tip (Model P-2000, Sutter instruments) was home-packed with 5 μm, 100 Å Magic C18AQ silica-based particles (Michrom BioResources Inc., Auburn, CA) and run at 300 nL/min with the following mobile phases (A: 97% water, 3% acetonitrile, and 0.1% formic acid; B: 80% acetonitrile, 20% water, and 0.1% formic acid). The LC gradient started at 10% B for 3 min and then was linearly increased to 40% in 40 min. Next, the gradient was quickly ramped to 90% in 2 min and stayed there for 10 min. The gradient was then switched back to 100% solvent A for column equilibration. Eluted peptides from the capillary column were electro-sprayed directly onto the mass spectrometer for MS and MS/MS analyses in a data-dependent acquisition mode. One full MS scan (m/z 400–1,200) was acquired and simultaneously the ten most intense ions were selected for MS/MS analyses. The selected ions were fragmented by collision-induced dissociation (CID) in the ion trap with the following parameters: ≥ +2 precursor ion charge, 2 Da precursor ion isolation window, and 35% normalized collision energy. Dynamic exclusion was set with a repeat duration of 30 s and an exclusion duration of 12 s.

### Wound-healing assay

Hela or Daoy PHF5A-TetOne-WT, Y36E, and Y36F cells were seeded into six-well plates and overgrew the dish for around 24 h. Then, cells were wounded with a sterile plastic tip after the inhibitors pretreatment within 24 h. Cell migration was observed and photographed by microscopy (Leica DM IL LED).

### Colony formation assay

Hela (sh-PHF5A with or without rescued PHF5A-WT, Y36E, and Y36F) cells or Daoy PHF5A-TetOne-WT, Y36E, and Y36F cells were seeded into 3.5 cm dishes (4000 cells per dish) and cultured with 10% serum-containing media for about 6 days. Cell clones were fixed with 4% PFA and stained with crystal violet.

### Centrosome enrichment

Centrosomal fractions were prepared from asynchronous HEK293T and Hela cells that were preincubated with 0.2 µM nocodazole and 1 µg/ml cytochalasin D (Sigma-Aldrich) for 1 h at 37 °C as previous study [37]. Cells were collected by trypsinization and centrifugation and the resulting pellet was washed in TBS followed by 0.1× TBS added 8% sucrose. Cells were resuspended in 2 ml of 0.1× TBS added 8% sucrose followed by the addition of 8 ml lysis buffer (1 mM HEPES (pH = 7.2) supplemented with 0.5% NP-40, 0.5 mM MgCl_2_, 0.1%DTT). The lysate was centrifuged at 2500×*g* for 10 min. The supernatant was filtered through a 0.8 µm nylon membrane. The supernatant was gently underlaid with 1 ml of 60% sucrose solution (10 mM Pipes pH 7.2, 0.1% Triton X-100, and 0.1% β-mercaptoethanol containing 60% [wt/wt] sucrose) and spun at 10,000×*g* for 30 min to sediment centrosomes onto the cushion. The upper 8 ml of the supernatant was removed, and the remainder, including the cushion, containing the concentrated centrosomes was gently vortexed and loaded onto a discontinuous sucrose gradient consisting of 70, 50, and 40% solutions from the bottom, respectively, and centrifuged at 100,000×*g* for 1.5 h. After centrifugation, several 500-µl fractions were collected and examined by Western blot analysis.

### Statistics and reproducibility

A two-tailed unpaired Student *t*-test and two-way ANOVA which were used to analyze the differences among more than two groups by SPSS 20 was done for the statistical analyses without a specific statement. ∗ denotes *p* < 0.05, ∗∗ denotes *p* < 0.01, ∗∗∗ denotes *p* < 0.005, and ∗∗∗∗ denotes *p* < 0.001, which were considered statistically significant between groups. Fractions are represented as mean ± s.e.m. Data were graphed using GraphPad Prism 8.0. The western blotting outcome was quantified by using ImageJ.

In cellular composition analysis, data distribution was assumed to be normal, but this was not formally tested.

Mice with similar tumor size and age were randomized and blinded to perform western blotting or IF/ IHC. The sample size for western blotting or IF/ IHC performed with a mouse model, based on the consistency of measurable differences between groups, usually, *n* > 5 mice per group unless otherwise indicated. No animals were excluded from the analysis.

## Results

### TrkA phosphorylates PHF5A at site Y36

**Fig. 1:**
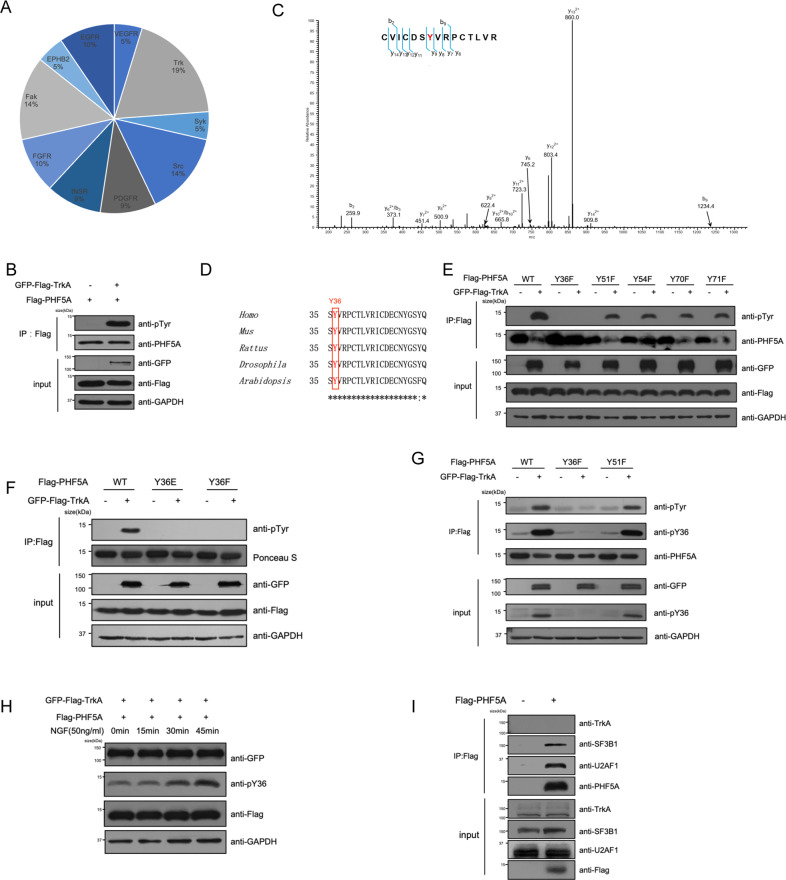
TrkA phosphorylates PHF5A at site Y36. **A** The result of the kinase predicted with software GPS 5.0. **B** HEK293T cells were transfected with plasmids as indicated, and Flag-PHF5A proteins were immunoblotted to verify the phosphorylation induced by TrkA overexpression. **C** The MS/MS spectrum of phosphorylated “CVICDSYVRPCTLVR”. **D** The sequences of PHF5A in five species were aligned. Evolutionarily conserved tyrosine 36 of PHF5A was highlighted in red. **E**, **F** All five tyrosine residues (Y) mutated to phenylalanine (F) of PHF5A (E) and Y36 mutated to glutamate (Y36E) or F (Y36F) of PHF5A (F) were transfected into HEK293T cells. Proteins were immunoprecipitated and detected by pan anti-tyrosine-phosphorylation antibody. **G** Y36F or Y51F was co-expressed with TrkA in HEK293T cells. Using pan anti-tyrosine-phosphorylation antibody and specific antibody pY36 to detect phosphorylation of PHF5A-Y36. **H** After serum starvation of 16 h, HEK293T cells were treated with NGF (50 ng/ml) for indicated time periods. **I** HEK293T cells were transfected with indicated plasmids with NGF treatment. Co-immunoprecipitation and immunoblotting were performed with indicated antibodies.

In spliceosome U2 snRNP, PHF5A interacts with SF3B1 to form the branch point pocket [38]. On their interaction interface, there are two important contact sides in PHF5A, Y36, and Y51/Y54 [39]. Since the regulation of the interaction between PHF5A and SF3B1 is important for alternative splicing, we speculated that Y36 and Y51/Y54 of PHF5A could be phosphorylated to regulate the interaction of PHF5A and SF3B1. To confirm this hypothesis, we first used the PhosphositePlus software to predict the potential phosphorylation of these sites. We found that Y36 and Y51 could be phosphorylated (Fig. S1A). To find the kinases for these sites, we used GPS 5.0 software for the prediction and found that TrkA could be the tyrosine kinase that phosphorylates PHF5A at Y36/Y51 (Fig. 1A) [40]. Phosphorylation assay and liquid chromatography-tandem mass spectrometry (LC-MS/MS) showed that TrkA phosphorylates PHF5A at the evolutionally conserved residue Y36 (Fig. 1B–D). We mutated all five tyrosine residues to phenylalanine (F), which mimicked the dephosphorylated state of PHF5A, and clarified that Y36 was the major phosphorylation site of PHF5A mediated by TrkA (Fig. 1E). We further examined the Y36F and Y36E (hyperphosphorylated mimic), and verified that both Y36F and Y36E mutants abolished phosphorylation of PHF5A (Fig. 1F). To further investigate the role of PHF5A phosphorylation at Y36, we generated Y36 phosphorylation specific antibody (labeled as “pY36” in figures) to detect the phosphorylation of PHF5A at Y36. Dot blotting and immunoprecipitated PHF5A-Y36F or Y51F were performed to ensure the pY36 antibody specificity (Fig. S1B and Fig. 1G). Treatment of NGF, the ligand of TrkA, rapidly induced the phosphorylation of PHF5A-Y36 WT, not the Y36F and Y36E mutants (Fig. 1H and Fig. S1C). As a canonical receptor tyrosine kinase, the majority of TrkA locates in the cell membrane or endosome, while PHF5A, a component of spliceosome, mainly locates in the nucleus. Co-immunoprecipitation analysis showed no interaction between TrkA and PHF5A (Fig. 1I and Fig. S1D). These results suggested that TrkA phosphorylates PHF5A at Y36 indirectly.

### TrkA-ERK1/2-ABL1 cascade phosphorylates PHF5A

**Fig. 2:**
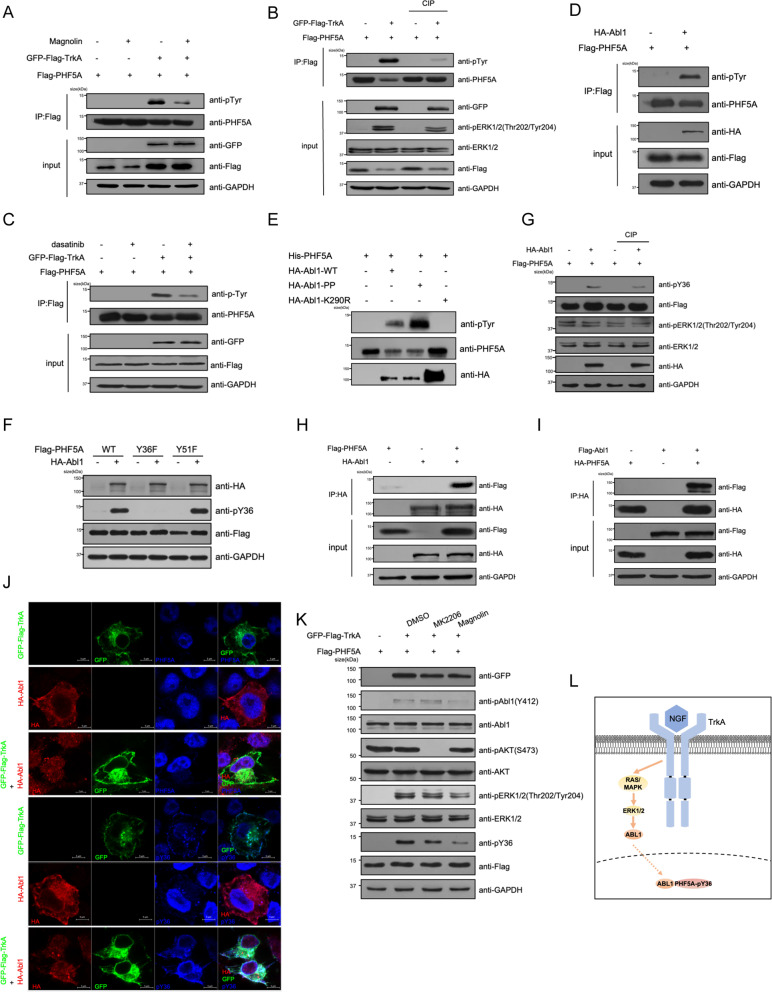
TrkA-ERK1/2-ABL1 cascade phosphorylates PHF5A. **A** HEK293T cells expressing Flag-PHF5A were treated with or without 60 μM ERK1/2 inhibitor magnolin for 1 h. Immunoprecipitated Flag-PHF5A was detected by a pan-tyrosine-phosphorylation antibody. **B** Immunoprecipitated Flag-PHF5A and whole-cell lysate co-expressed with TrkA were incubated with or without CIP (5 u) for 30 min at 37 °C. **C** HEK293T cells expressing Flag-PHF5A were treated with or without 5 nM ABL1 inhibitor dasatinib for 1 h. **D** Flag-PHF5A and HA-Abl1 were co-expressed in HEK293T cells. Immunoprecipitation of PHF5A were immunoblotted and detected by pan anti-tyrosine-phosphorylation antibody. **E** Bacterially purified His-PHF5A was incubated with or without HA-Abl1(wild-type, constitutively activate mutant PP, and kinase-inactive mutant K290R) purified from HEK293T cells for an in vitro kinase assay. **F** Y36F or Y51F was co-expressed with Abl1 in HEK293T cells. Using pY36 to detect phosphorylation of PHF5A-Y36. **G** Immunoprecipitated Flag-PHF5A and whole-cell lysate co-expressed with Abl1 were incubated with or without CIP (5 u) as previously. **H**, **I** HEK293T cells were transfected with indicated plasmids. Using anti-HA agarose beads (**H**) and anti-HA magnetic beads (**I**) to immunoprecipitate HA-Abl1 or HA-PHF5A. **J** HEK293T cells were transfected with indicated plasmids for 48 h. Immunofluorescence analyses were performed with indicated antibodies. **K** HEK293T cells were transfected with indicated plasmids and treated with or without MK2206 (5 μM) or magnolin (60 μM) for 1 h. Immunoblotting was performed with indicated antibodies. **L** Model for TrkA-ERK1/2-ABL1-PHF5A phosphorylation cascade.

TrkA has been reported to transduce two major downstream signal pathways canonically: PI3K-AKT and MEK-ERK. To determine the pathway that TrkA phosphorylates PHF5A, we inhibited the signaling pathway downstream of TrkA. We found that treatment with the ERK1/2 inhibitor, magnolin, blocked the phosphorylation of PHF5A (Fig. 2A). In contrast, MK2206, the inhibitor of AKT, had no effect on the phosphorylation level of PHF5A (Fig. S2A). The treatment of calf intestinal phosphatase (CIP) reduced the phosphorylation levels of both ERK1/2 and PHF5A induced by TrkA (Fig. 2B). Among the prediction of GPS 5.0 software, src-family kinase could also be the kinase that phosphorylates the PHF5A. ABL1 is a member of the src-family tyrosine kinase which can be translocated into the nucleus [41]. Treating cells with dasatinib, an inhibitor of ABL1, reduced the phosphorylation level of PHF5A mediated by TrkA (Fig. 2C). In vivo and in vitro phosphorylation assays showed that ABL1 phosphorylates PHF5A directly (Fig. 2D, E). ABL1-K290R (kinase-inactive mutant) or PHF5A-Y36E abolished the phosphorylation of PHF5A (Fig. 2E, F). Also, overexpression of ABL1 phosphorylated PHF5A which could be dephosphorylated by CIP (Fig. 2G). Co-immunoprecipitation analysis confirmed the interaction between ABL1 and PHF5A (Fig. 2H, I). Indeed, immunofluorescent staining showed that ABL1 translocated into the nucleus when co-expressed with TrkA, which promoted pY36-PHF5A exporting out of the nucleus (Fig. 2J). Treating cells with magnolin and taletrecitinib, the inhibitor of TrkA, can abolish the phosphorylation of both ABL1 and PHF5A, while MK2206 has no effect (Fig. 2K and Fig. S2C, D). These results demonstrated that TrkA-ERK1/2-ABL1 cascade phosphorylated PHF5A (Fig. 2L).

### ABL1 is translocated into the centrosome to phosphorylate PHF5A

To investigate whether phosphorylated PHF5A could regulate the alternative splicing, we performed the RNA-seq in cells with PHF5A knocked down and rescued with PHF5A-WT or PHF5A-Y36E. The results showed that the genes that PHF5A-Y36E regulated were clustered mainly in proteolysis (Fig. S3A, B). And the alternative splicing was not the major event regulated by PHF5A-Y36E (Table S1). Therefore, we speculated that pY36-PHF5A may perform spliceosome-independent functions.

**Fig. 3:**
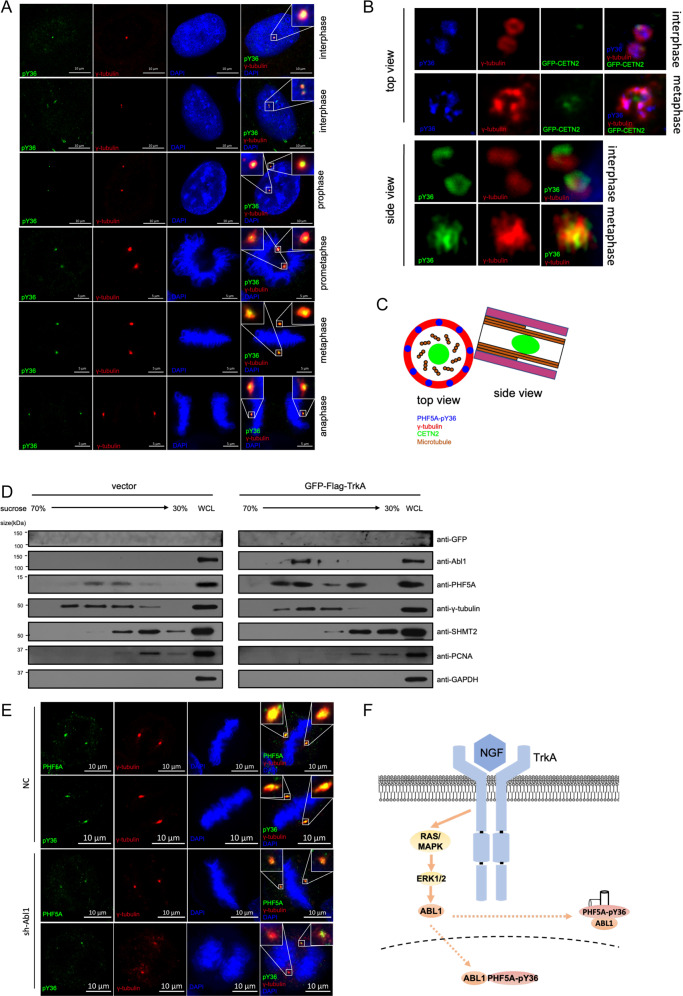
ABL1 translocates to centrosome to phosphorylate PHF5A. **A** HeLa cells were co-stained with anti-pY36 and anti-γ-tubulin antibodies. Insets show the enlarged centrosomal region. **B** Super-resolution images of PHF5A-pY36 and γ-tubulin with or without stable overexpression of GFP-CETN2. **C** Model for centrosomal localization of PHF5A-pY36. **D** After enriching centrosomal fractions by centrifugation, endogenous PHF5A and ABL1 levels were measured in fractions of HEK293T whole-cell lysates (WCL) with or without the expression of GFP-Flag-TrkA. **E** HeLa cells were infected with sh-Abl1 lentivirus or control lentivirus and stained with PHF5A or PHF5A-pY36. **F** Model for ABL1 translocation and phosphorylation of PHF5A in the centrosome.

To investigate the spliceosome-independent function of PHF5A, we first examined the location of pY36-PHF5A, the pY36-specific antibody was used for immunofluorescent staining. Surprisingly, the result showed that the majority of pY36-PHF5A appeared as puncta outside the nucleus, and co-localized with γ-tubulin (Fig. 3A). With high-resolution microscopy, we further found that pY36-PHF5A located in the plus-end of the centrosome during the interphase, then expanded in the whole centrosome during the metaphase (Fig. 3B, C). To confirm the centrosome localization of PHF5A, we purified the centrosome by sucrose gradient ultracentrifugation and found that PHF5A copurified with γ-tubulin. Overexpression of TrkA, PHF5A centrosome localization levels was increased (Fig. 3D). Interestingly, Overexpression of TrkA, ABL1 was also copurified with γ-tubulin (Fig. 3D), suggesting that ABL1 was also translocated into the centrosome. In addition, we found that knockdown ABL1 abolished the pY36 phosphorylation in the centrosome (Fig. 3E). These data suggested that ABL1 was translocated into the centrosome to phosphorylate PHF5A (Fig. 3F).

To investigate the pre-mRNA splicing process at the centrosome, we performed sucrose gradient ultracentrifugation to purify the centrosome. The results showed that SF3B1, the core component of U2 snRNP, was not copurified with γ-tubulin (Fig. S3C). Importantly, mRNA was also shown low abundance in centrosome (Fig. S3D). These data suggested that there was a low possibility to proceed pre-mRNA splicing at the centrosome and pY36-PHF5A is very likely to play spliceosome-independent function at the centrosome.

### pY36-PHF5A downregulates CEP250 by promoting the interaction between CEP250 and Nek2A

**Fig. 4:**
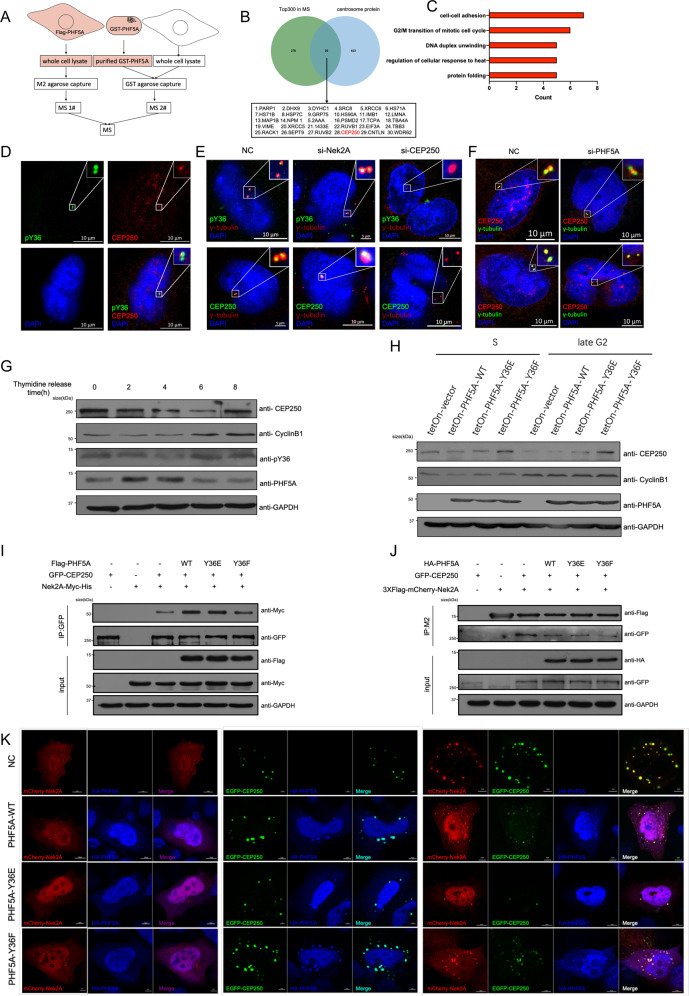
pY36-PHF5A downregulates CEP250 by promoting the interaction between CEP250 with Nek2A. **A**, **B** The schematic depicts the experimental procedure of mass spectrometry (**A**), the box (**B**) shows centrosomal proteins identified by mass spectrometry which are ordered by spectral counts. **C** The bar graph shows the functional enrichment of proteins shown in the box (**B**). **D** The HeLa cells were co-stained with PHF5A-pY36 and CEP250. **E**, **F** SiRNA transfection was used to deplete, Nek2A (**E**), CEP250 (**E**), or PHF5A (**F**), and immunofluorescence were performed with indicated antibodies. **G** HeLa cells were synchronized by thymidine block and released at the indicated times. Whole-cell lysates were analyzed with immunoblotting. **H** Inducible expression of PHF5A (wild-type, Y36E and Y36F) in HeLa cells upon doxycycline treatment for 24 h, and then cells were synchronized by treatment with thymidine block, and then collected after 0 and 6 h of release. **I**, **J** HEK293T cells were transfected with indicated plasmids. CEP250 or Nek2A were immunoprecipitated and Nek2A or CEP250 were detected with immunoblotting. **K** HeLa cells were transfected with indicated plasmids. Immunofluorescence staining were performed to show the colocalization of CEP250, Nek2A, and PHF5A.

To gain insight into the function of PHF5A at the centrosome, we identified the interaction partners of PHF5A using in vitro and in vivo co-immunoprecipitation assays (Fig. 4A). We found that a fraction of 30 centrosomal proteins interacted with PHF5A, including CEP250, a well-characterized centrosomal linker (Fig. 4B, C) [42]. We confirmed the colocalization of PHF5A and CEP250, which were located in the proximal end of the centrosome and contributed to centrosome engagement (Fig. 4D). To investigate whether the centrosomal colocalization of PHF5A and CEP250 was interdependent, we performed siRNA assay that targeted either protein or used drugs to destabilize cytoskeletons. The results showed that only the depletion of CEP250 but not cytoskeleton destabilizing decreased the signal of both PHF5A and pY36-PHF5A in the centrosome (Fig. 4E and Fig. S4A, B). In contrast, the knockdown of PHF5A had little effect in abolishing the centrosomal signal of CEP250 (Fig. 4F). We investigated whether pY36-PHF5A was recruited and involved in the dissociation of CEP250 from the centrosome. We found an increasing signal of pY36-PHF5A during the S phase to the G2 phase accompanied by a decreasing signal of CEP250 (Fig. 4G). To investigate the correlation between CEP250 and pY36-PHF5A, we constructed a conditional expression system in the HeLa cells and synchronized them in the S phase and late G2 phase. The overexpression of PHF5A-Y36F rescued the decreasing signal of CEP250 at the late G2 phase (Fig. 4H). The processing of CEP250 is regulated by Nek2A-mediated phosphorylation [43]. We further investigated the role of pY36-PHF5A in affecting CEP250 processing. The co-immunoprecipitation assays showed that PHF5A-Y36E but not PHF5A-Y36F could promote the interaction between CEP250 and Nek2A (Fig. 4I, J). The immunofluorescent staining assay showed that PHF5A-Y36E but not PHF5A-Y36F could downregulate the protein levels of CEP250 (Fig. 4K). All the above data demonstrated that pY36-PHF5A downregulates CEP250 during the S phase to the G2 phase by promoting the interaction between CEP250 and Nek2A.

### pY36-PHF5A promotes centrosome separation and microtubule remodeling

**Fig. 5:**
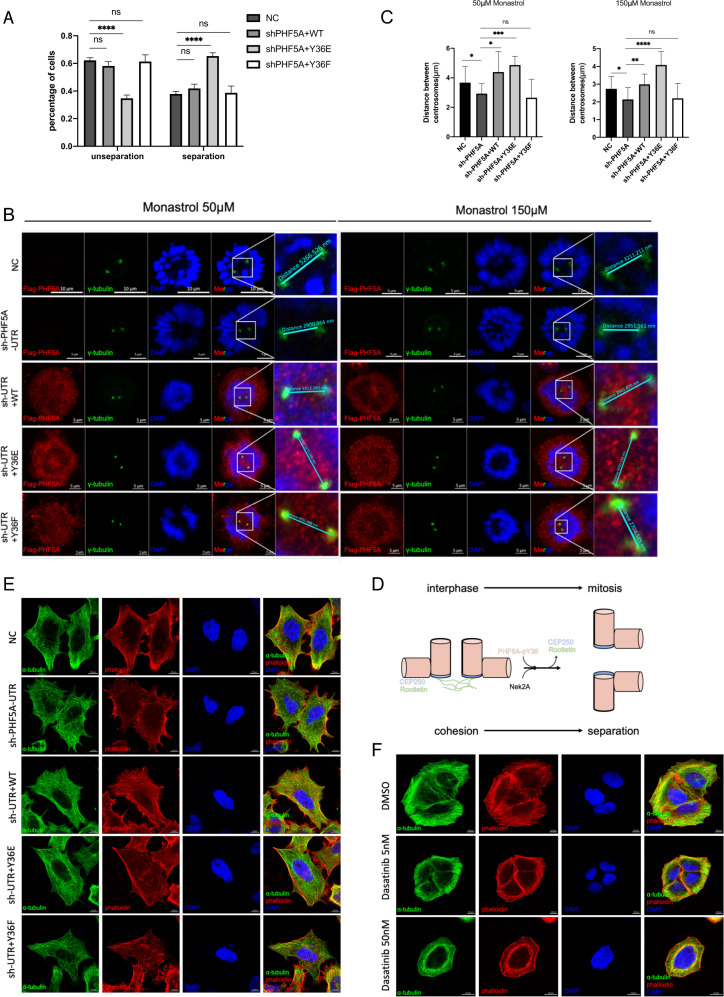
pY36-PHF5A locates in the centrosome to promote centrosome separation and microtubule organization. **A** HeLa cells were infected with sh-PHF5A-UTR lentivirus and recued with PHF5A (wild-type, Y36E and Y36F). Inter-centrosome distances were measured from cells in interphase (*n* = 30). **B**, **C** Sh-PHF5A and sh-rescued HeLa cells were synchronized with thymidine block and released to enrich cells in the G2 phase (*n* = 20). Monastrol treatment inhibited Eg5-dependent centrosome splitting. Immunostaining was performed (**B**) and analyzed (**C**) to show the inter-centrosome distances. **D** Model for PHF5A promoting centrosome separation. **E**, **F** Sh-PHF5A, sh-rescued (**E**) or dasatinib treated (**F**) HeLa cells were labeled of microtubule or F-actin with anti-α-tubulin antibody or phalloidin. Immunostaining was performed to show the organization of the cytoskeleton.

We further investigated the role of PHF5A in regulating centrosome separation. The cells rescued by PHF5A-Y36E resulted in a significant increase in centrosome separation (Fig. 5A). After synchronizing to the late G2 phase and inactivating Eg5 with monastrol, cells rescued by PHF5A-Y36E showed longer inter-centrosome distances, same as the depletion of CEP250 (Fig. 5B, C and Fig. S5A). In contrast, PHF5A-Y36F rescued cells showed shorter inter-centrosome distances, the same as the depletion of Nek2A (Fig. 5B, C and Fig. S5A). The above results showed that PHF5A-Y36E promotes centrosome separation (Fig. 5D). Given that the centrosome is the most important microtubule organization center which contributes to cell migration, we examined whether PHF5A-Y36E or PHF5A-Y36F could affect microtubule attachment. The immunofluorescent staining showed that cells rescued by PHF5A-Y36F and the cells depleted with Nek2A or treatment with dasatinib contained a loosened microtubule and F-actin (Fig. 5E, F and Fig. S5B). In contrast, the cells rescued by PHF5A-Y36E and the cells depleted with CEP250 both showed a dense microtubule (Fig. 5E and Fig. S5B). The wound-healing assay showed that the treatment of dasatinib inhibited cell migration (Fig. S5C). All these data demonstrated that pY36-PHF5A promotes centrosome separation and microtubule remodeling.

### Y36-PHF5A is hyperphosphorylated in medulloblastoma

**Fig. 6:**
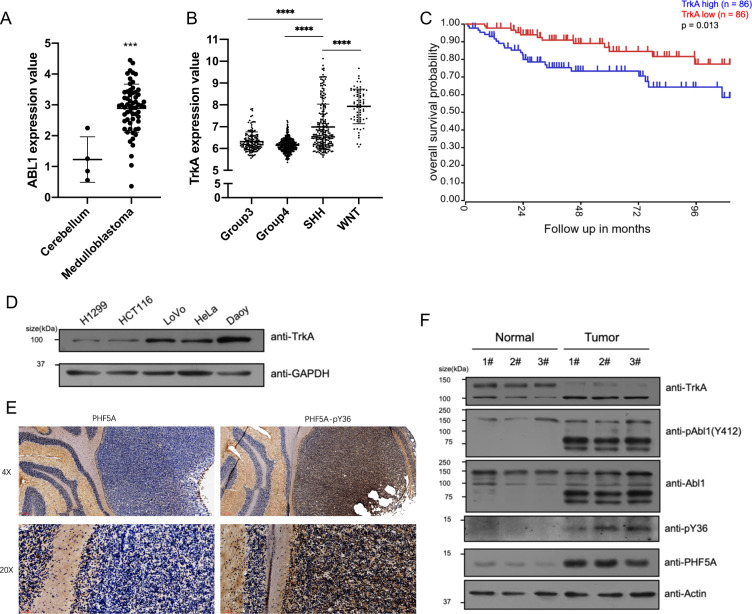
pY36-PHF5A hyper-phosphorylates in medulloblastoma. **A** ABL1 expression level was analyzed with a microarray of samples from Oncomine: normal cerebellum (*n* = 4) and medulloblastoma (*n* = 67). **B** TrkA expression level was analyzed with microarray analysis of 763 primary medulloblastomas from GEO (GSE85217): Group 3 (*n* = 144), Group 4 (*n* = 326), SHH (*n* = 223), and WNT (*n* = 70). **C** Kaplan–Meier analysis was performed in two groups of Shh-subtype medulloblastomas from GEO (GSE85217) for TrkA expression. **D** Indicated cell lines were used to verify the expression level of TrkA. **E** Immunohistochemistry staining for PHF5A and pY36-PHF5A in Shh-subtype medulloblastoma mouse model. **F** Immunoblotting was performed in normal cerebellum or medulloblastoma of Shh-subtype mouse model with indicated antibodies.

Since both ABL1 and TrkA are protooncogenes, we wondered whether the TrkA-ABL1-PHF5A cascade could function in tumorigenesis or tumor progression. Because the TrkA is activated by NGF, we focused on the tumors from the nervous system. The database showed that ABL1 is overexpressed in large-scale medulloblastoma (Fig. 6A) (Oncomine [44, 45]). Meanwhile, TrkA is overexpressed in the Shh-subtype and Wnt-subtype of medulloblastoma (GSE85217) (Fig. 6B). Furthermore, in Shh-subtype medulloblastoma, the TrkA high expression patient group corresponded to a lower overall survival rate in comparison with the low expression patient group (GSE85217 [46]) (Fig. 6C). We examined several cancer cell lines for TrkA expression levels and found that Daoy cell line, a human Shh-type medulloblastoma cell line, had the highest expression levels of TrkA (Fig. 6D). We also observed the high pY36-PHF5A levels in the tumor tissues of the medulloblastoma mouse model (Fig. 6E). We also verified the activation of TrkA-ABL1 cascade and the hyperphosphorylation of PHF5A at Y36 in the Shh-type medulloblastoma mouse model (Fig. 6F). All these data demonstrated that PHF5A is hyperphosphorylated at Y36 in medulloblastoma.

### pY36-PHF5A promotes medulloblastoma proliferation and migration by regulating centrosome separation and microtubule remodeling

**Fig. 7:**
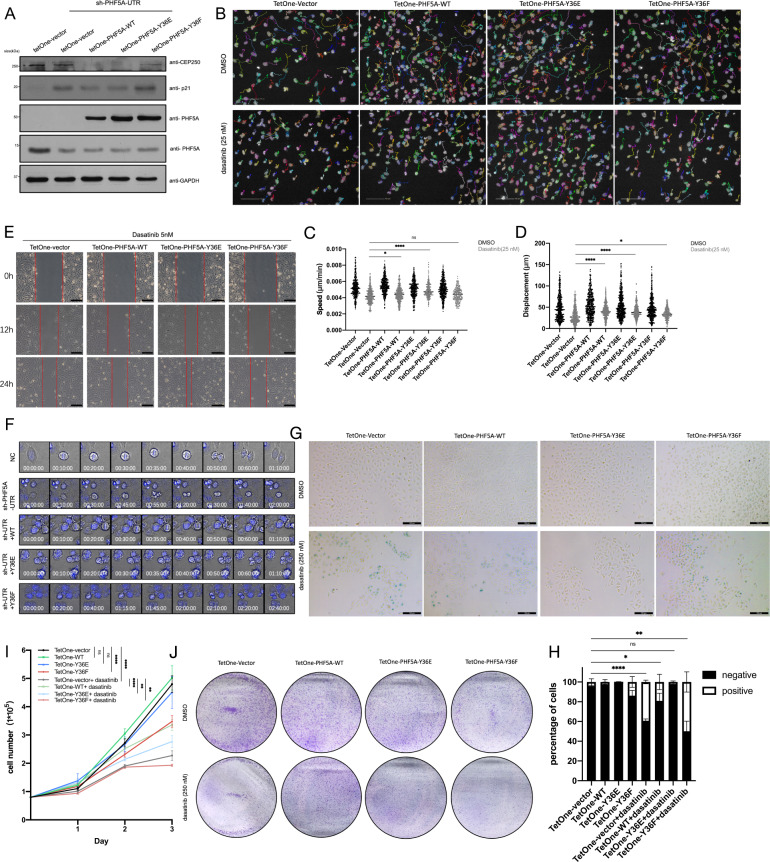
pY36-PHF5A promotes medulloblastoma migration through centrosome separation and microtubule remodeling. **A** Immunoblotting analysis of CEP250 in PHF5A-WT, Y36E, or Y36F-DAOY-rescued cells. **B** Cell migration assays were performed in TetOne-DAOY cells (WT, Y36E or Y36F) treated with or without dasatinib (25 nM). Images were captured every 5 min. Scale bars: 100 μm. **C**, **D** Cell migration speed (**C**) and displacement (**D**) were measured with cell migration assay (Harmony software). **E** Wound-healing assays were performed in TetOne-DAOY cells (WT, Y36E or Y36F) treated with dasatinib (5 nM). Images were captured at times 0, 12, and 24 h. Scale bars: 100 μm. **F** Cell mitotic images were captured in PHF5A-WT, Y36E, or Y36F-shHela-rescued cells after being released from thymidine synchronization. Images were captured every 5 min. **G**, **H** SA-β-gal staining was performed in TetOne-DAOY cells (WT, Y36E or Y36F) treated with or without dasatinib (250 nM) (**G**). The percentage of positive staining cells were calculated (**H**). **I** DAOY cells were seeded into six-well plates at the same number with or without the treatment of dasatinib (250 nM). Cell numbers were counted every day (*n* = 5). Scale bars: 100 μm. **J** Colony formation assay was performed in TetOne-DAOY cells (WT, Y36E or Y36F) treated with or without dasatinib (250 nM).

To further gain insight into the function of PHF5A in medulloblastoma, we first verified that downregulation of ABL1 reduced pY36-PHF5A level in centrosome (Fig. S6A), and pY36-PHF5A decreased the protein level of CEP250 in Daoy cells (Fig. 7A and Fig. S6B). Cells rescued by PHF5A-WT and PHF5A-Y36E increased the ratio of centrosome separation while cells rescued by PHF5A-Y36F decreased the ratio of centrosome separation in interphase Daoy cells (Fig. S6C, D). Downregulation of pY36-PHF5A by treating cells with TrkA inhibitor taletrectinib or ABL1 inhibitor dasatinib also decreased the ratio of centrosome separation in interphase Daoy cells (Fig. S6E, F). Additionally, we observed the loosened microtubule arrangement in PHF5A-Y36F rescued cells (Fig. S6G) or treatment with low-dose of dasatinib (Fig. S6H). The migration assay showed that low-dose of dasatinib inhibited the migration speed (Fig. 7B, C) and displacement (Fig. 7D) of Daoy cells, however, PHF5A-Y36E could rescue the inhibitory phenomenon of dasatinib (Fig. 7B–D). The wound-healing assay showed that the treatment of dasatinib inhibited the cell migration (Fig. S6I), in contrast, PHF5A-Y36E also could recuse this inhibitory phenomenon of dasatinib (Fig. 7E). Indeed, the video showed that the treatment of both low-dose of dasatinib and taletrectinib could inhibit the migration of medulloblastoma cells with little effect on their proliferation (Supplemental Movies S1–S4 and Fig. S6J). All these data demonstrated that pY36-PHF5A promotes medulloblastoma migration by regulating centrosome separation and microtubule remodeling.

In mitotic cells, the centrosome plays a critical role in spindle formation to ensure the correct cell division. To investigate the function of pY36-PHF5A during mitosis, we used time-lapse microscopy to record the mitotic process of the cells that knockdown PHF5A without or with the rescue. The result showed that the cells that knockdown of PHF5A displayed triple polarity cell division and cytokinesis failure (Fig. 7F), while the rescue of PHF5A-WT or PHF5A-Y36E can reverse this phenotype (Fig. 7F). However, the cells rescued of PHF5A-Y36F still displayed cell cycle arrest (Fig. 7F). Also, the cells rescued by either PHF5A-Y36F or PHF5A-Y36E couldn’t avoid the irreversible cell death induced by PHF5A knockdown in a long period of cell culture (Fig. S6K). To investigate if the cell cycle arrest in PHF5A-Y36F rescued cells induced cellular senescence, SA-β-Gal staining was performed in cells which overexpressed PHF5A mimics. The results showed that the cellular senescence was indeed induced by the treatment of high-dose of dasatinib or overexpression of PHF5A-Y36F (Fig. 7G, H). To determine whether PHF5A-pY36 affects cell proliferation, colony formation assay and cell number counting assay were performed in cells overexpressing PHF5A with or without the treatment of high-dose of dasatinib. The results showed that the treatment of high-dose of dasatinib inhibited the proliferation of control and PHF5A-Y36F rescued cells but the cells rescued by PHF5A-WT and PHF5A-Y36E can resume cell proliferation (Fig. 7I, J). All these data demonstrated that pY36-PHF5A promotes medulloblastoma proliferation and rescues cellular senescence induced by dasatinib by regulating the cell cycle process.

## Discussion

It is well-established that the TrkA pathway functions in neurogenesis, cell differentiation, and proliferation. Also, TrkA, as well as ABL1, could be a pro-oncogene function in tumorigenesis [47, 48]. According to recent reports, with the stimulation of nerve growth factor, TrkA was activated and induced downstream phosphorylation cascade to promote cell proliferation [49]. In the canonical growth factor phosphorylation cascade, the activated Mst2 can phosphorylate and active Nek2A, which directly induces the centrosome separation [50]. In the present study, we provided evidence that ABL1 was recruited to the centrosome when the TrkA-ERK1/2-ABL1 cascade was activated. Activated ABL1 phosphorylated PHF5A at site Y36 in the centrosome. The hyperphosphorylated PHF5A-Y36 was observed in medulloblastoma with the activation of the TrkA-ABL1 cascade which subsequently promoted cell migration. The treatment of inhibitors of both TrkA and ABL1 could inhibit the migration of medulloblastoma cells. In contrast, the cells rescued by PHF5A-Y36E could abolish the migration inhibitory phenomenon induced by dasatinib. Our results demonstrated that phosphorylation of PHF5A by TrkA-ERK1/2-ABL1 cascade plays an important role in regulating centrosome function.

PHF5A is well characterized as a component of spliceosome U2 snRNPs. During pre-mRNA splicing, PHF5A interacts with SF3B1 for branch point recognition. However, increasing evidence suggests that PHF5A also could play spliceosome-independent functions. It stabilizes the Paf1 transcriptional complex in pluripotent embryonic stem cells' self-renewal and maintenance of pluripotency [27]. It stabilizes the p400 histone chaperone complex and functions in DNA repair [16]. Our findings on PHF5A regulating centrosome function by affecting the interaction of CEP250 and Nek2A supported the notion that it plays a spliceosome-independent function.

The other components of spliceosomes have been reported to regulate cellular events. PQBP1, a splicing regulator protein, interacts with Dynamin 2 to orchestrate neuronal ciliary morphogenesis in the brain [51]. Pre-mRNA processing factors (PRPF6, PRPF8, and PRPF31) mutated in autosomal dominant retinitis pigmentosa and were identified as regulators during ciliogenesis [52]. In the current study, we identified a previously unrecognized centrosomal localization and function of PHF5A during centrosome separation. Our study further confirmed that the spliceosome components regulate functions in various cellular processes.

During interphase, a newly duplicated pair of centrosomes are connected by a centrosome linker composed of CEP250, rootletin, CEP68 et al. At the onset of mitosis, the centrosome linker CEP250 is phosphorylated by the activated Nek2 and leads to its displacement, which initiates the centrosome separation. The precise timing of centrosome separation plays an important role in mitotic spindle formation and cell division, and correct segregation of chromosomes requires timely centrosome separation. Premature centrosome separation causes multipolar mitotic spindle formation and induces chromosome instability. Delay or unaccomplished centrosome separation will lead to multiple centrosomes or centrosome structure destruction which induces abnormal cell division and aneuploid. Importantly, the centrosome is the major microtubule organizing center in mammalian cells. The unstructured centrosome causes disassembling of microtubules which leads to the decrease of cellular substrate transportation, misplacing of organelles, and affecting cell migration. However, centrosome separation involves a sequence of events and regulation cascades that remain poorly understood. In this study, we demonstrated that pY36-PHF5A is located in the centrosome and mediated centrosome separation by promoting the interaction between CEP250 and Nek2A. These results increased our understanding of the regulation of centrosome separation. However, the regulation of CEP250 stability remains elusive, whether pY36-PHF5A participates in this regulation is worth further investigation.

In summary, our study revealed that phosphorylation of PHF5A-Y36 in the centrosome promotes centrosome linker displacement. The unmatured centrosome separation leads to a remodeling of the microtubule, subsequently regulating cell migration. In medulloblastoma, the hyperphosphorylation of the TrkA-ABL1-PHF5A cascade could regulate cancer proliferation and migration, which provides potential targets for cancer therapy.

## Supplementary information

Supplementary figure 1

Supplementary figure 2

supplementary figure3

supplementary figure4

supplementary figure5

supplementary figure6

Supplementary figure legends

Table 1

Supplemental Movie S1.

Supplemental Movie S2.

Supplemental Movie S3.

Supplemental Movie S4.

Original Data File

aj-checklist

## Data Availability

All datasets generated and analyzed during this study are included in this published article and its Supplementary Information files. Additional data were available from the corresponding author on reasonable request.
