# Supplementary figures and images for: The phosphorylation of PHF5A by TrkA-ERK1/2-ABL1 cascade regulates centrosome separation
Source: Cell Death Dis. 2023 Feb 9;14(2):98. doi: 10.1038/s41419-023-05561-1 (PMC9911754; doi:10.1038/s41419-023-05561-1)

Fig. S1

A

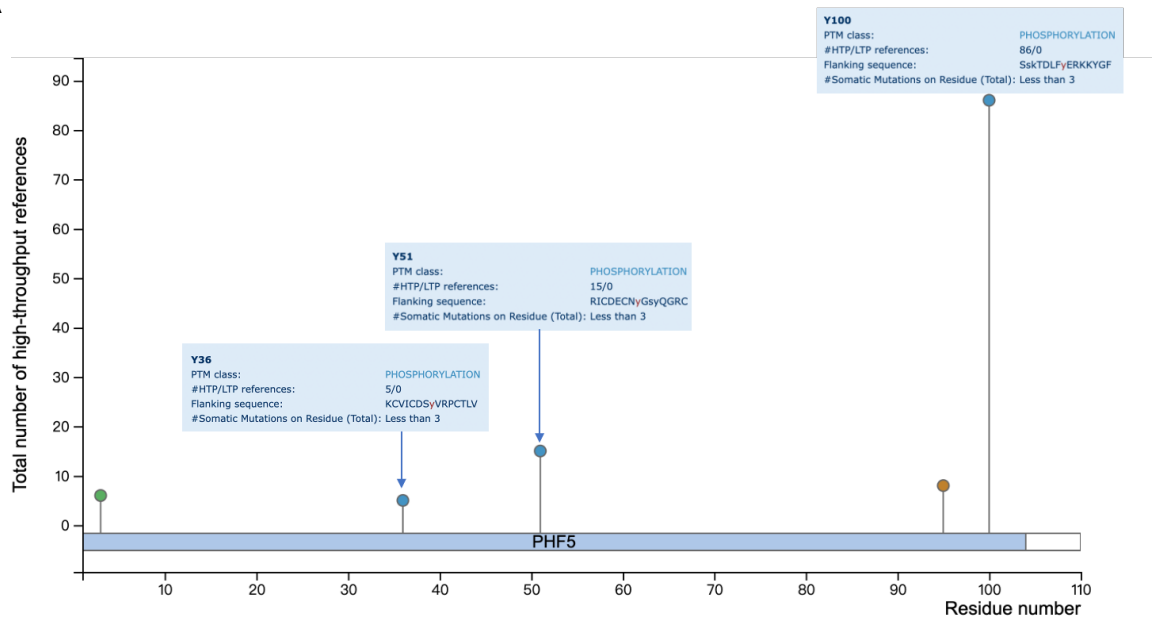

B

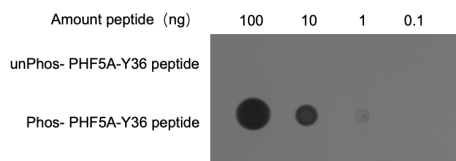

C

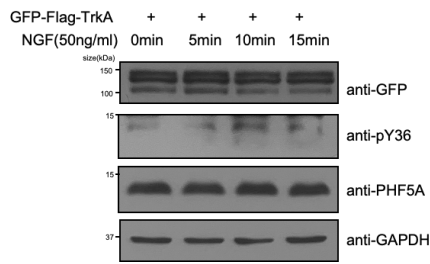

D

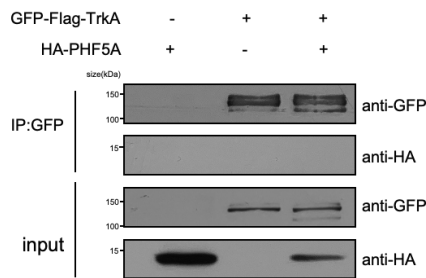

Supplement: Supplementary file 1 — Supplementary figure 1 [file 41419_2023_5561_MOESM1_ESM.pdf]

Fig. S2

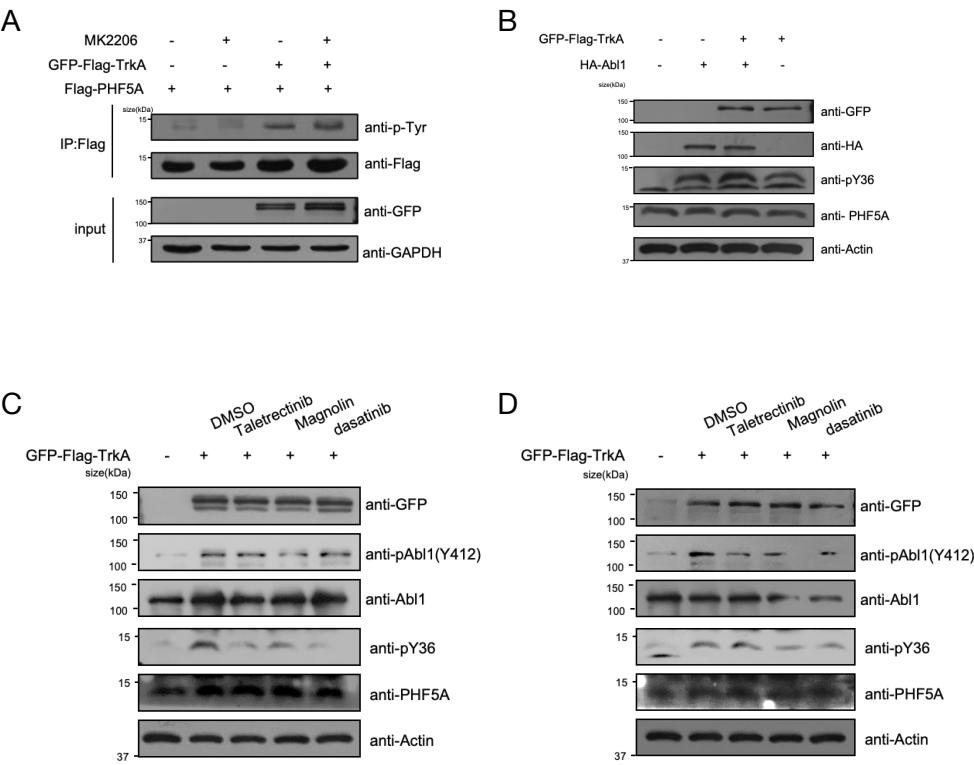

Supplement: Supplementary file 2 — Supplementary figure 2 [file 41419_2023_5561_MOESM2_ESM.pdf]

Fig. S3

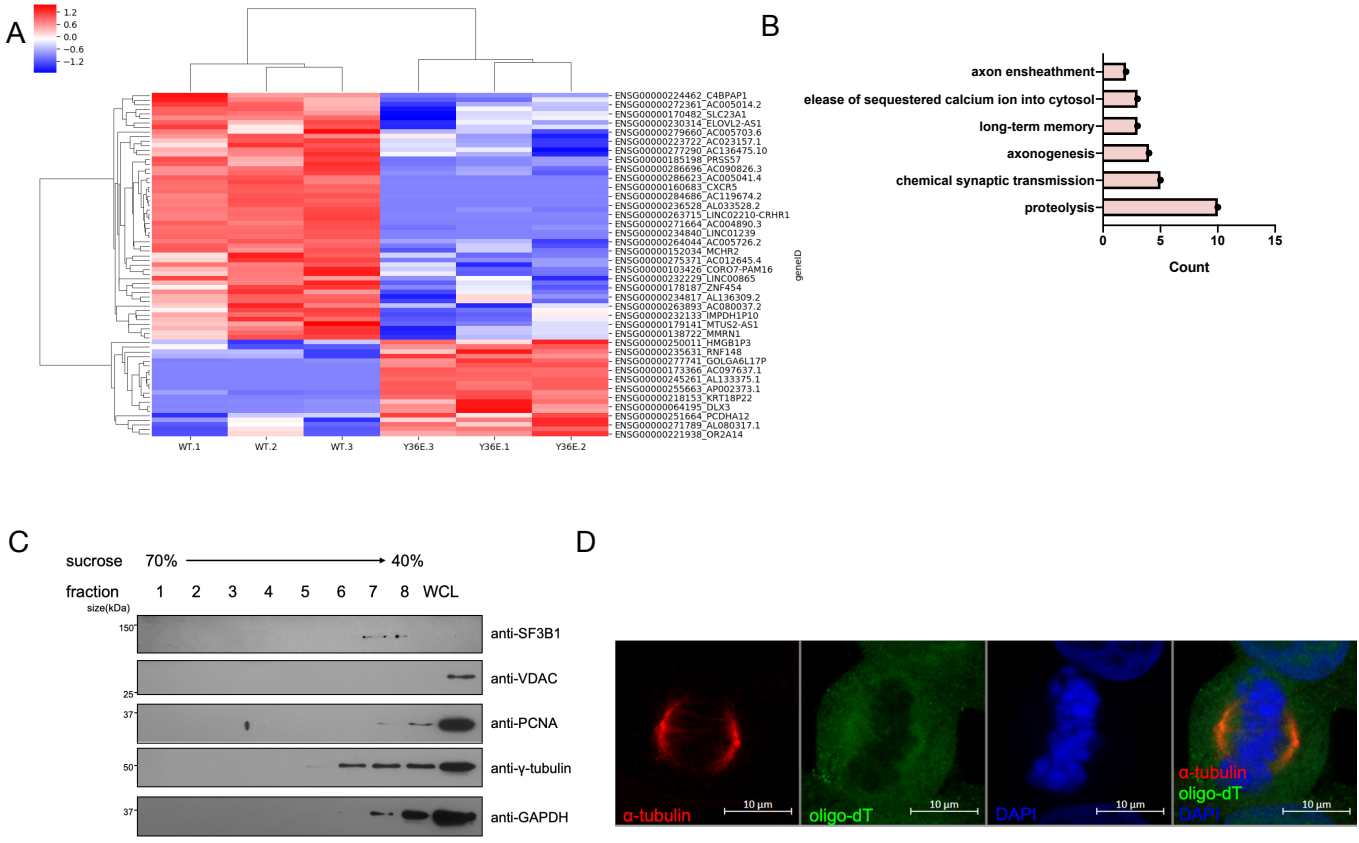

Supplement: Supplementary file 3 — supplementary figure3 [file 41419_2023_5561_MOESM3_ESM.pdf]

Fig. S4

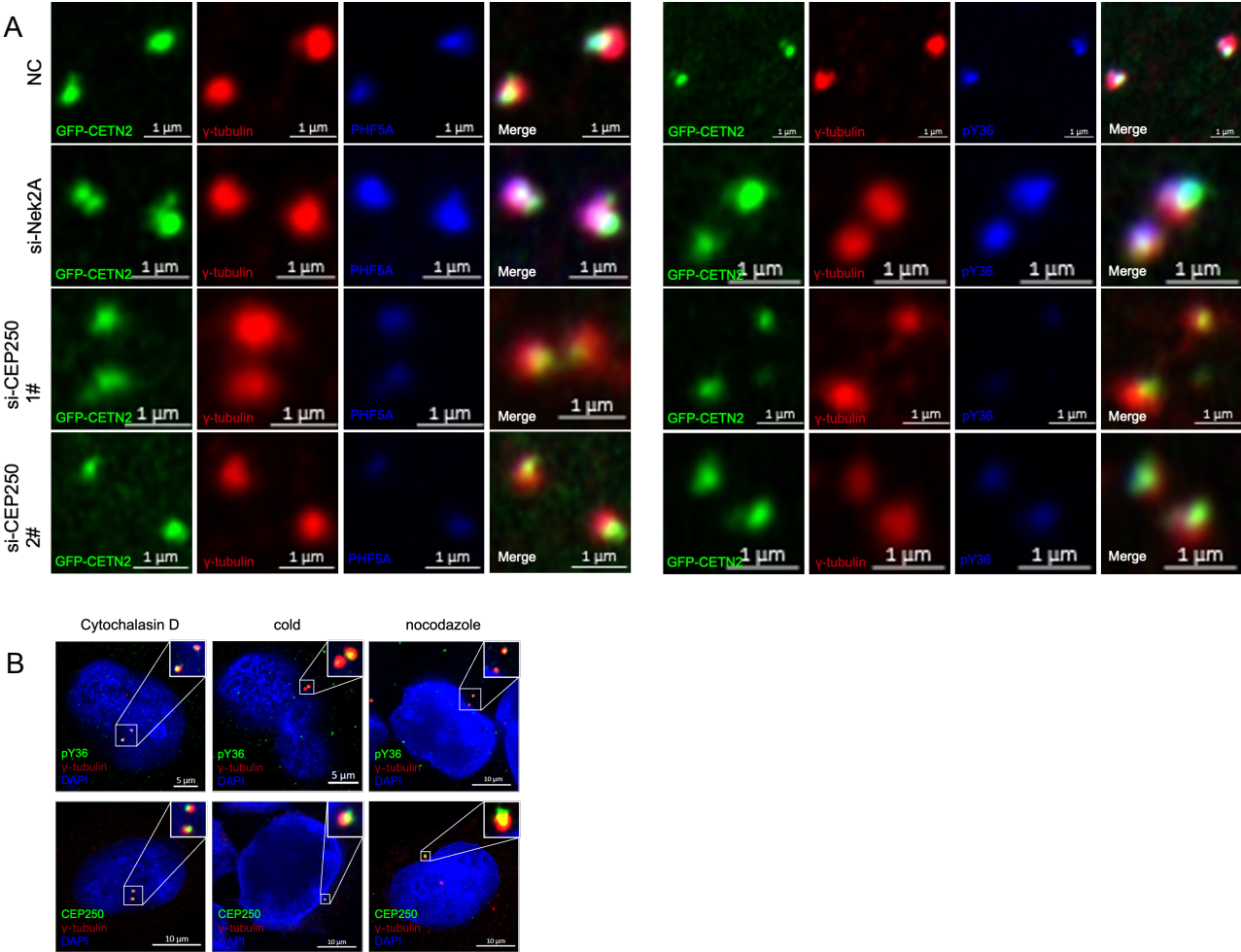

Supplement: Supplementary file 4 — supplementary figure4 [file 41419_2023_5561_MOESM4_ESM.pdf]

Fig. S5

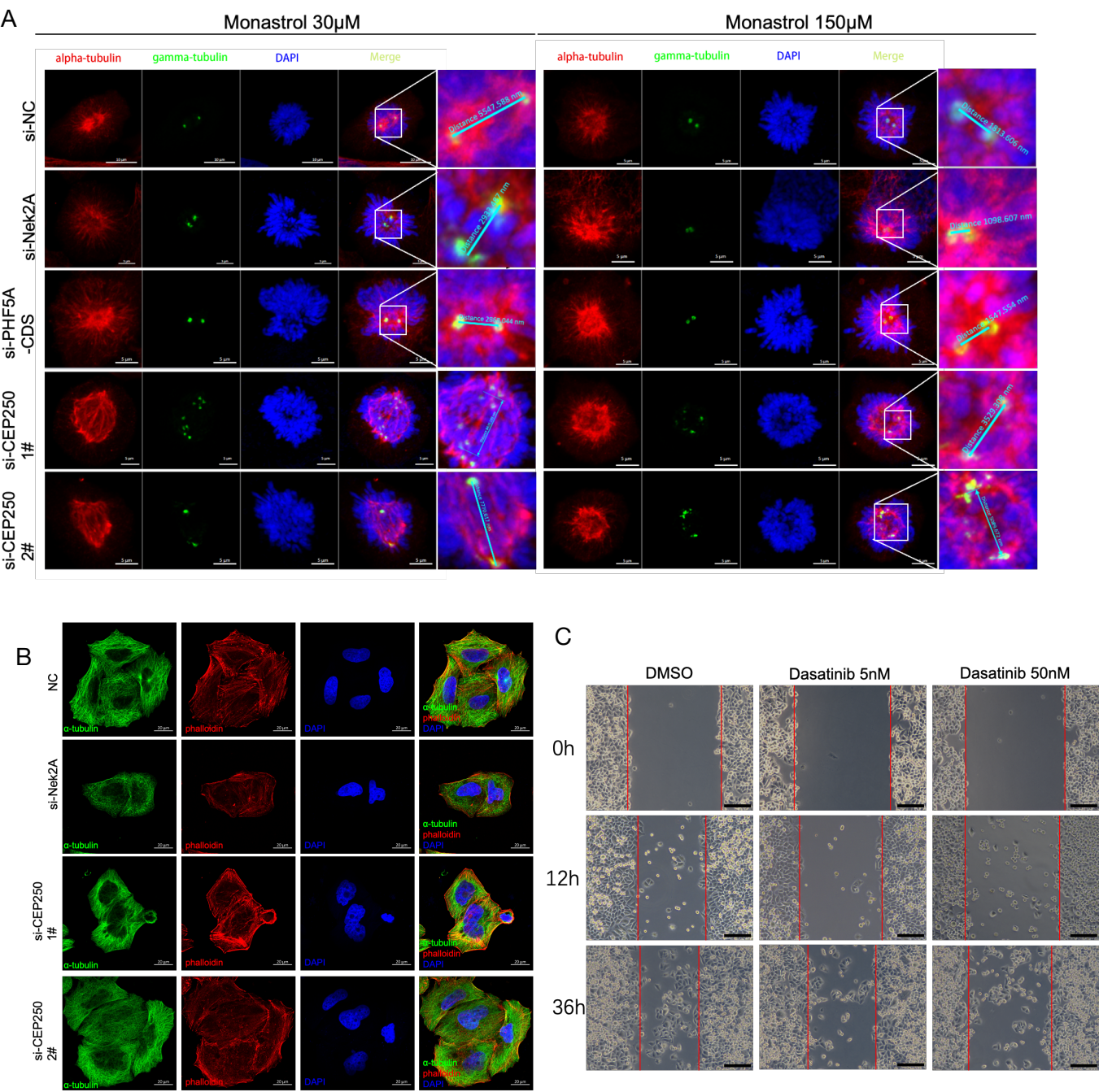

Supplement: Supplementary file 5 — supplementary figure5 [file 41419_2023_5561_MOESM5_ESM.pdf]

Fig. S6

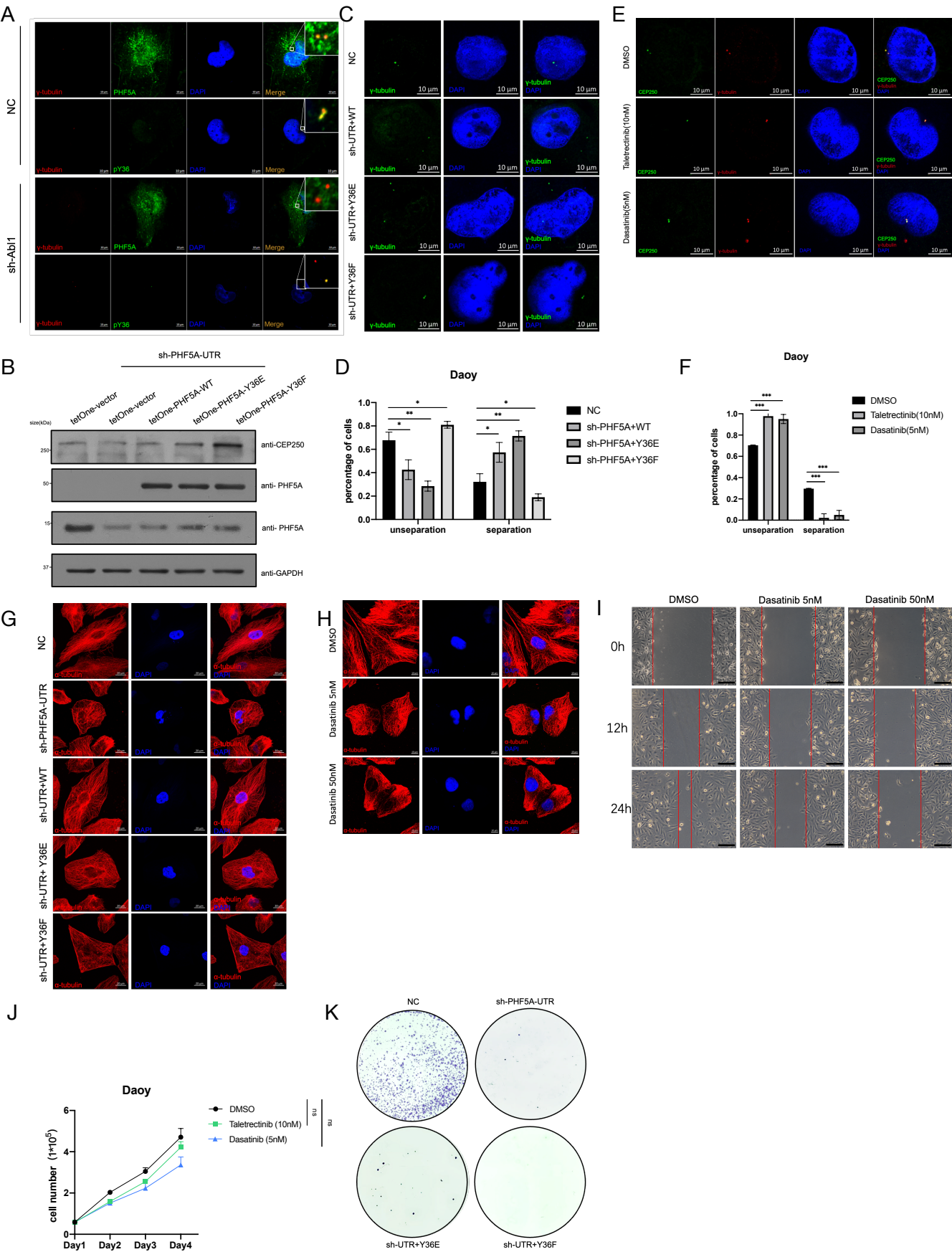

Supplement: Supplementary file 6 — supplementary figure6 [file 41419_2023_5561_MOESM6_ESM.pdf]
