# Supplementary material for: The phosphorylation of PHF5A by TrkA-ERK1/2-ABL1 cascade regulates centrosome separation
Source: Cell Death Dis. 2023 Feb 9;14(2):98. doi: 10.1038/s41419-023-05561-1 (PMC9911754; doi:10.1038/s41419-023-05561-1)
Supplement: Supplementary file 7 — Supplementary figure legends [file 41419_2023_5561_MOESM7_ESM.docx]

**Fig. S1**

A. Post-transcriptional modification of PHF5A investigated in PhosphoSitePlus. Blue dots showed the phosphorylation sites. Green dot showed the acetylation site. Brown dot showed the ubiquitination site. B. 100 ng, 10 ng, 1 ng or 0.1ng Y36 phosphorylated or unphosphorylated peptides were detected by PHF5A pY36 antibody. C. Endogenous pY36 was increased after NGF stimulation. After serum starvation of 16h, HEK293T cells were treated with NGF (50ng/ml) for indicated time periods with the overexpression of TrkA. D. HEK293T cells were transfected with indicated plasmids. Co-immunoprecipitation and immunoblotting were performed with indicate antibodies.

**Fig. S2**

A. HEK293T cells were transfected with indicated plasmids and treated with or without MK2206 (5 μM) for 1h. Immunoprecipitated Flag-PHF5A was detected by pan-tyrosine phosphorylation antibody. B. HEK293T cells were transfected with indicated plasmids. C and D. HeLa cells (C) and HEK293T cells (D) were treated with indicated inhibitors (taletrectinib 40nM, magnolin 60μM, dasatinib 50nM for 30 min) after overexpression of TrkA. Immunoblotting was performed with indicate antibodies.

**Fig. S3**

A-B. Heatmap (A) representation of differential expression analysis of genes in knocked down and rescued PHF5A WT or Y36E cells. Red and blue points mark the genes with significantly increased or decreased expression respectively in PHF5A WT compared to PHF5A Y36E. The bar graph (B) showed the enrichment analysis of the significant genes. C. After enriching centrosomal fractions by centrifugation, endogenous SF3B1 were measured in fractions of HEK293T whole cell lysates (WCL). D. FISH was perfoemed in Hela cells co-stained with anti-γ-tubulin antibodies and oligo-dT probe.

**Fig. S4**

A. The GFP-CETN2-HeLa cells, which were transfected with si-RNA to deplete Nek2A or CEP250, were co-stained with PHF5A (endogenous or pY36) and CEP250. B. Using cytochalasin D (1 μg/μL, 1h), cold (30min on ice) or nocodazole (0.2 μmol/μL, 1h) treat HeLa cells, immunofluorescence was performed with indicated antibodies.

**Fig. S5**

A. HeLa cells, which were transfected with indicated si-RNA, were synchronized with thymidine block and release to enrich cells in G2 phase (n=20). Monastrol treatment inhibited Eg5-dependent centrosome splitting. Immunostaining was performed to show the inter-centrosome distances. B. HeLa cells with or without si-CEP250 were labeled of microtubule or F-actin with anti-α-tubulin antibody or phalloidin. Immunostaining was performed to show the organization of cytoskeleton. C. Wound healing assays were performed in HeLa cells treated with or without dasatinib (5nM or 50nM). Images were captured at time 0 h, 12h and 24 h. Scale bars: 100μm.

**Fig. S6**

A. DAOY cells were infected with sh-Abl1 lentivirus or control lentivirus and stained with PHF5A or PHF-5A-pY36. B. Immunoblotting analysis of CEP250 in PHF5A-WT, Y36E or Y36F-Hela-rescued cells. C and D. Immunofluorescent staining (C) and inter-centrosome distances (D) were measured from PHF5A-WT, Y36E or Y36F-DAOY-rescued cells in interphase (n=30). E and F. Immunofluorescent staining (E) and inter-centrosome distances (F) were measured from DAOY cells treated with TrkA inhibitor taletrectinib (10nM) or dasatinib (5nM) in interphase (n=30). G and H. Sh-PHF5A, sh-rescued (G) or dasatinib treated (H) DAOY cells were labeled of microtubule with anti-α-tubulin antibody. Immunostaining was performed to show the organization of cytoskeleton. I. Wound healing assays were performed in DAOY cells treated with or without dasatinib (5nM or 50nM). Images were captured at time 0 h, 12h and 24 h. Scale bars: 100μm. J. DAOY cells were seeded into 6-well plates at the same number with or without the treatment of dasatinib (5nM) or taletrectinib (10nM). Cell numbers were counted every day (n = 5). K. Colony formation assay was performed in Hela cells knocked down with PHF5A and rescued with PHF5A-Y36E or PHF5A-Y36F.

**Movie S1.**

GFP-CETN2-DAOY cells treated with DMSO.

**Movie S2.**

GFP-CETN2-DAOY cells were treated with ABL1 inhibitor dasatinib (5nM).

**Movie S3.**

GFP-CETN2-DAOY cells were treated with ABL1 inhibitor dasatinib (25nM).

**Movie S4.**

GFP-CETN2-DAOY cells were treated with TrkA inhibitor taletrectinib (40nM).

**Table 1.**

Alternative splicing intron was analyzed with Leafcutter software in cell which knocked down PHF5A and rescued with PHF5A-WT or PHF5A-Y36E.
