# Supplementary material for: The phosphorylation of PHF5A by TrkA-ERK1/2-ABL1 cascade regulates centrosome separation
Source: Cell Death Dis. 2023 Feb 9;14(2):98. doi: 10.1038/s41419-023-05561-1 (PMC9911754; doi:10.1038/s41419-023-05561-1)
Supplement: Supplementary file 13 — Original Data File [file 41419_2023_5561_MOESM13_ESM.pdf]

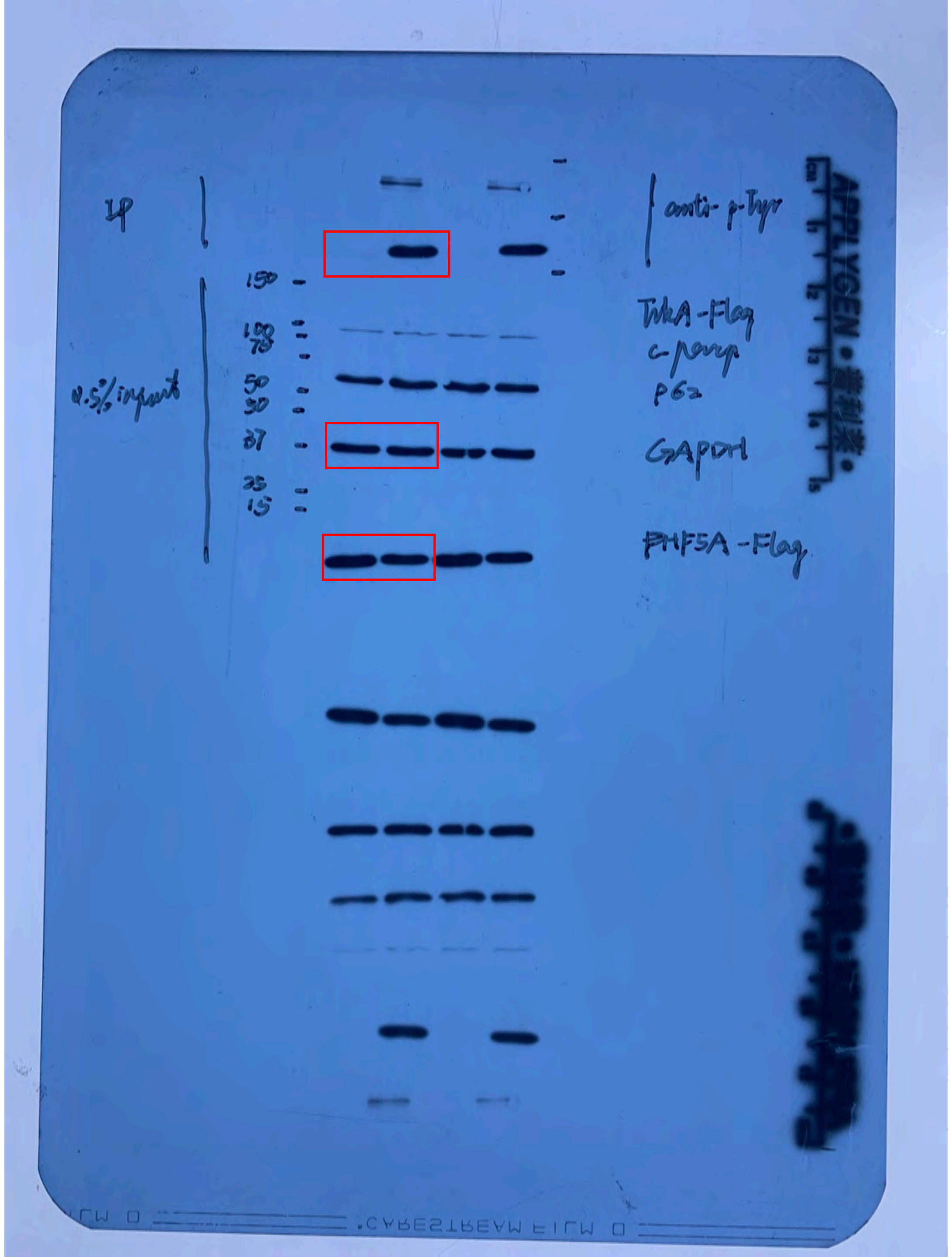
IP
anti-p-Tyr
0.5% input
150
50
37
TrkA-Flag
p62
GAPDH
PHF5A-Flag
APPLYGEN

Fig. 1b

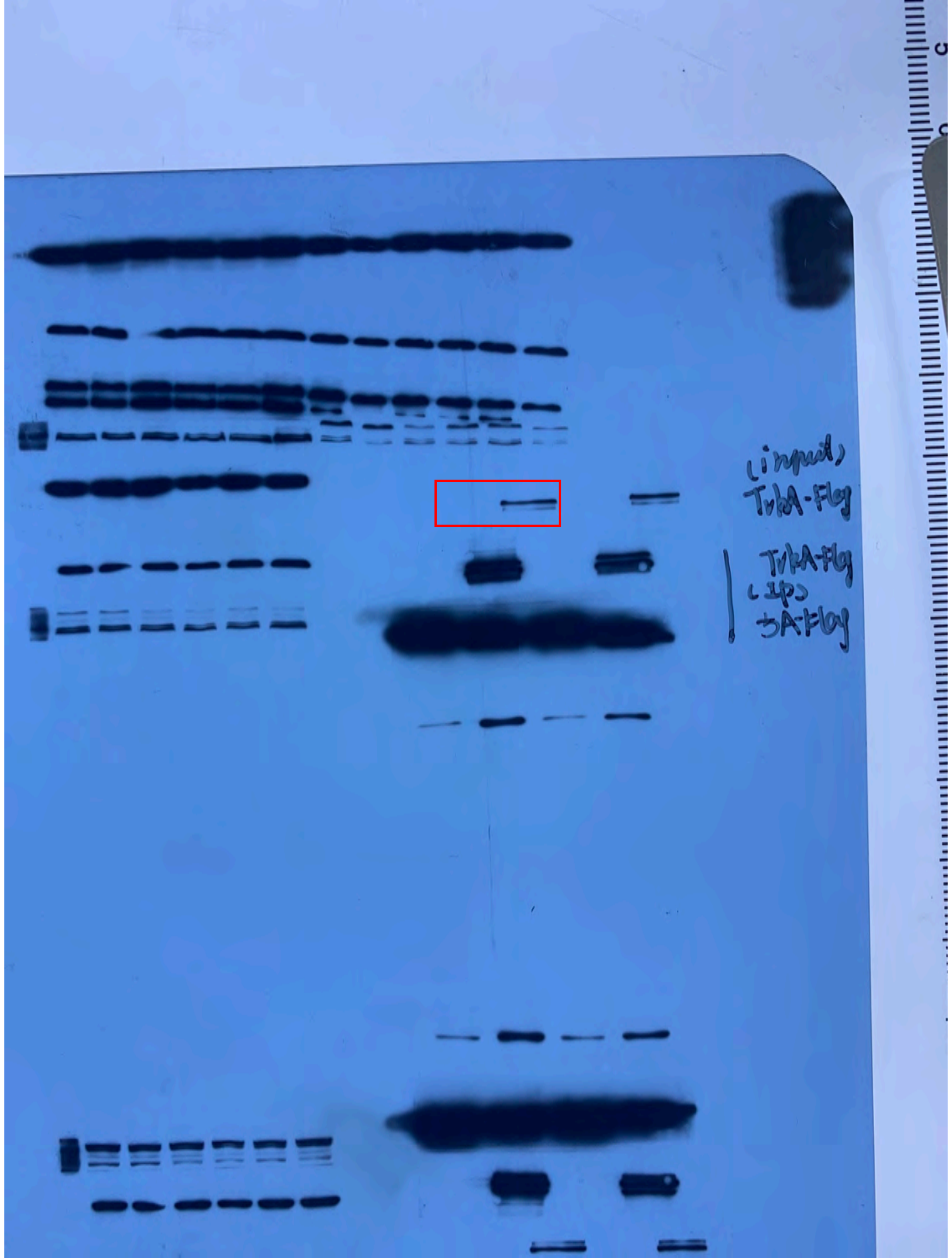

Fig. 1b

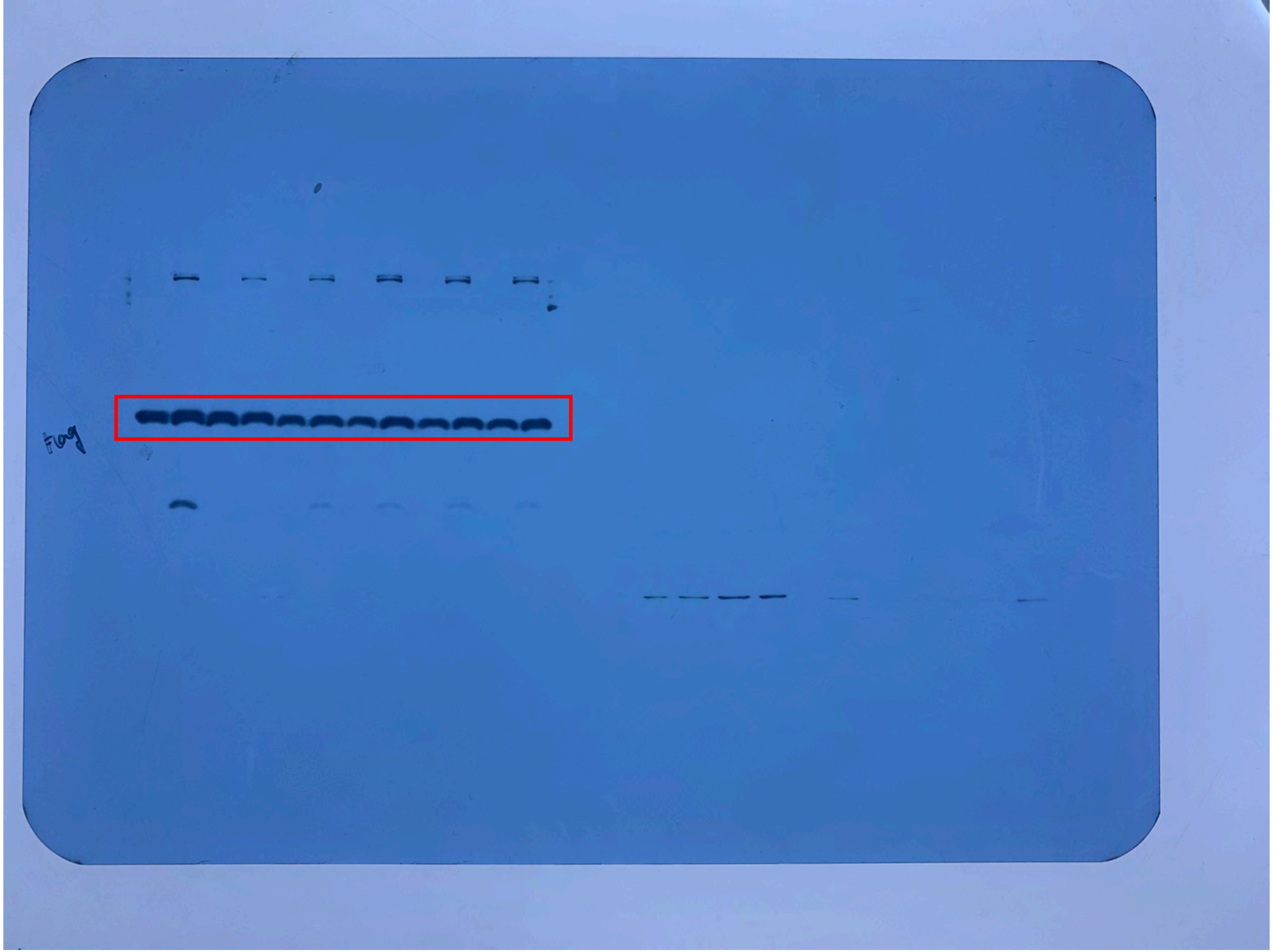

Fig. 1e

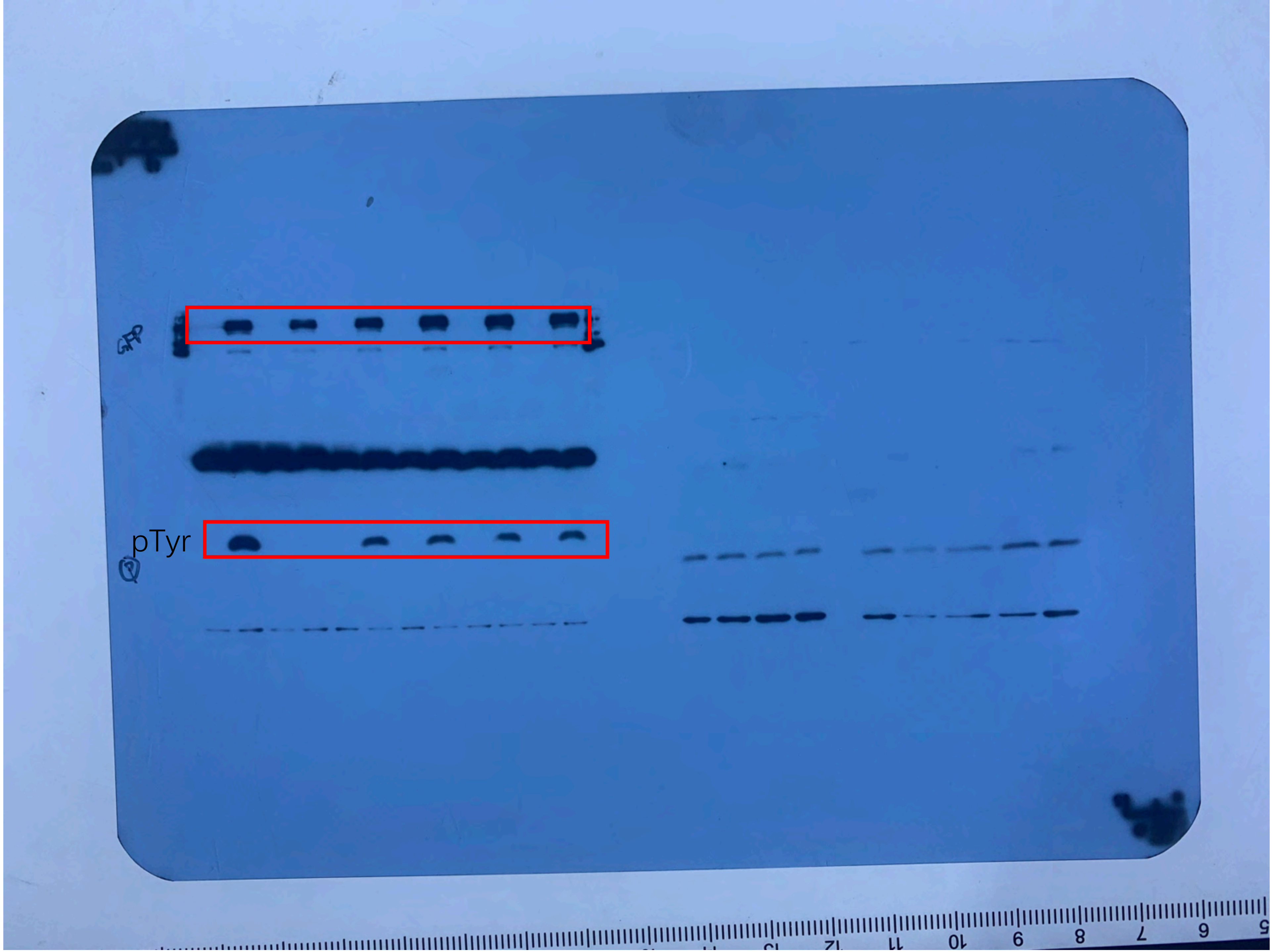

Fig. 1e

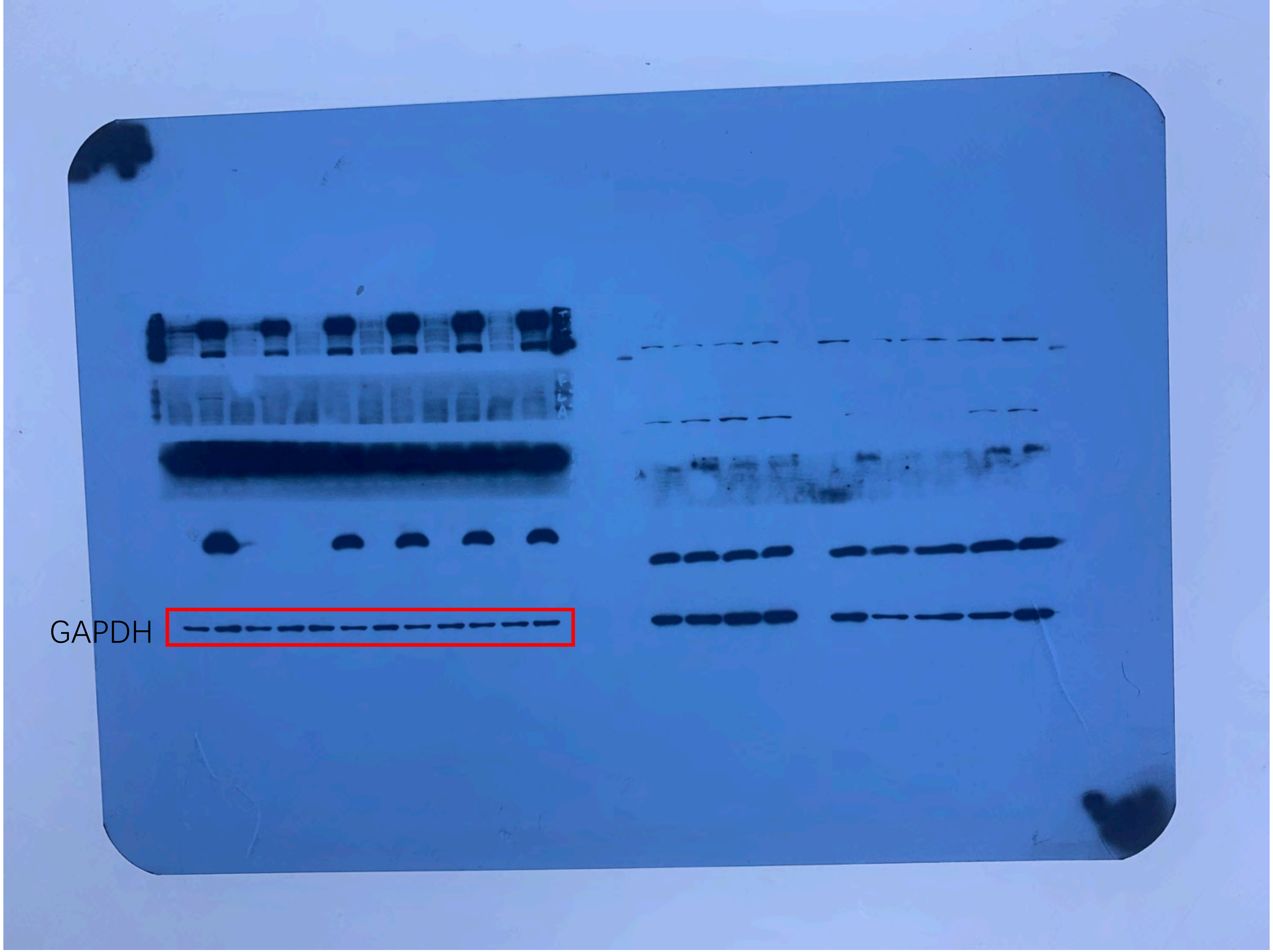

Fig. 1e

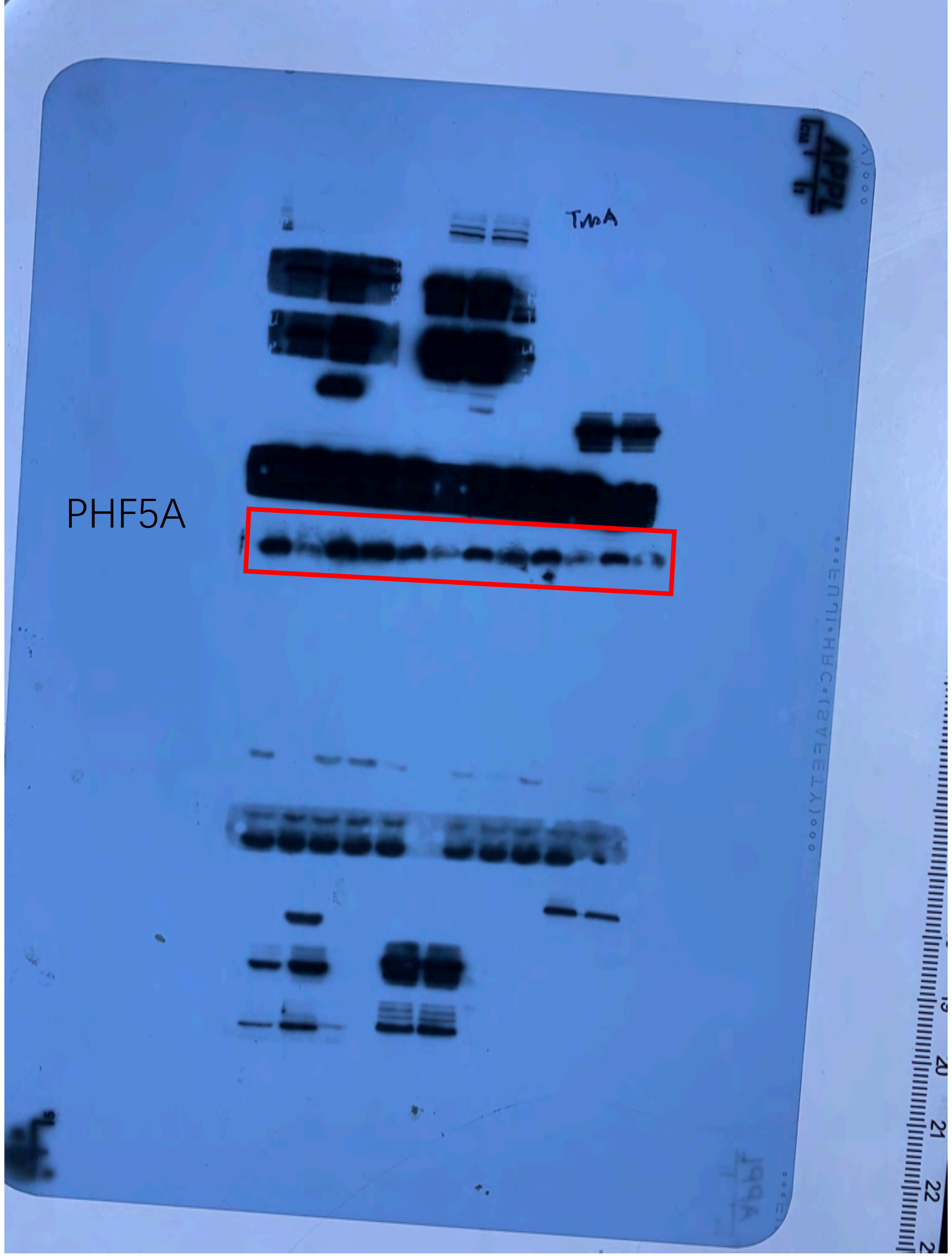

Fig. 1e

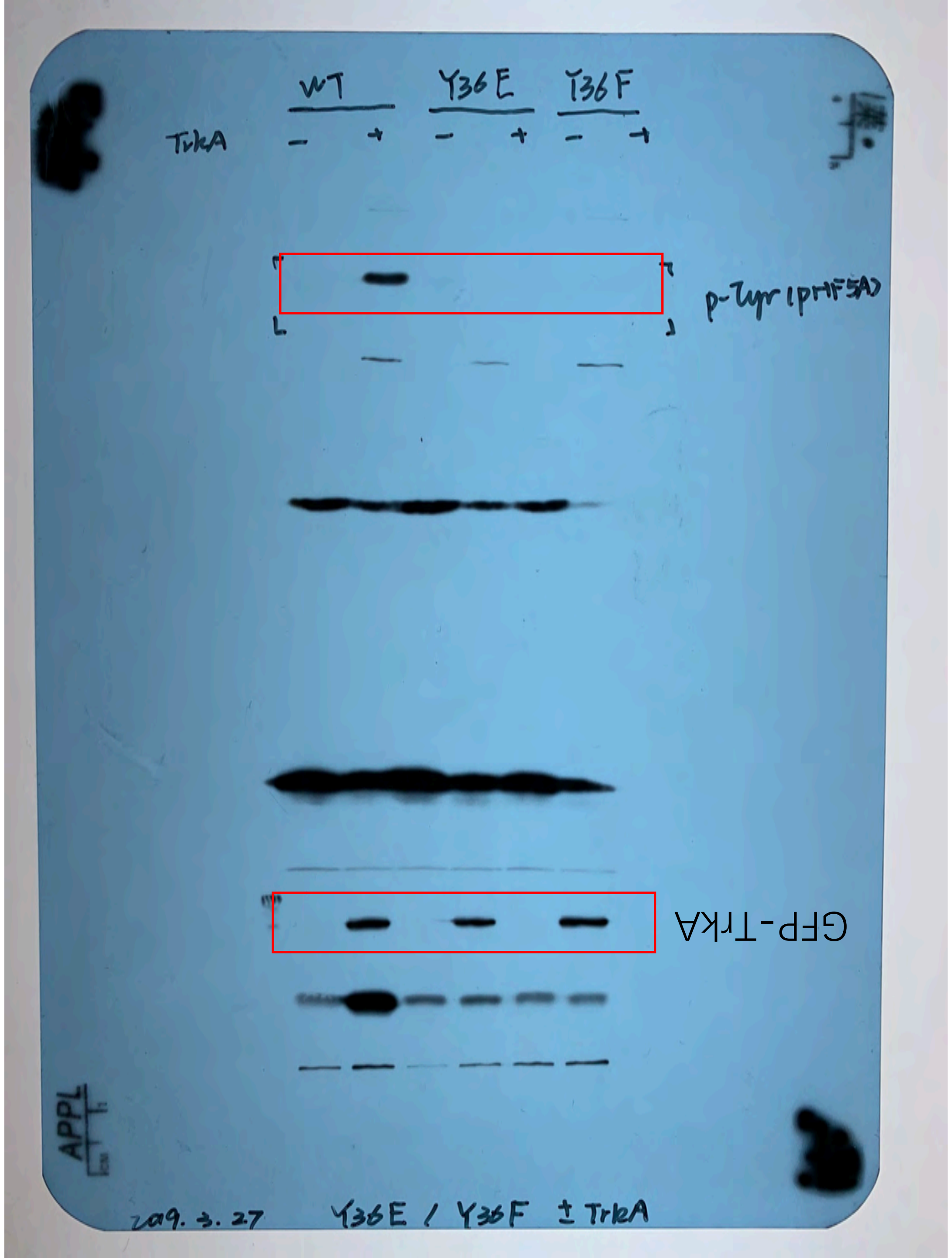

Fig. 1f

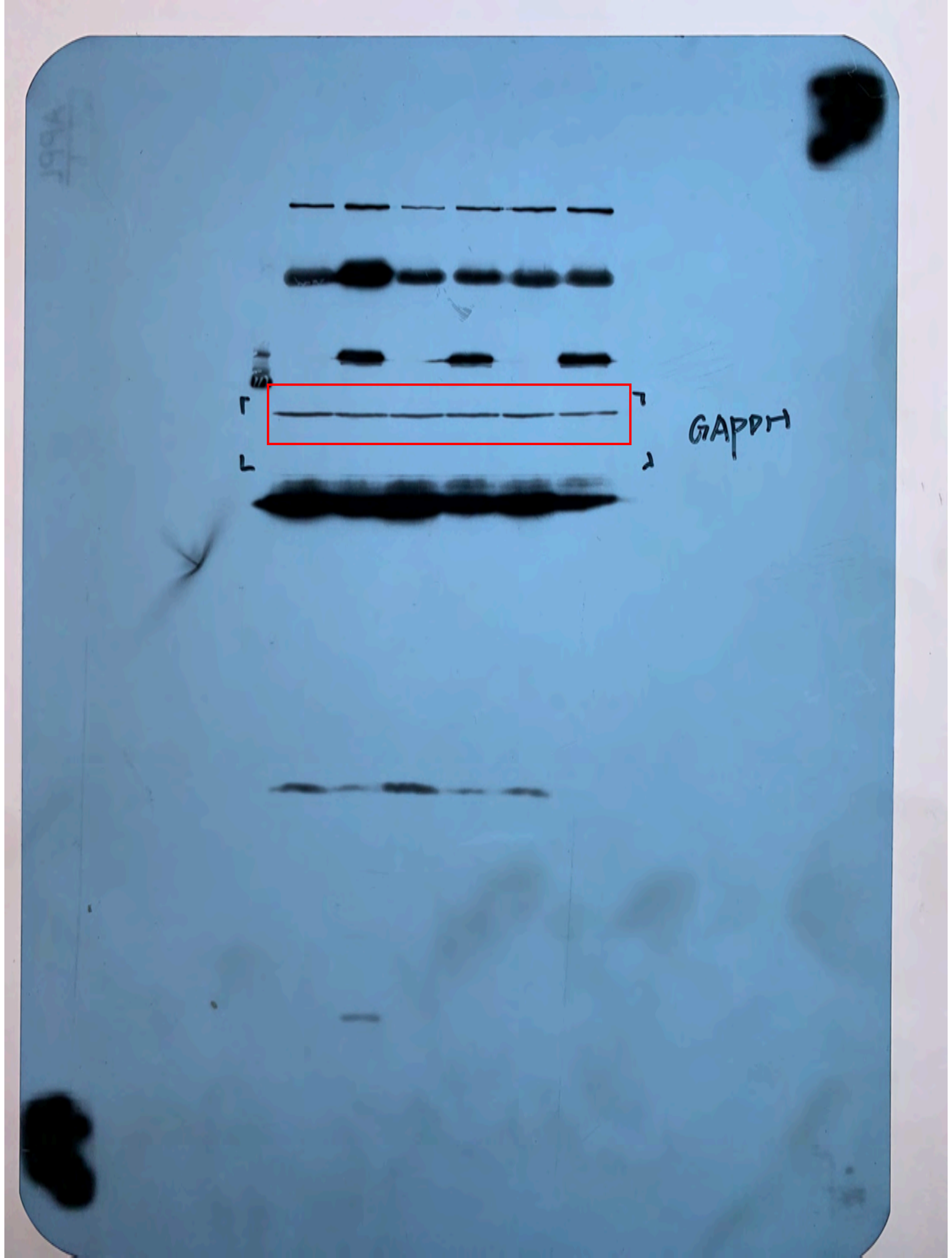

Fig. 1f

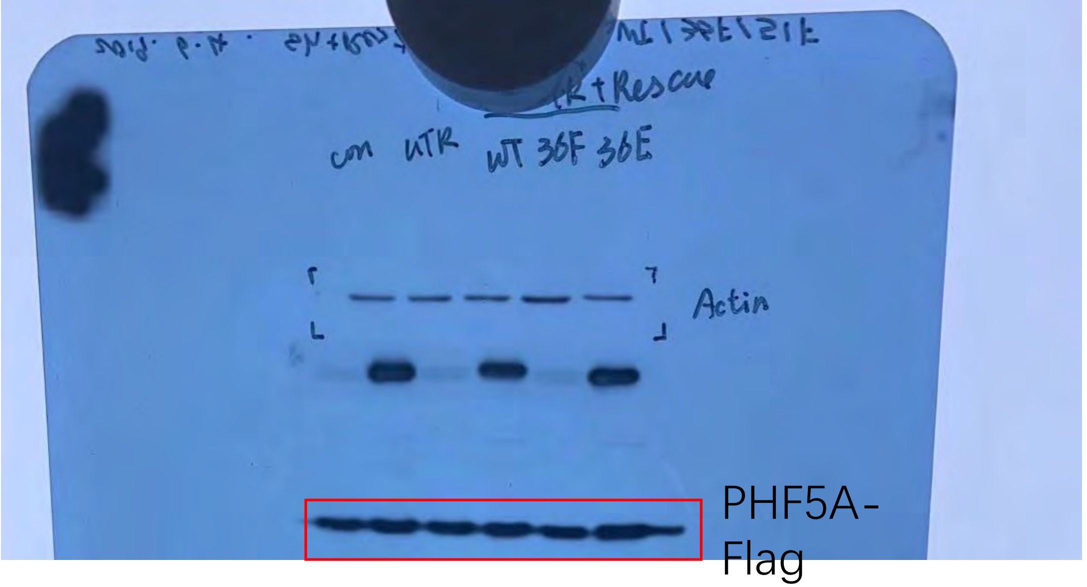

Fig. 1f

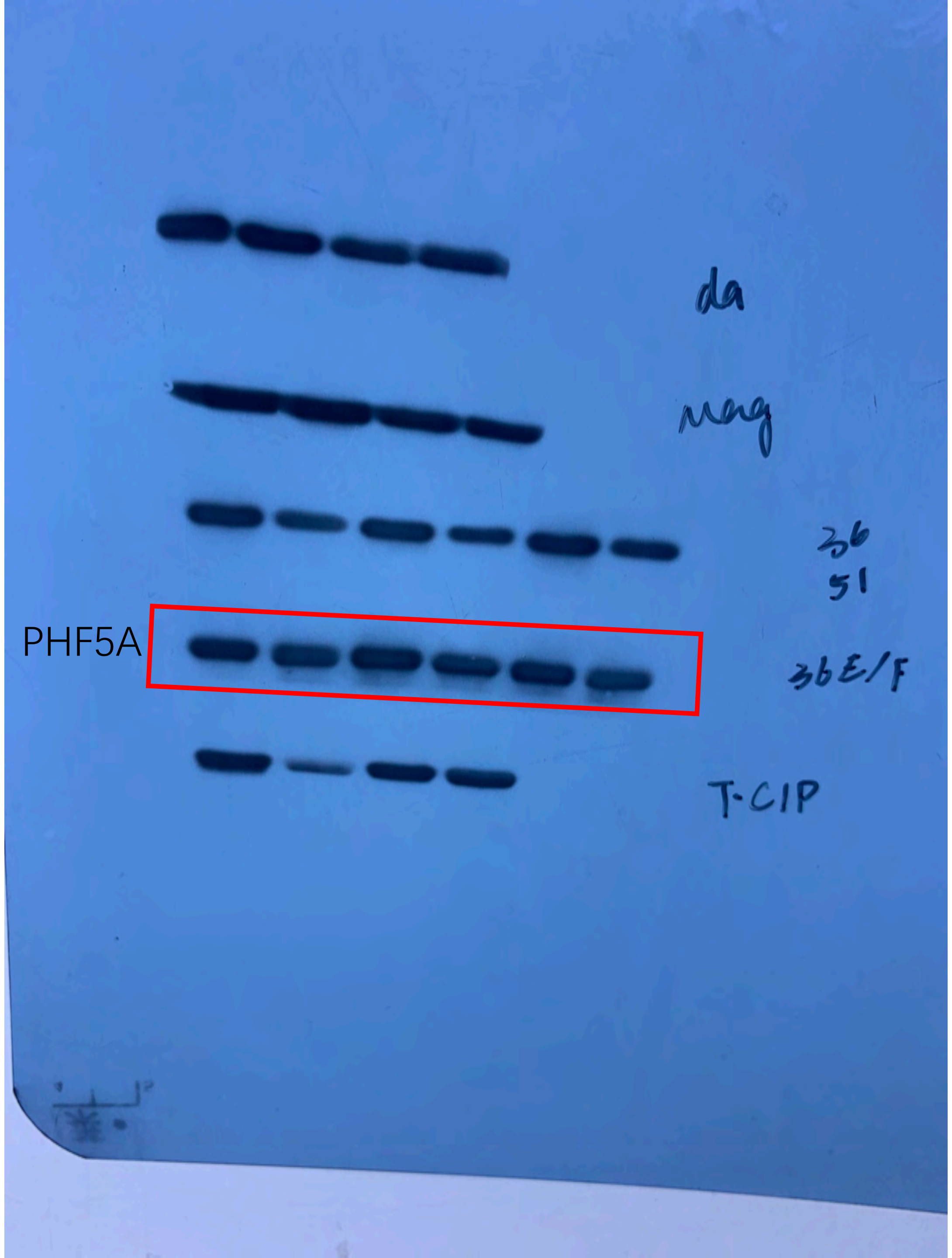

Fig. 1f

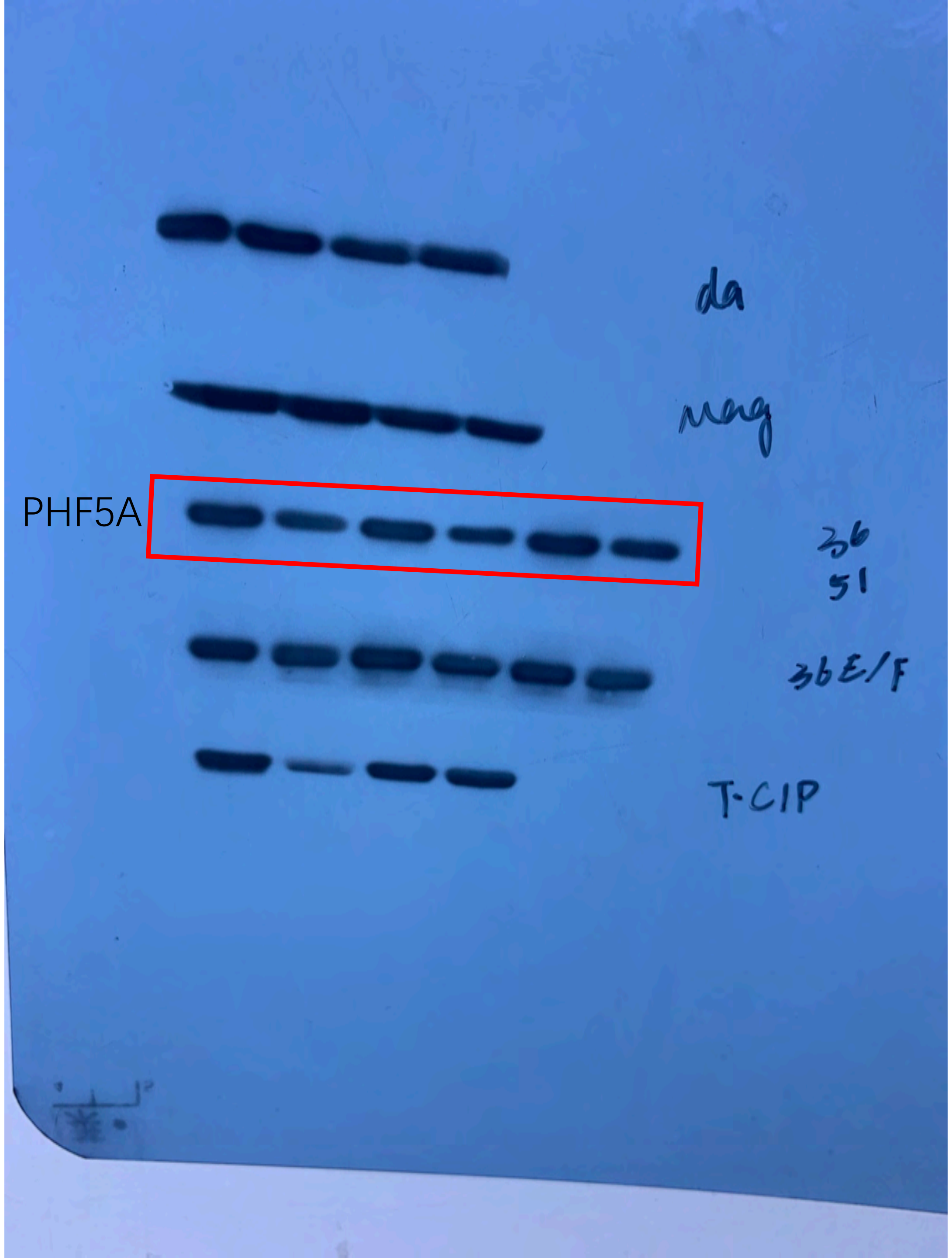

Fig. 1g

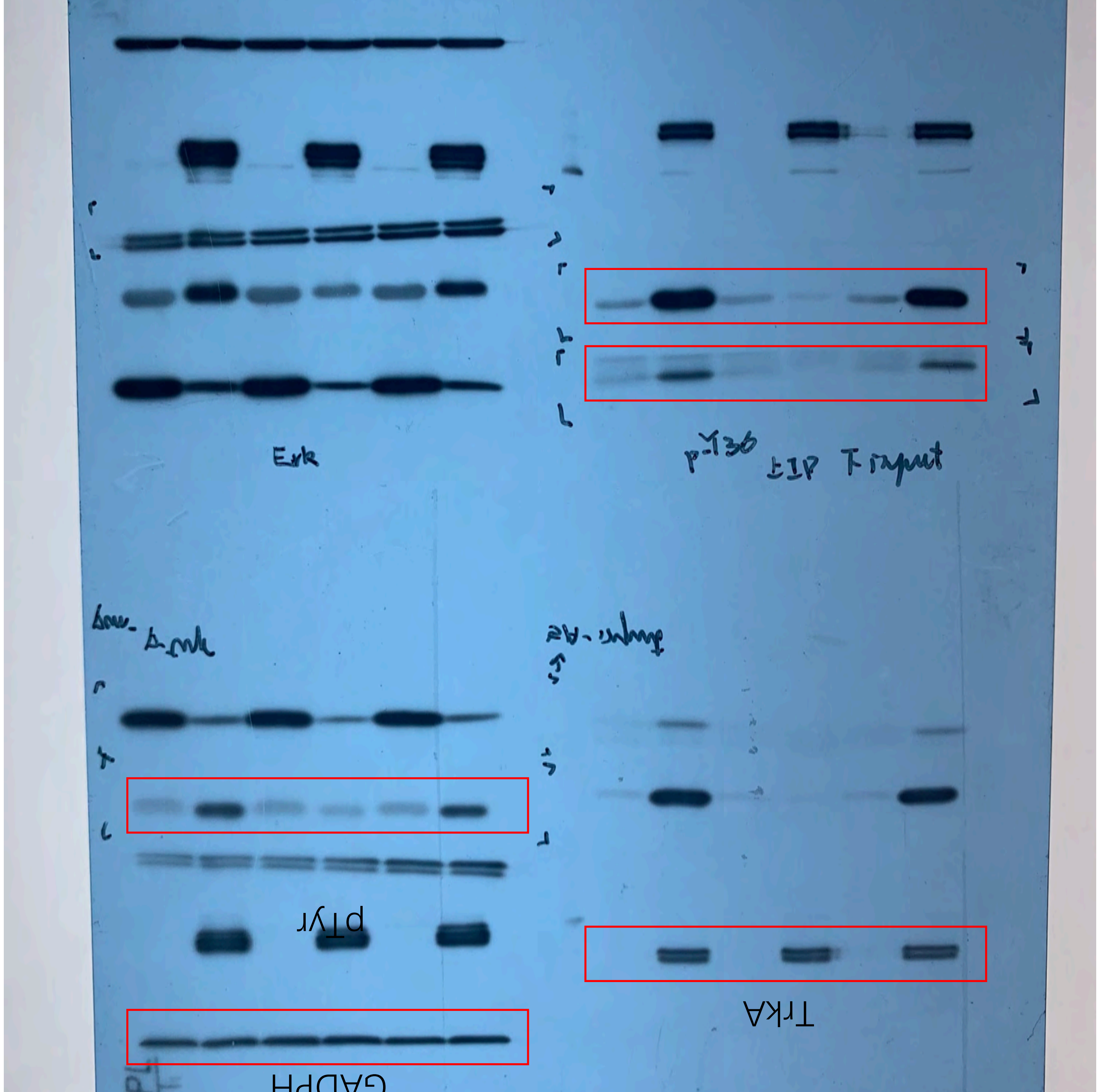

Fig. 1g

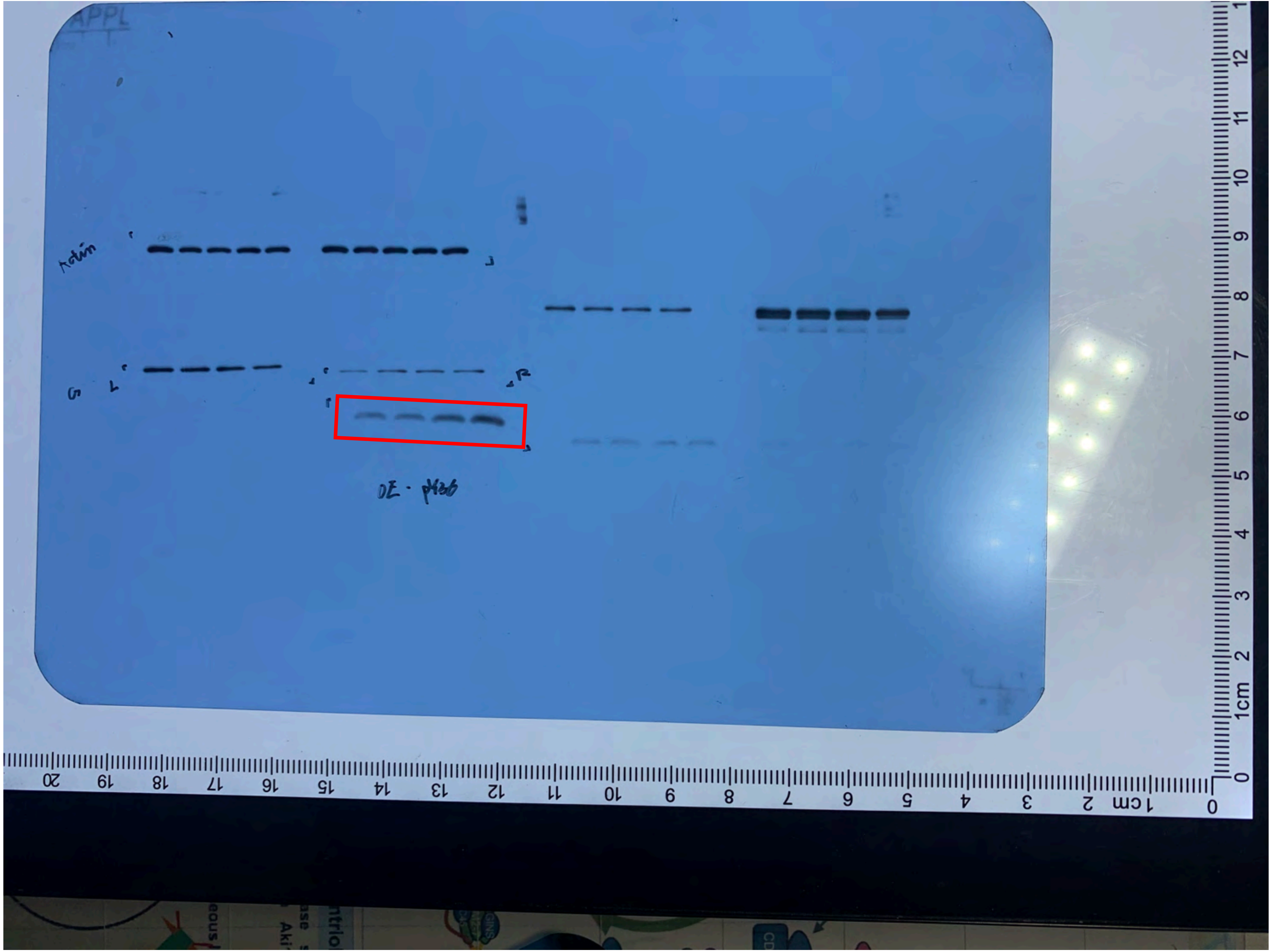

Fig. 1h

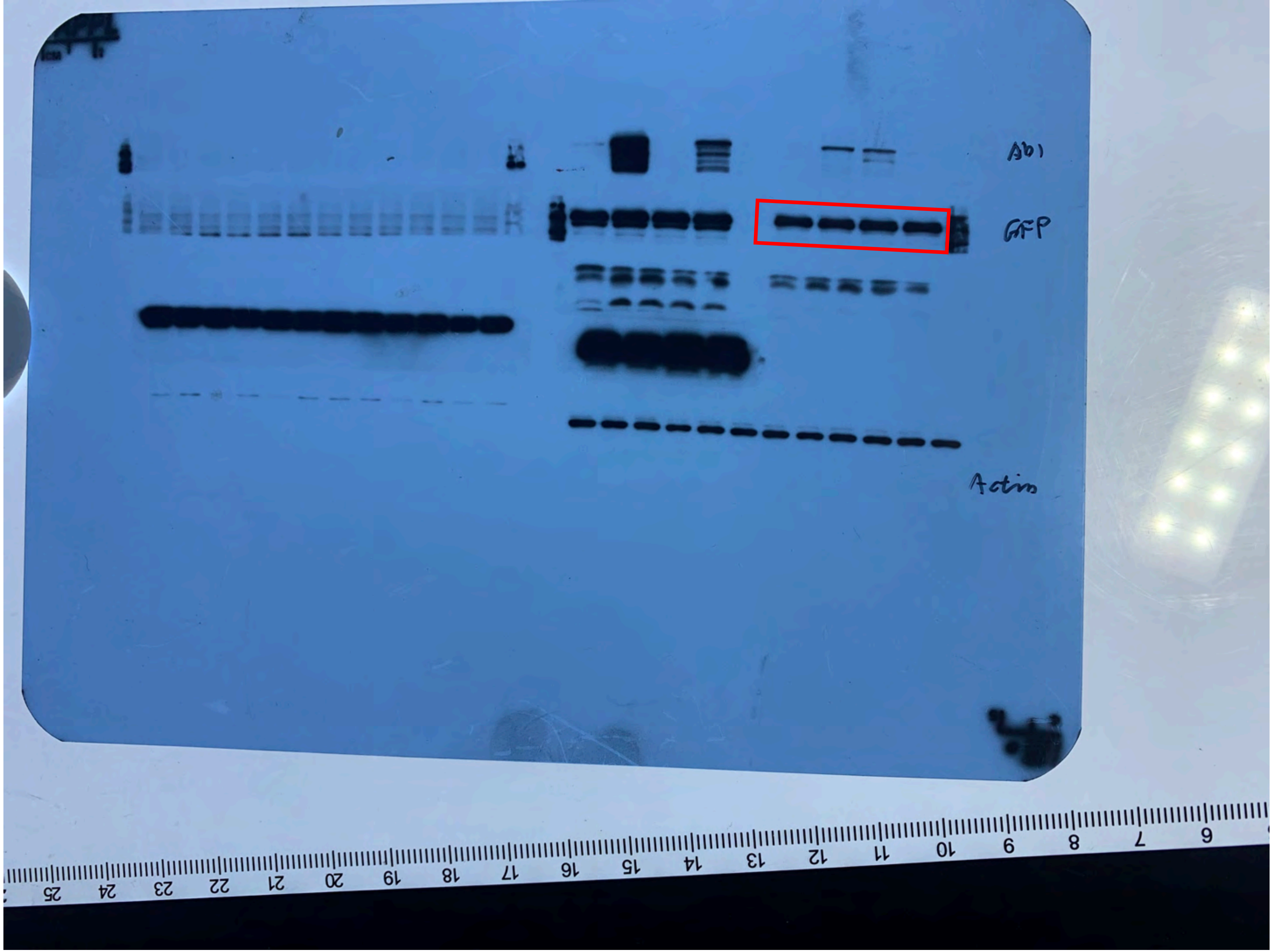

Fig. 1h

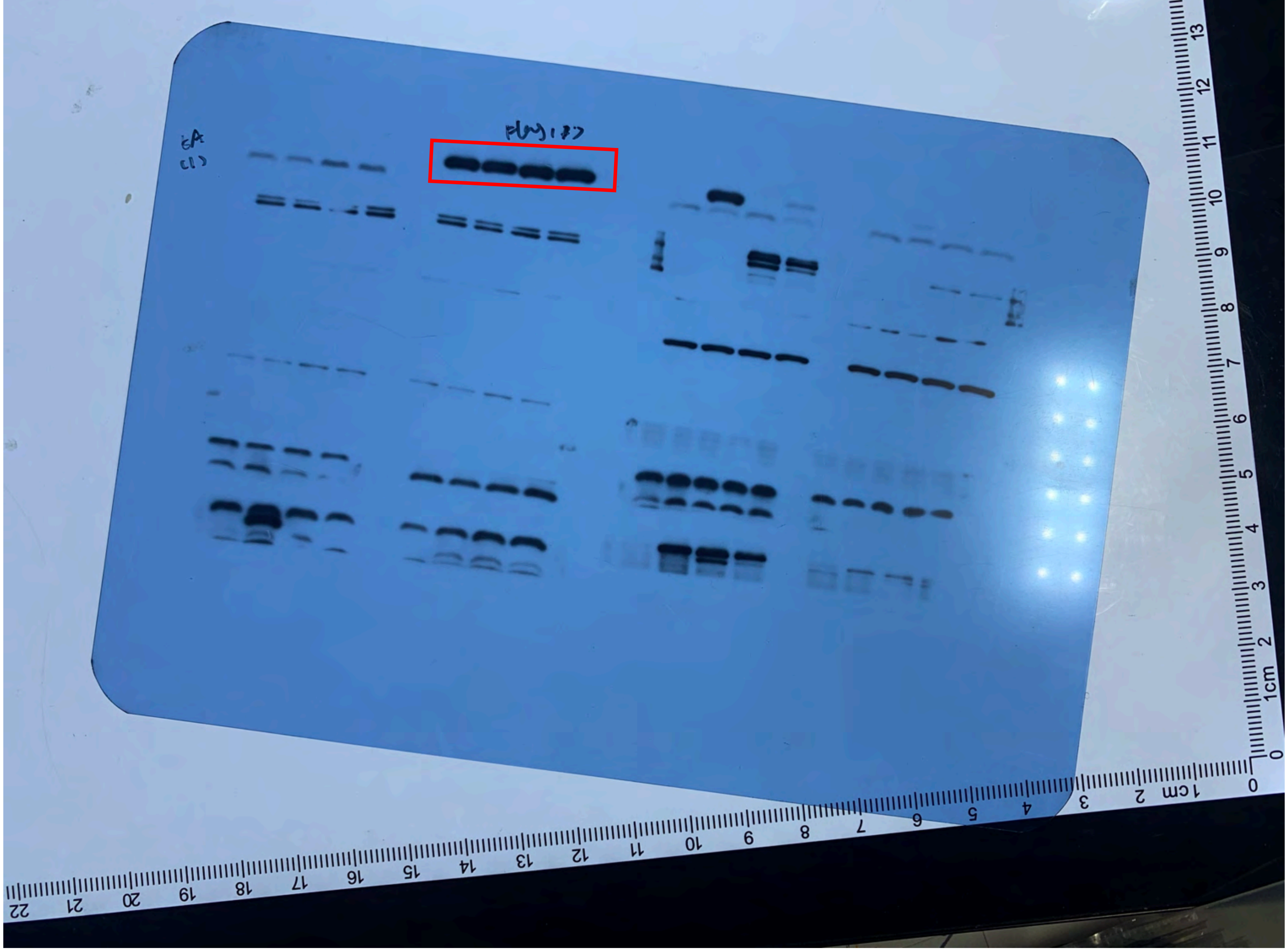

Fig. 1h

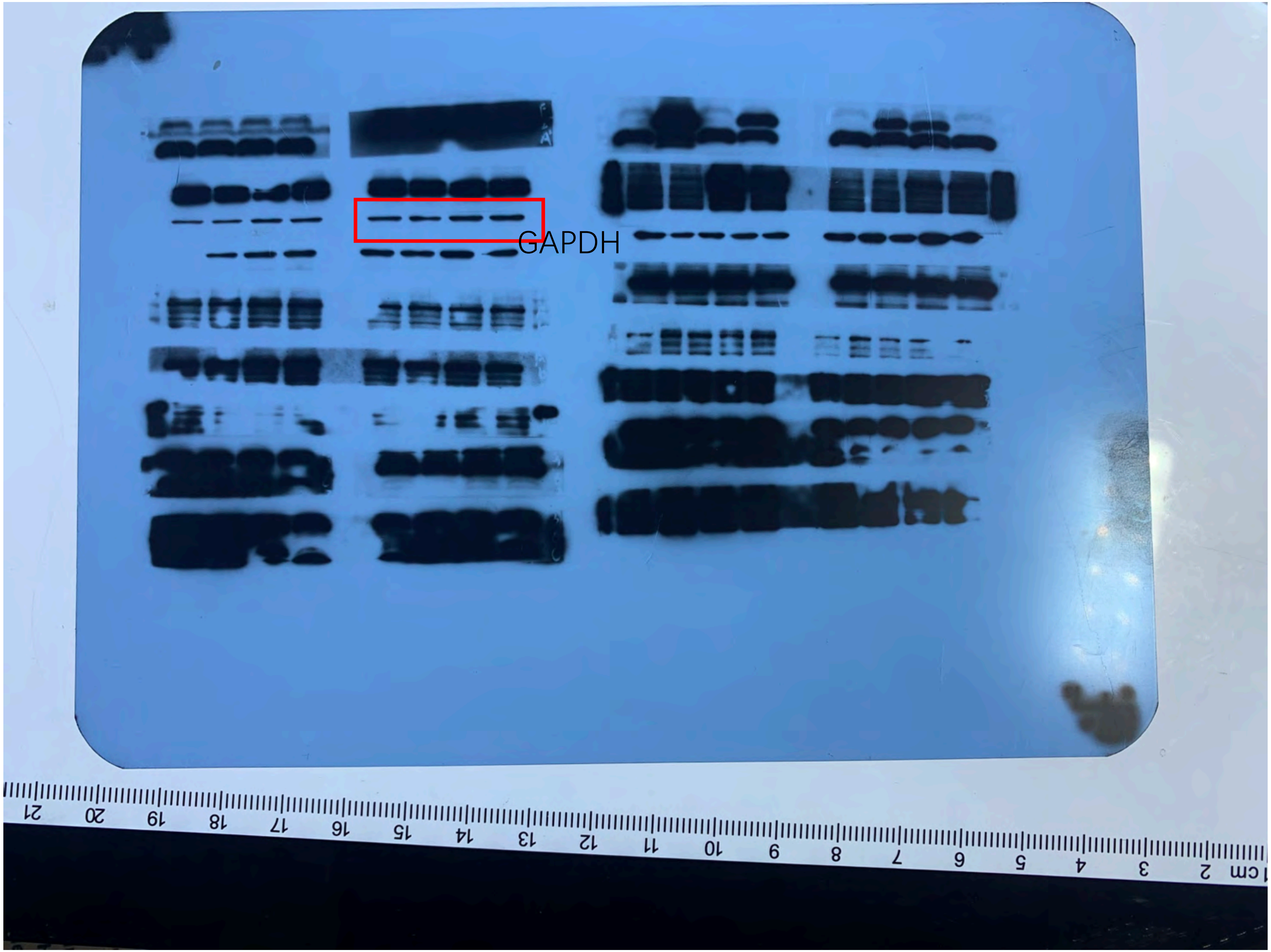

Fig. 1h

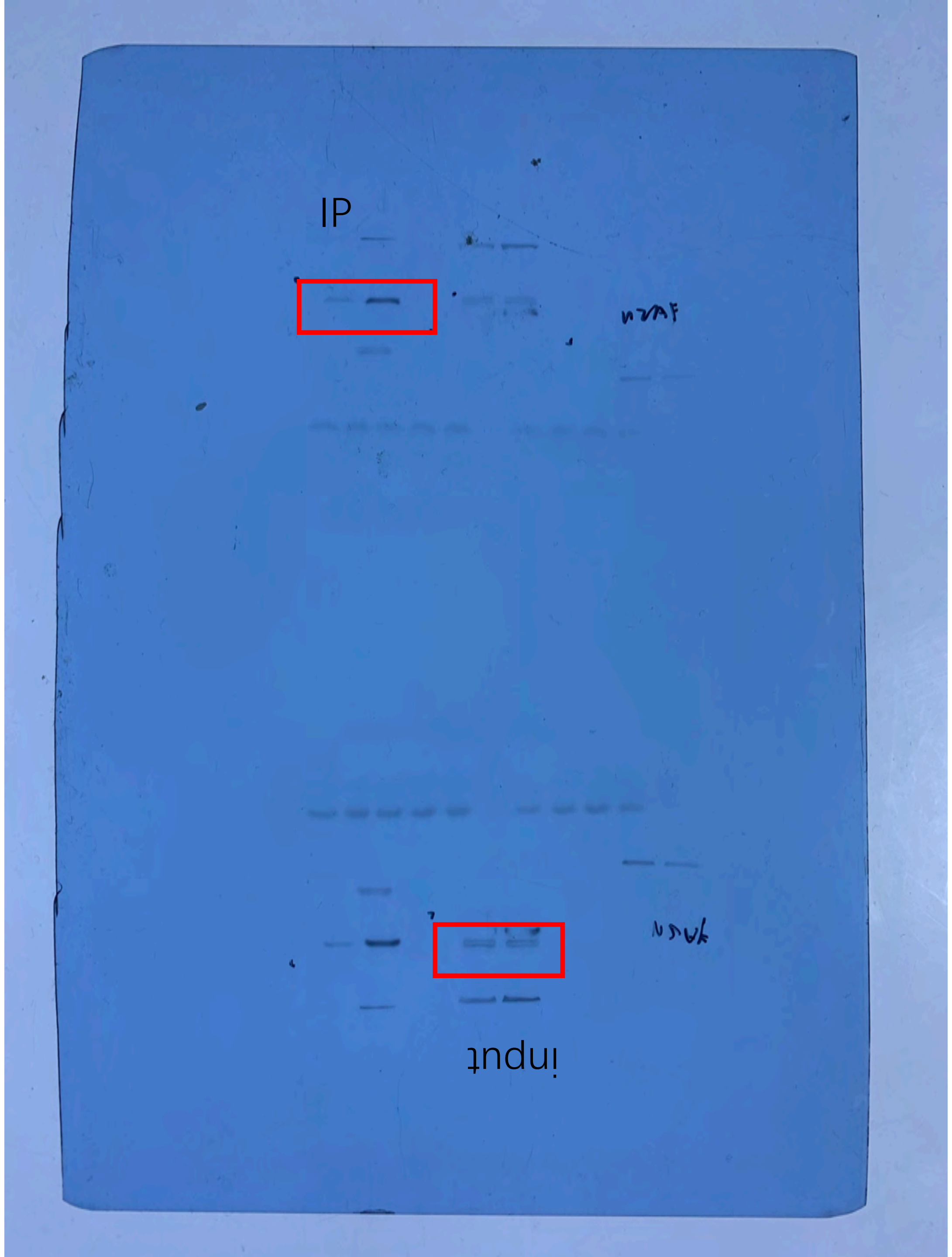

Fig. 1i

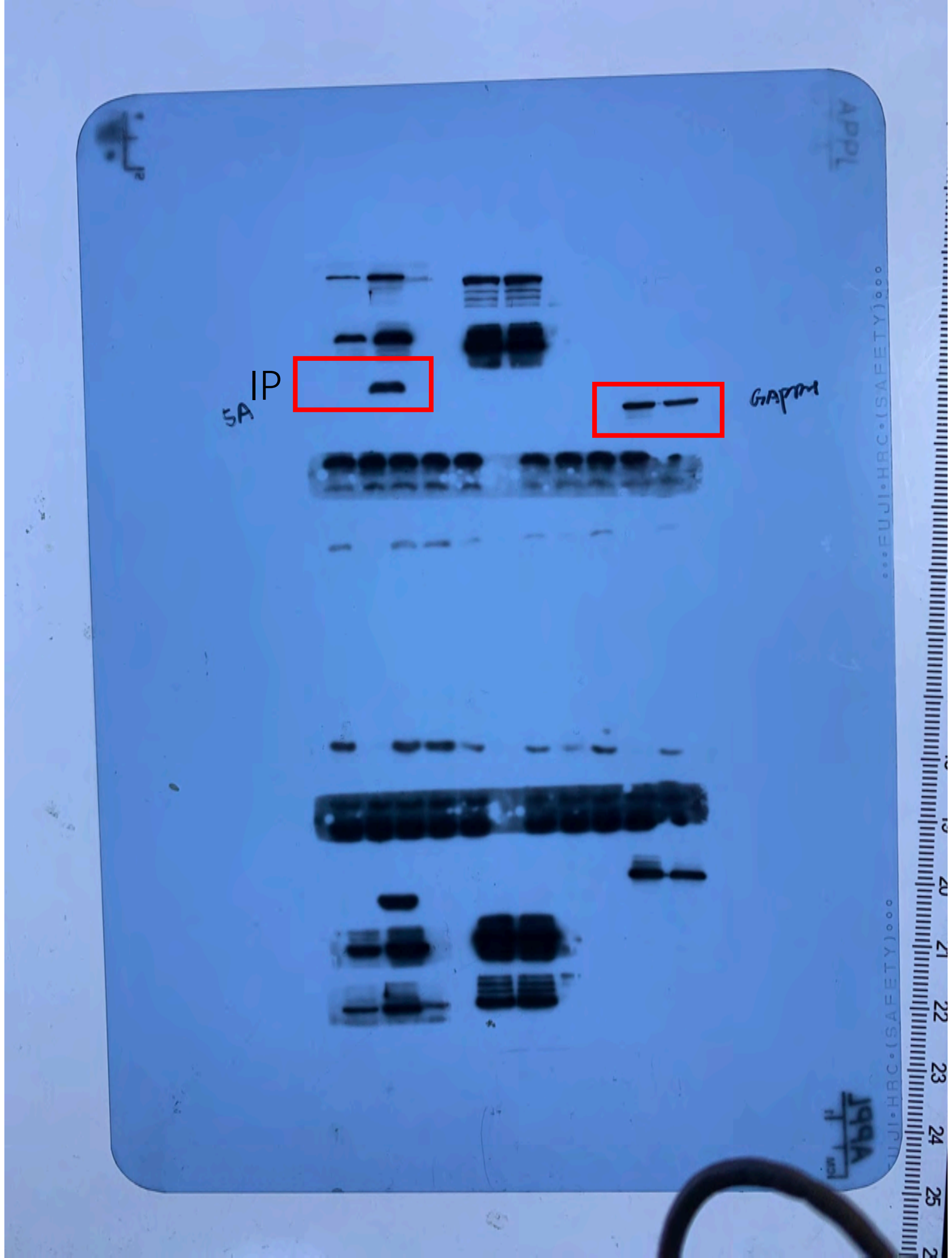

Fig. 1i

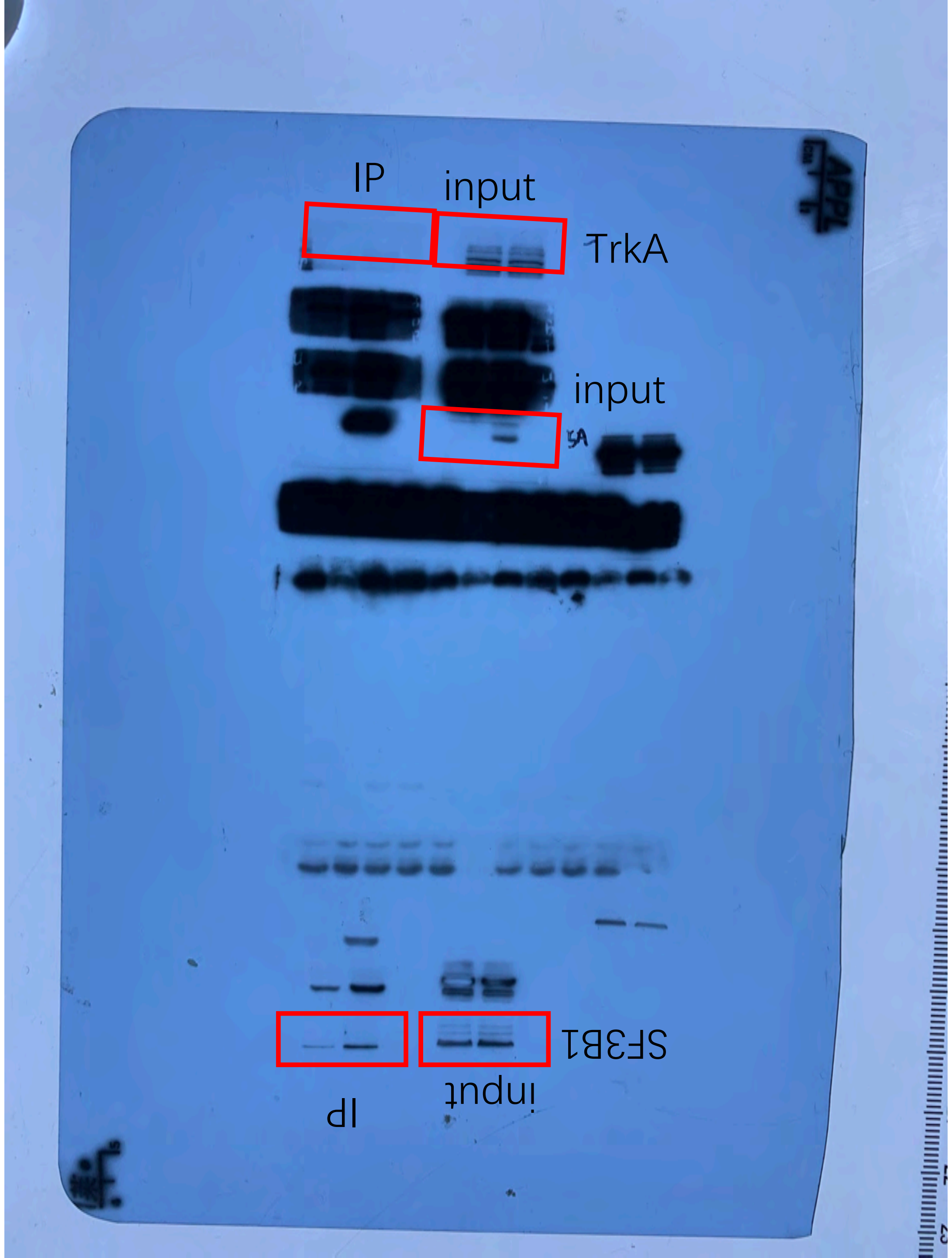

Fig. 1i

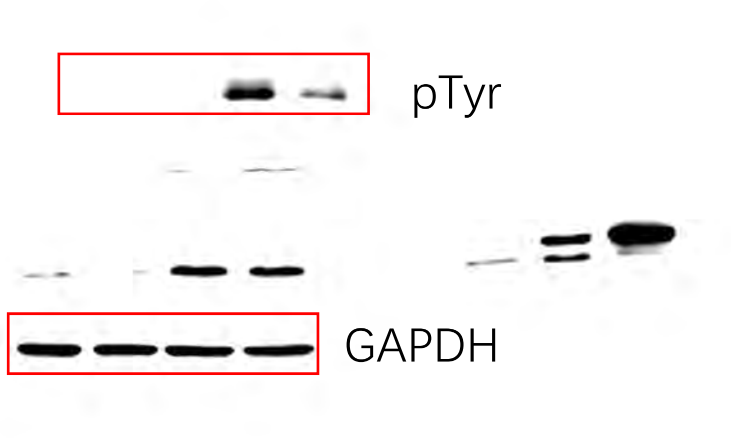

Fig. 2a

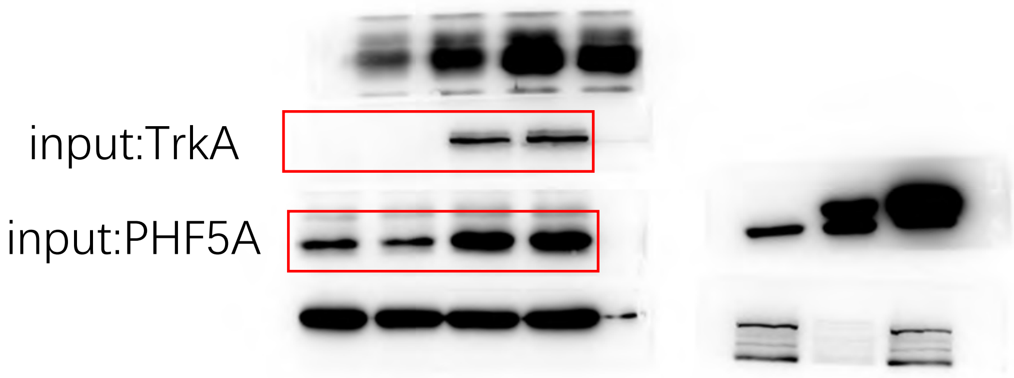

Fig. 2a

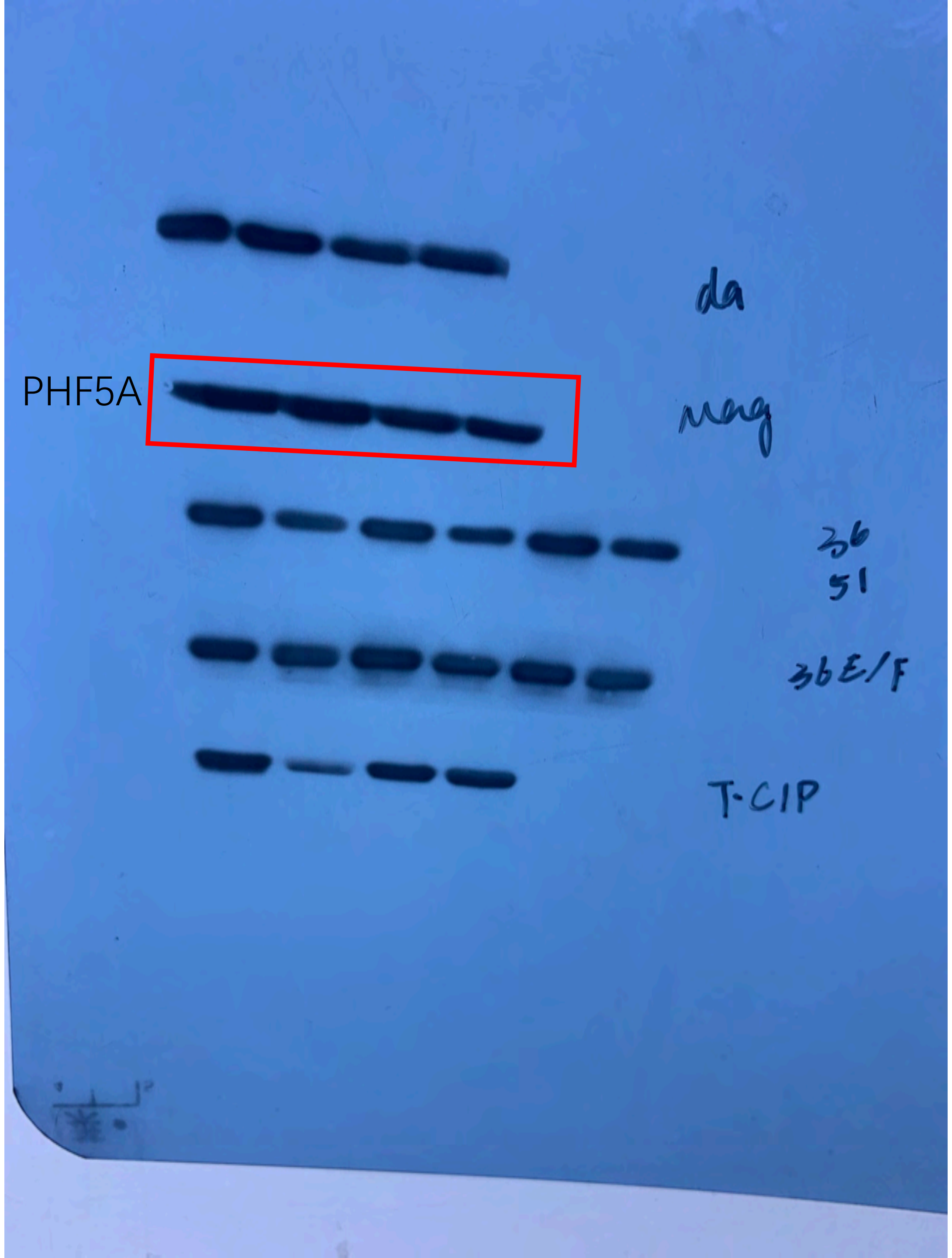

Fig. 2a

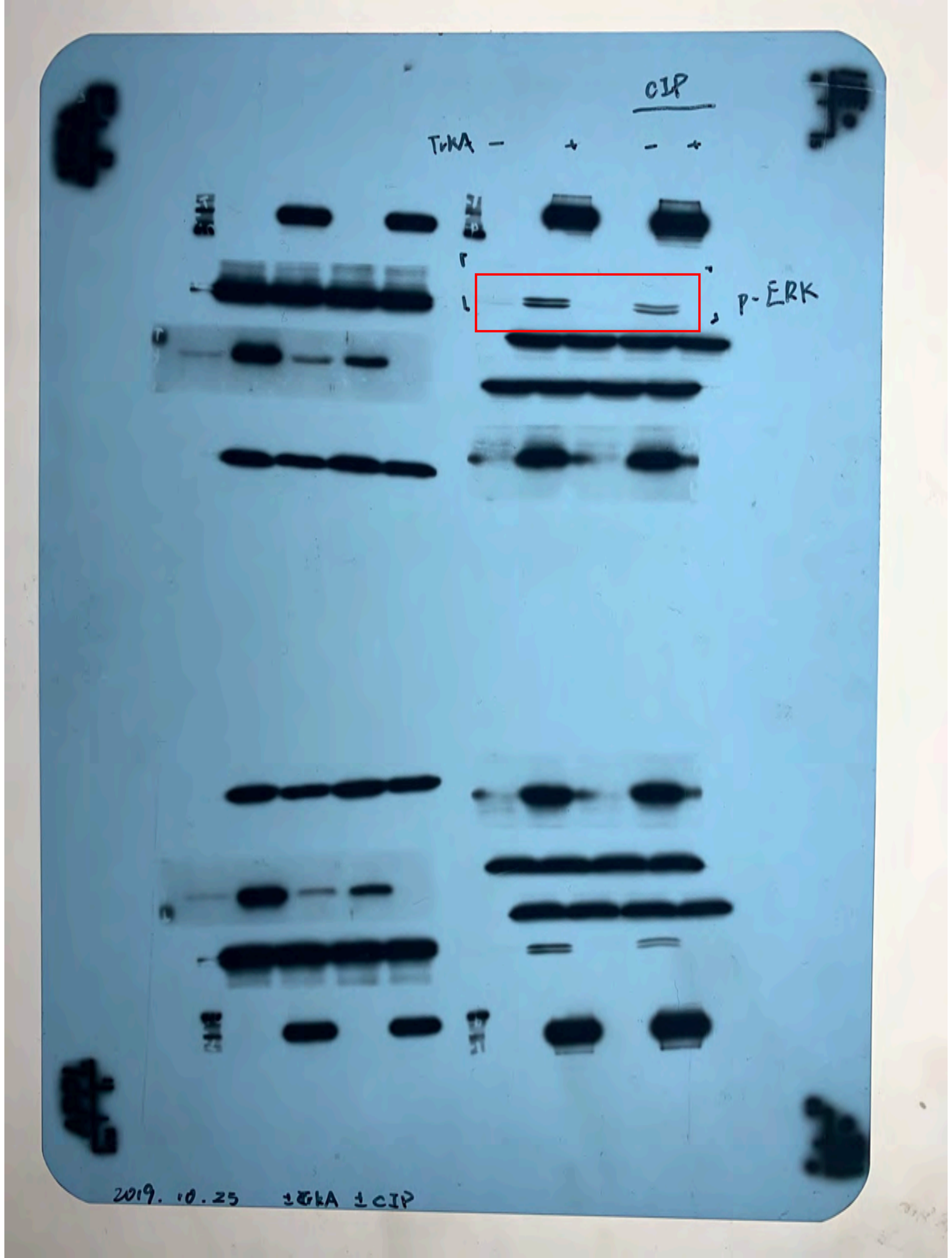

Fig. 2b

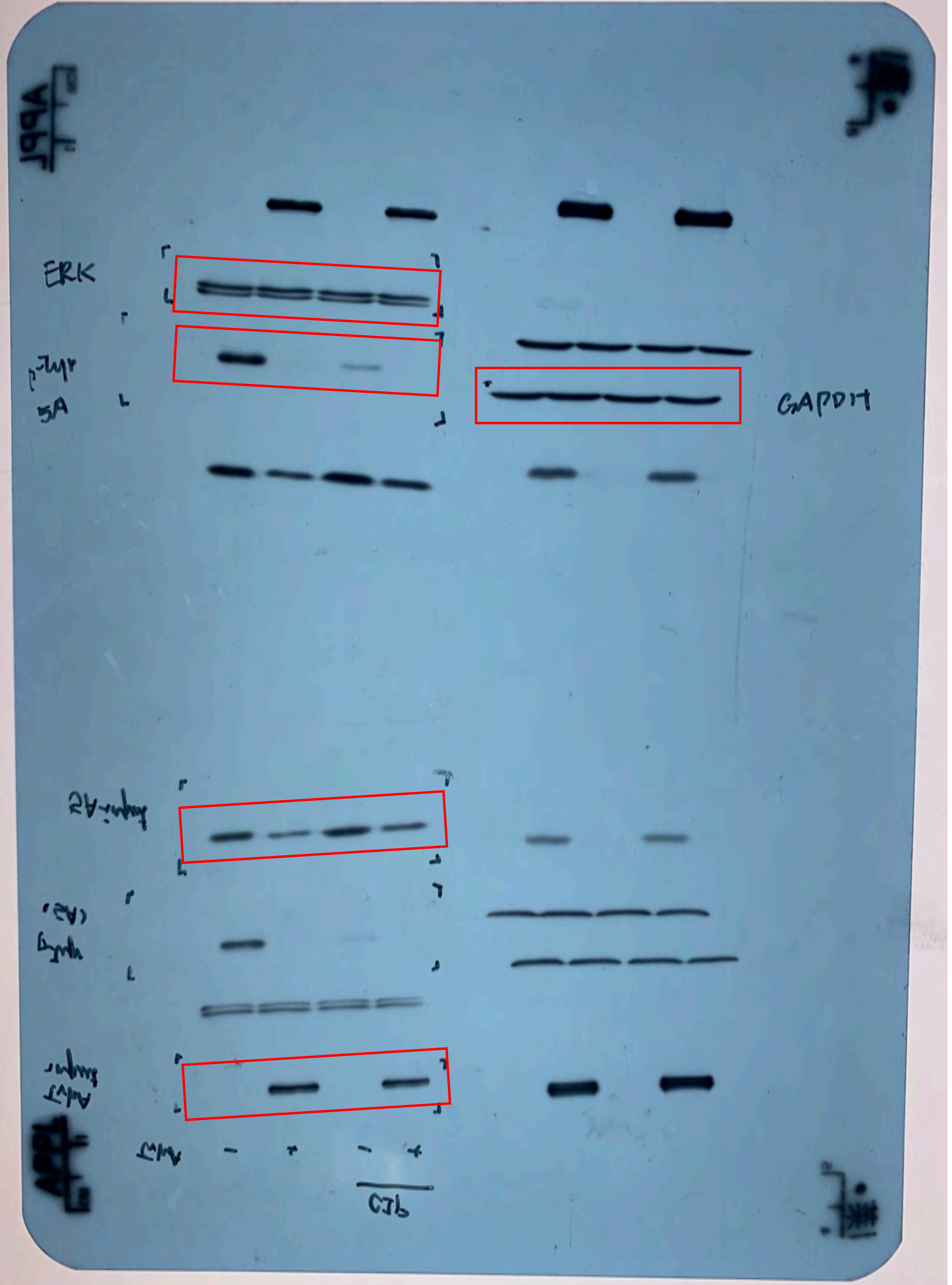

Fig. 2b

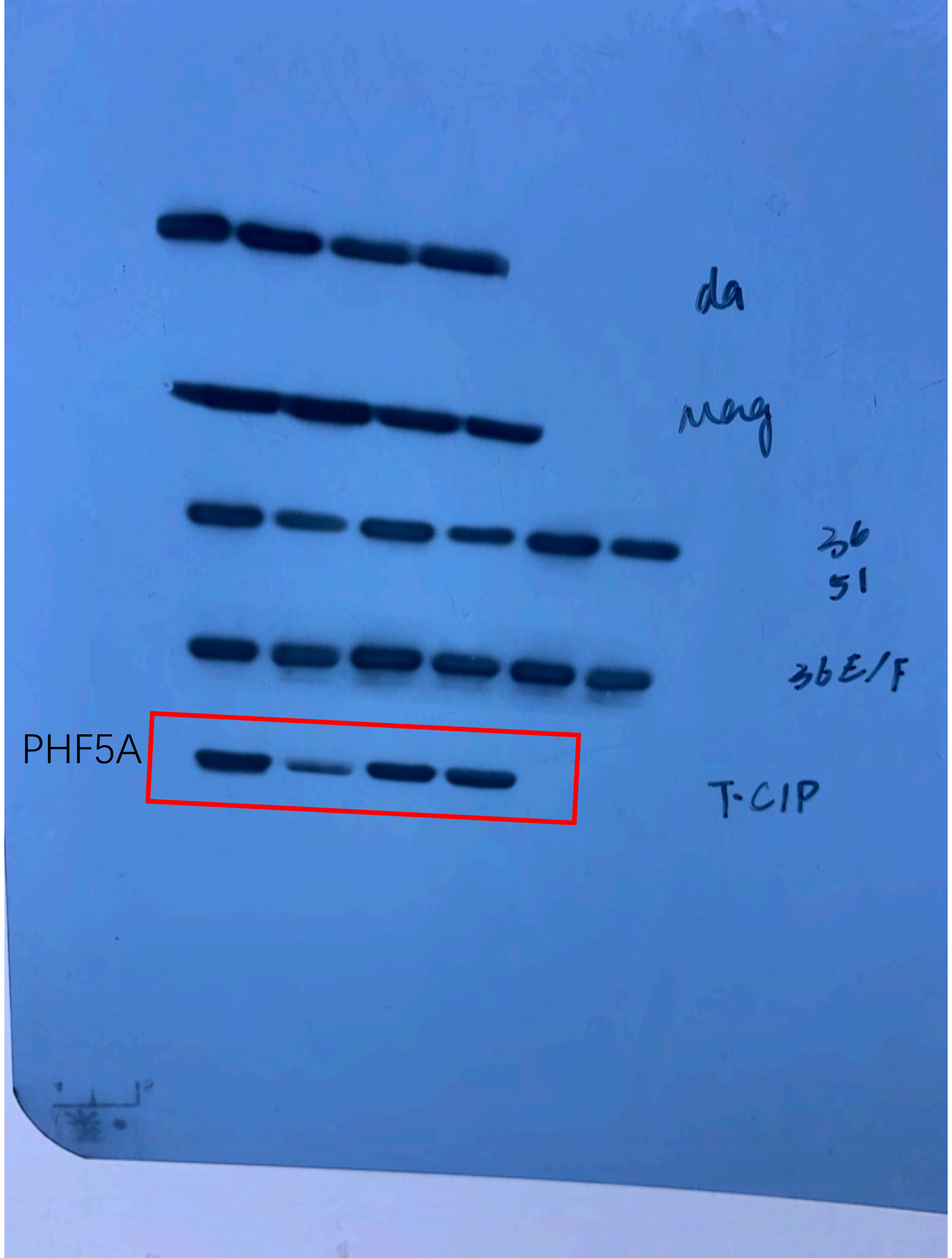

Fig. 2b

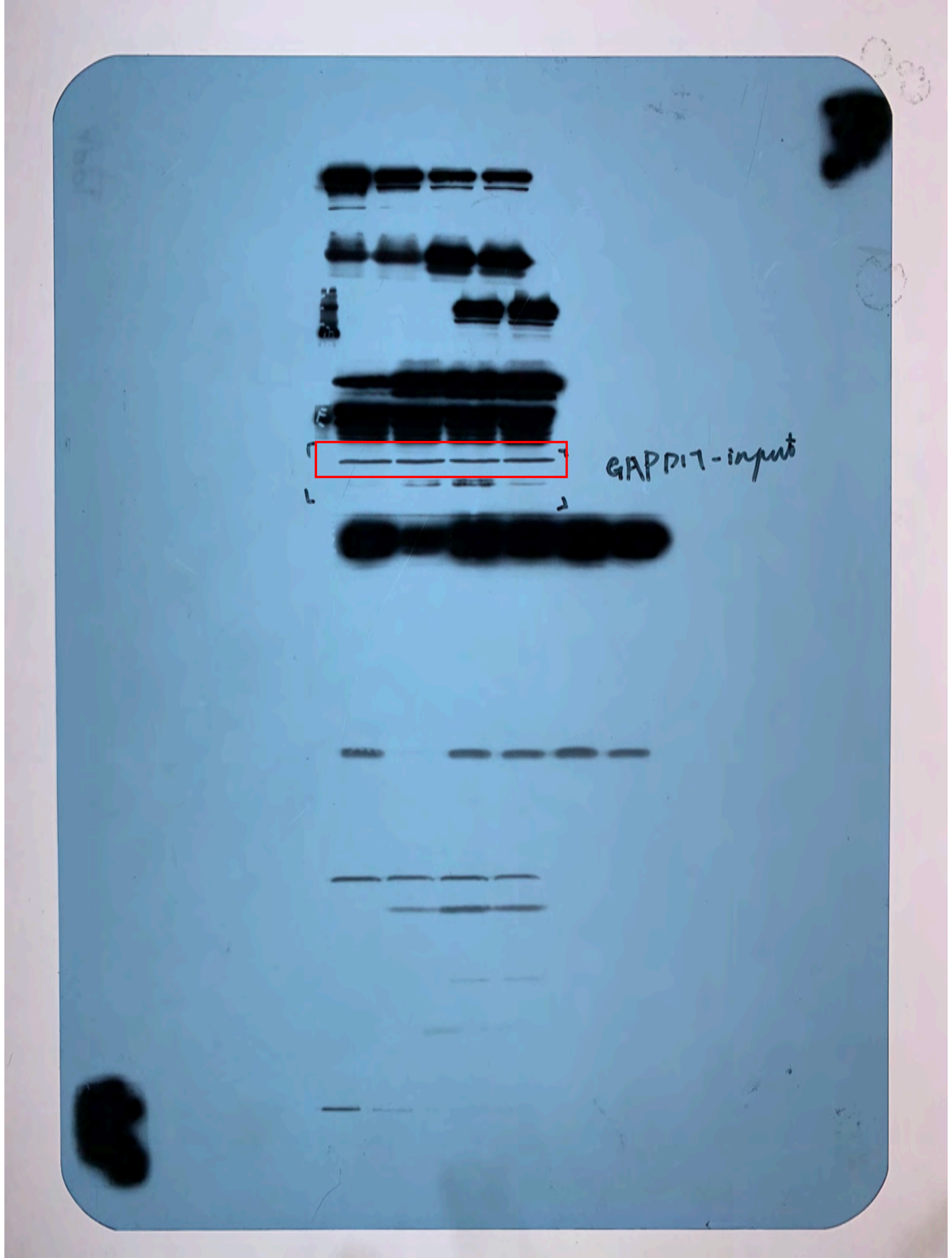

Fig. 2c

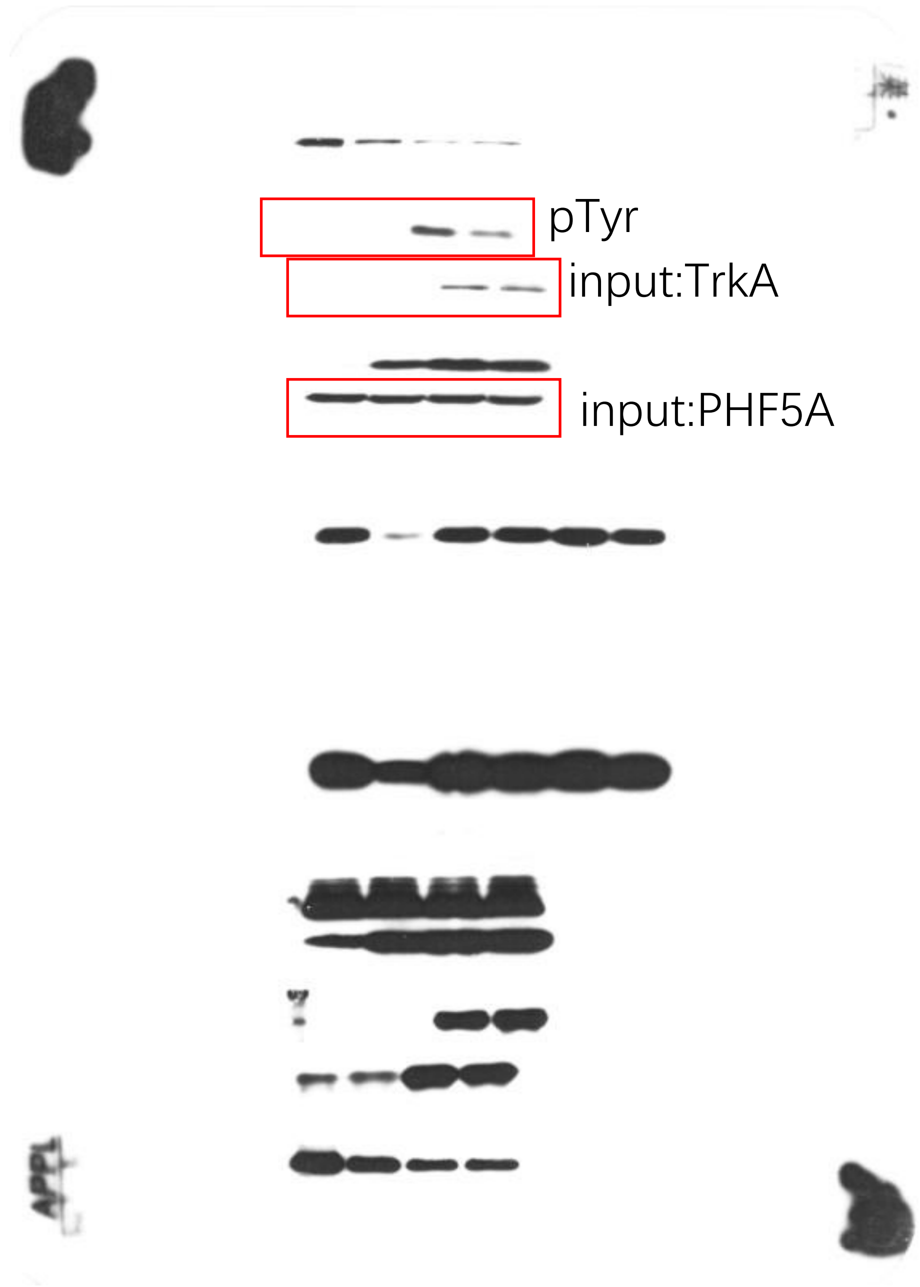

Fig. 2c

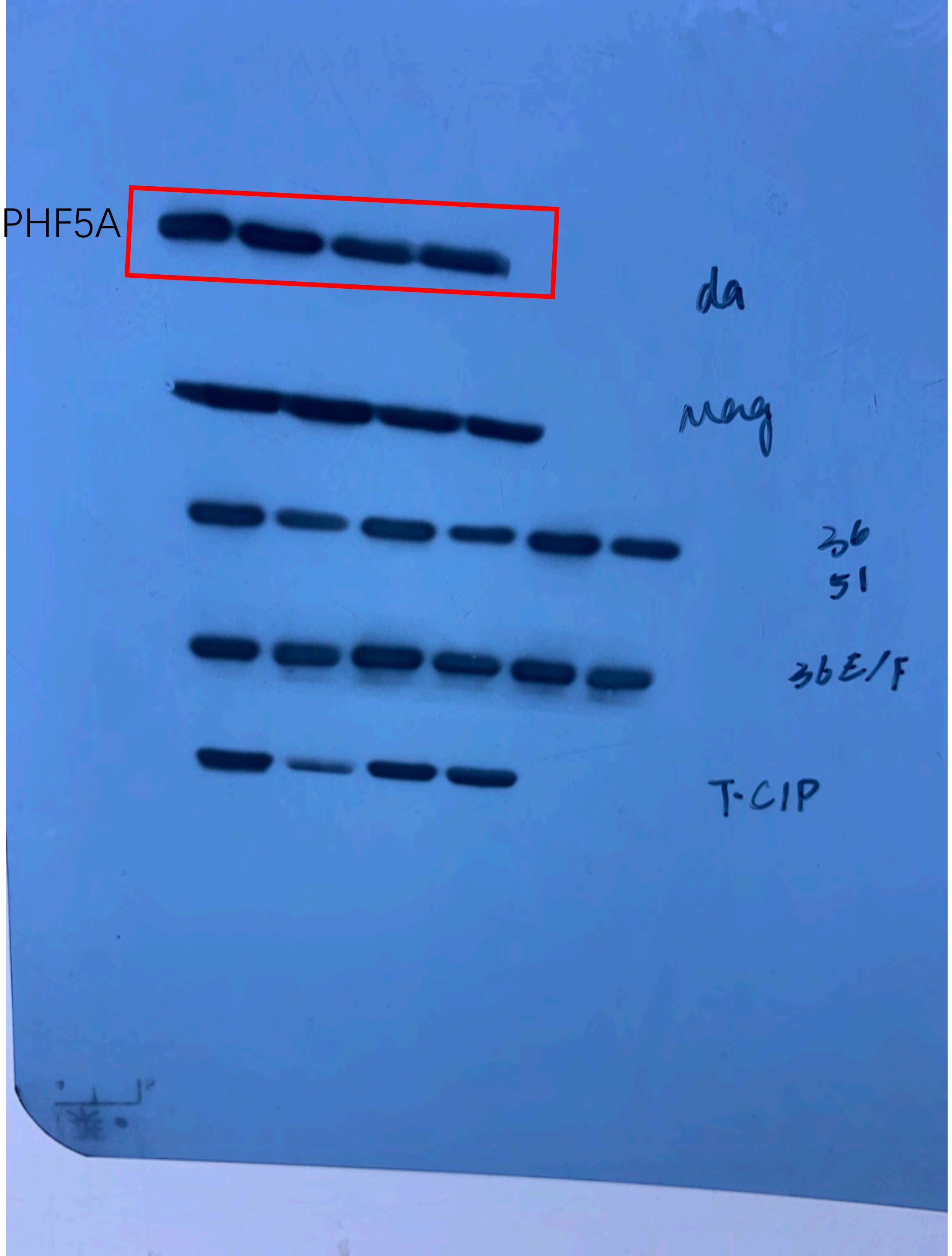

Fig. 2c

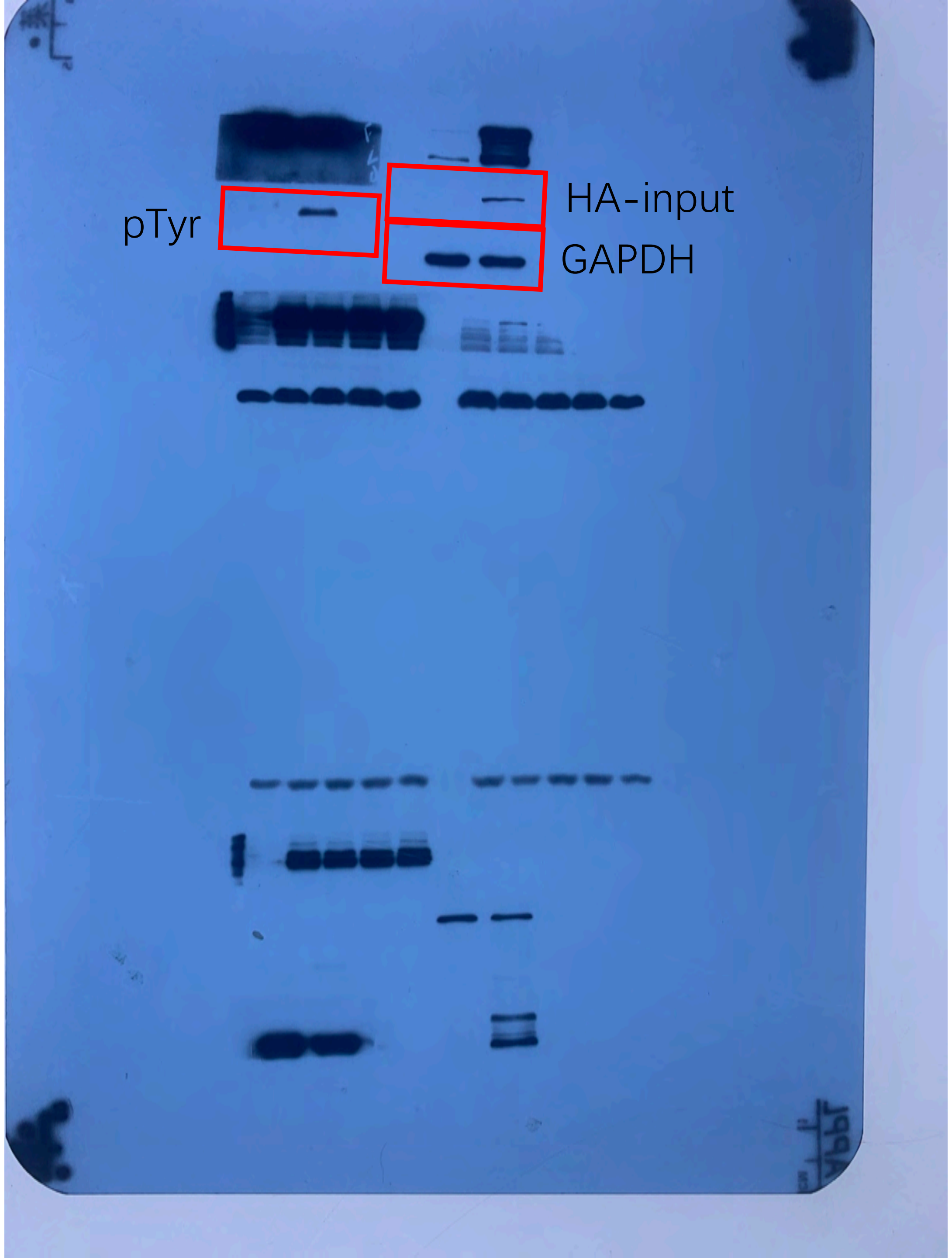

Fig. 2d

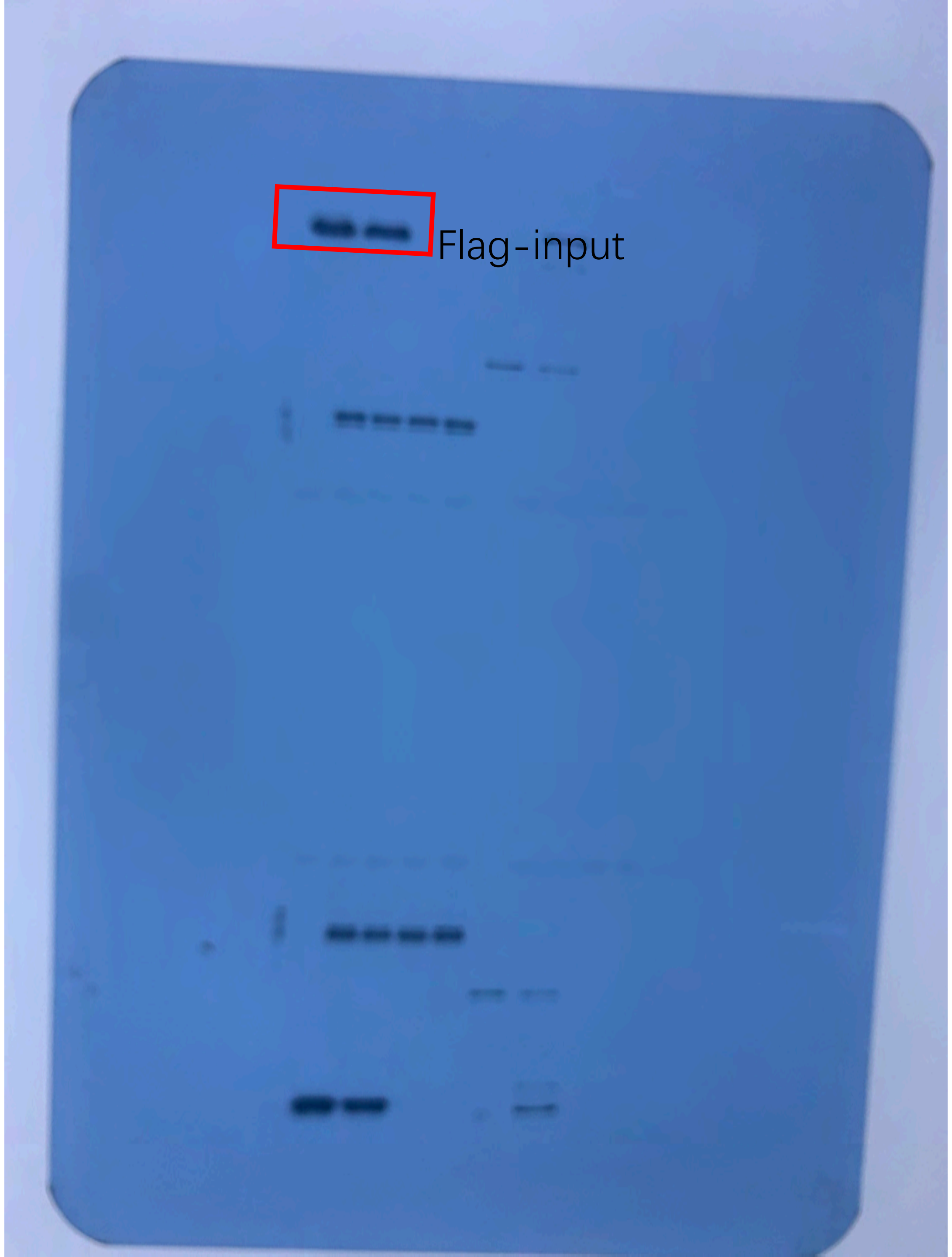

Fig. 2d

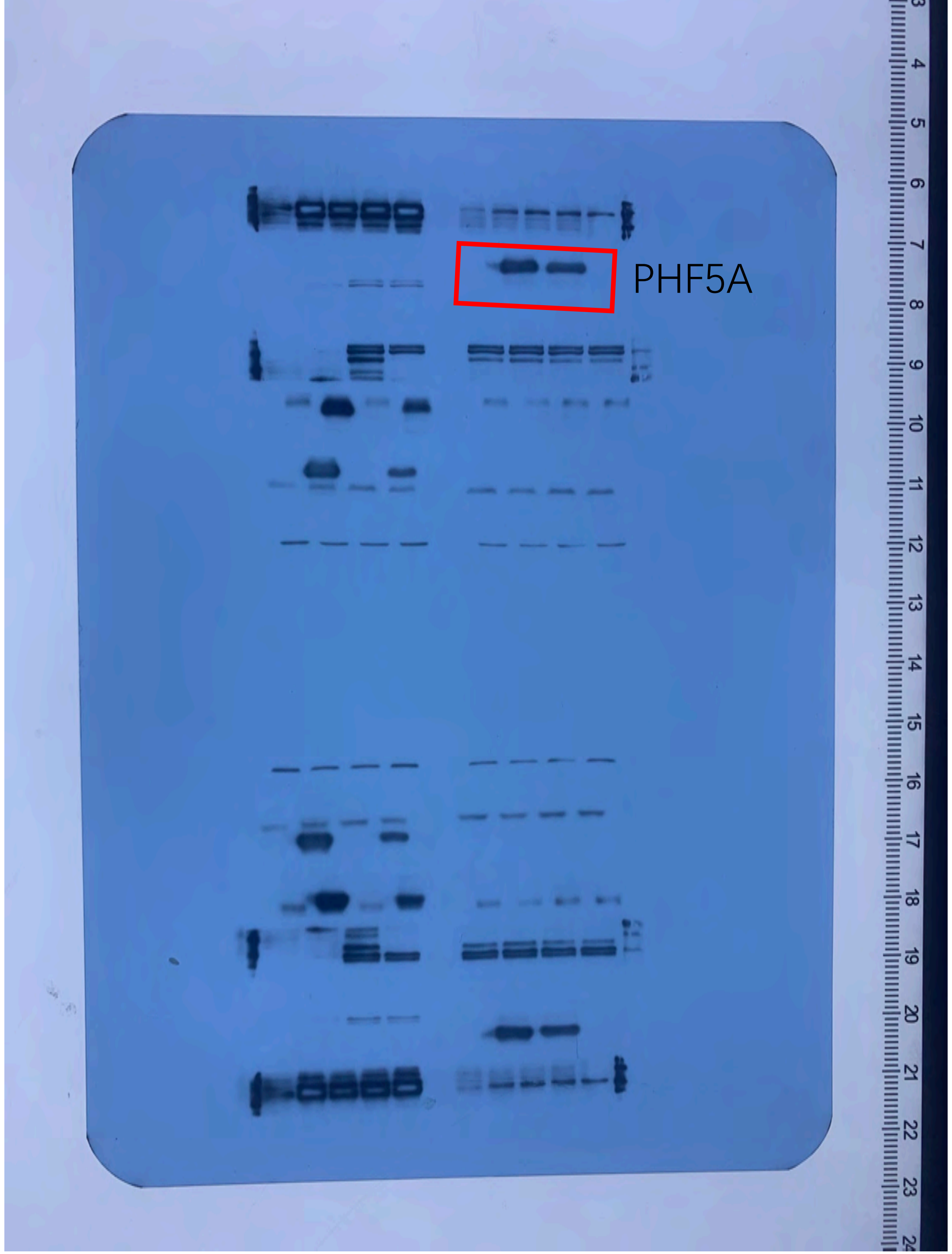

Fig. 2d

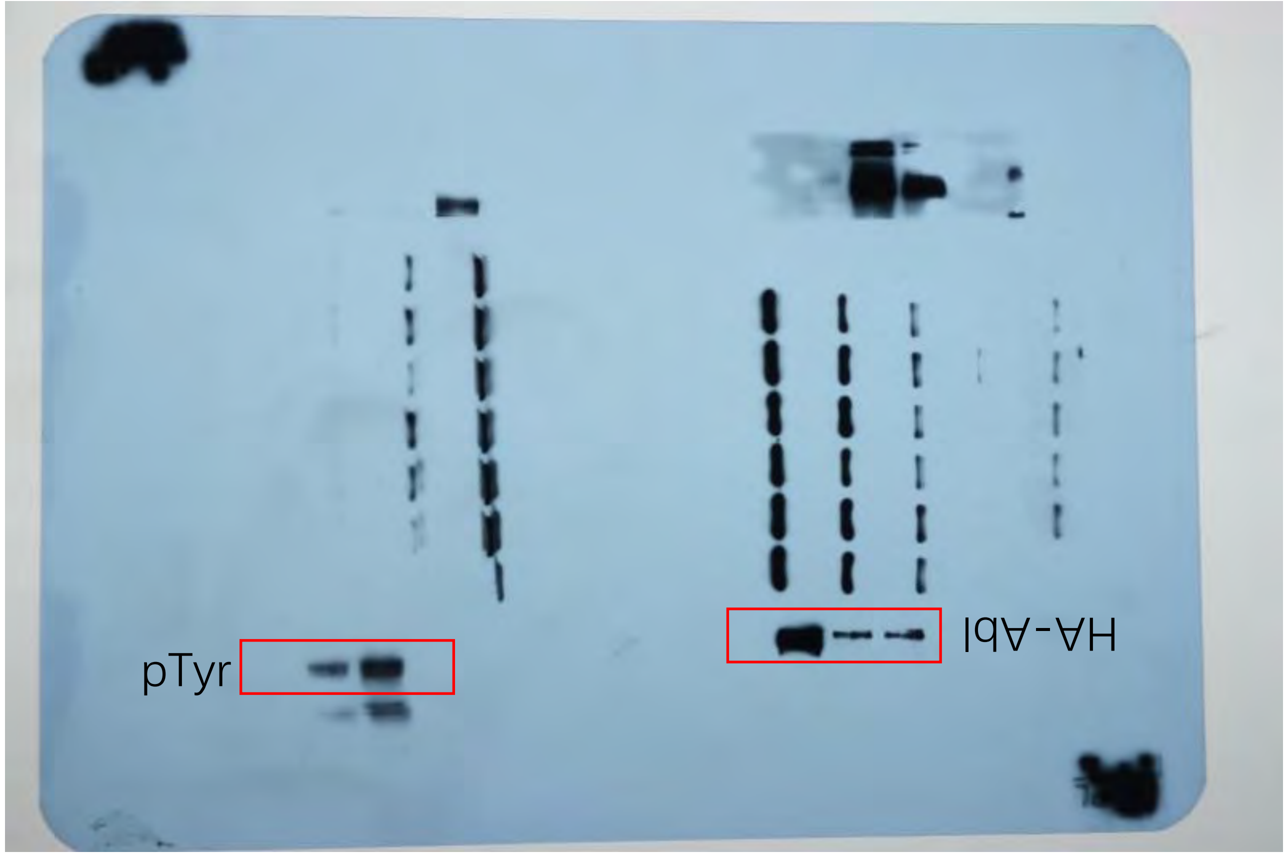

Fig. 2e

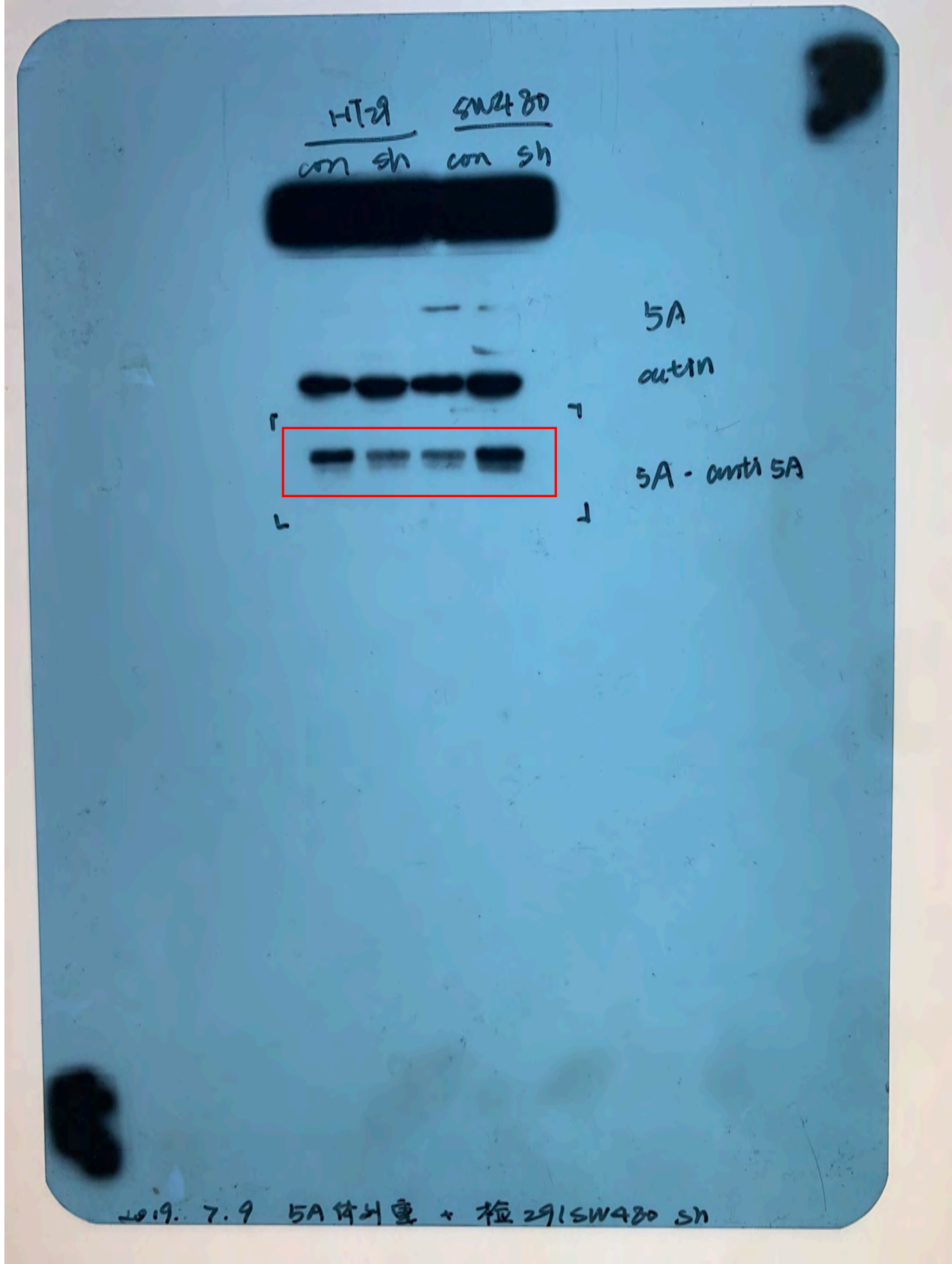

Fig. 2e

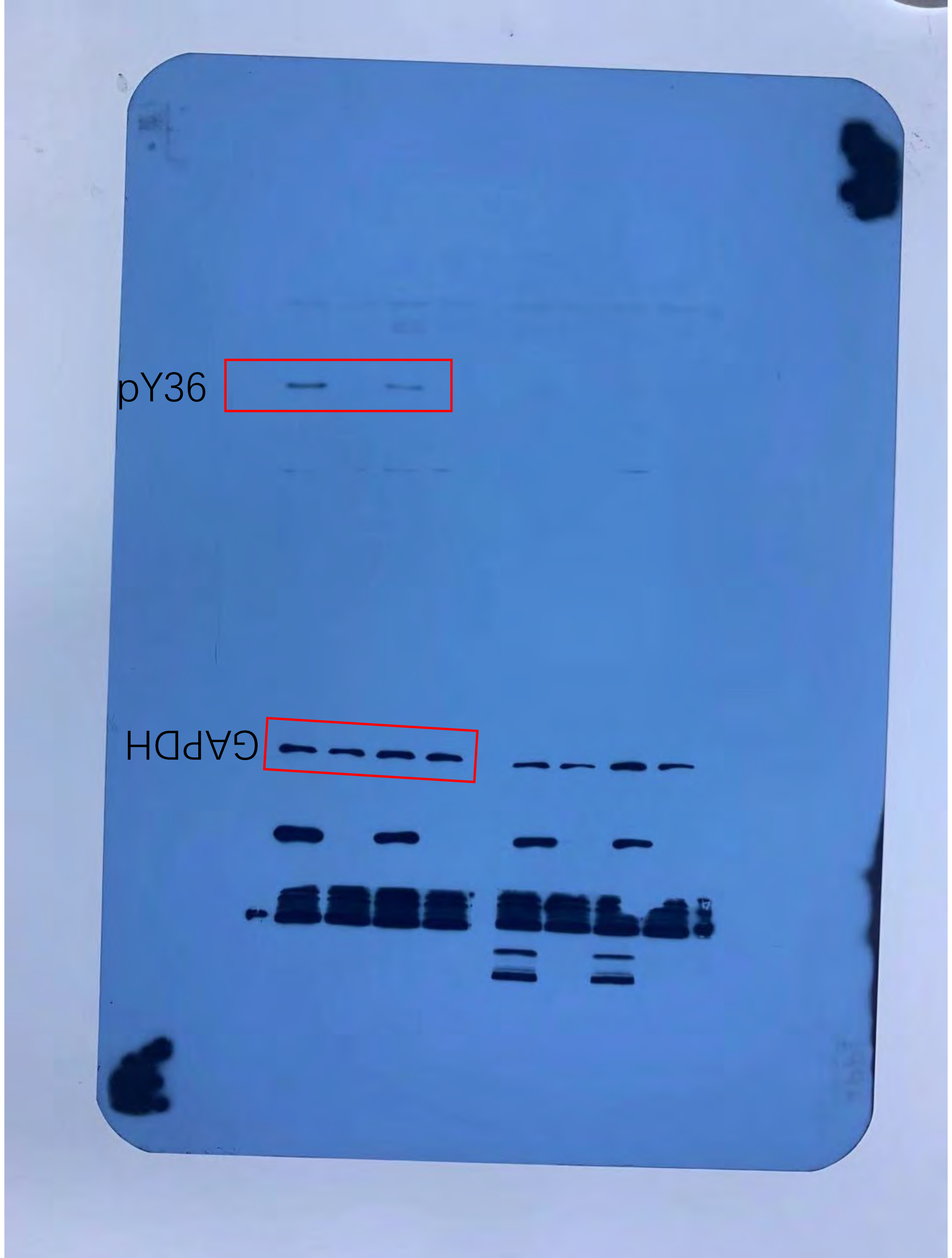

Fig. 2g

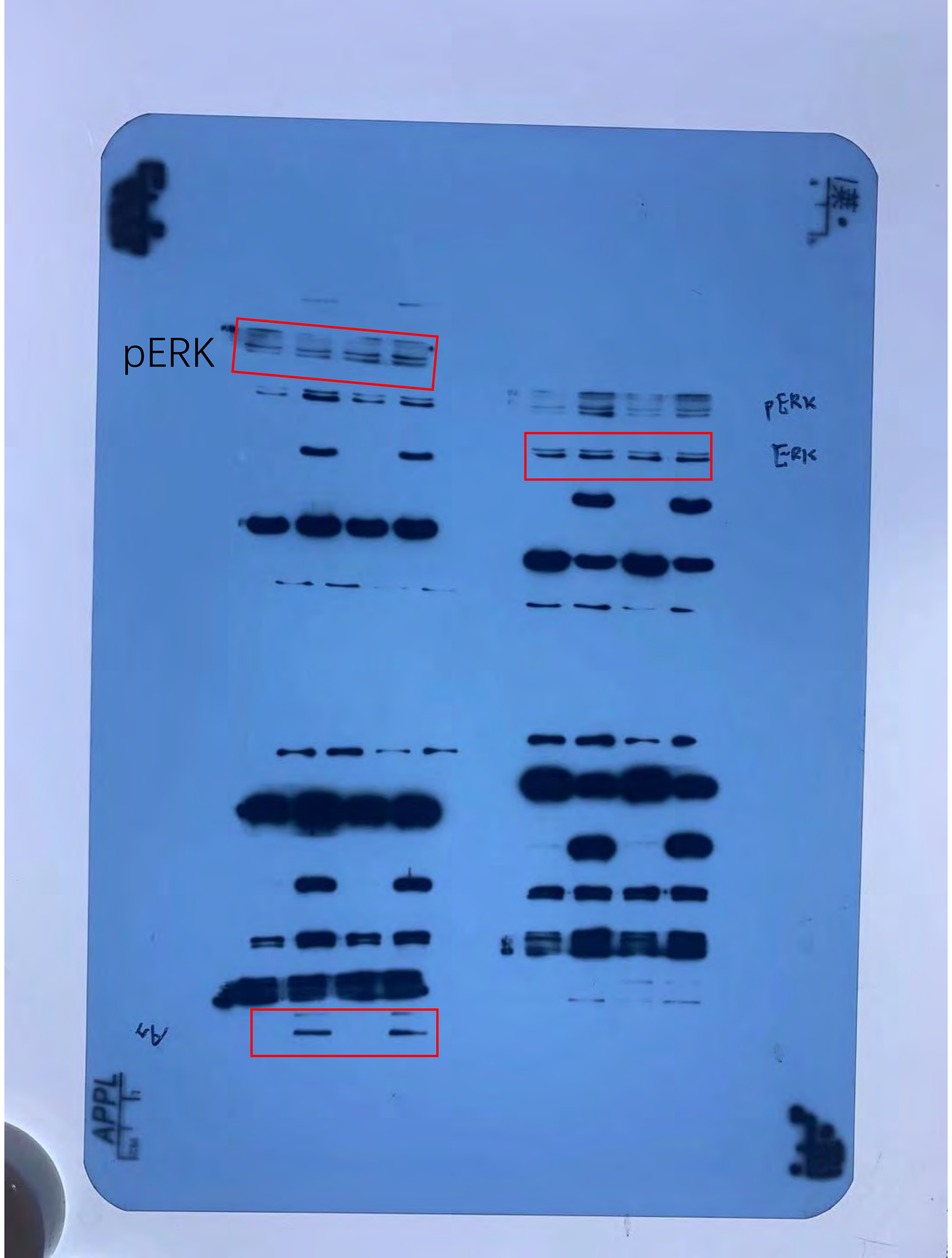

Fig. 2g

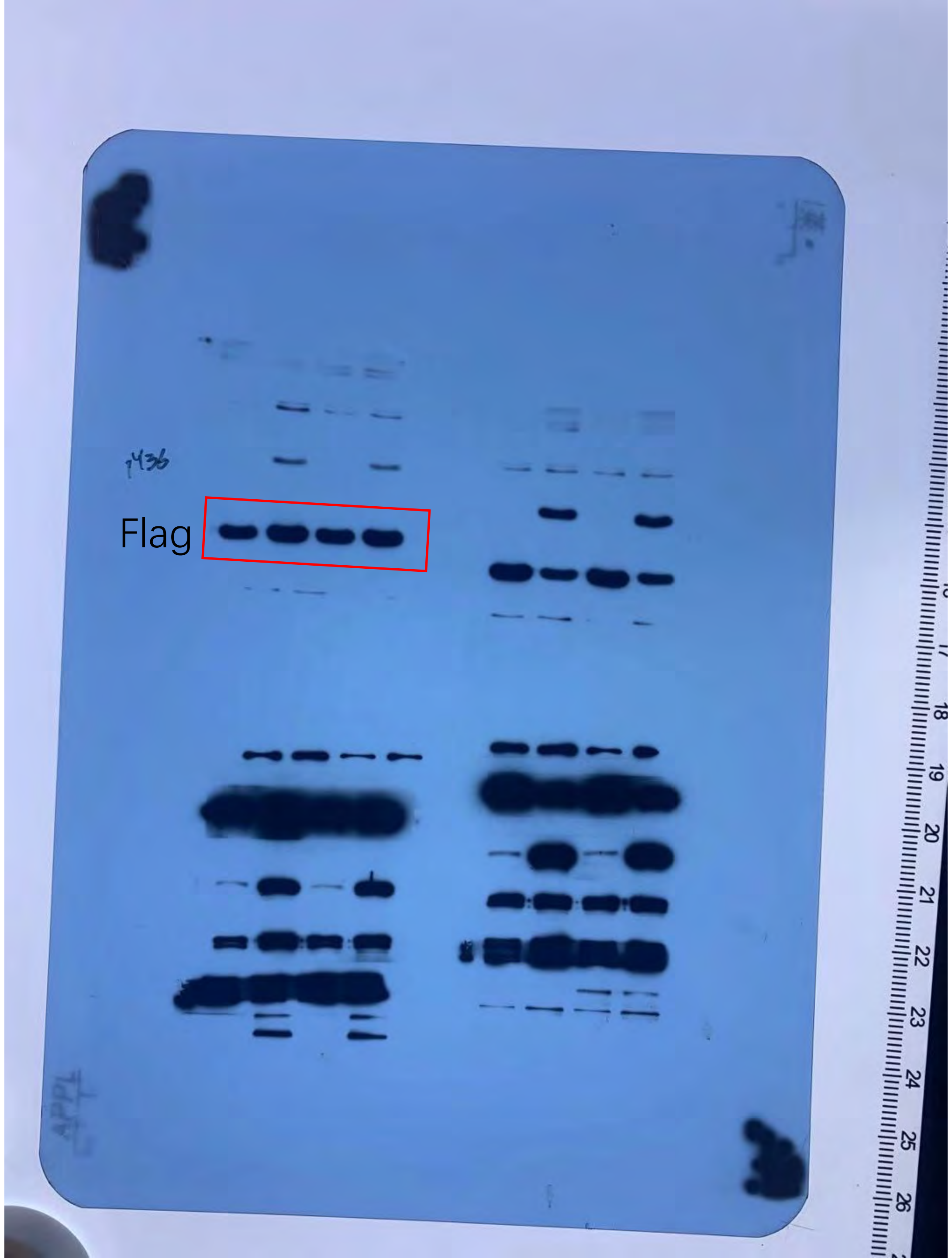

Fig. 2g

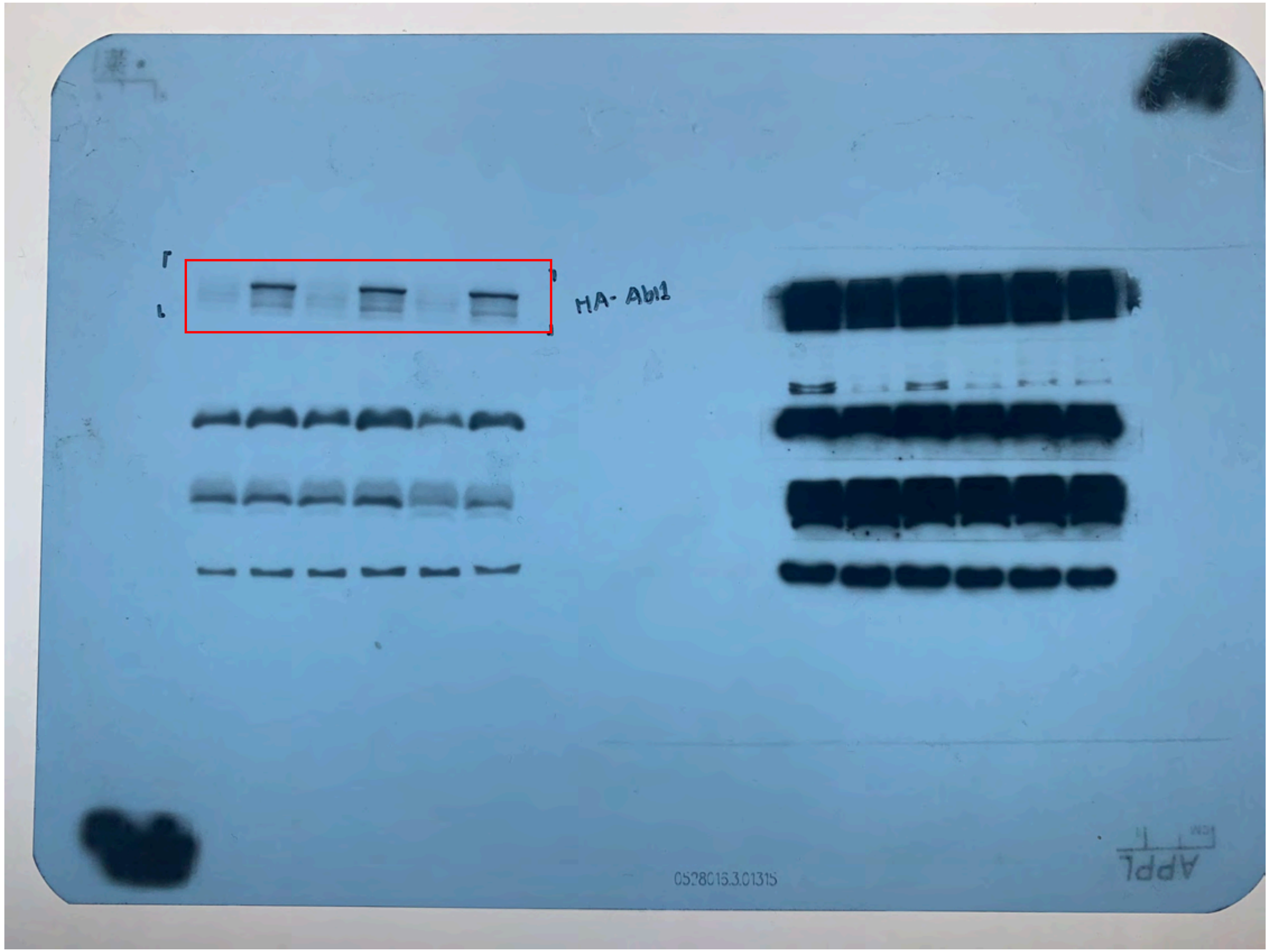

Fig. 2f

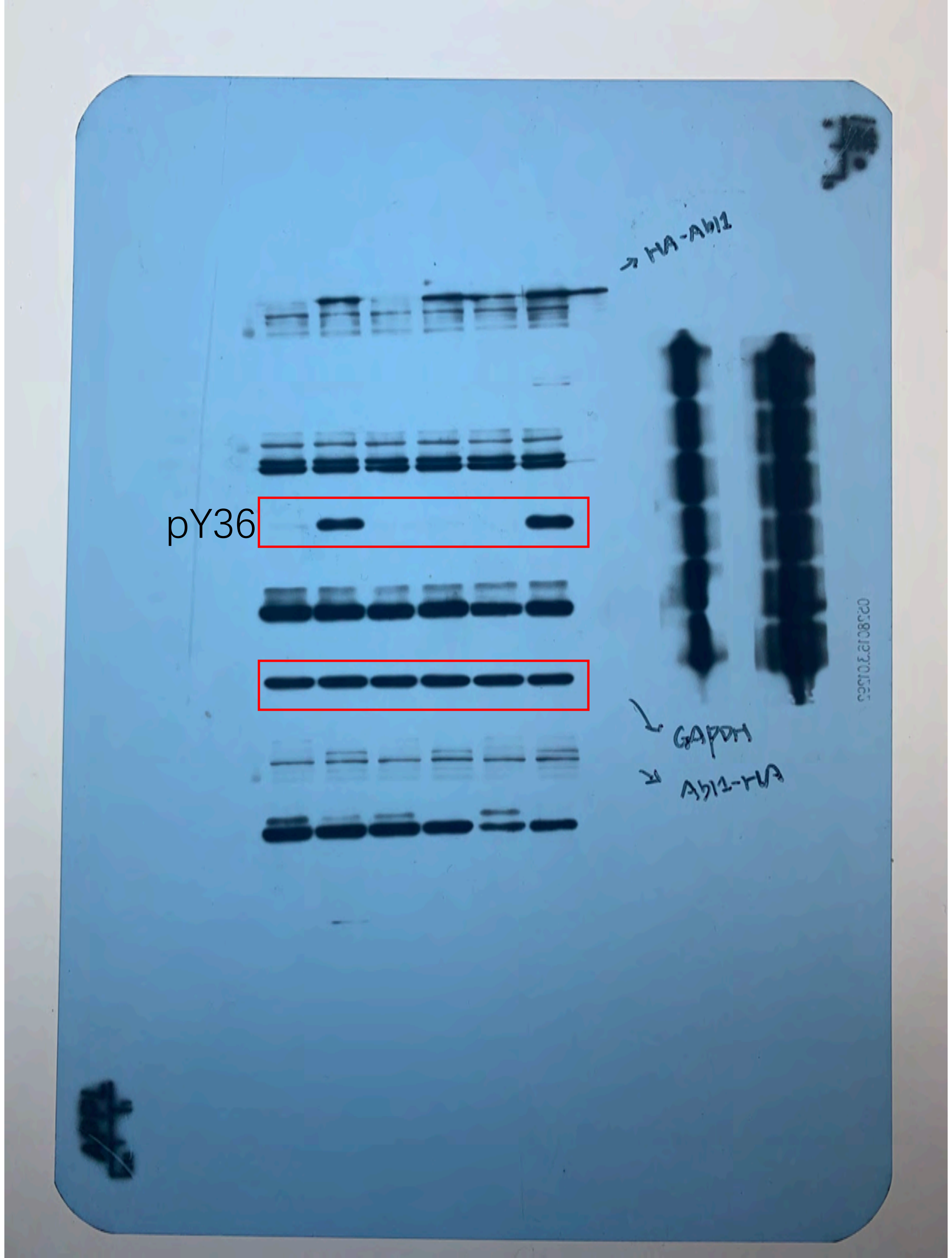

Fig. 2f

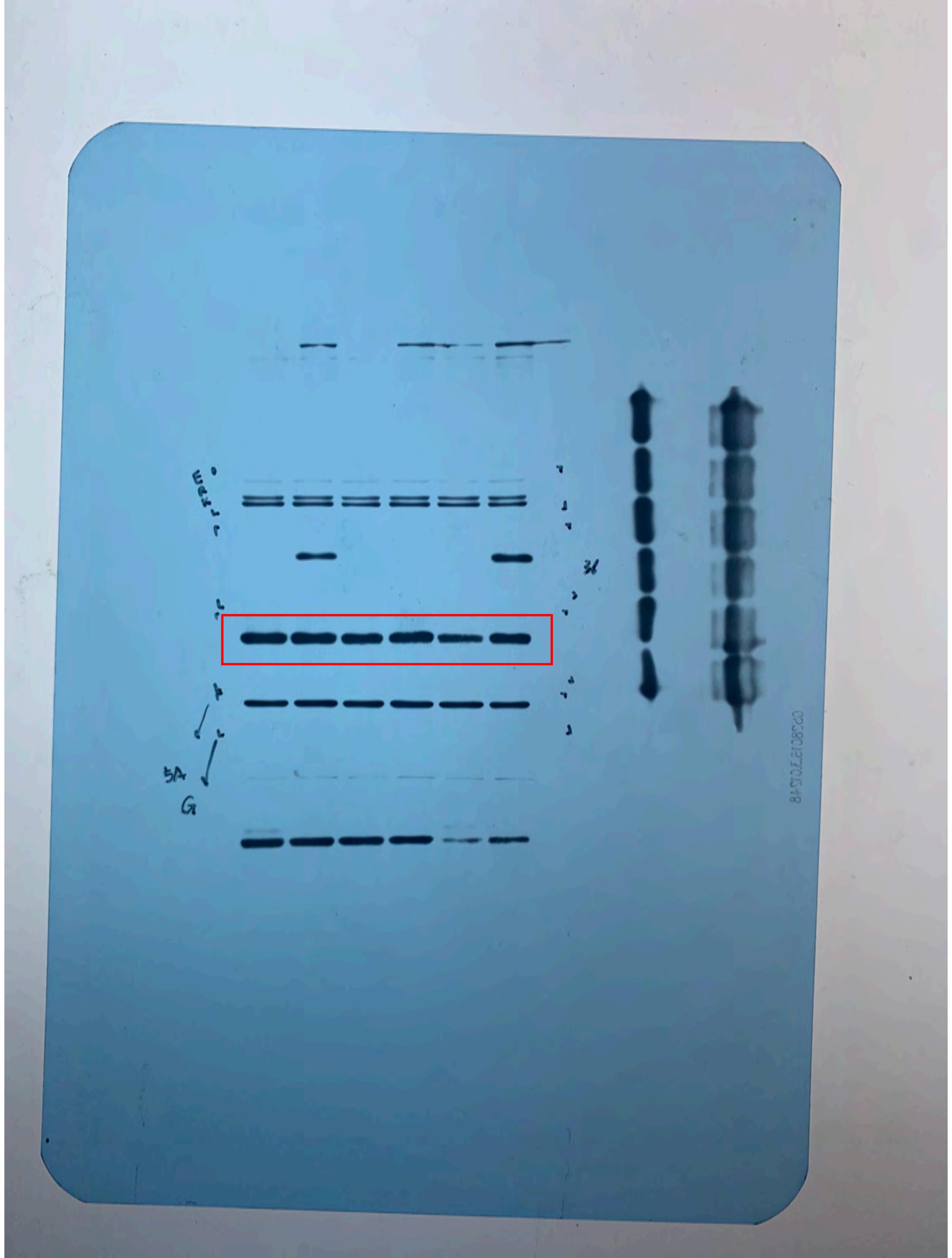

Fig. 2f

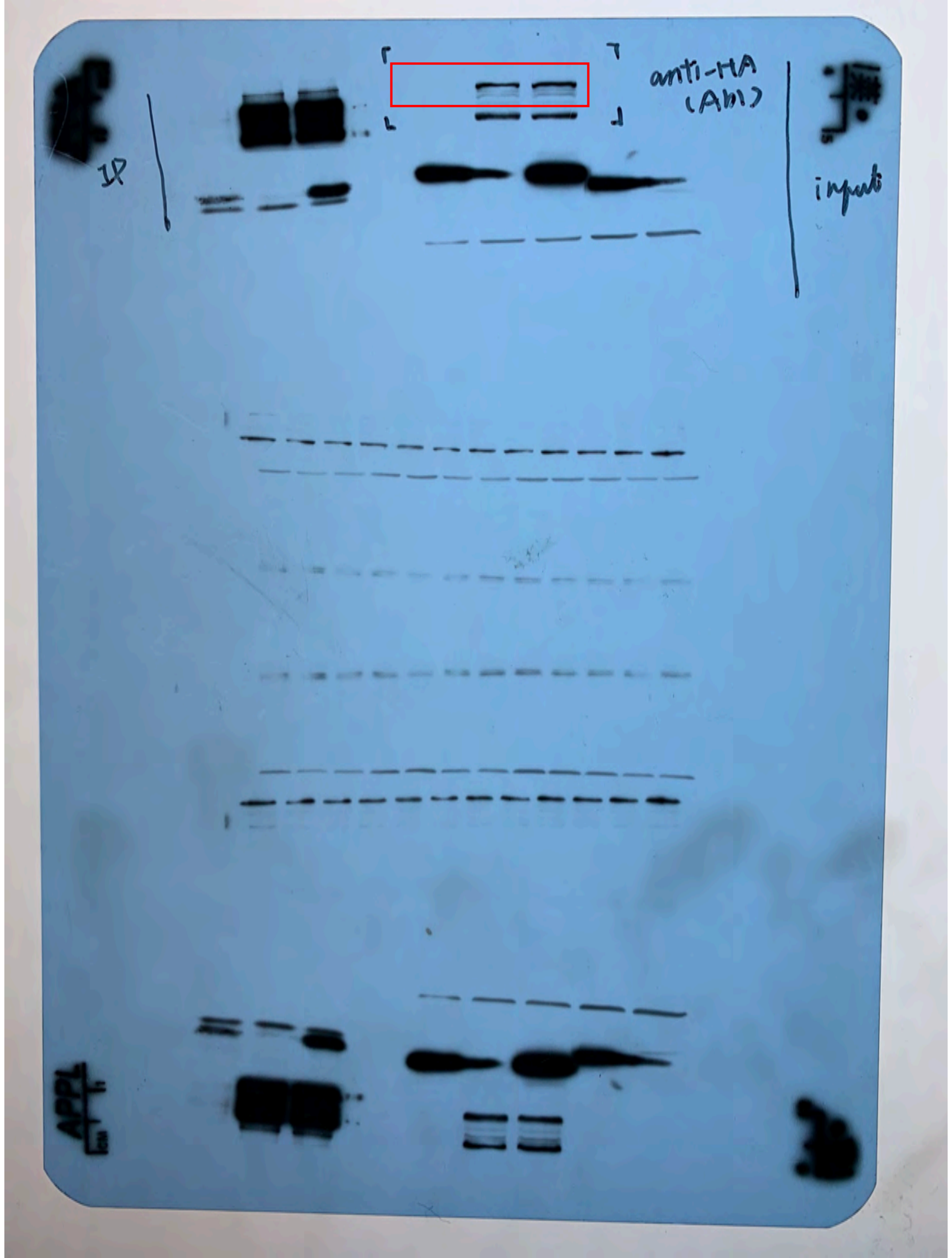

Fig. 2h

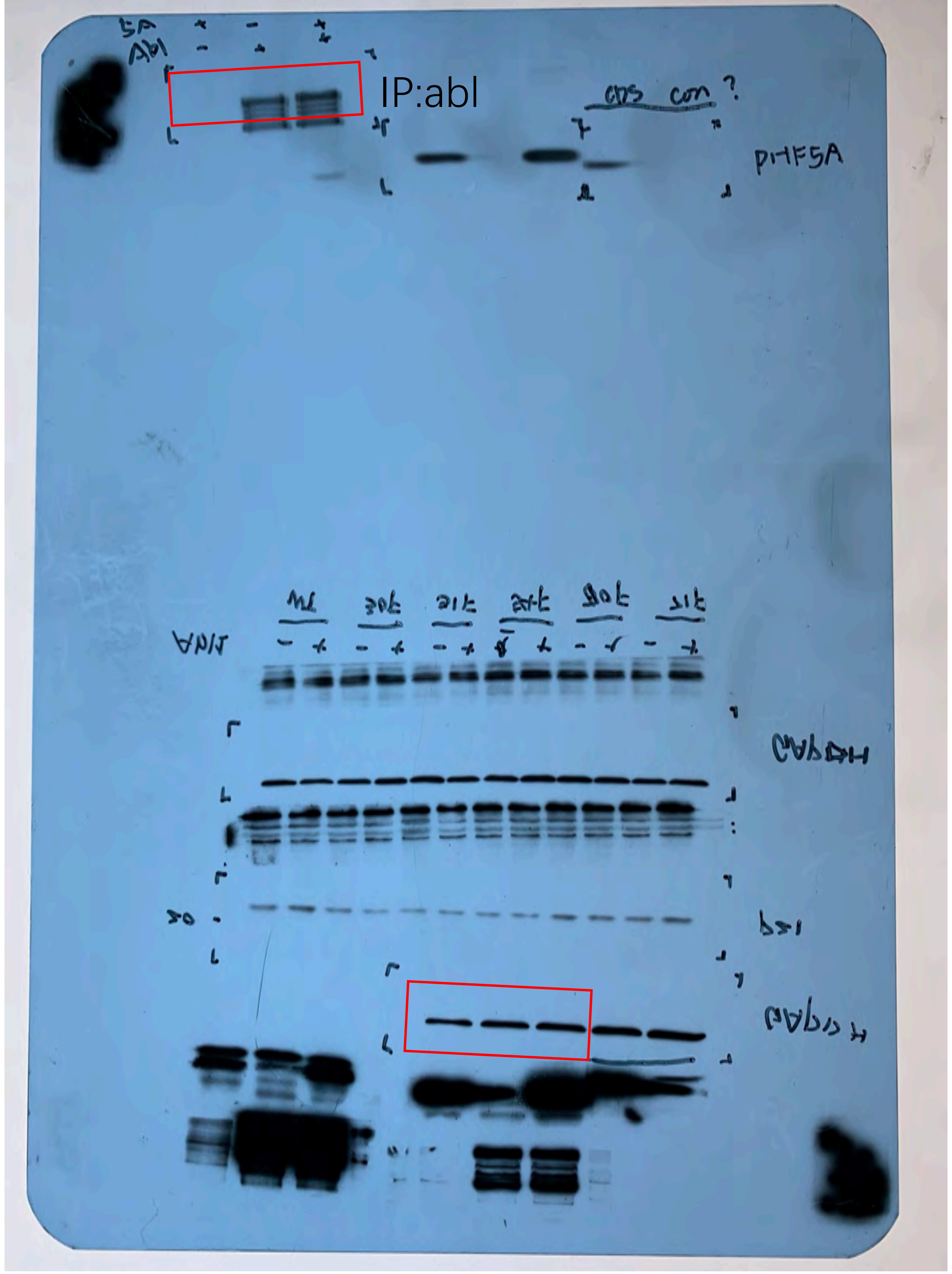

Fig. 2h

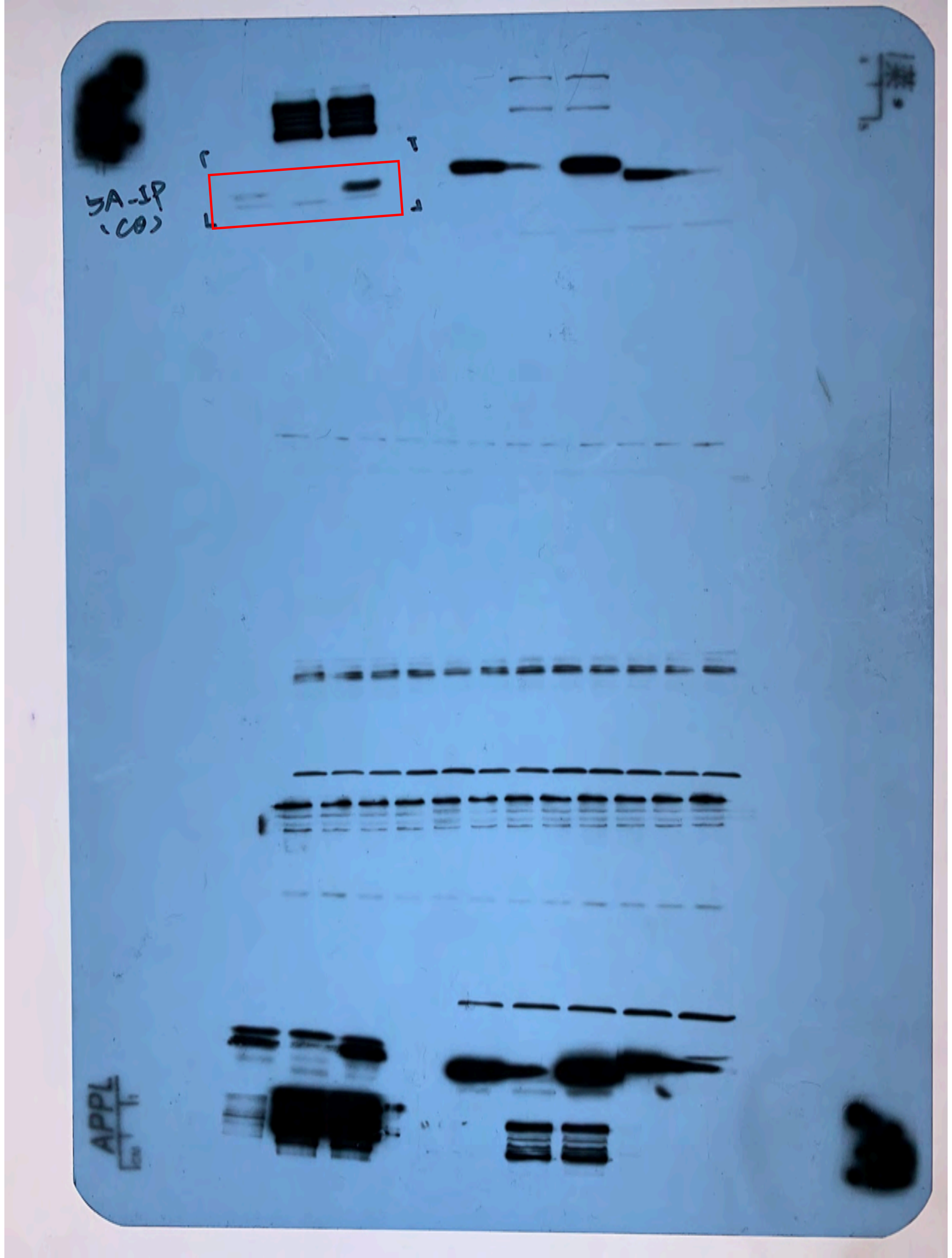

Fig. 2h

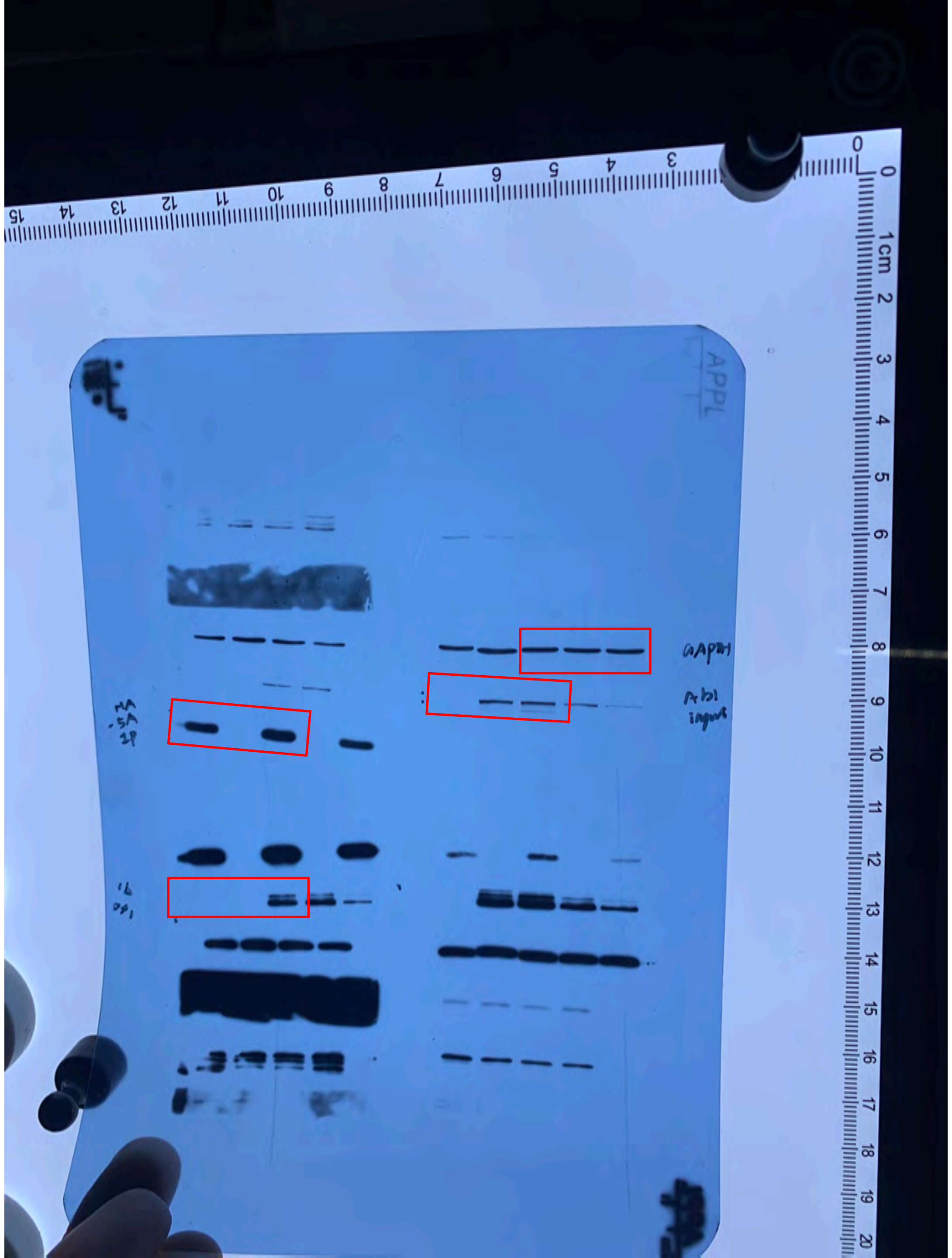

Fig. 2i

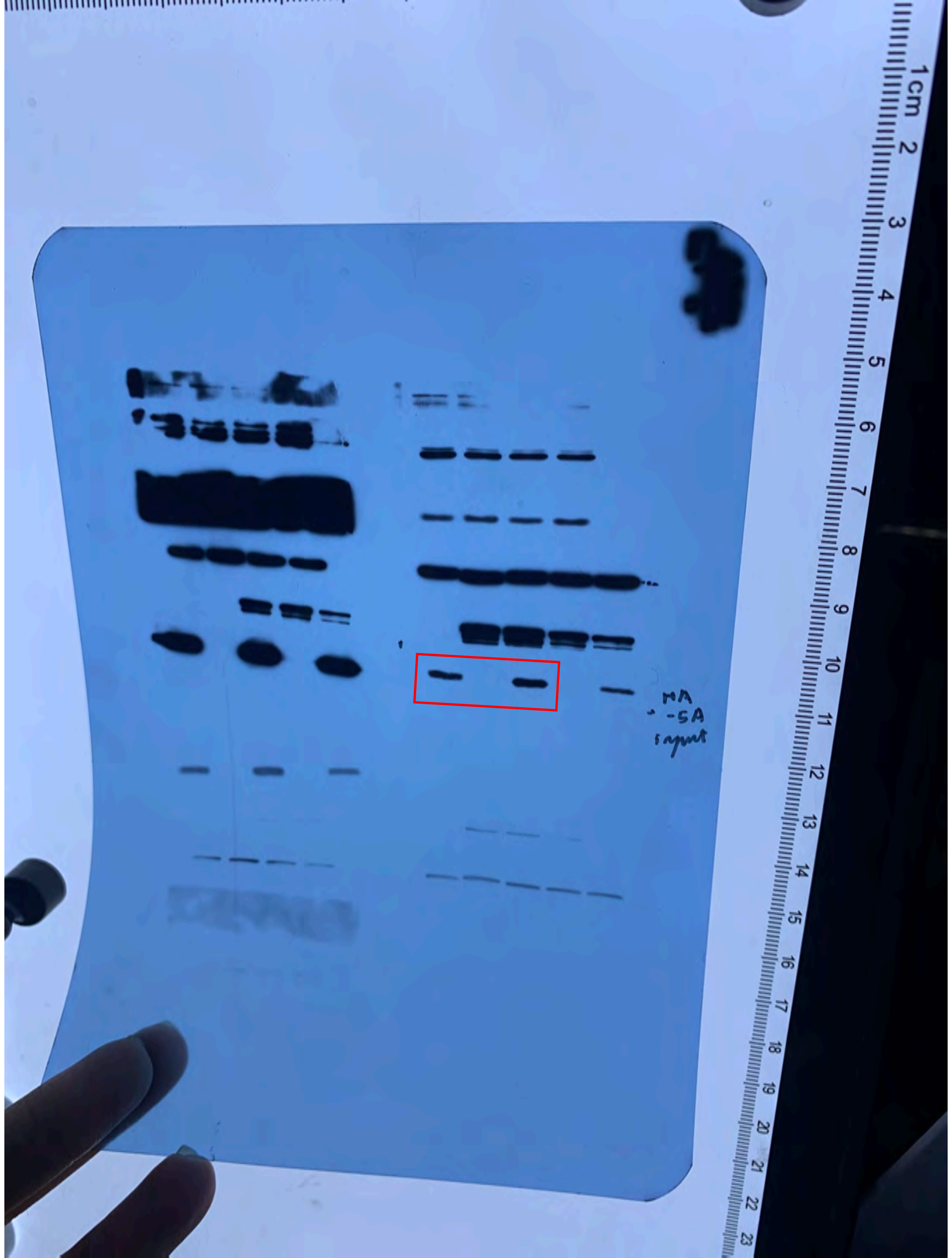

Fig. 2i

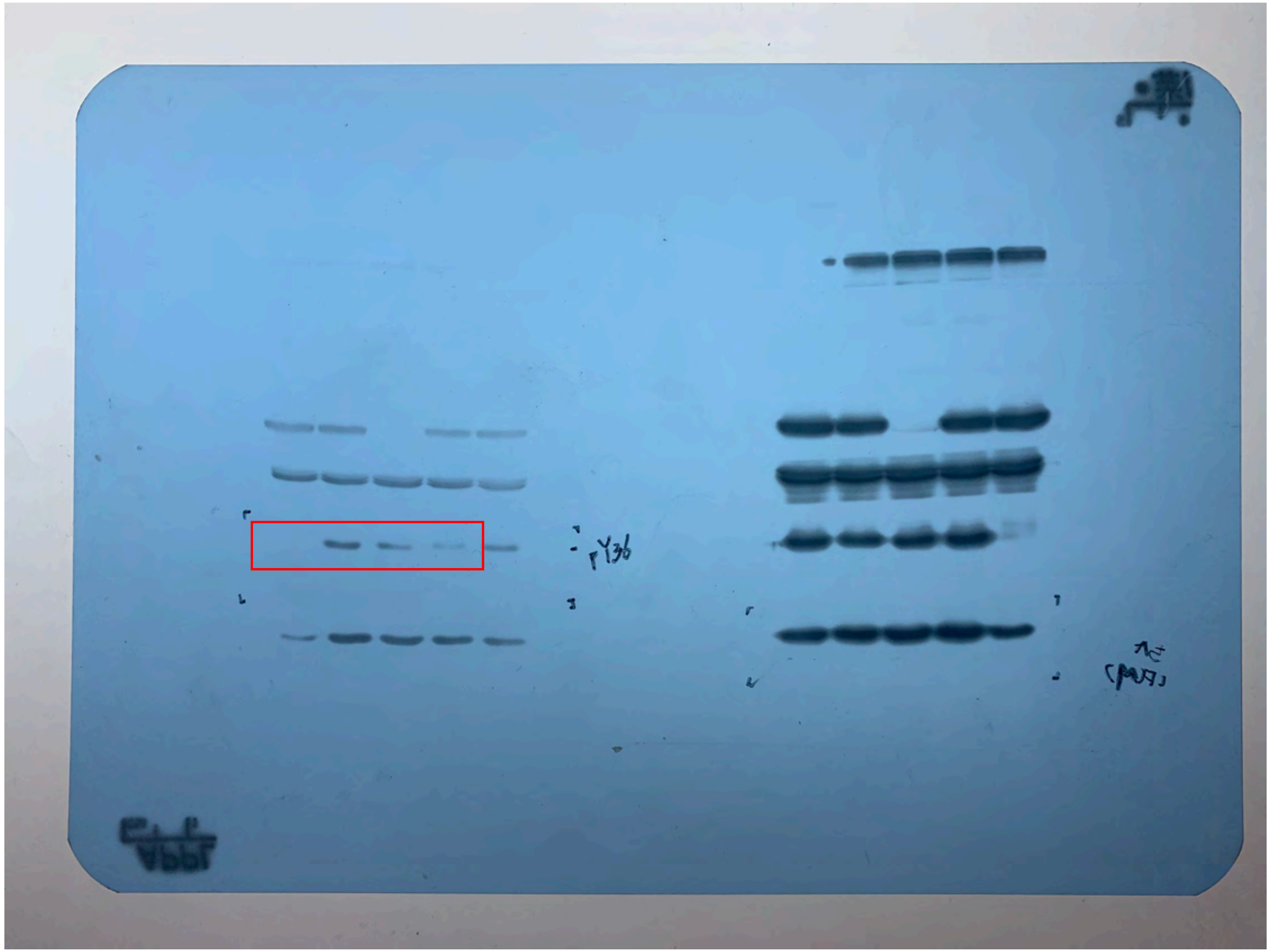

Fig. 2k

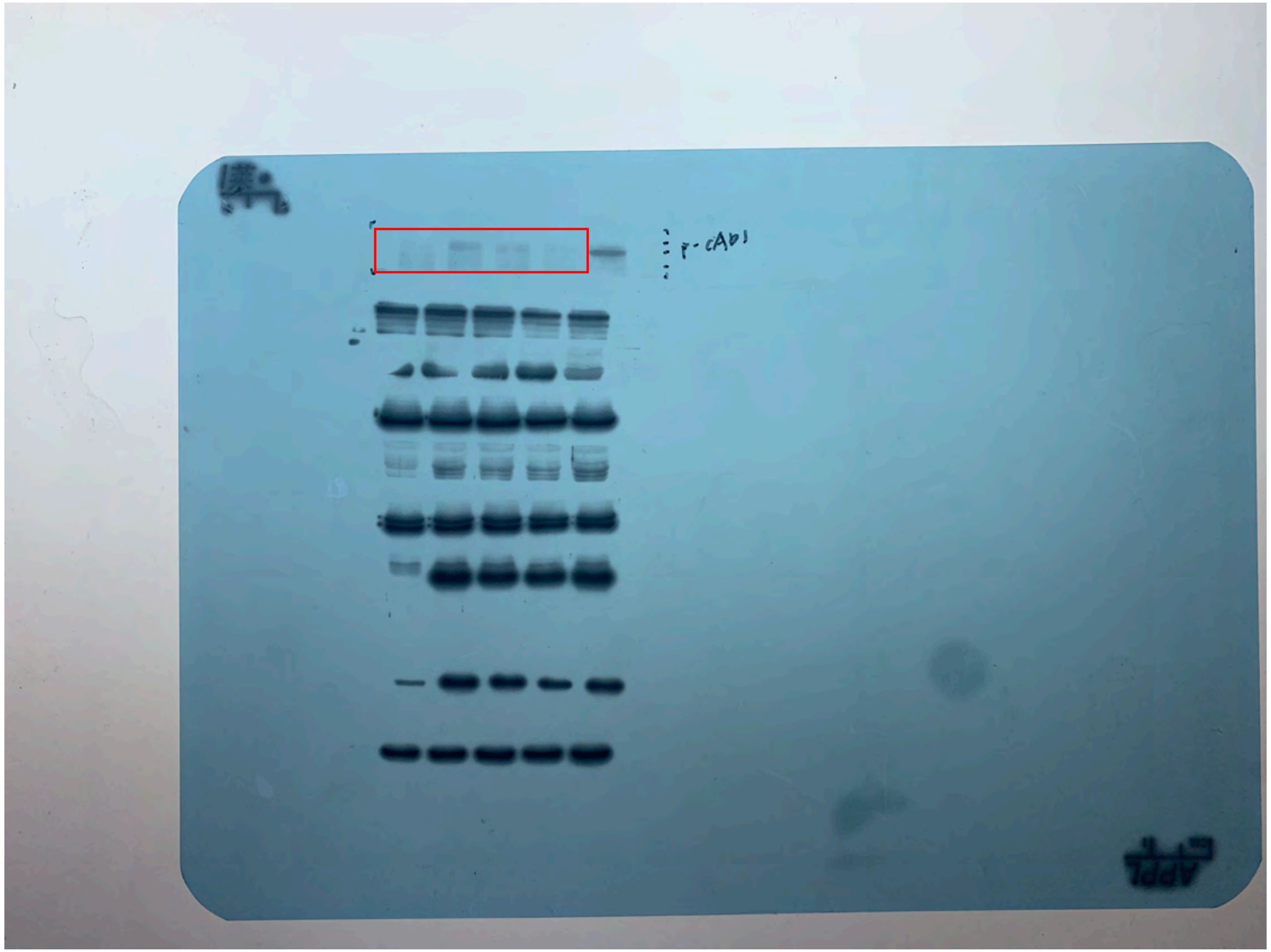

Fig. 2k

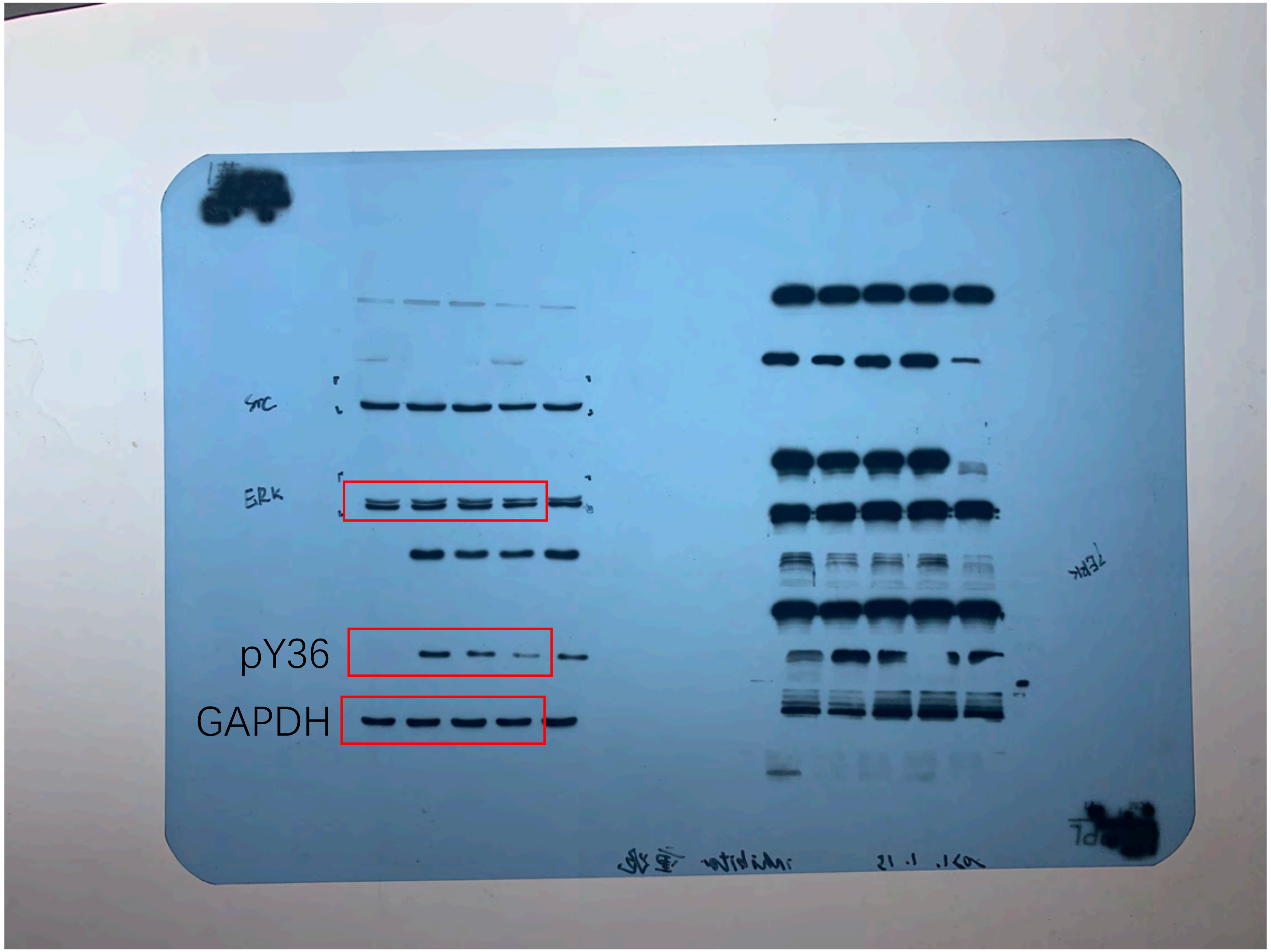

Fig. 2k

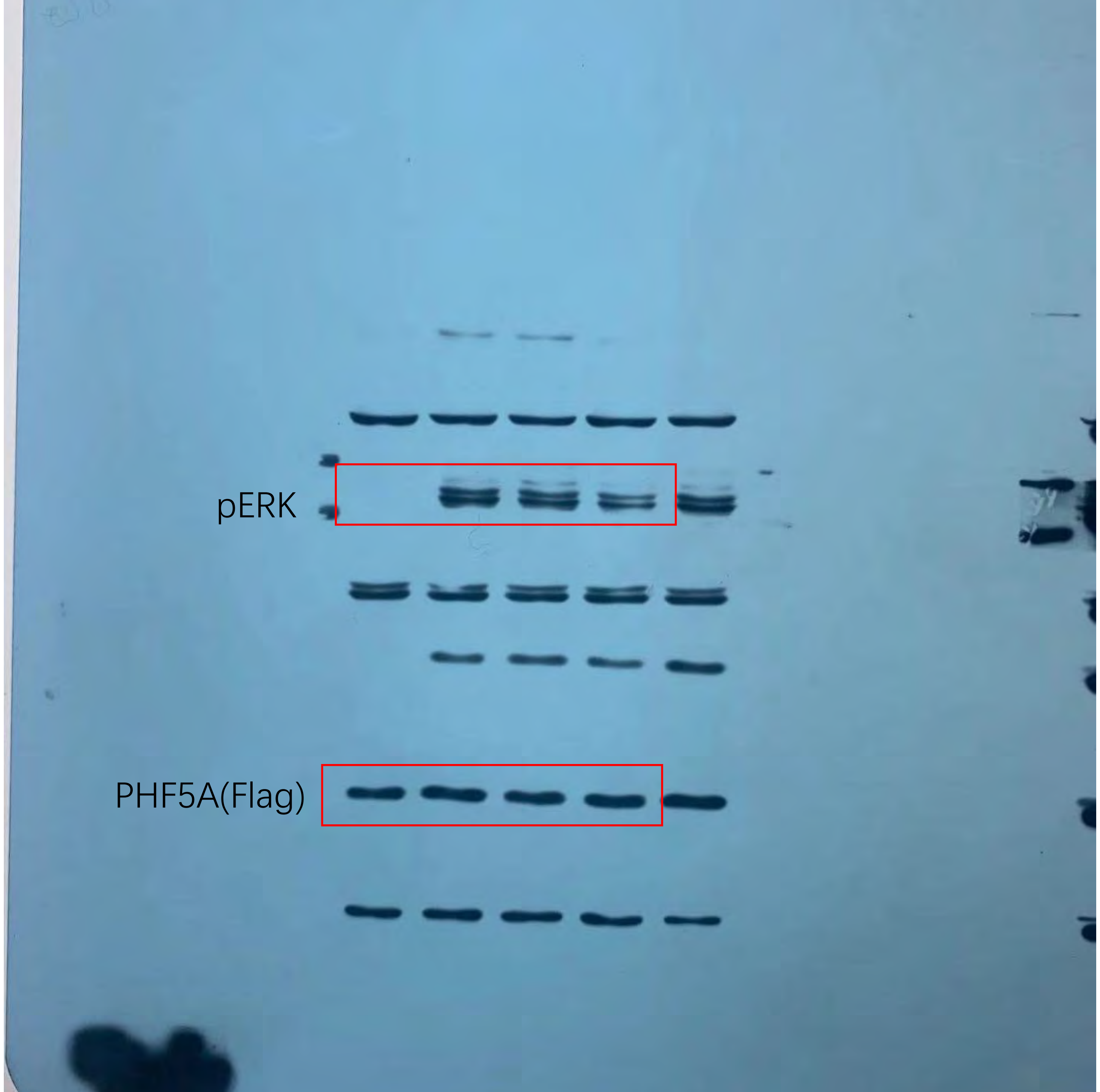

Fig. 2k

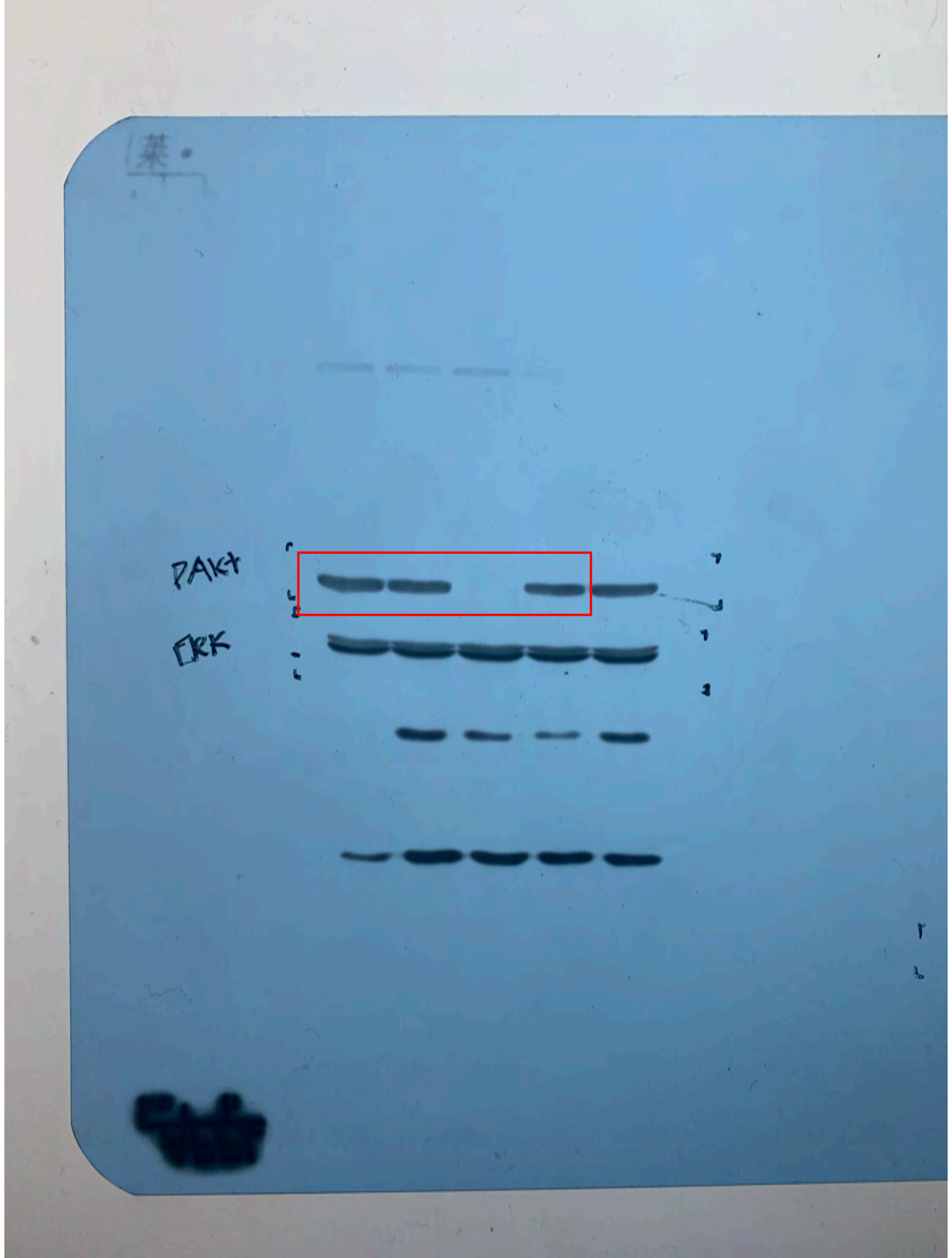

Fig. 2k

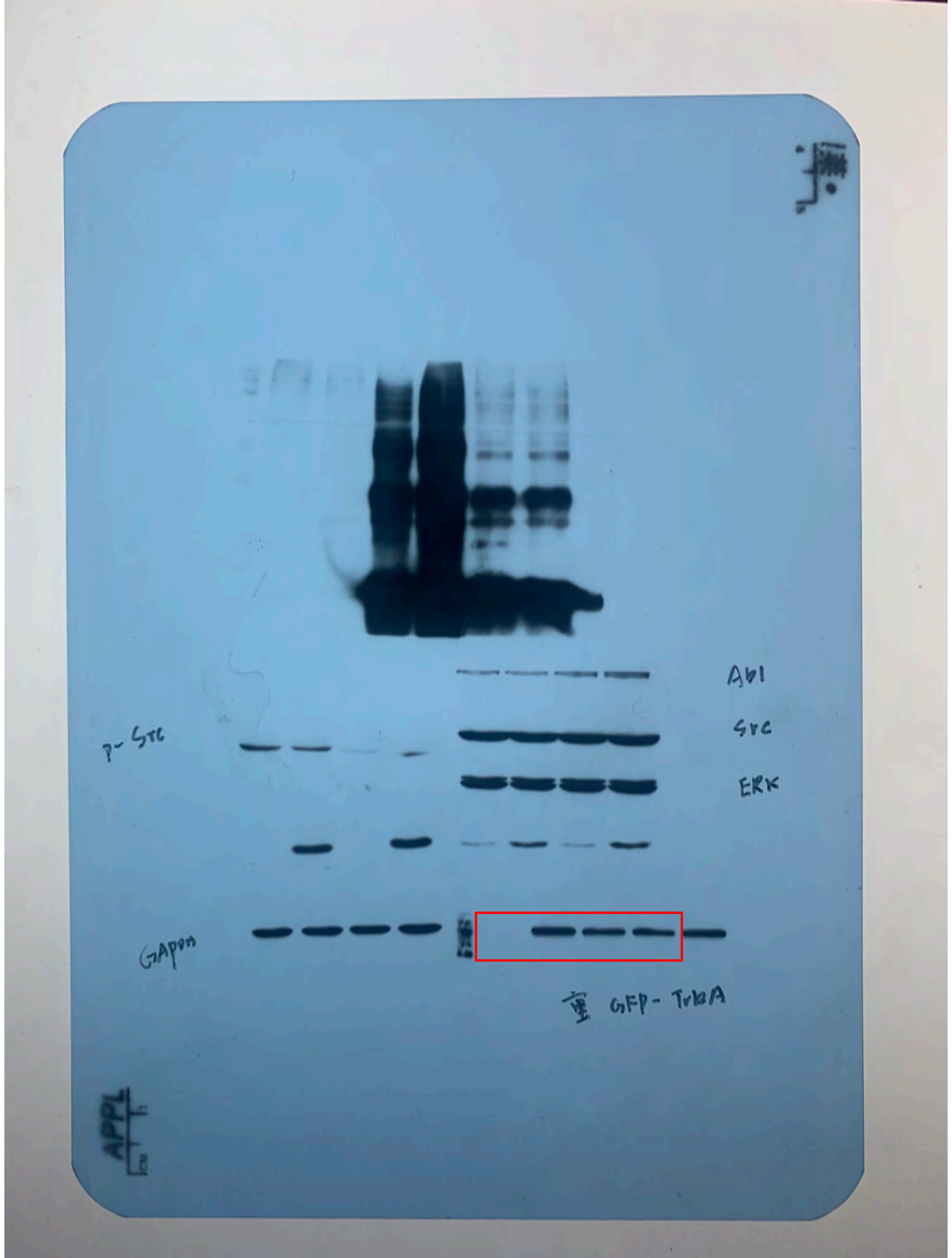

Fig. 2k

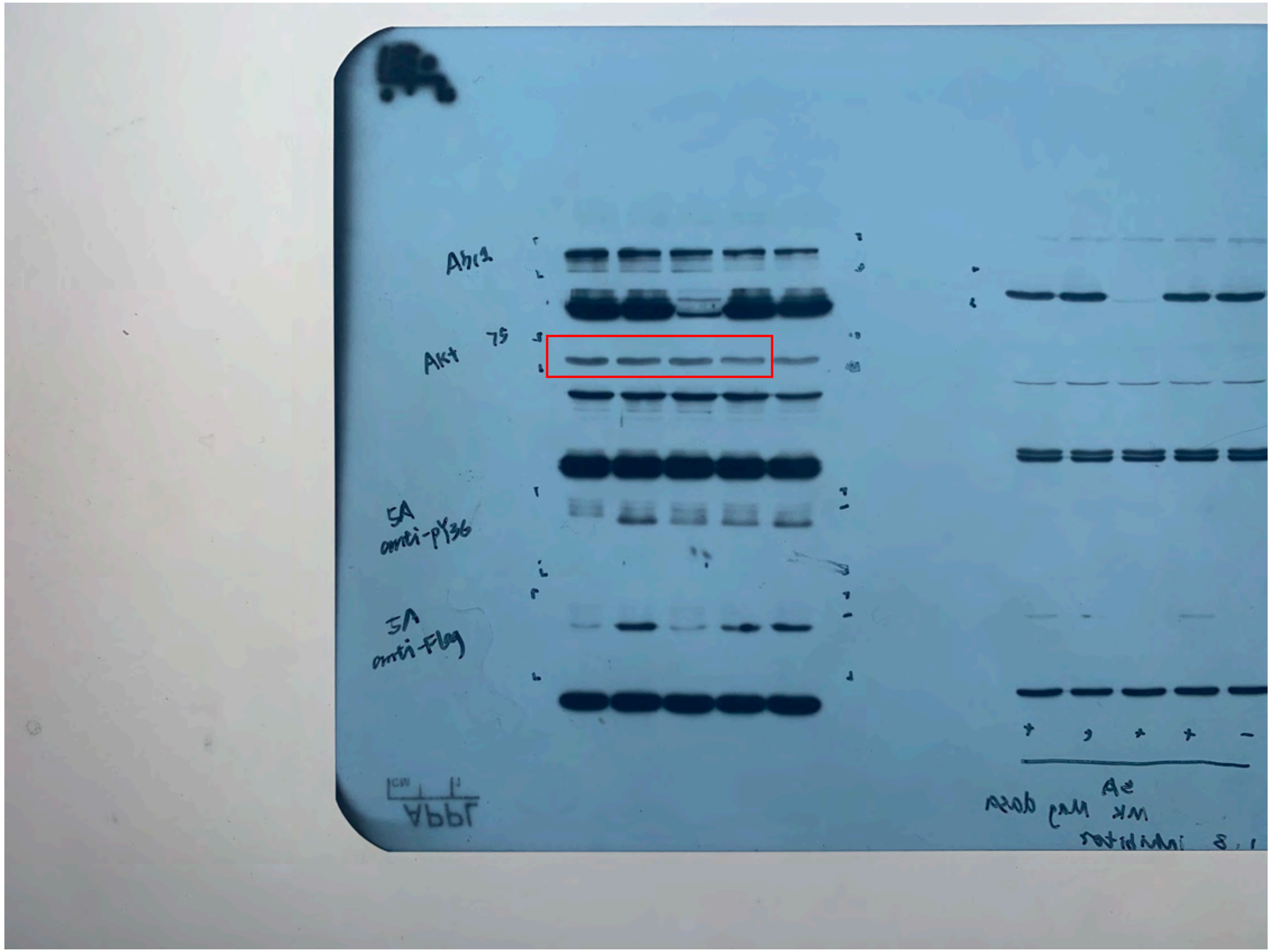

Fig. 2k

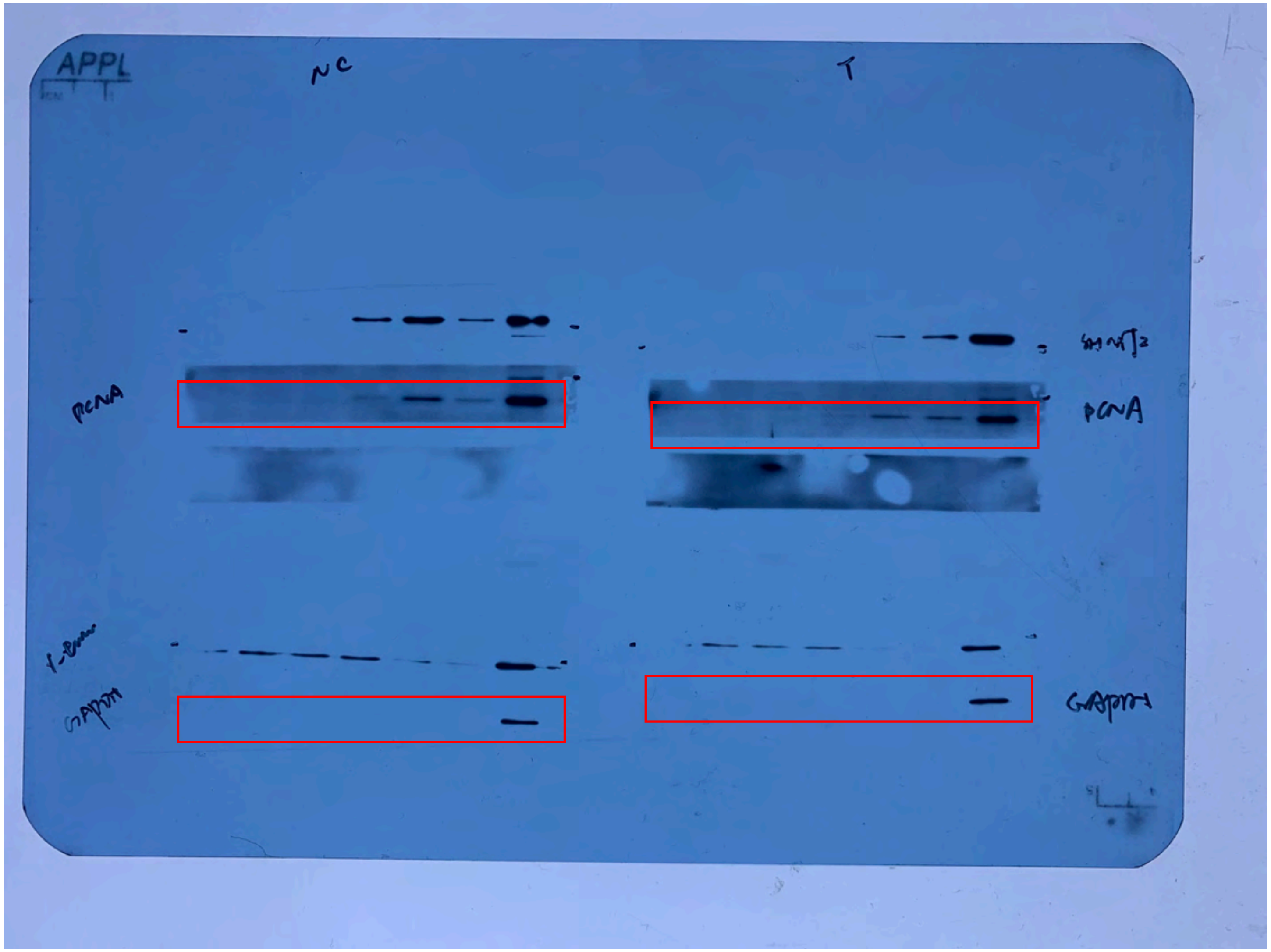

Fig. 3d

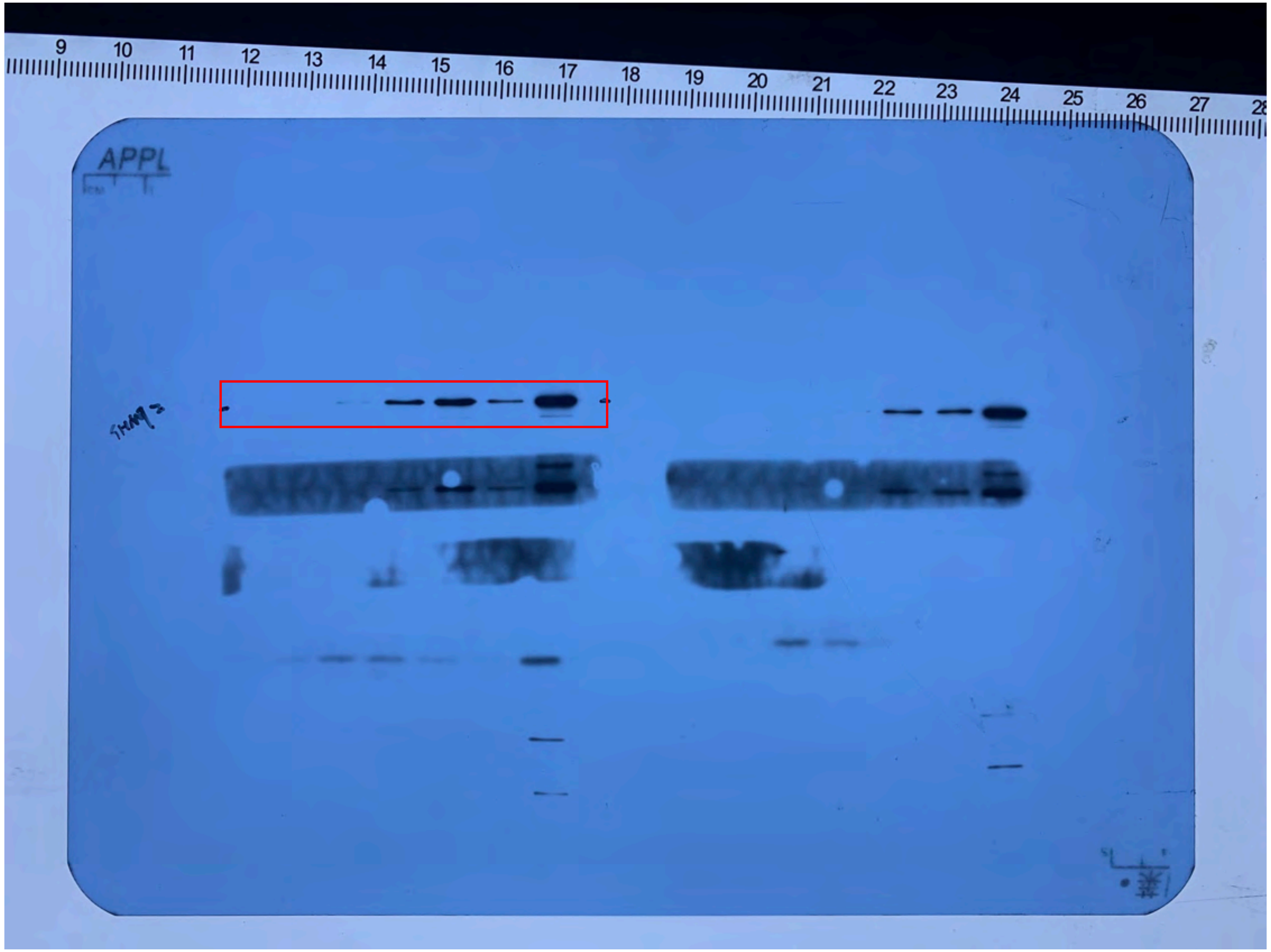

Fig. 3d

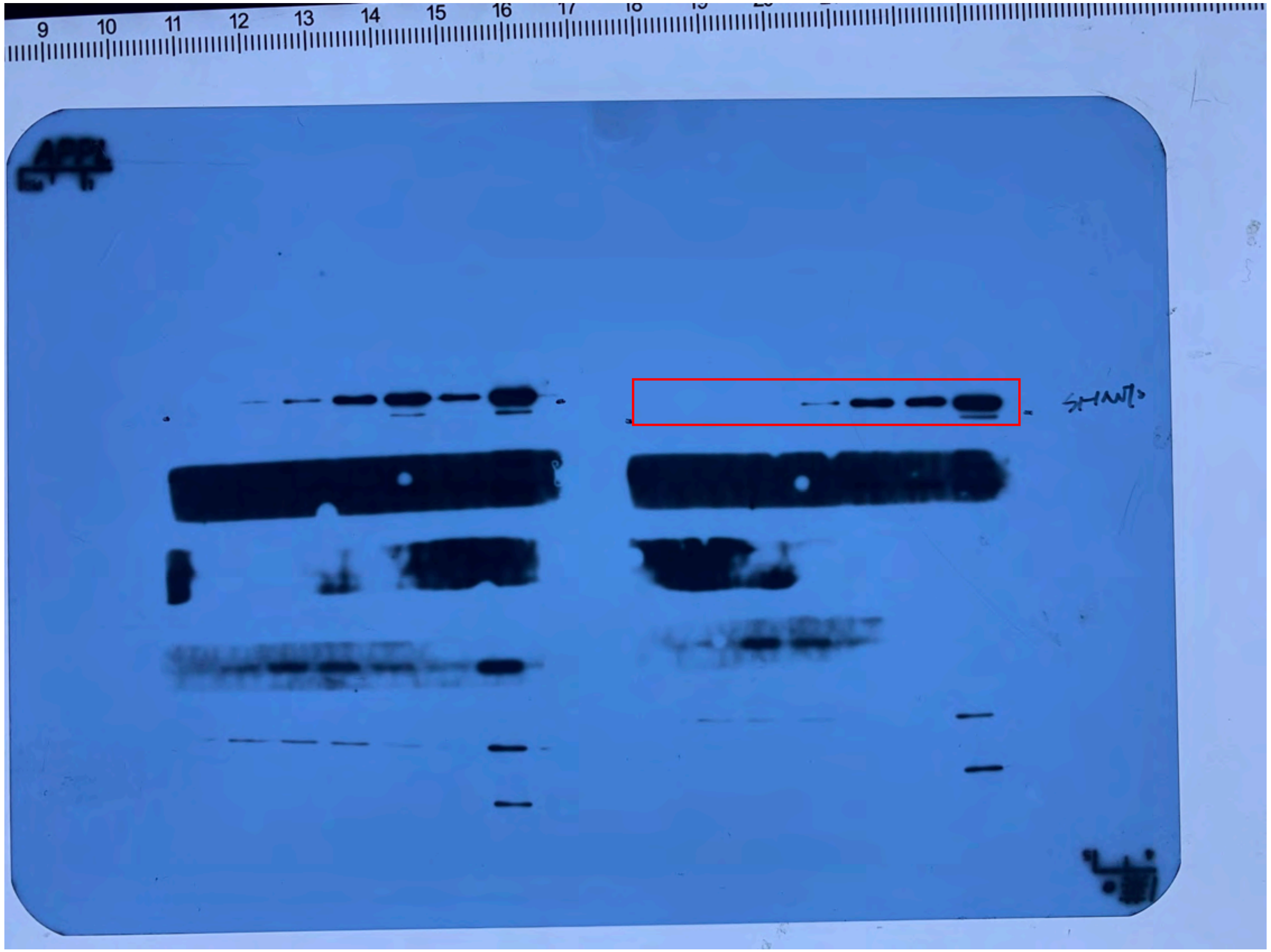

Fig. 3d

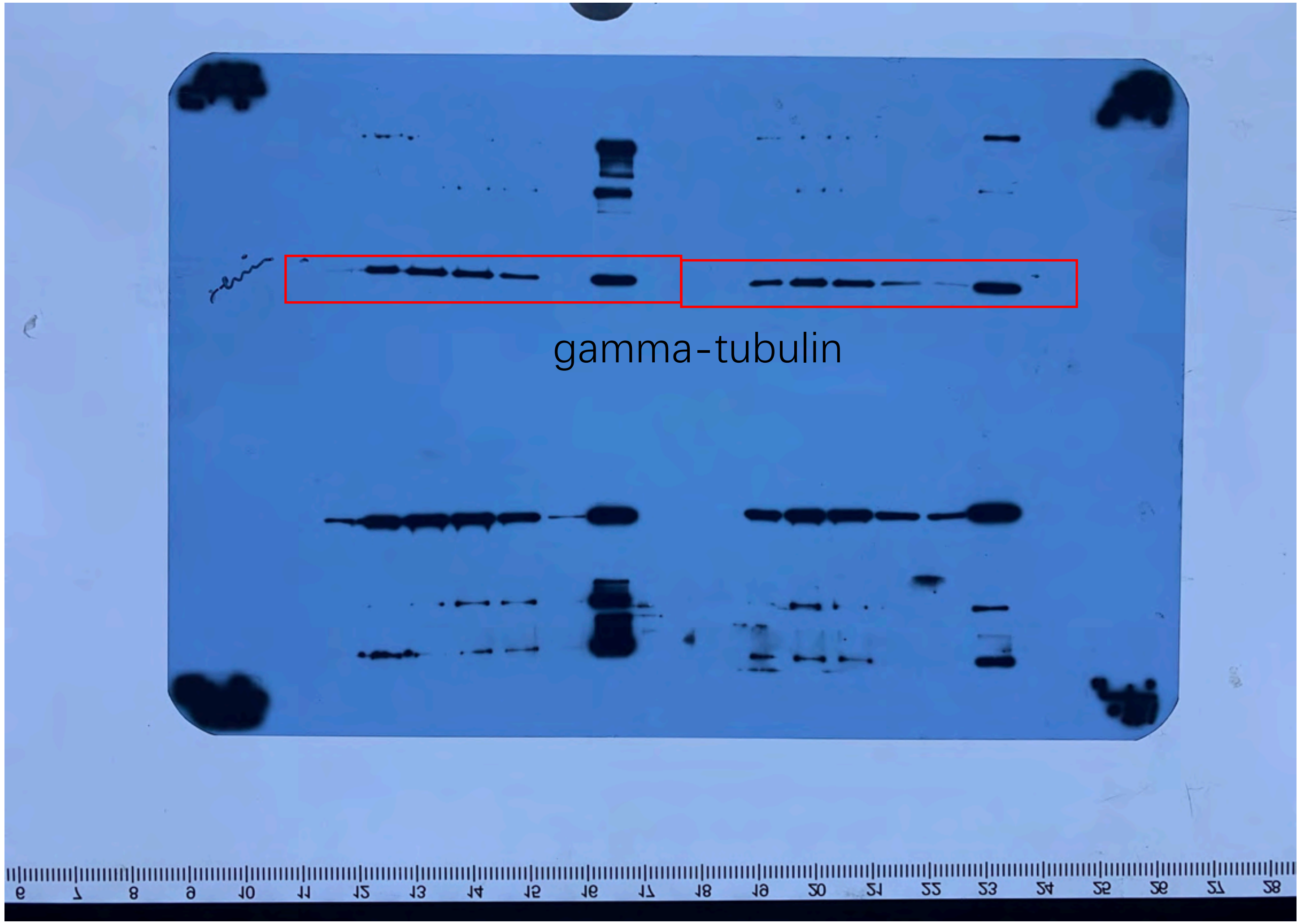

Fig. 3d

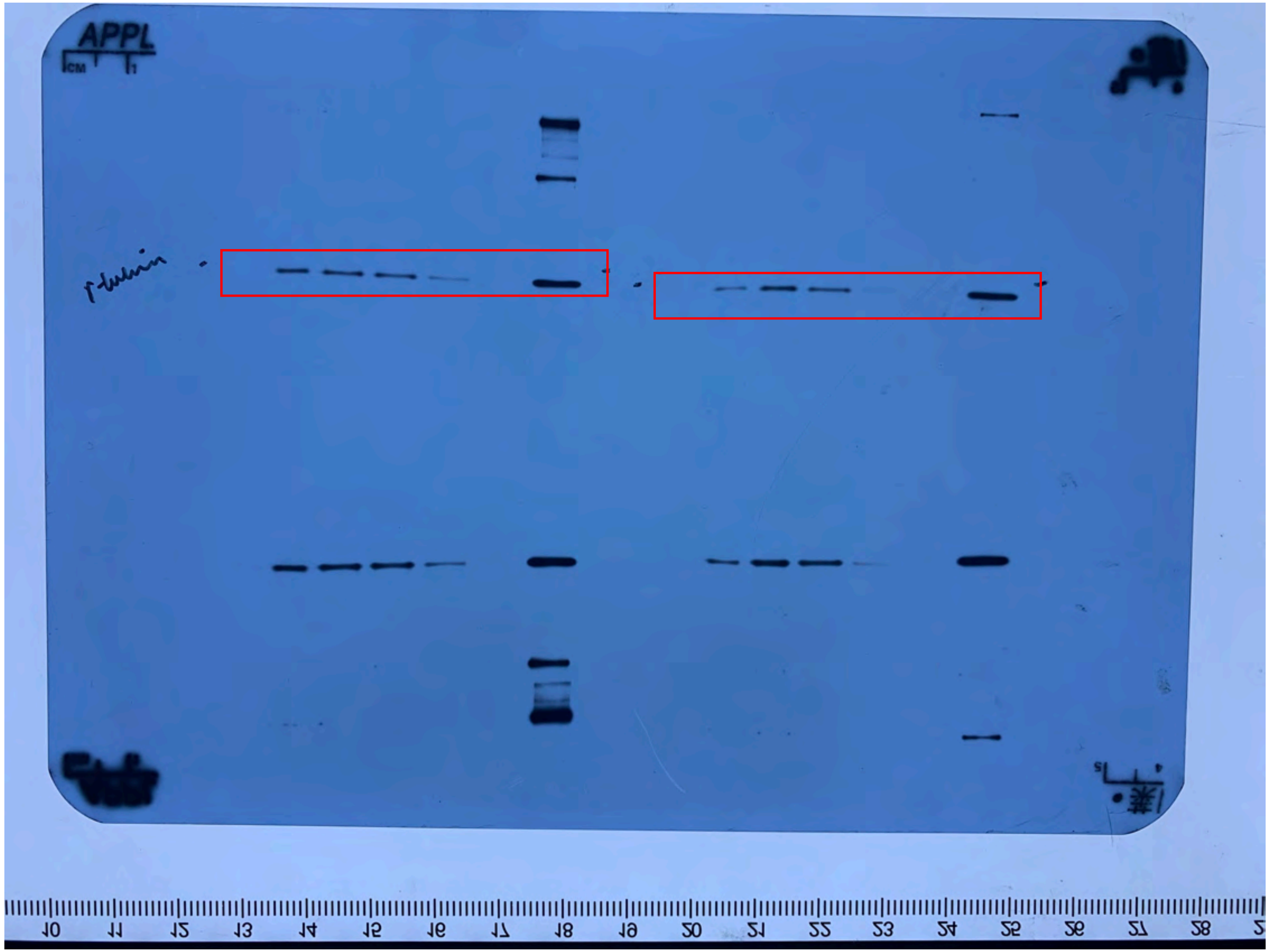

Fig. 3d

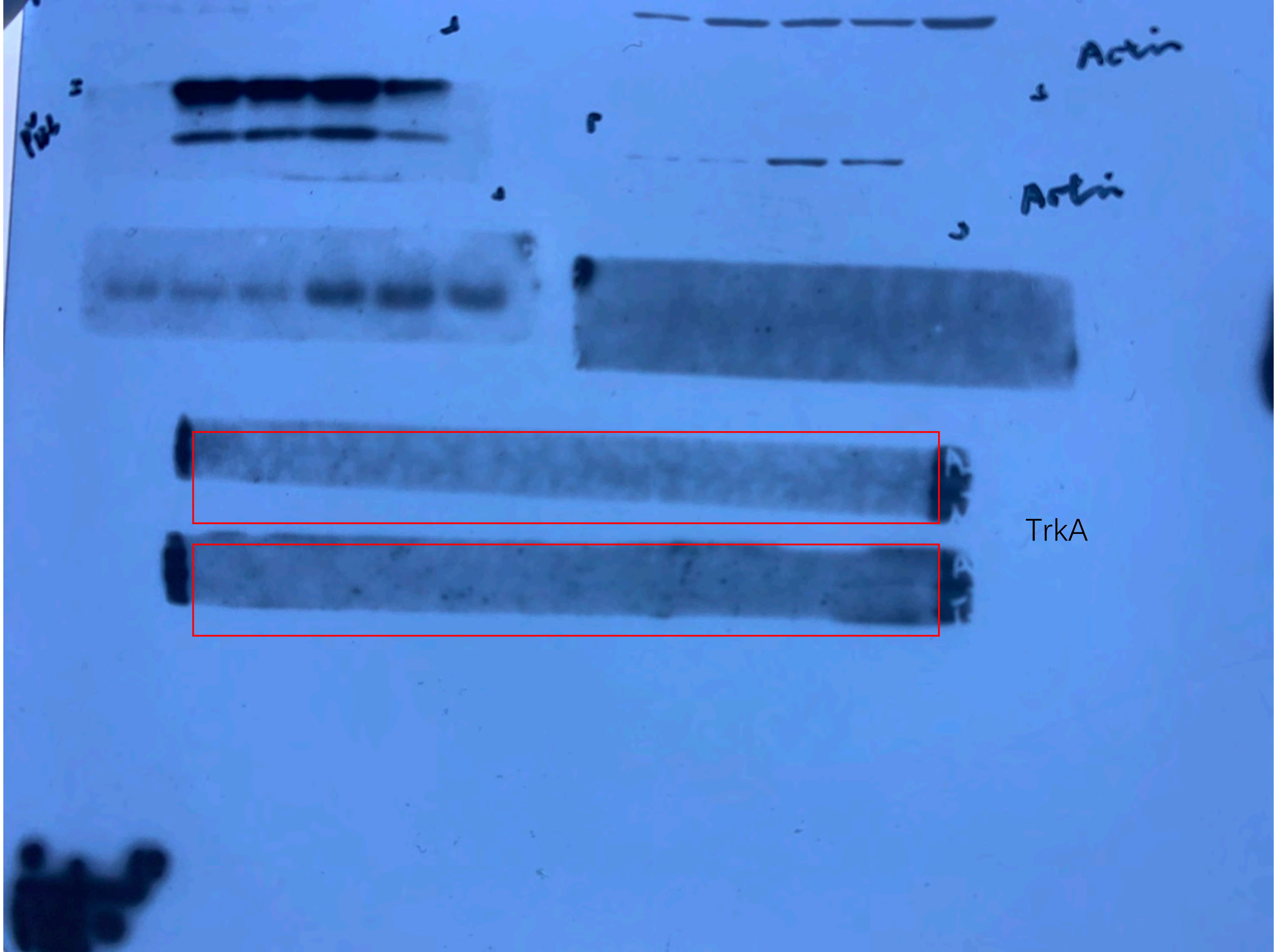

Fig. 3d

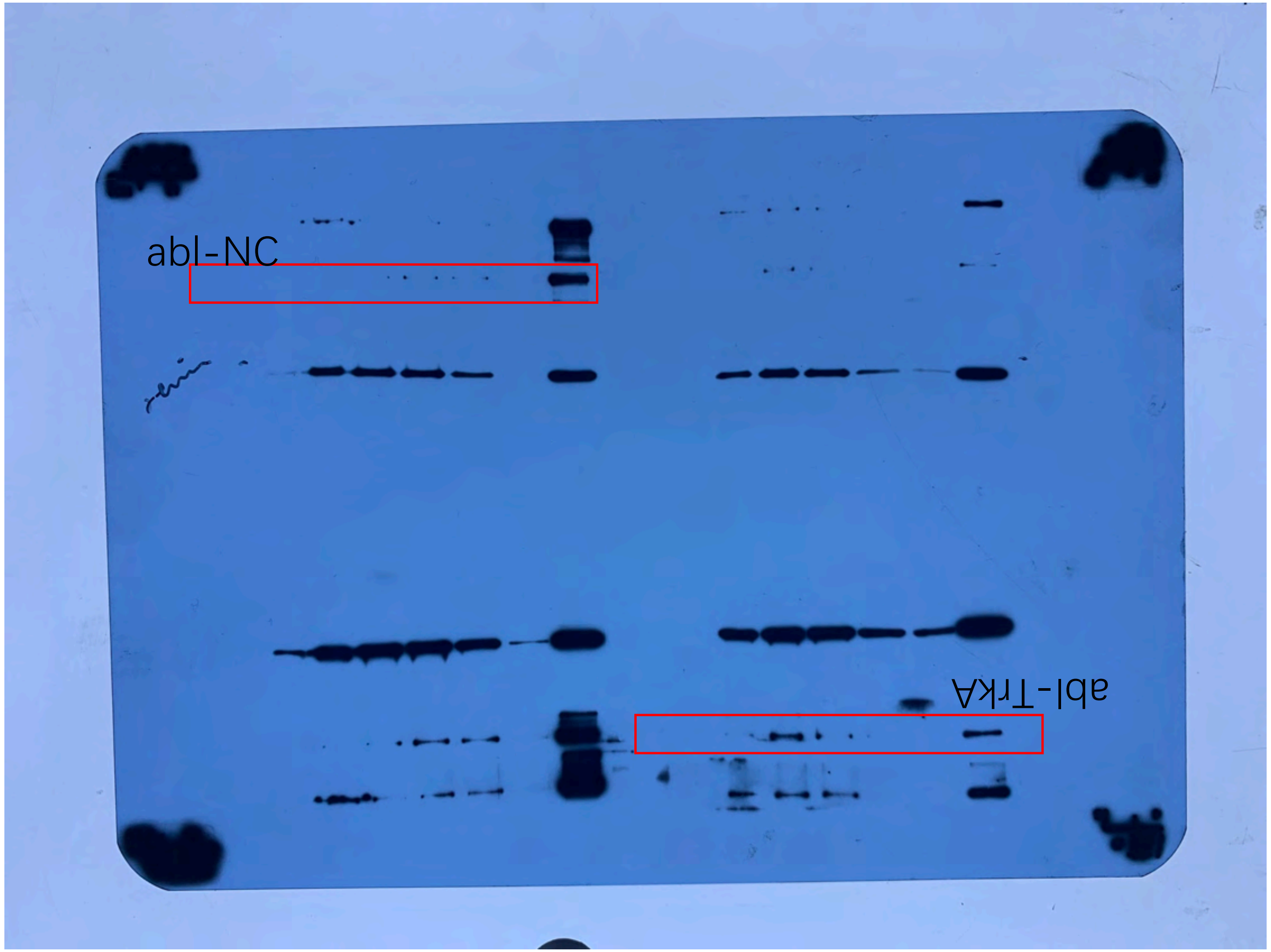

Fig. 3d

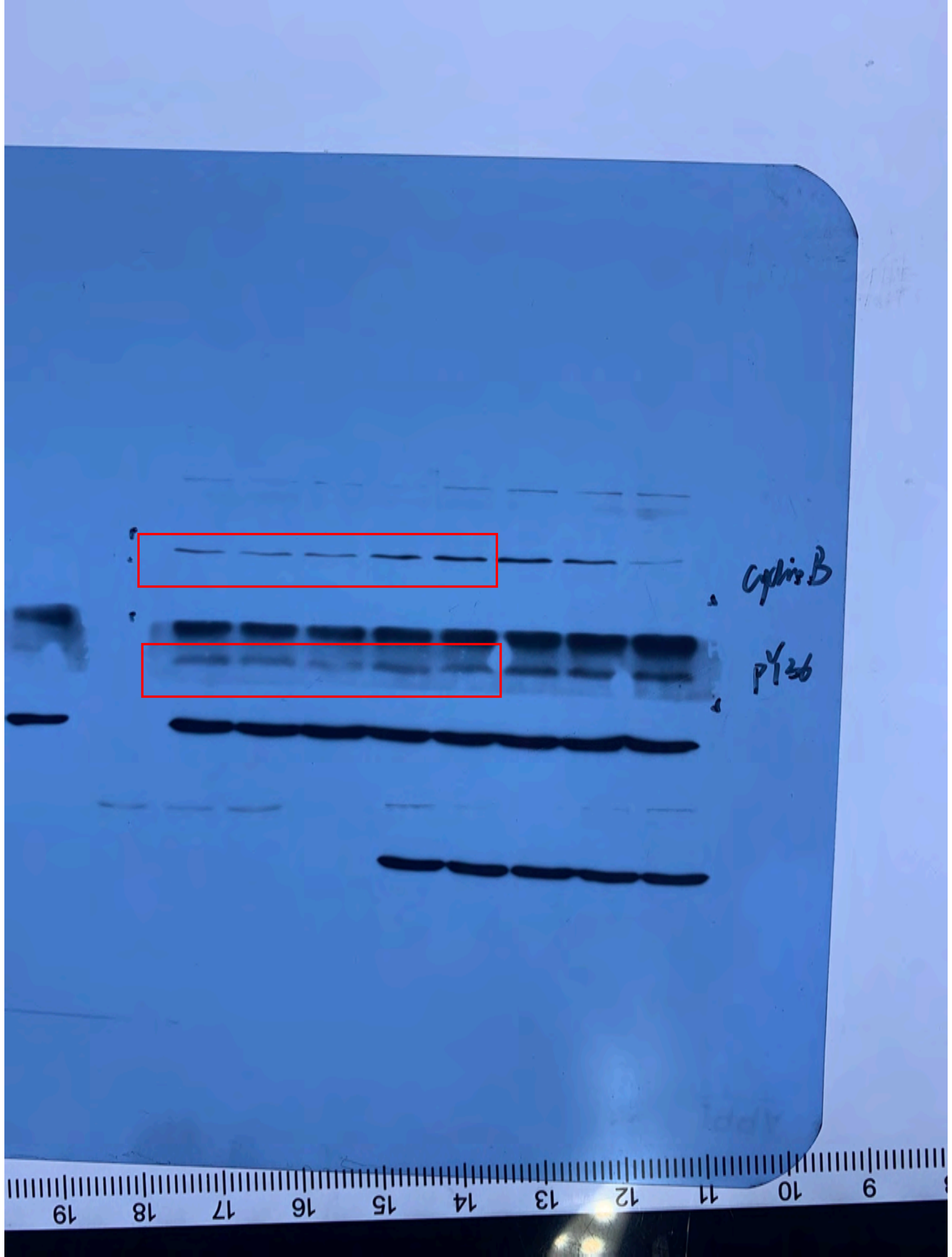

Fig. 4g

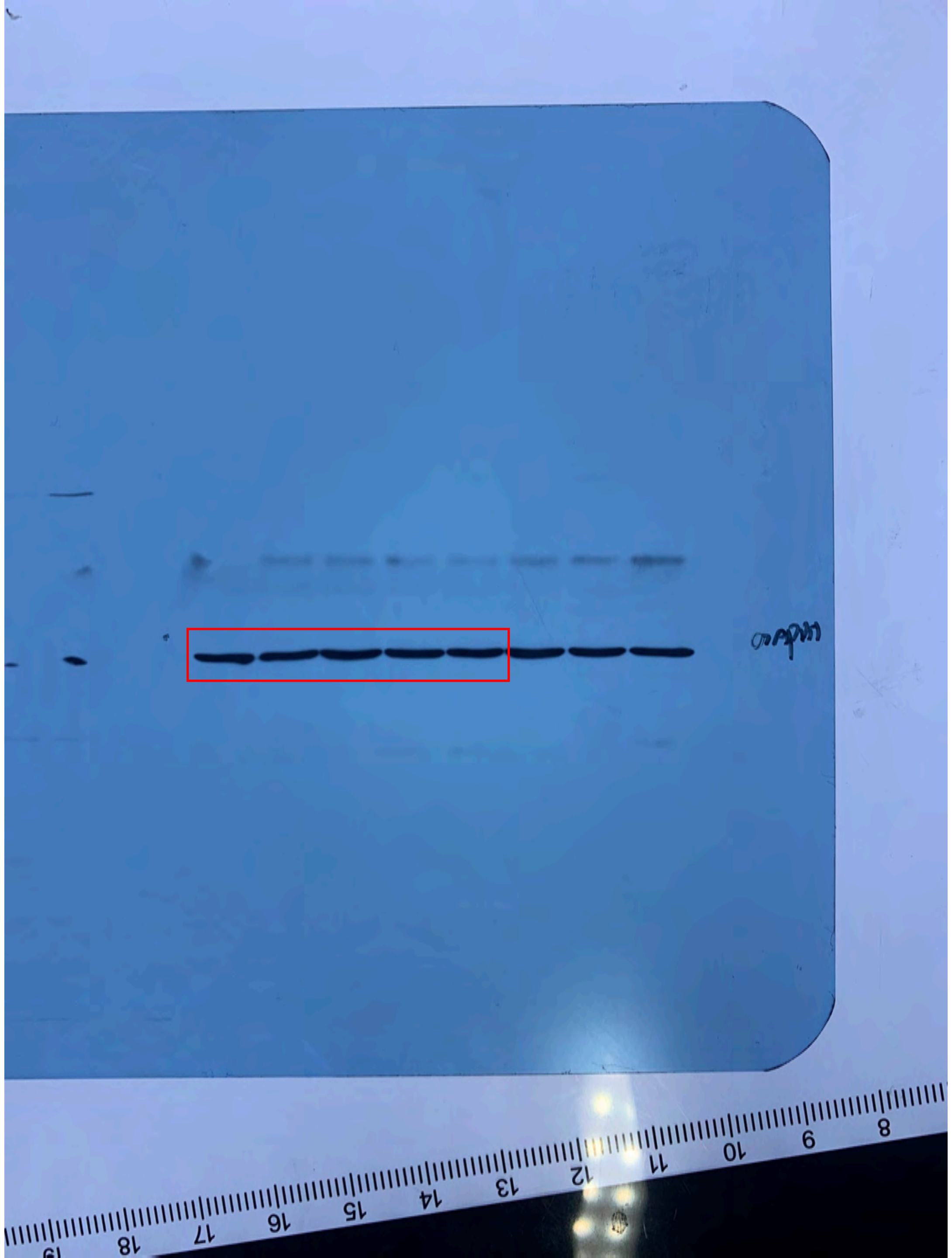

Fig. 4g

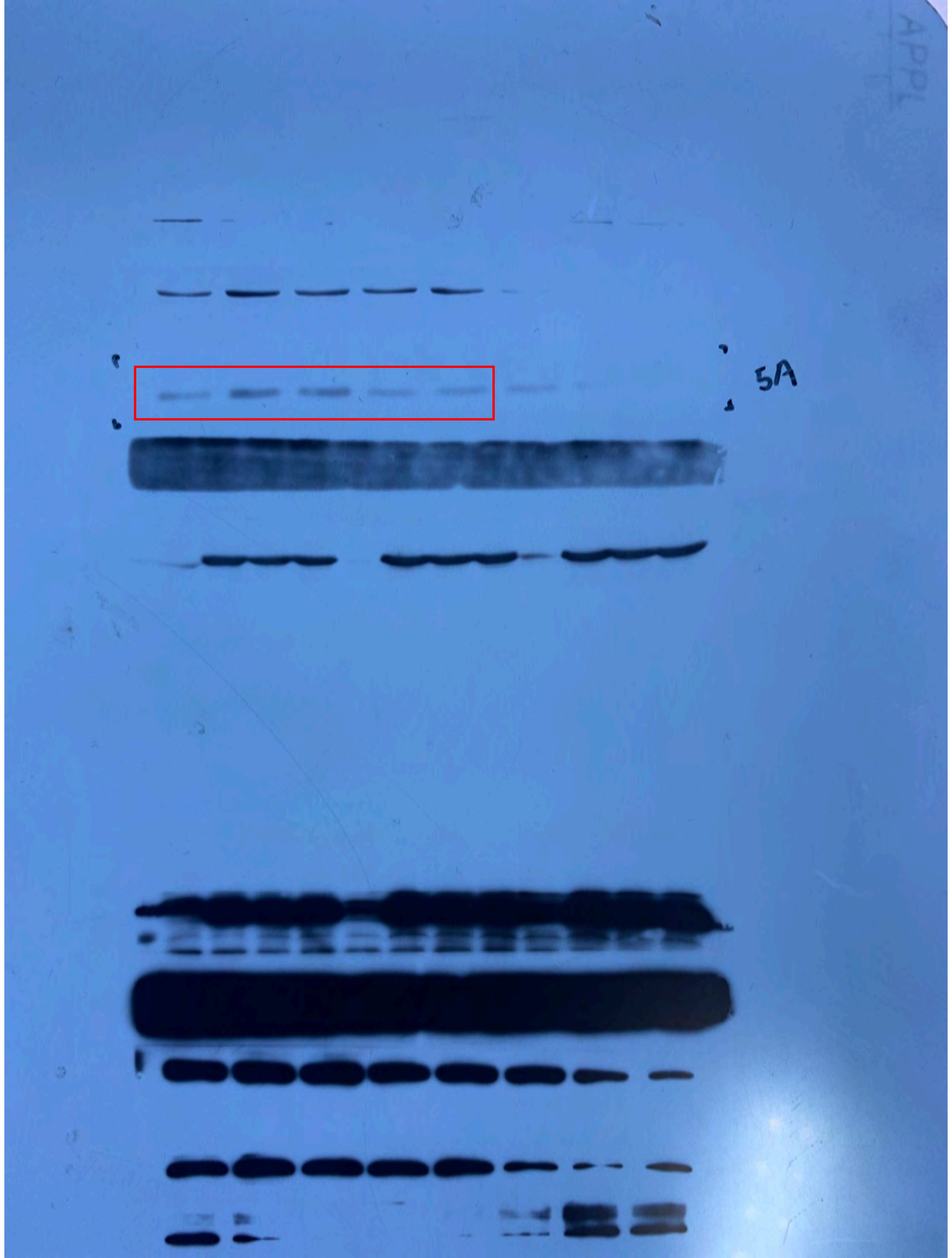
5A

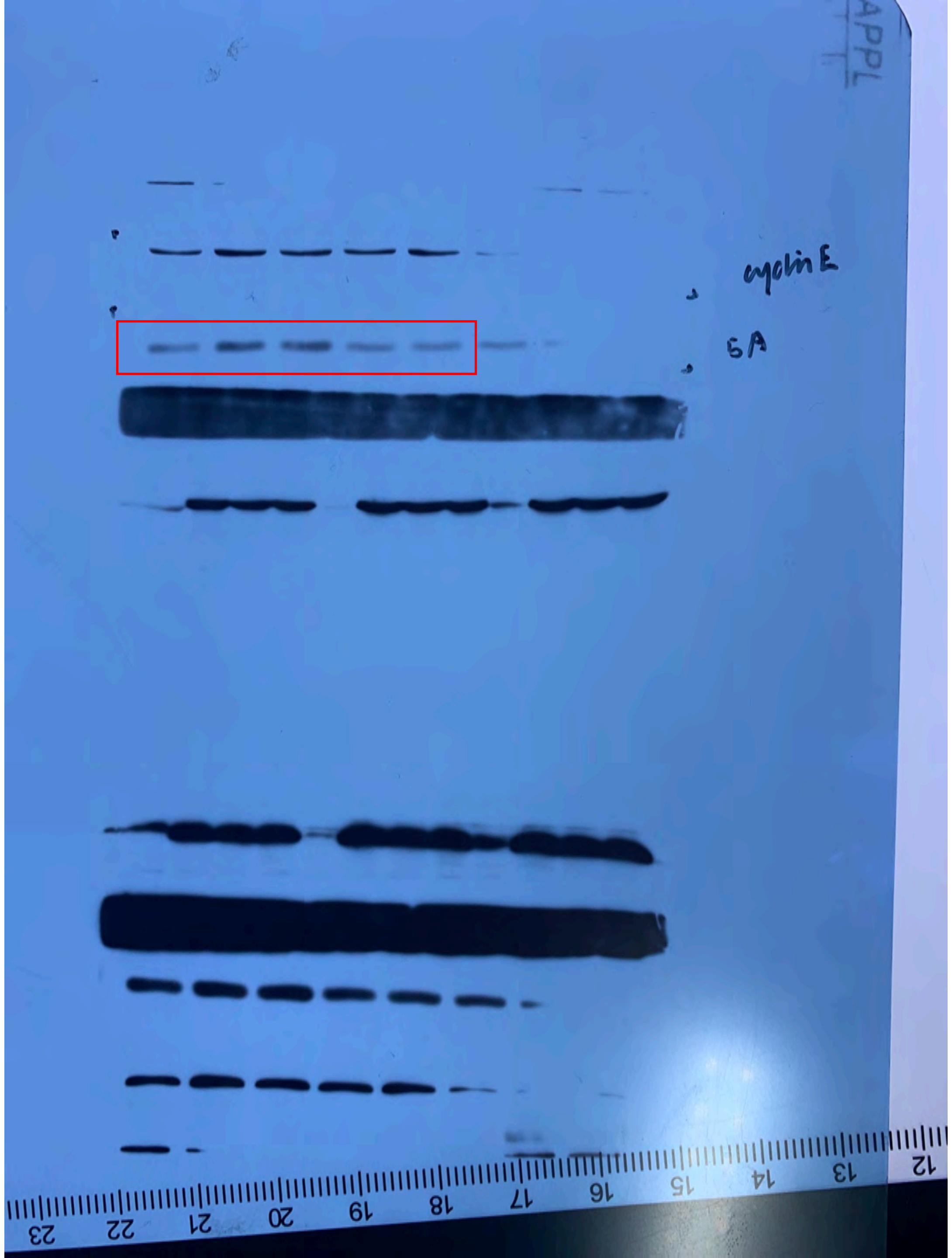

Fig. 4g

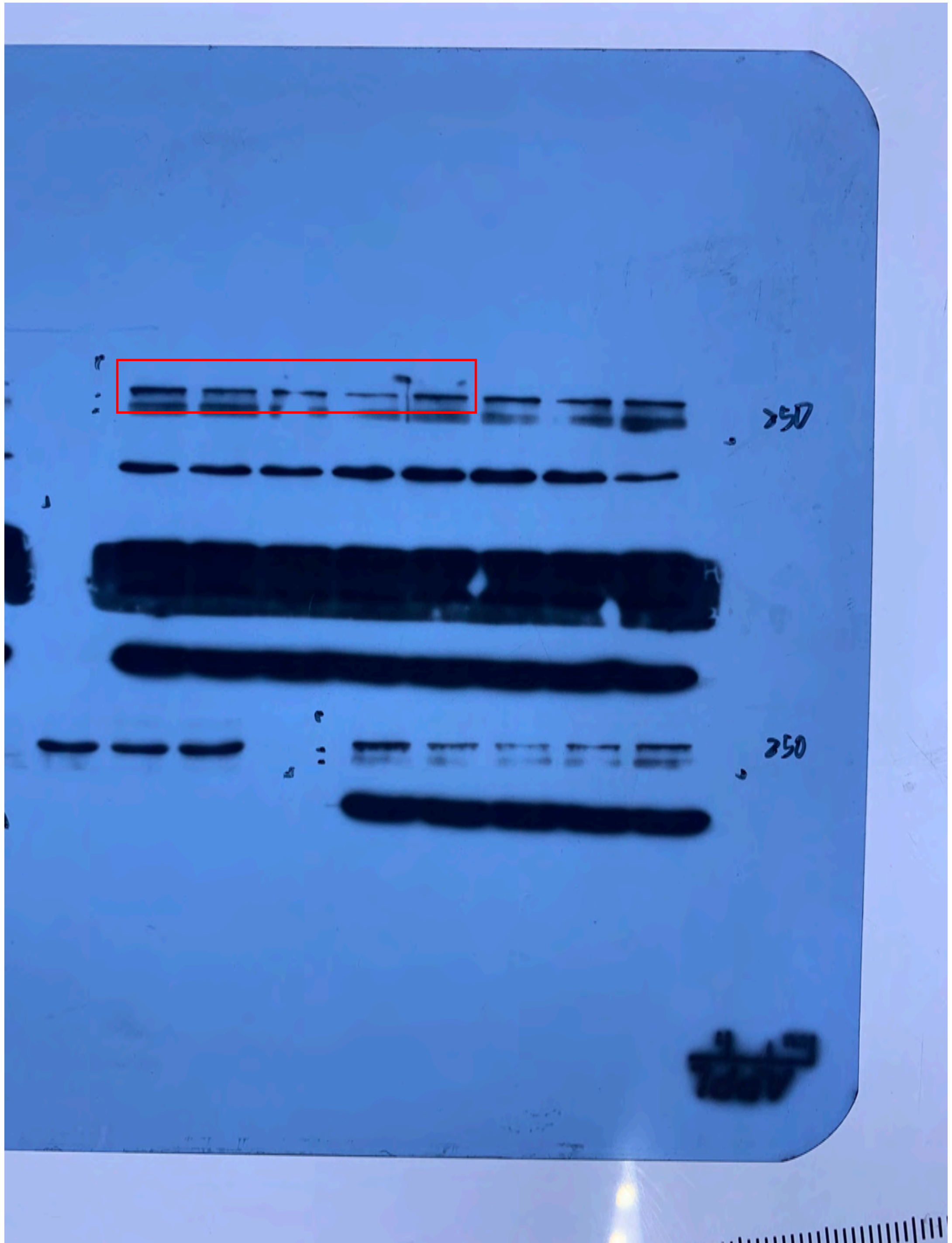

Fig. 4g

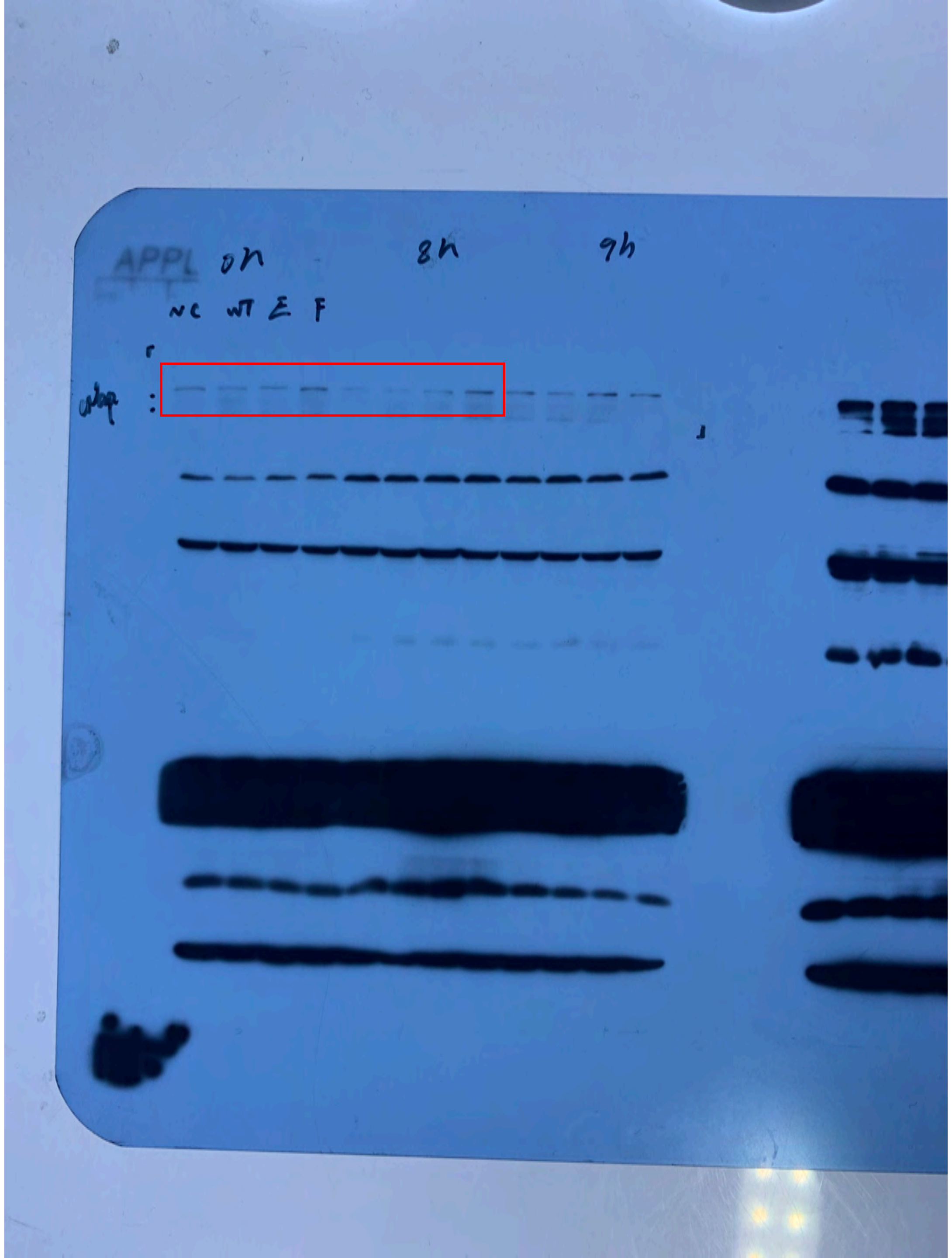

Fig. 4h

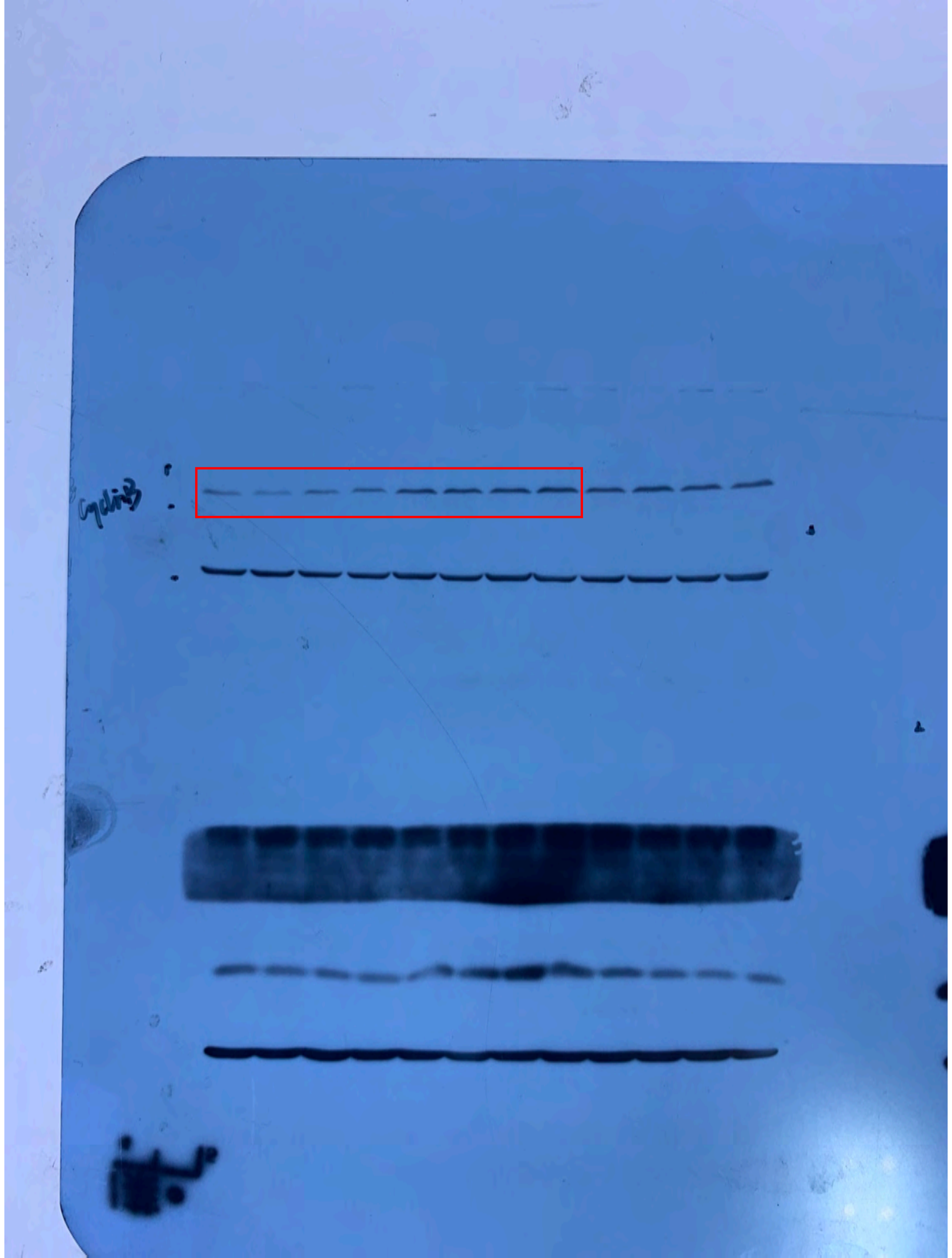

Fig. 4h

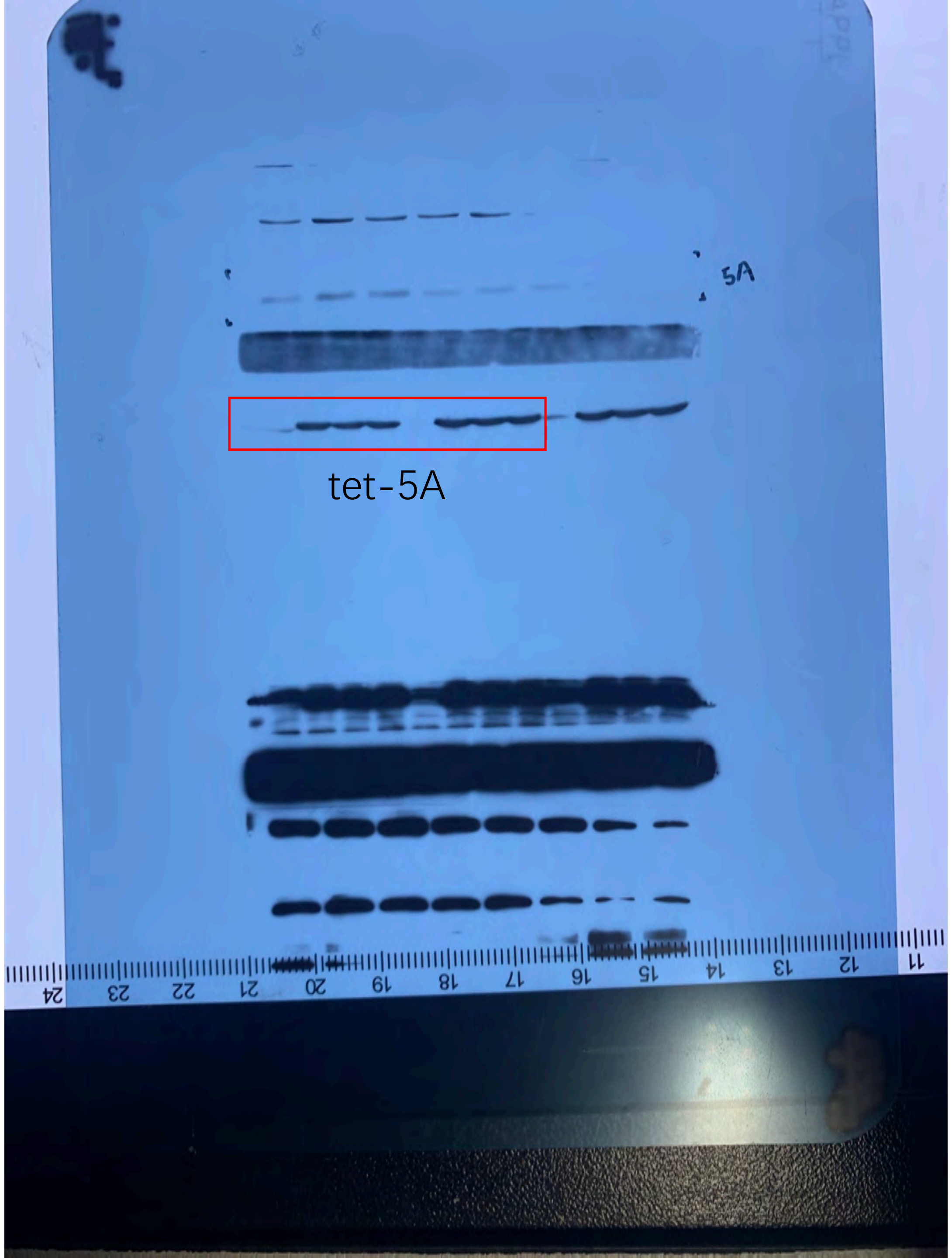

Fig. 4h

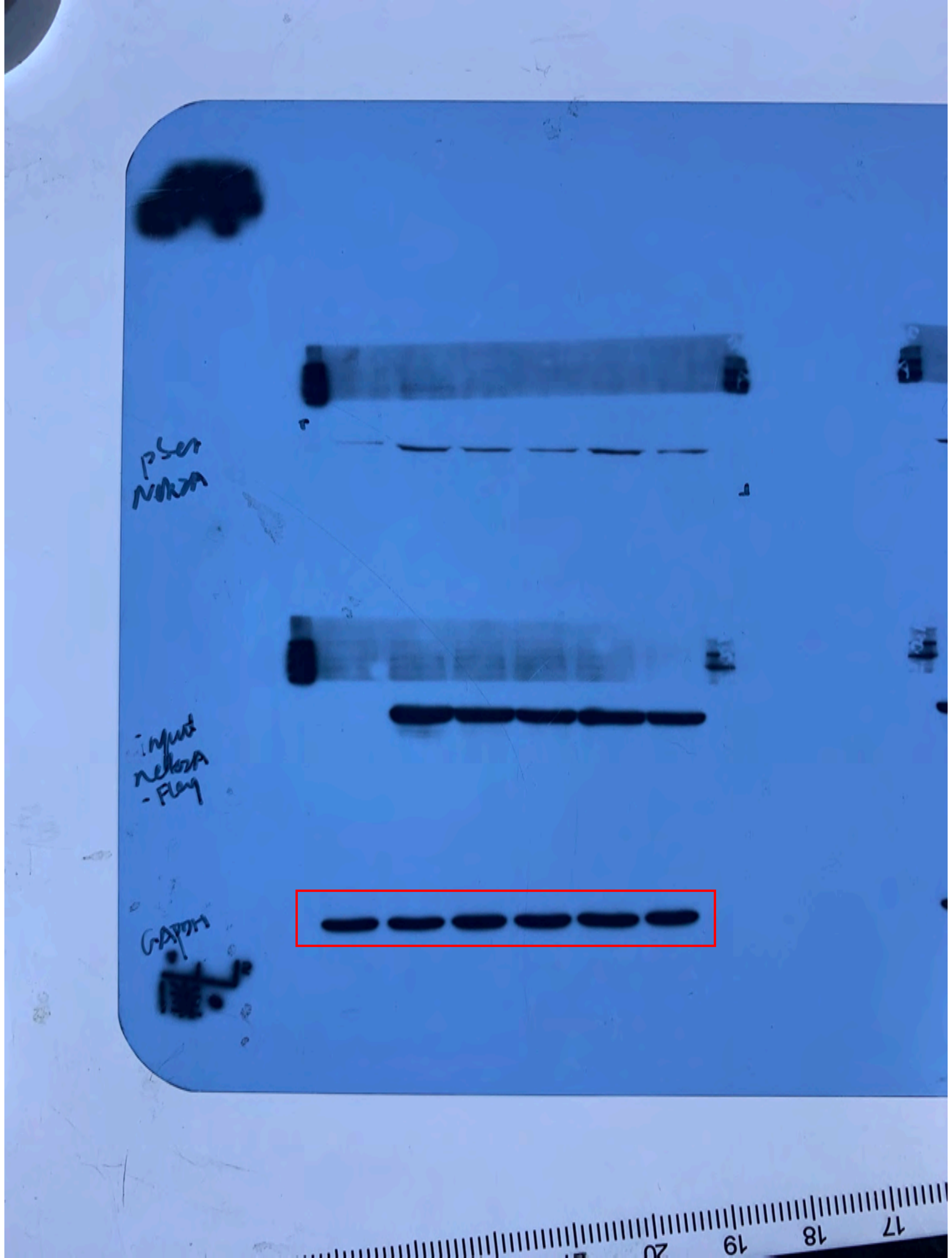

Fig. 4j

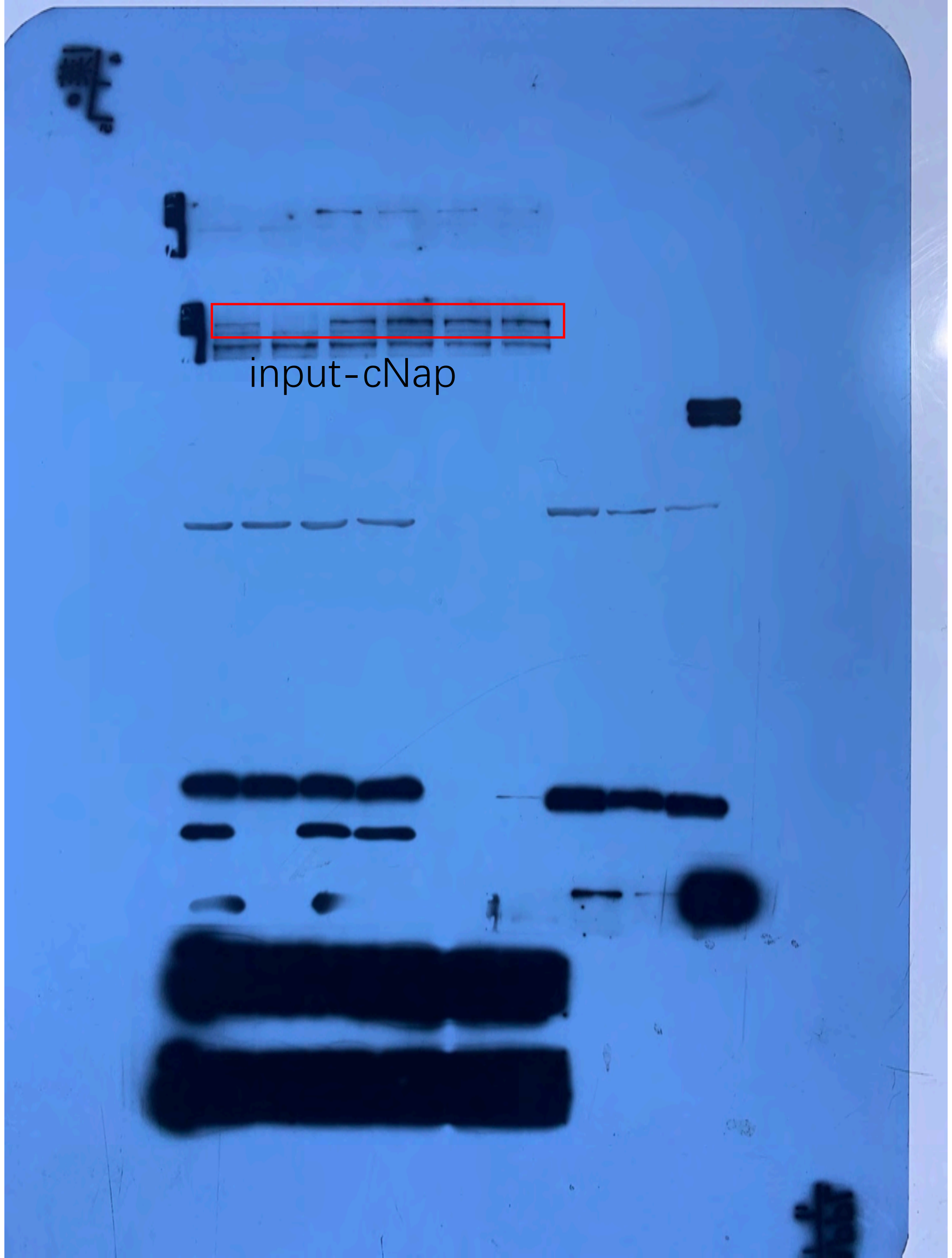

Fig. 4j

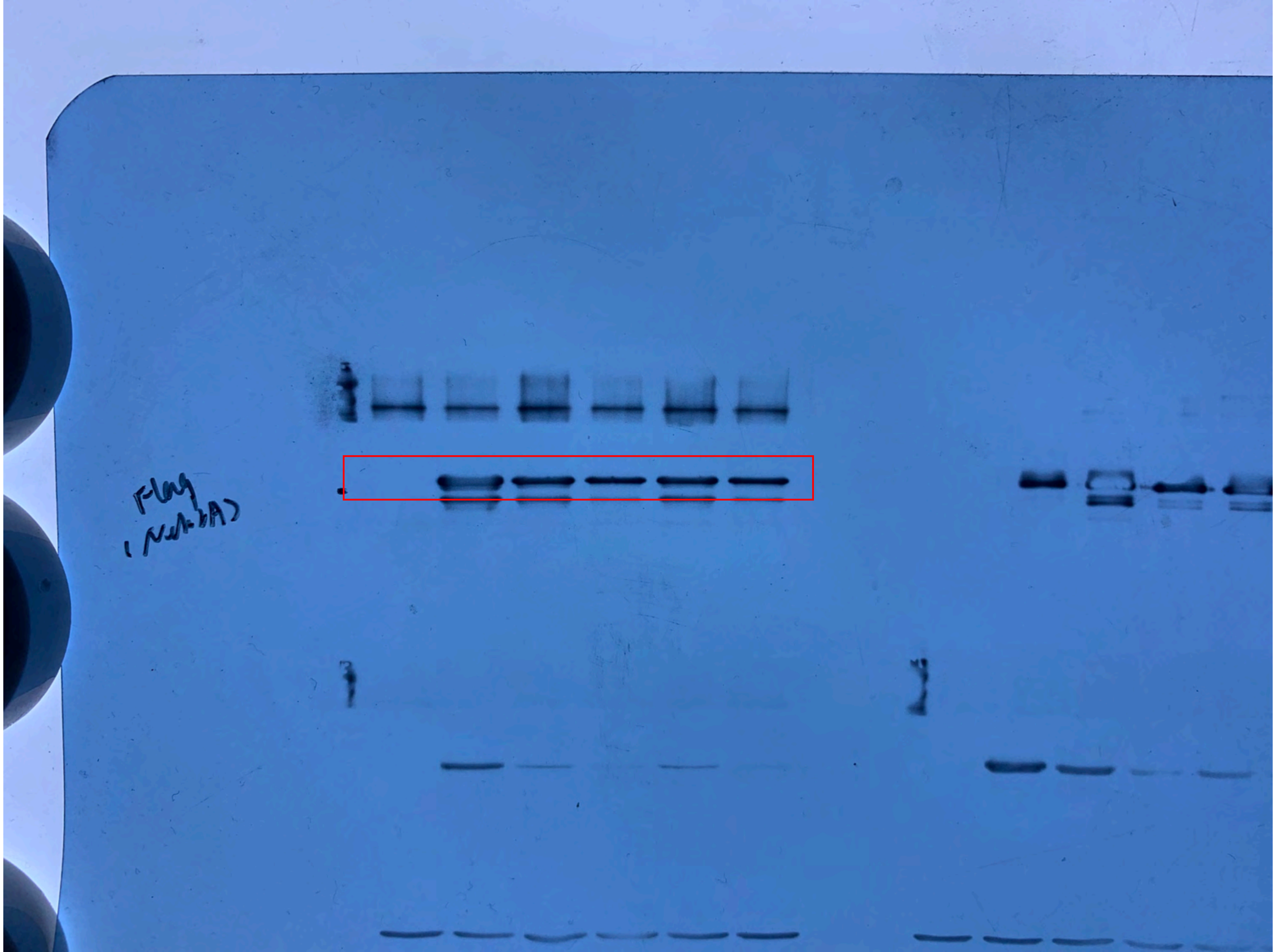

Fig. 4j

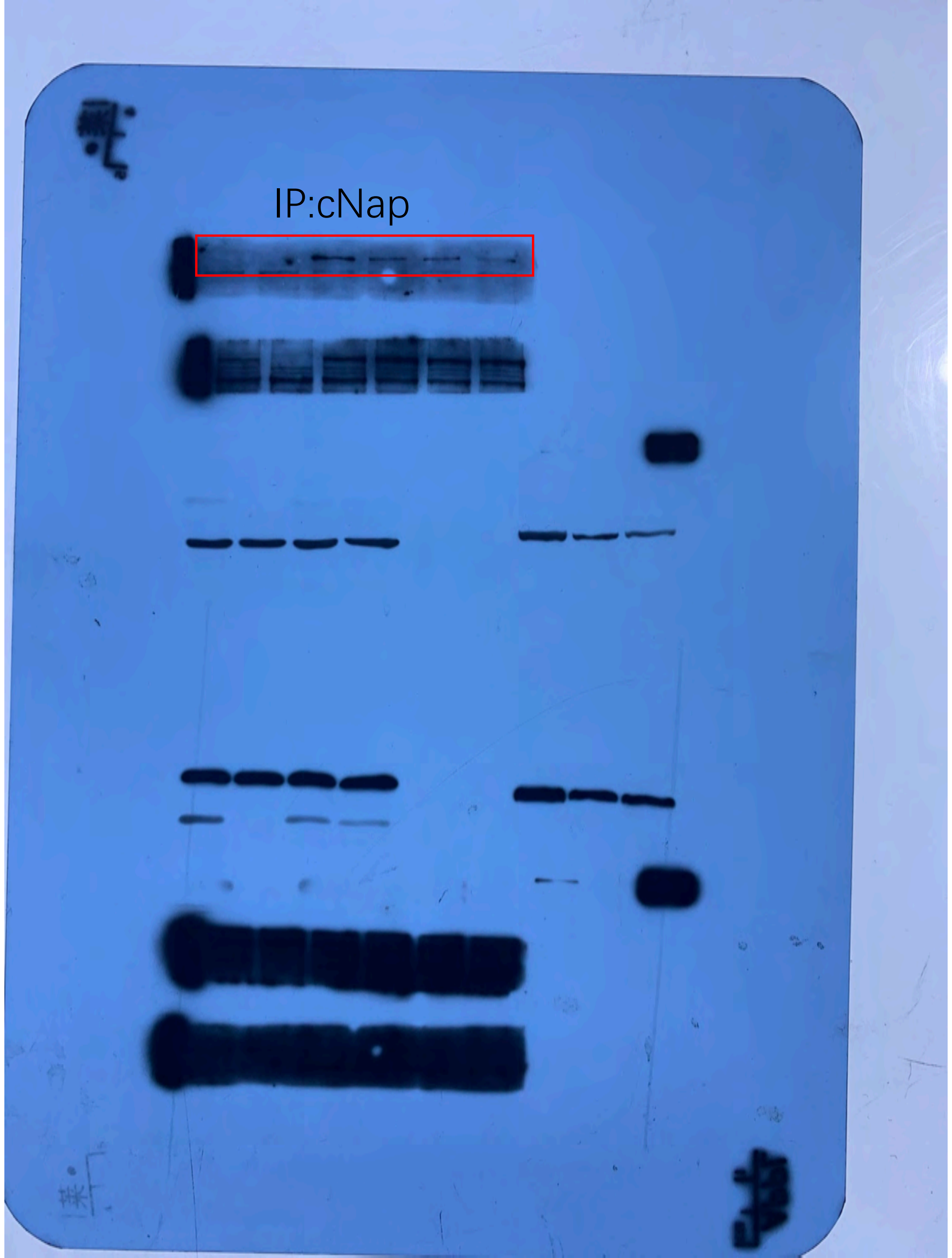

Fig. 4j

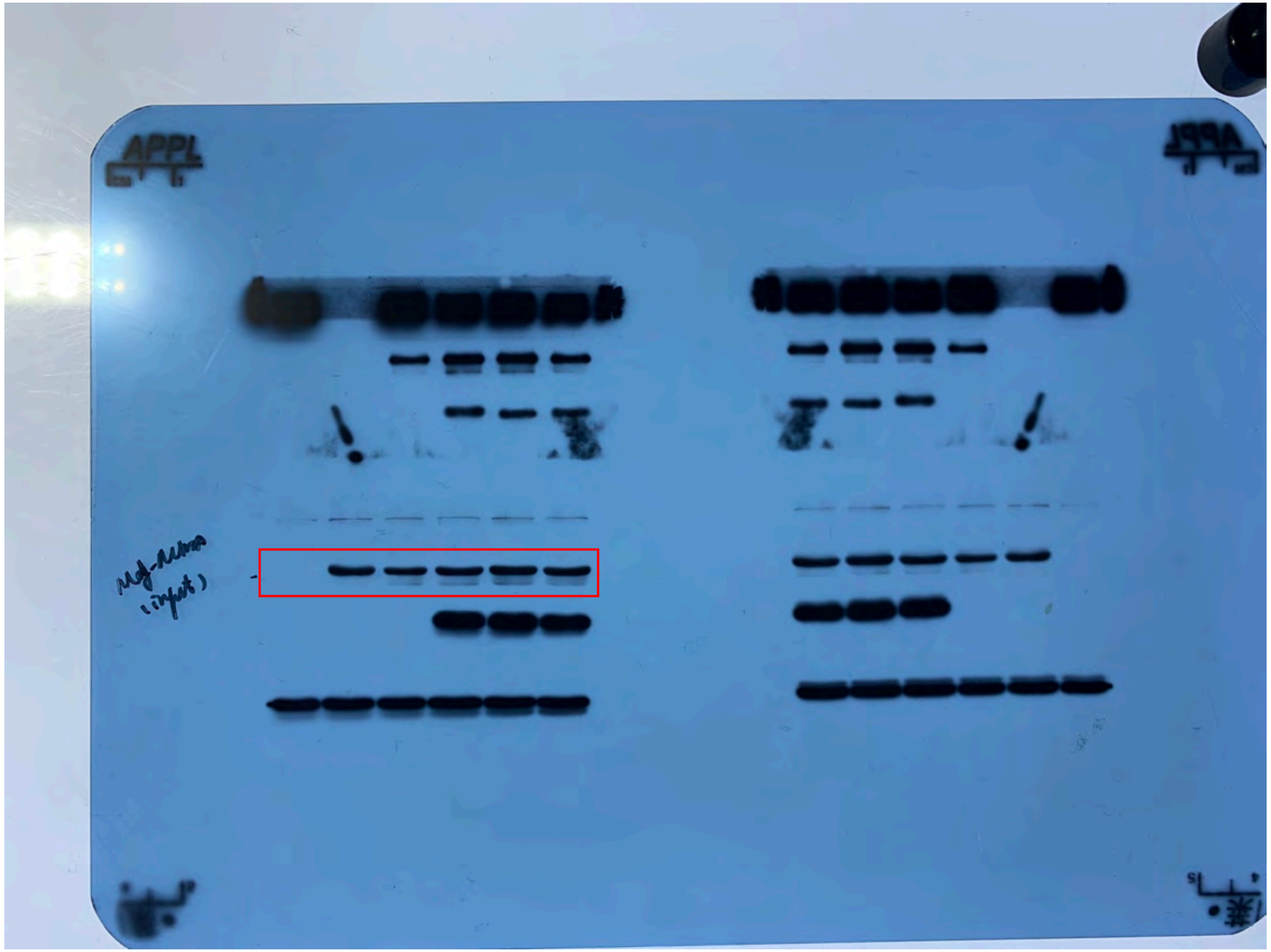

Fig. 4i

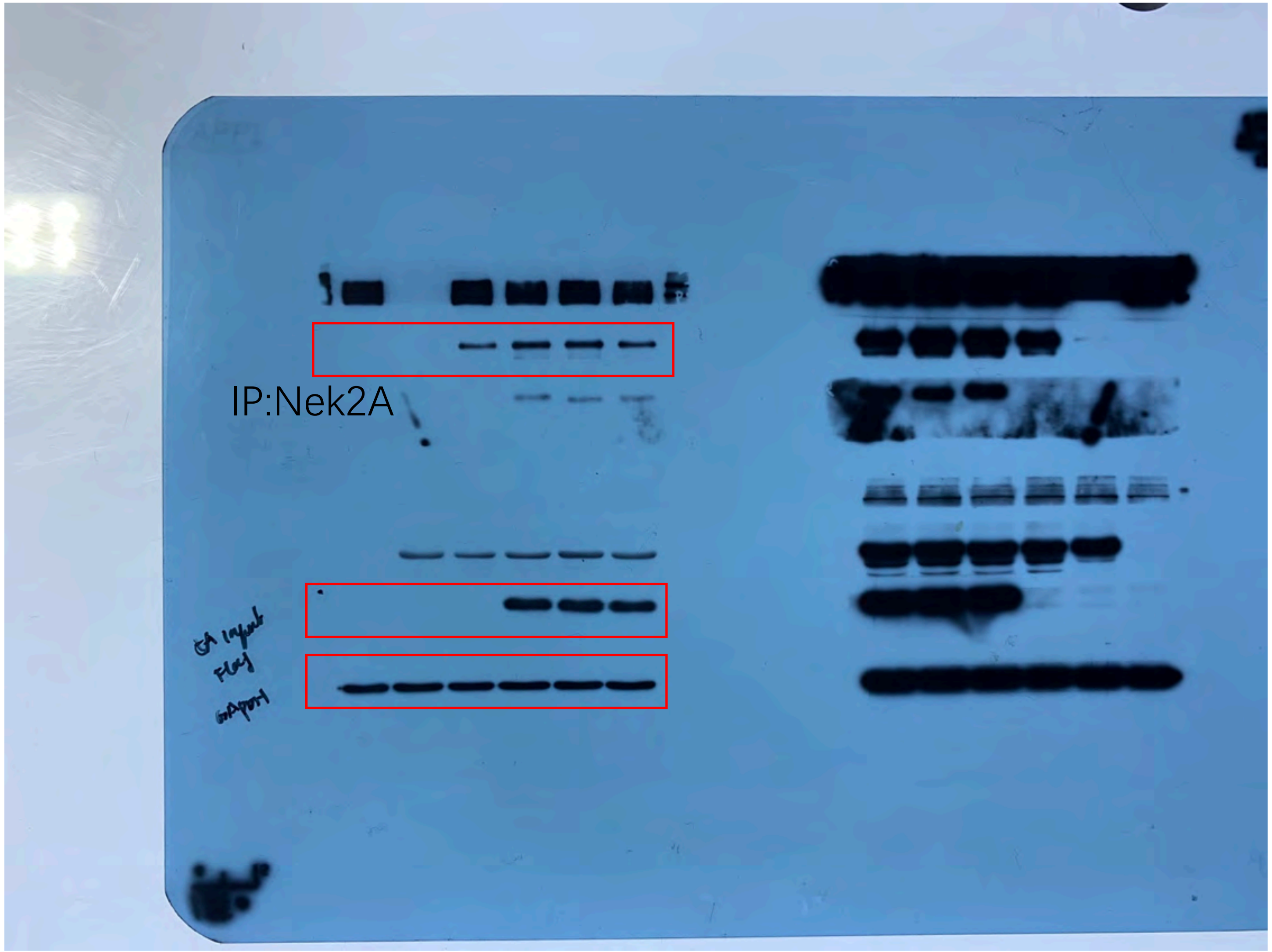

Fig. 4i

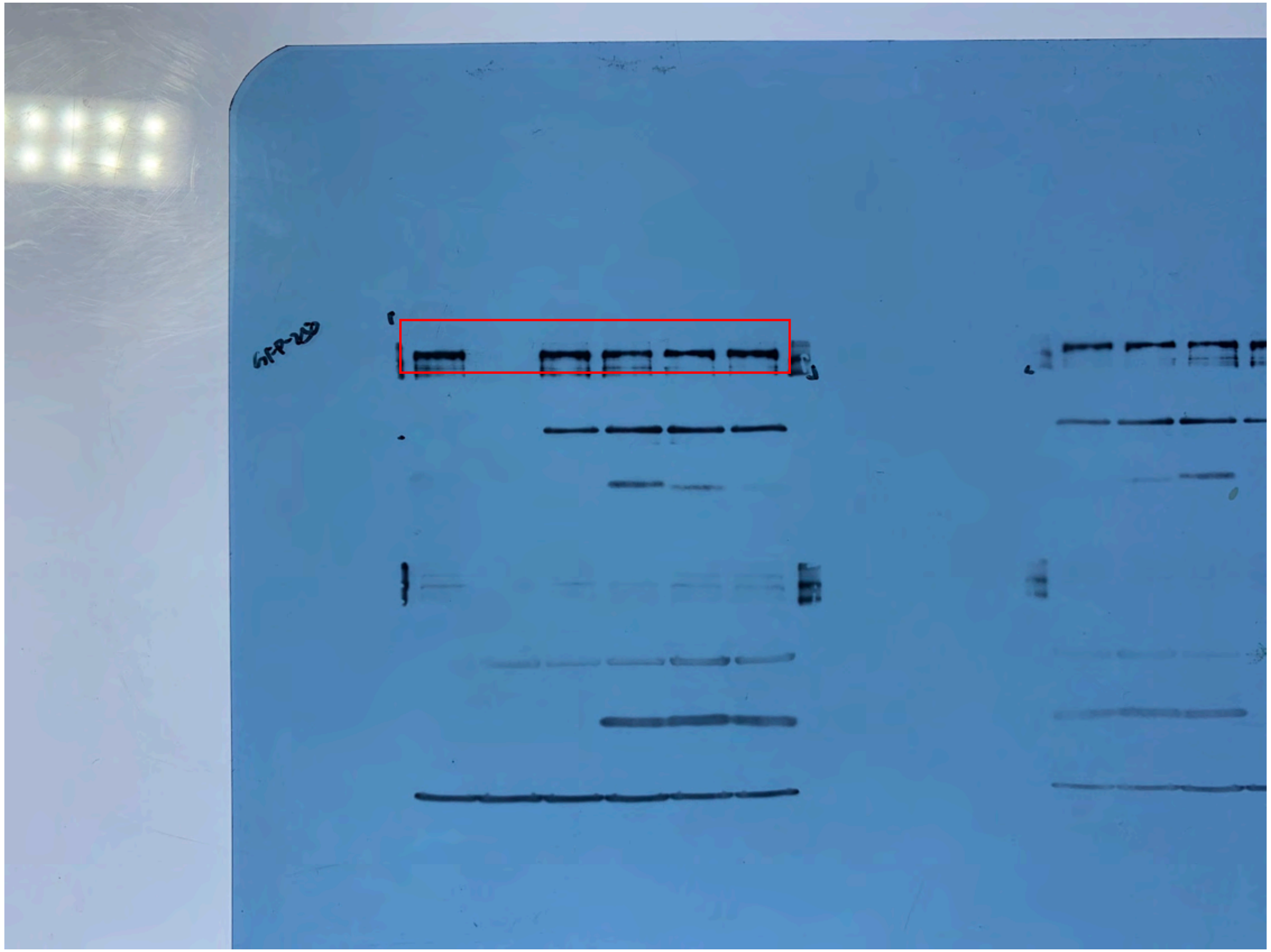

Fig. 4i

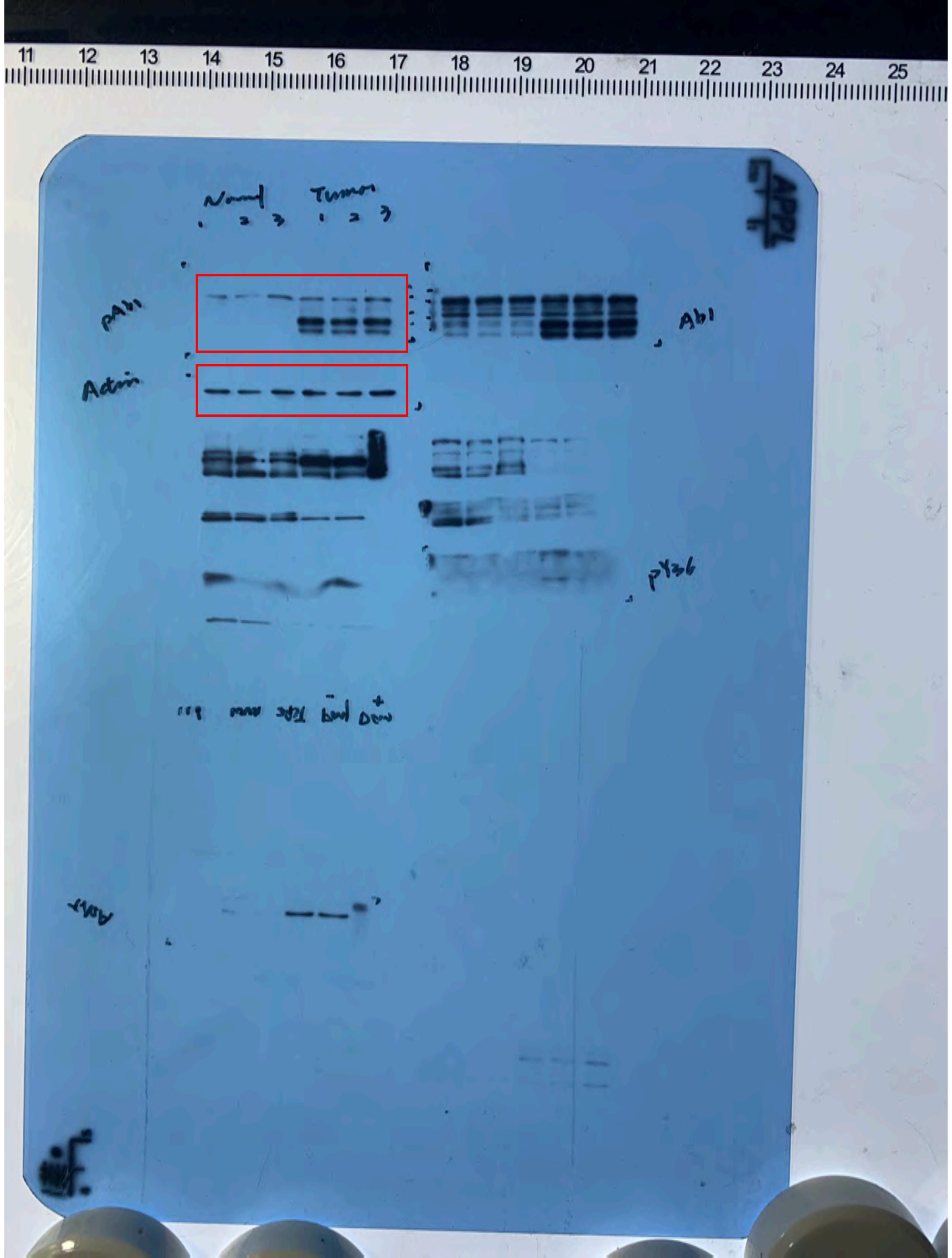

Fig. 6f

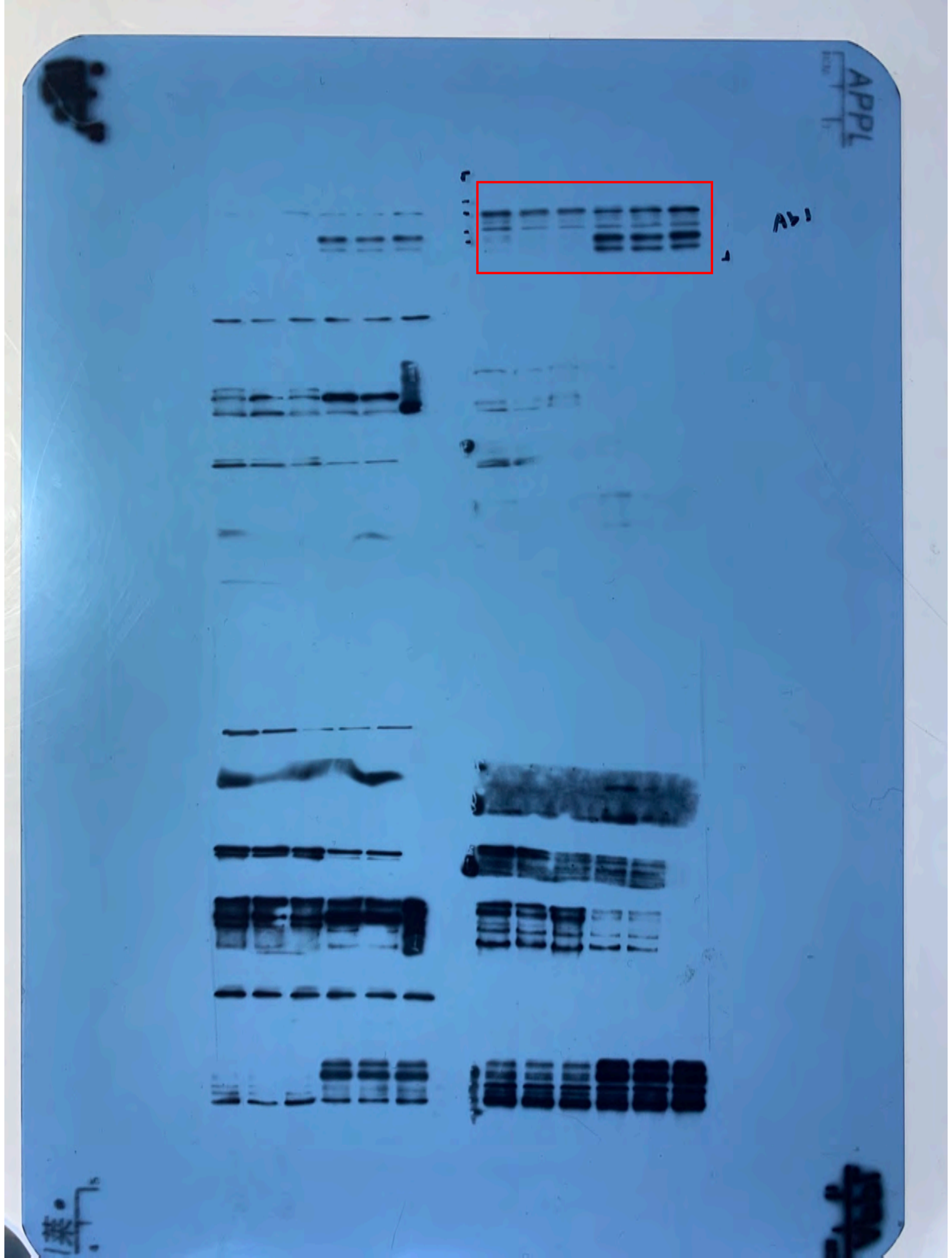

Fig. 6f

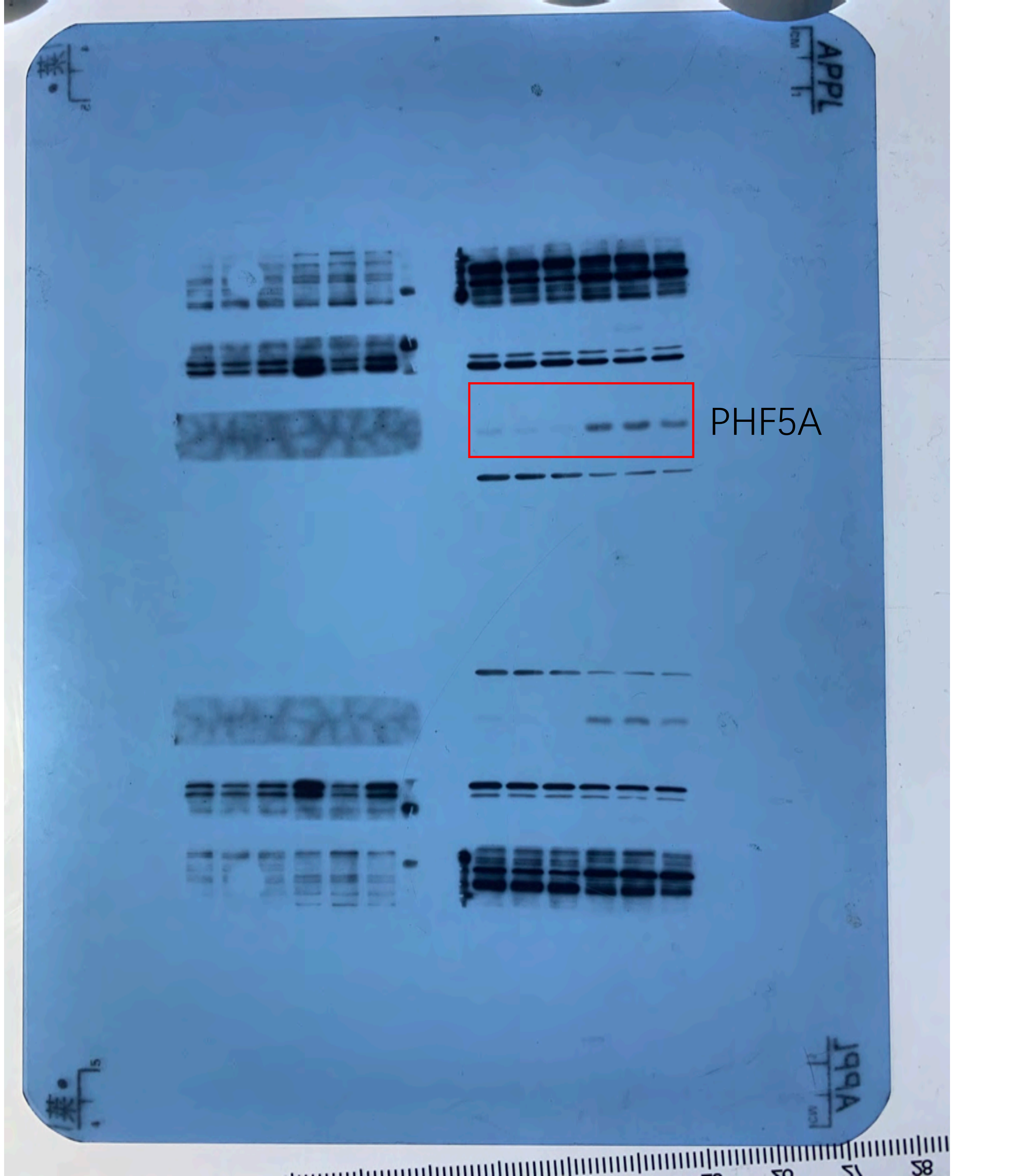

Fig. 6f

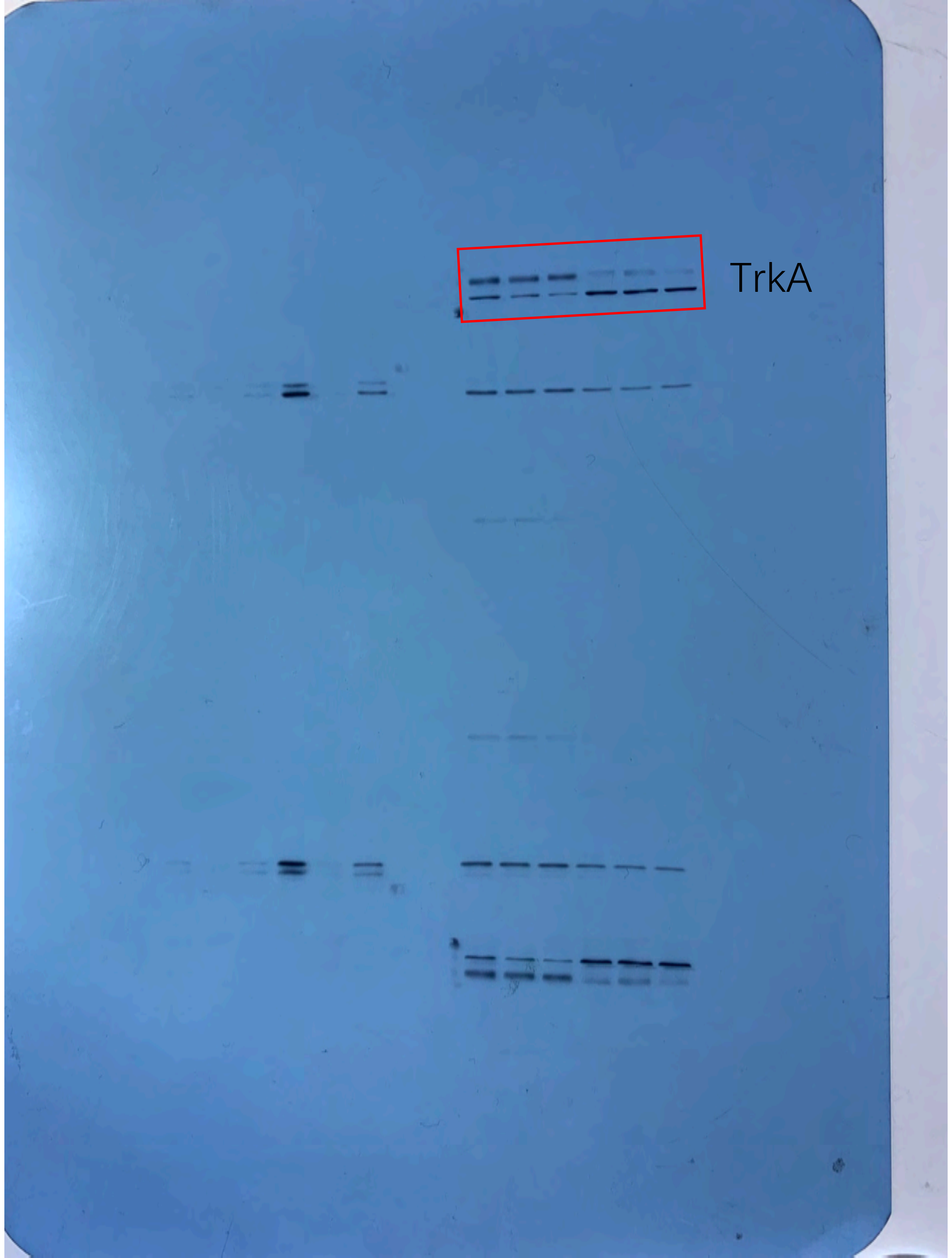

Fig. 6f

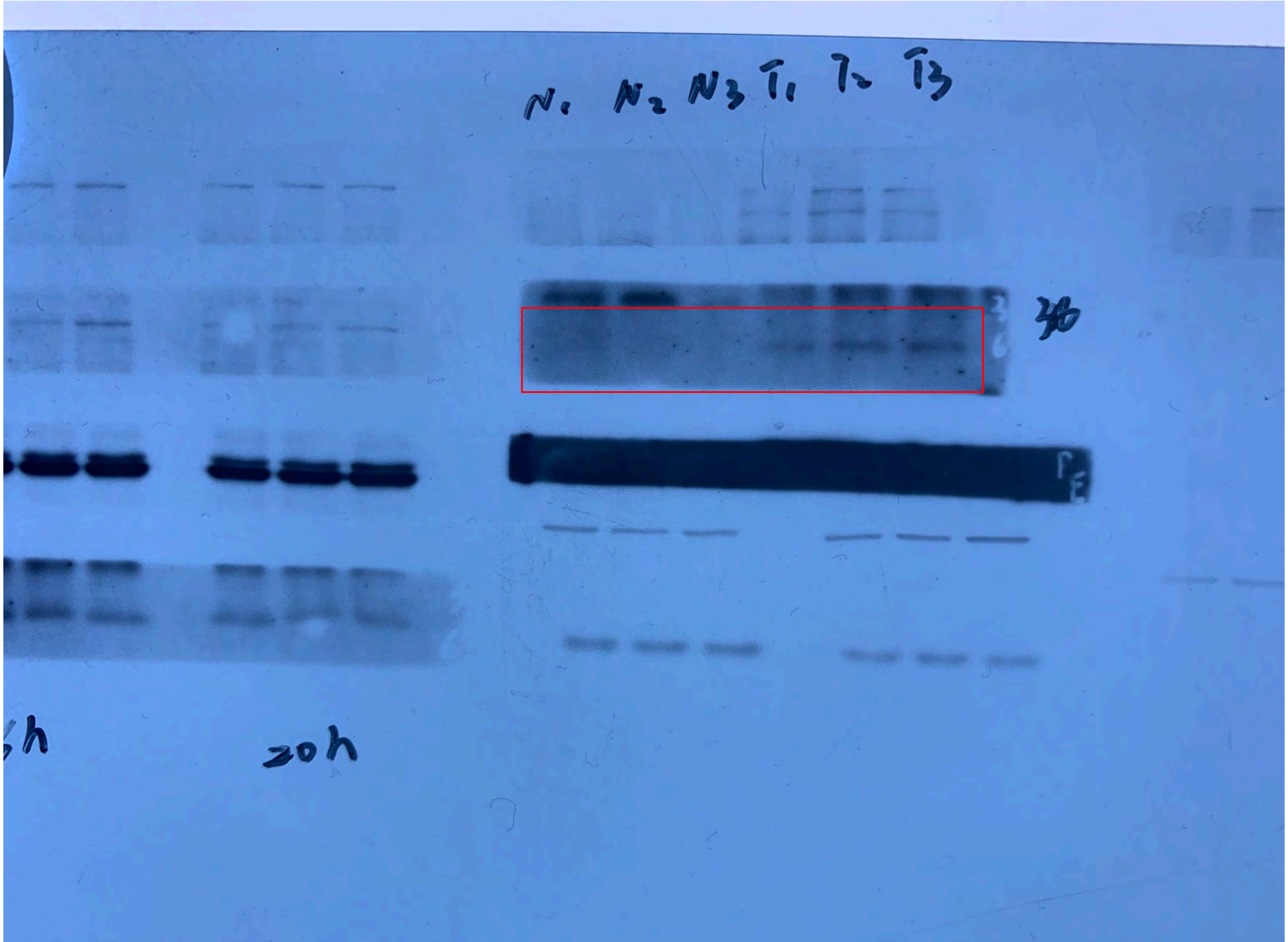

Fig. 6f

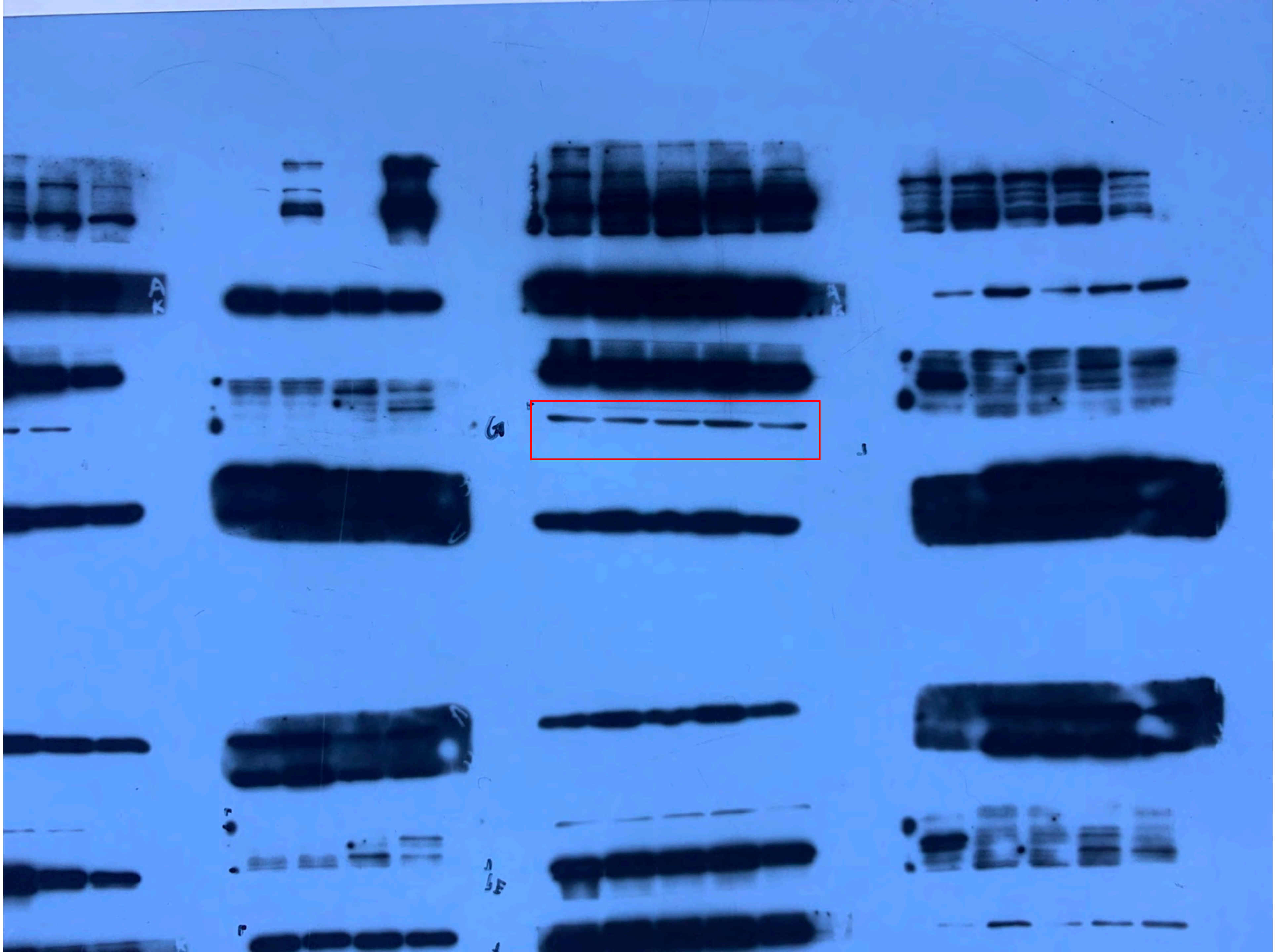

Fig. 6d

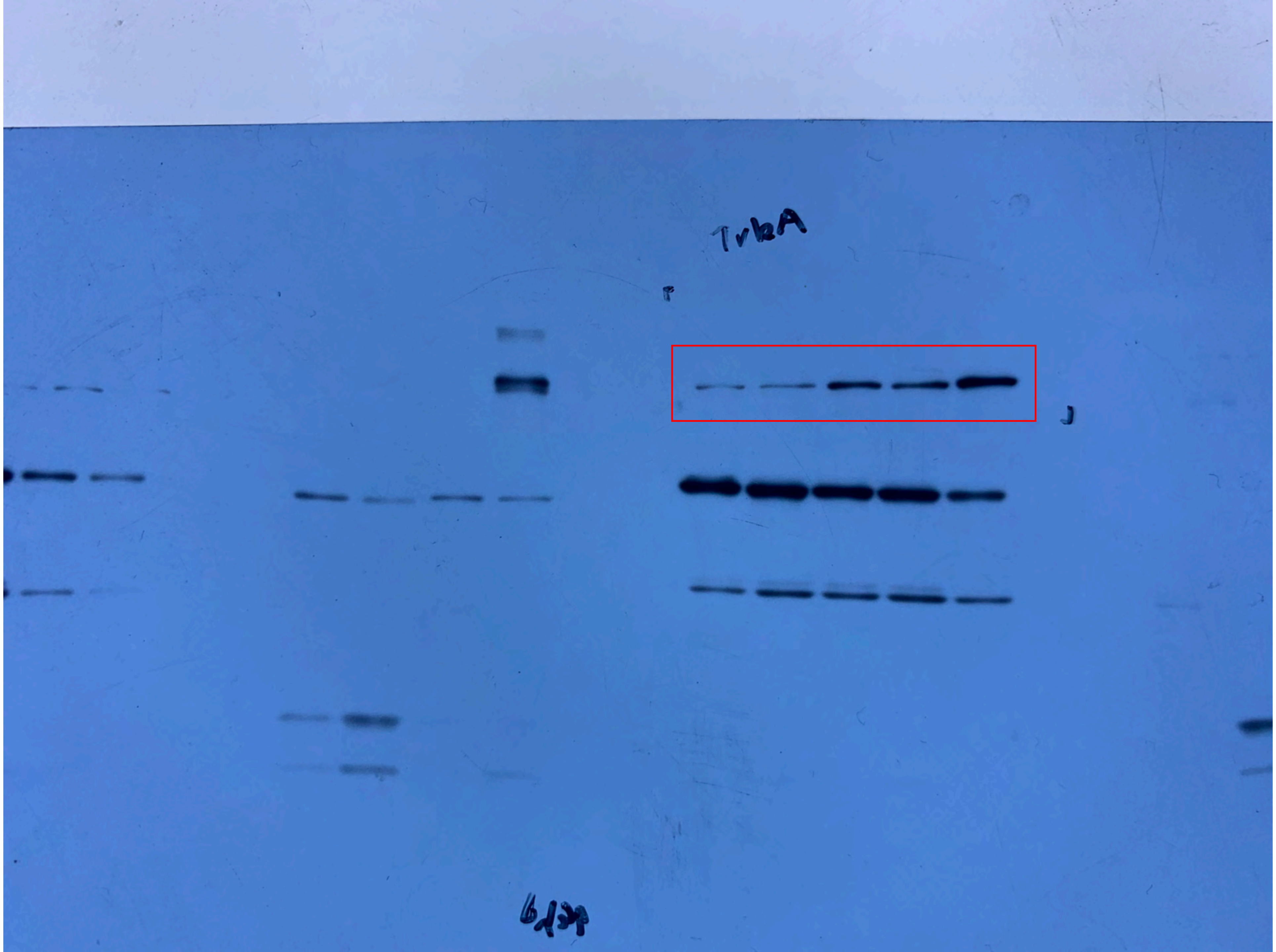

Fig. 6d

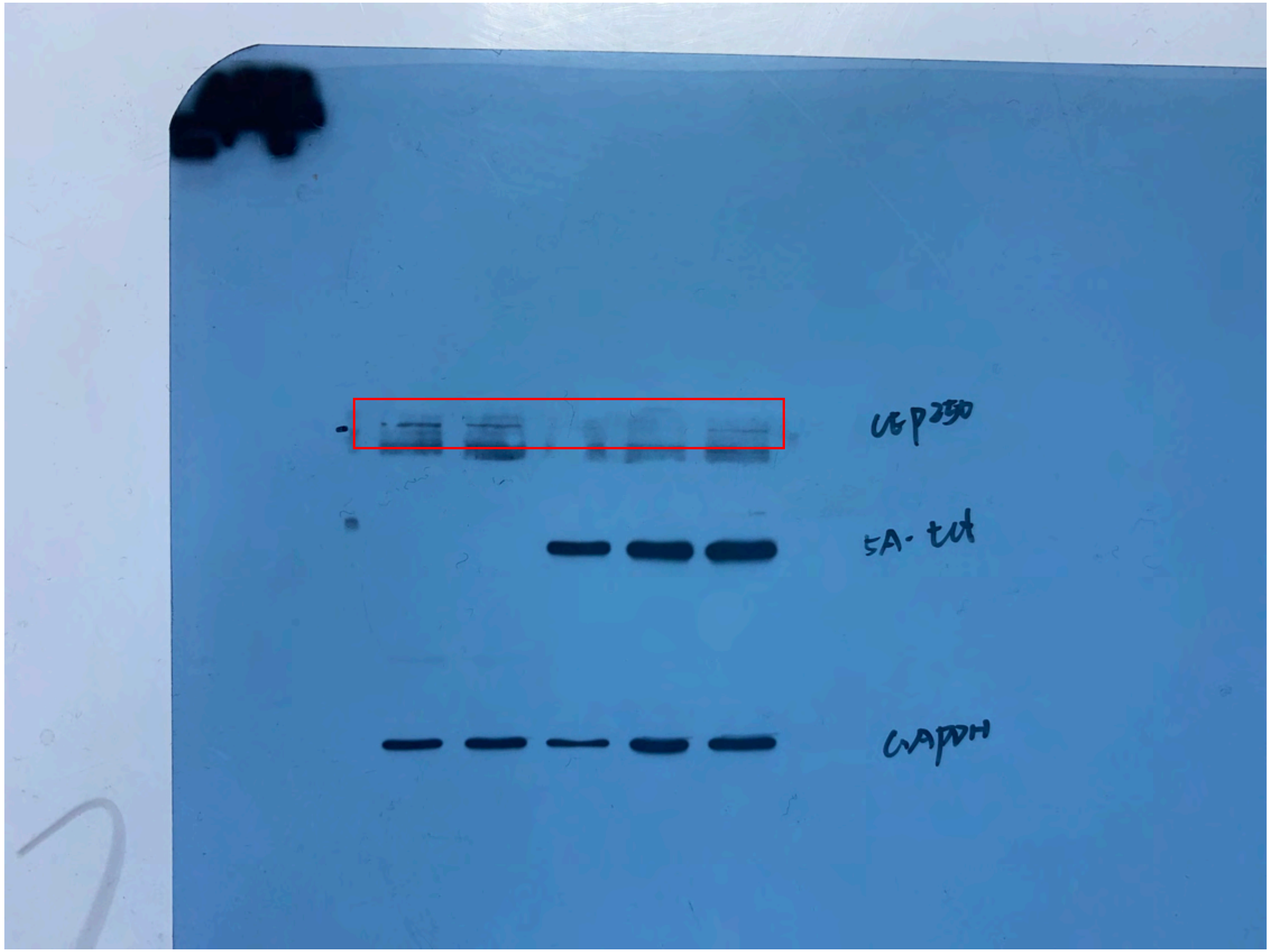

Fig. 7b

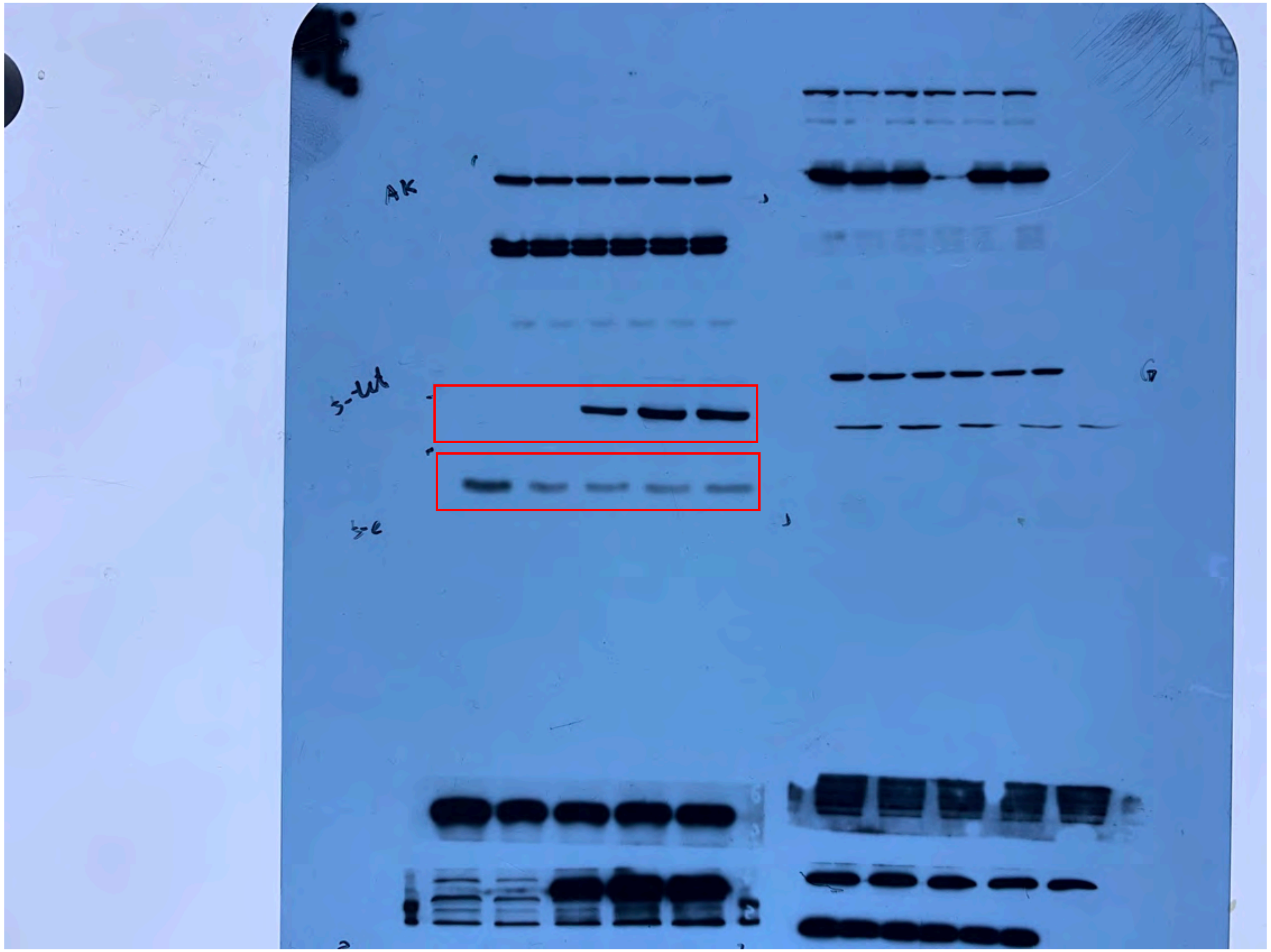

Fig. 7b

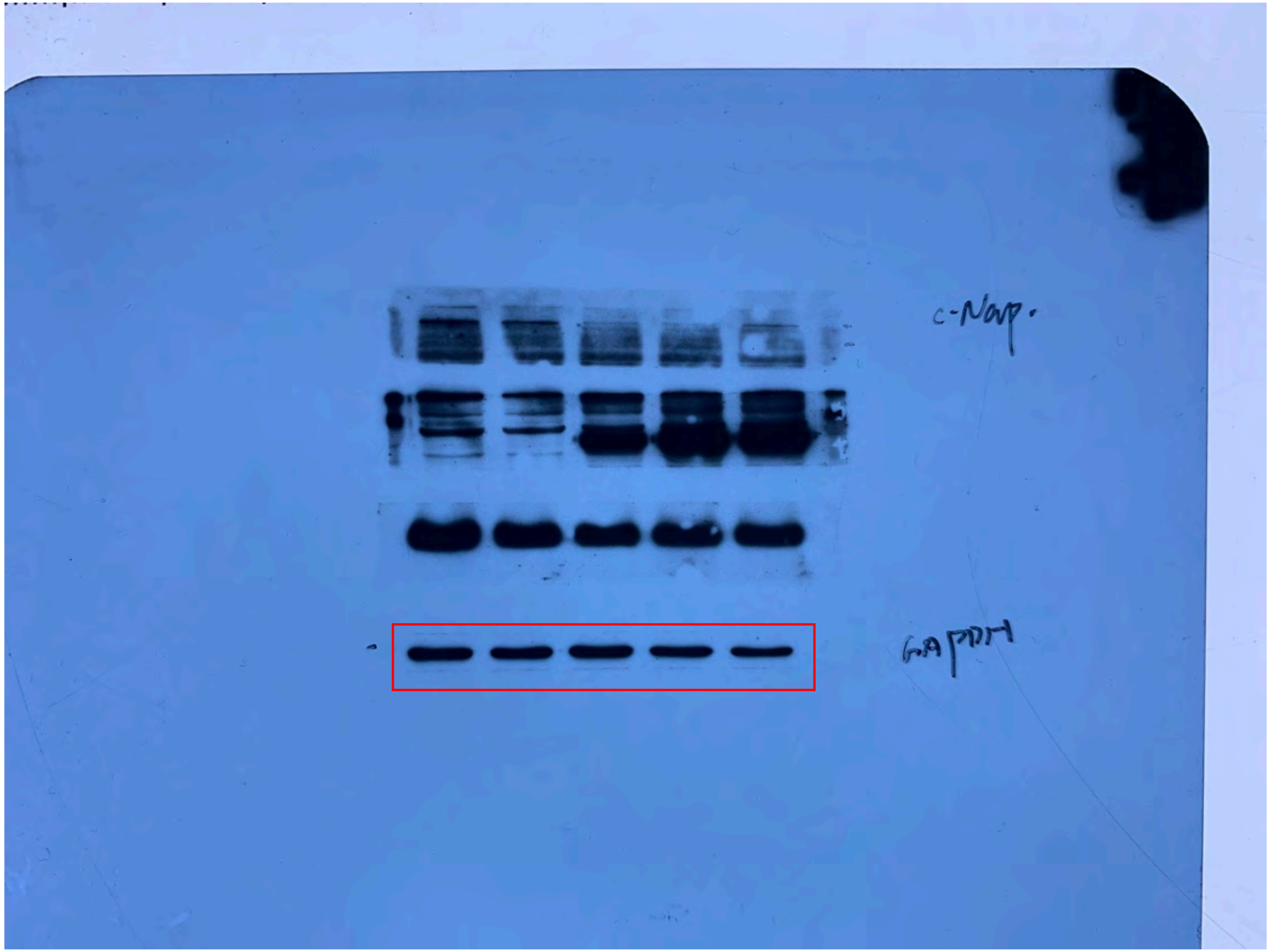

Fig. 7b

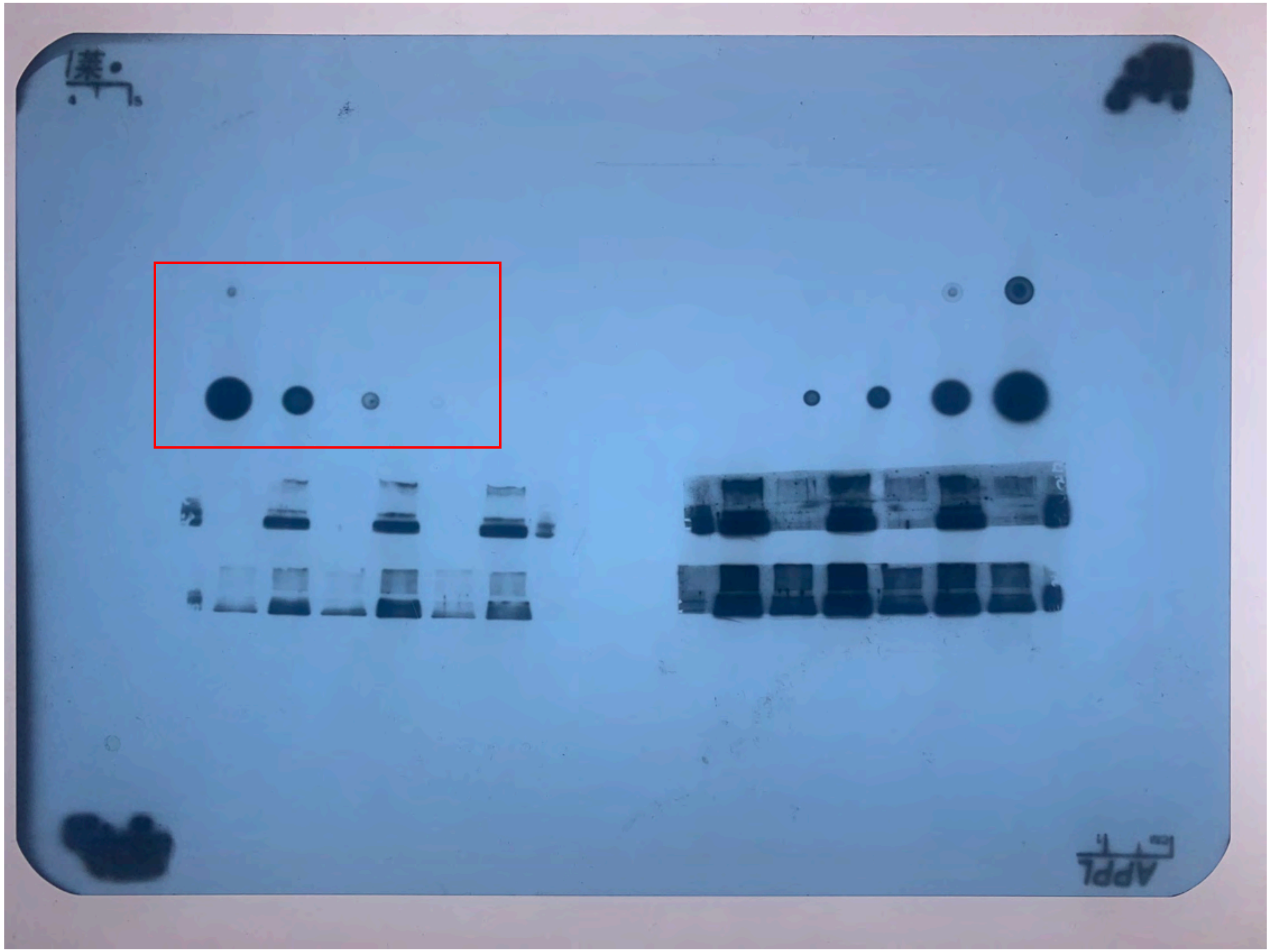

Fig. S1b

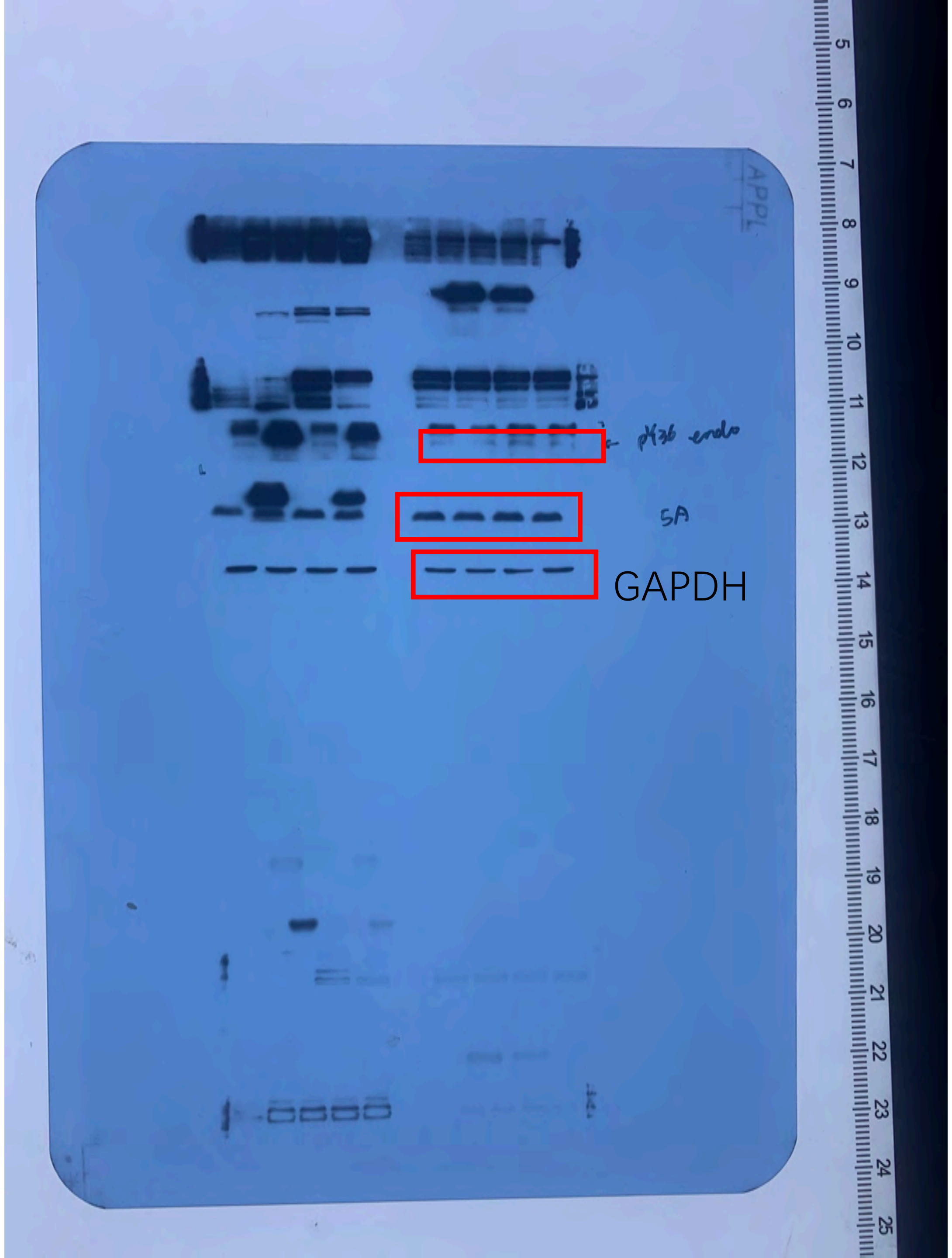

Fig. S1c

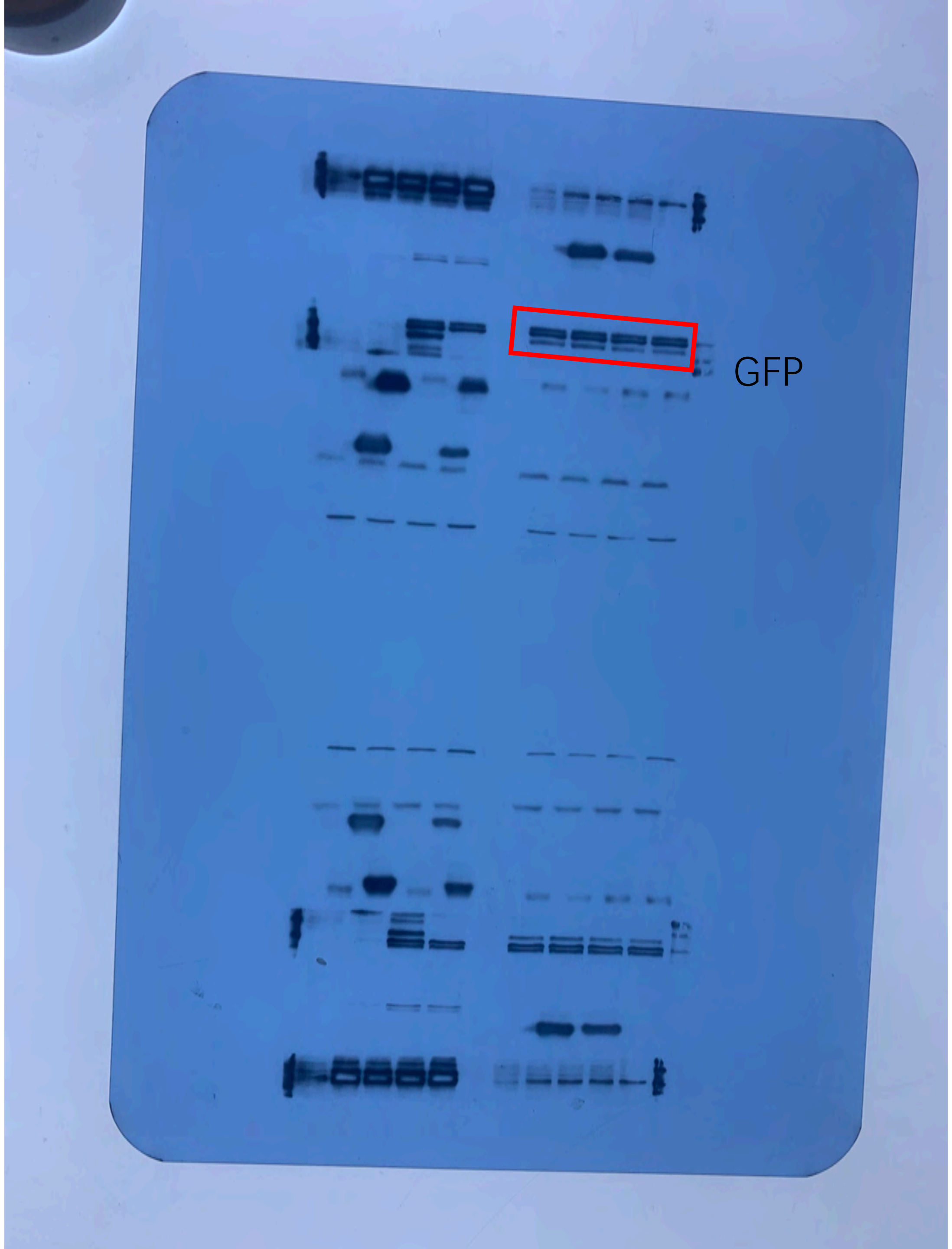

Fig. S1c

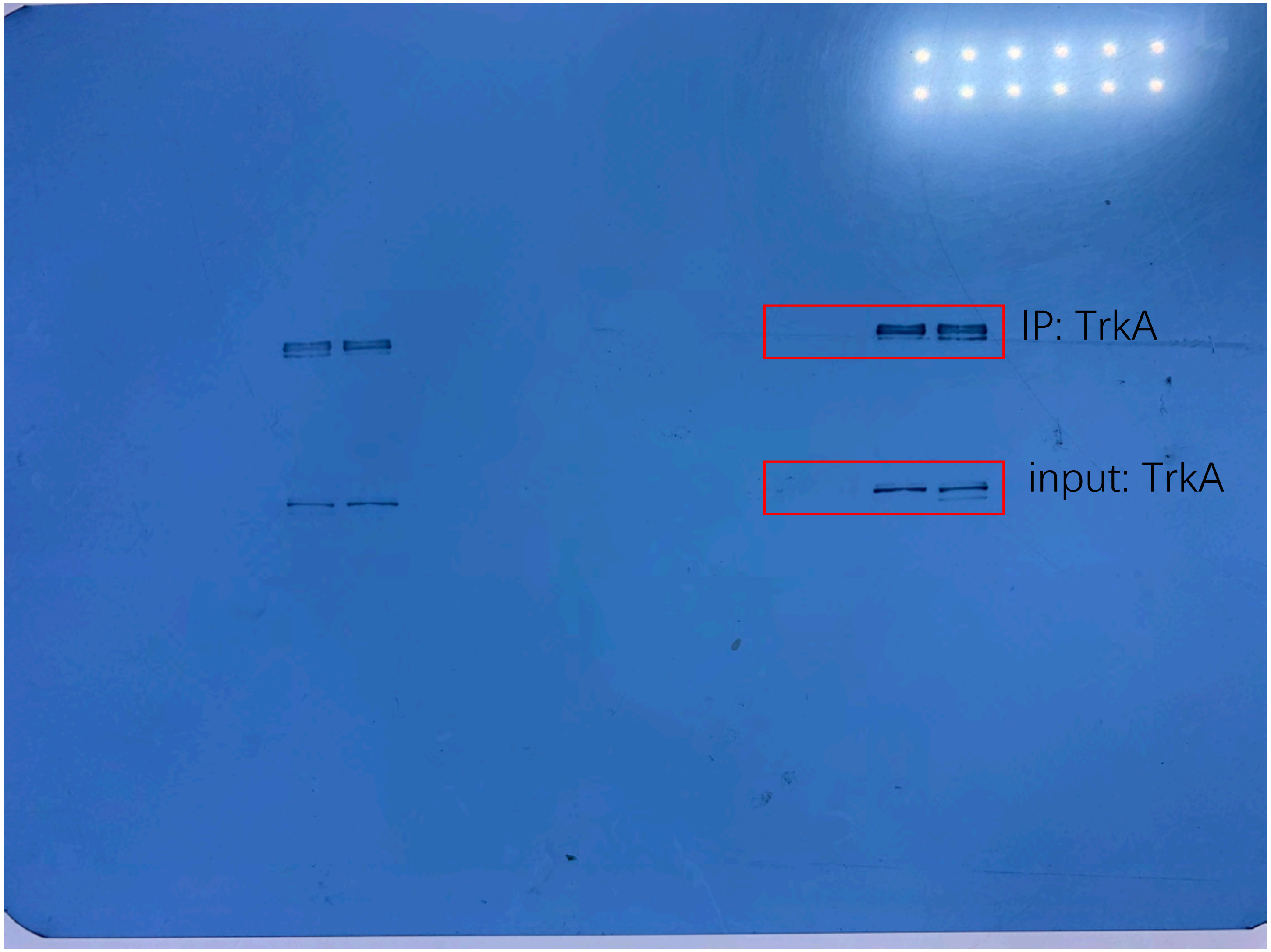

Fig. S1d

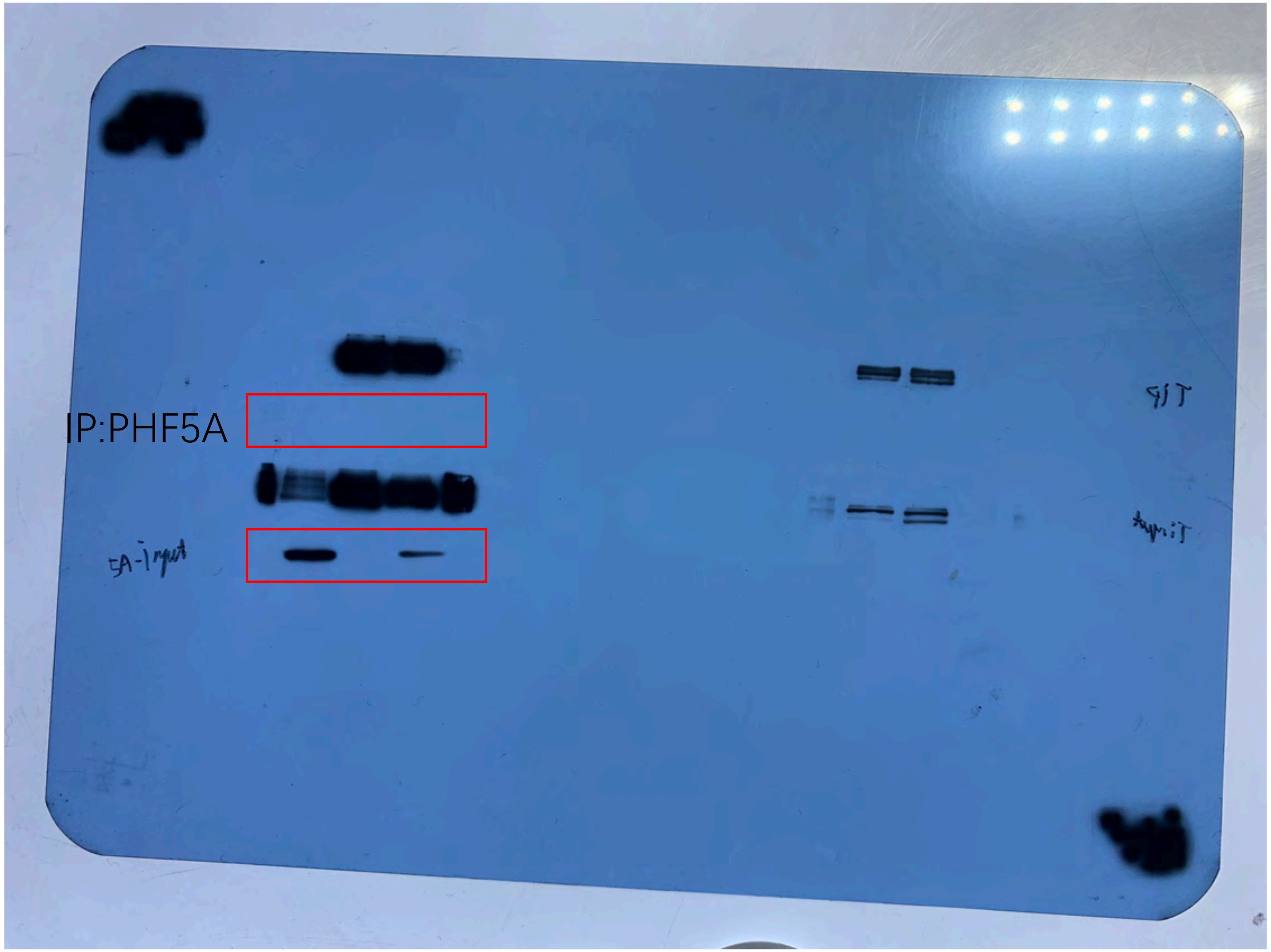

Fig. S1d

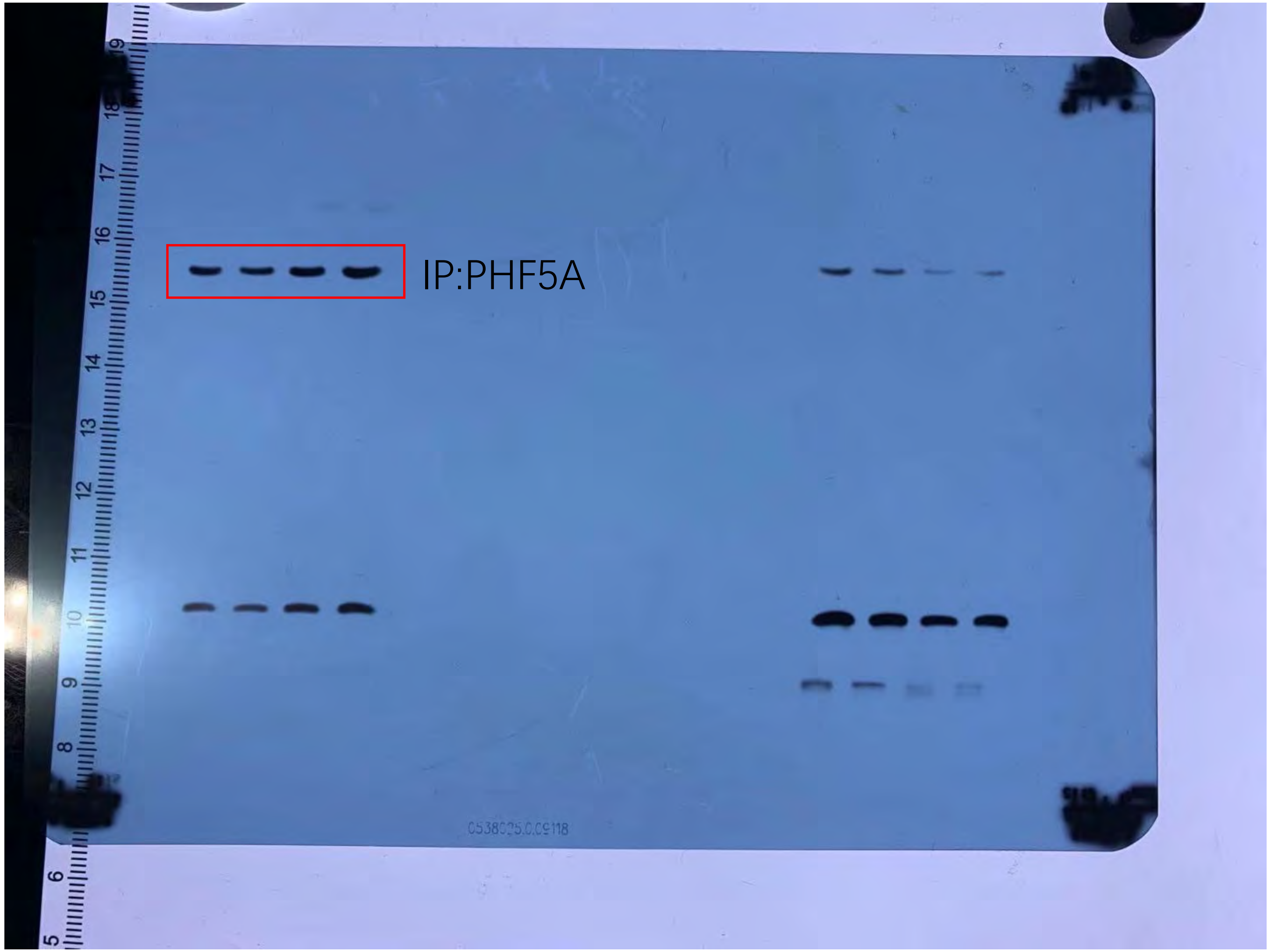

Fig. S2a

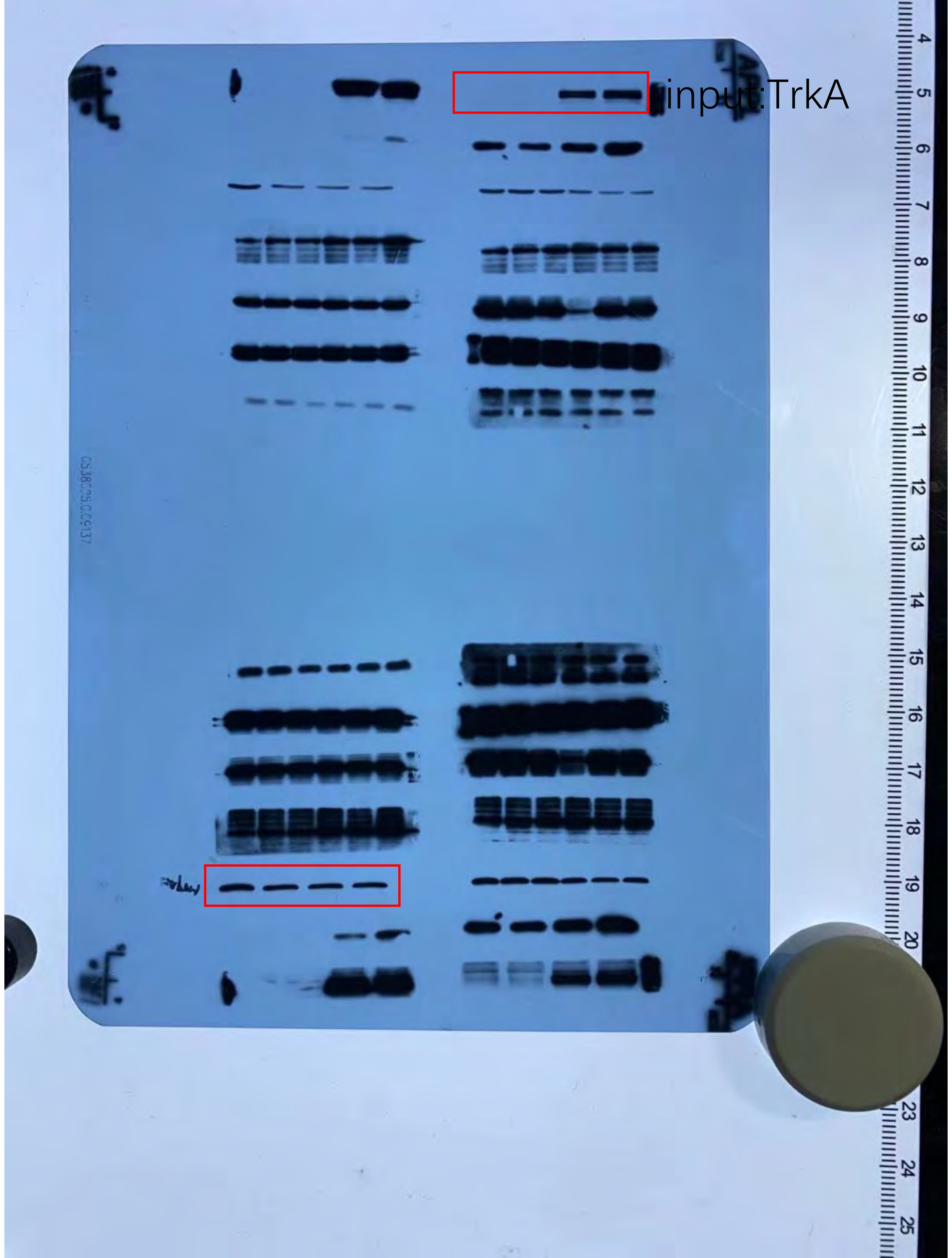

Fig. S2a

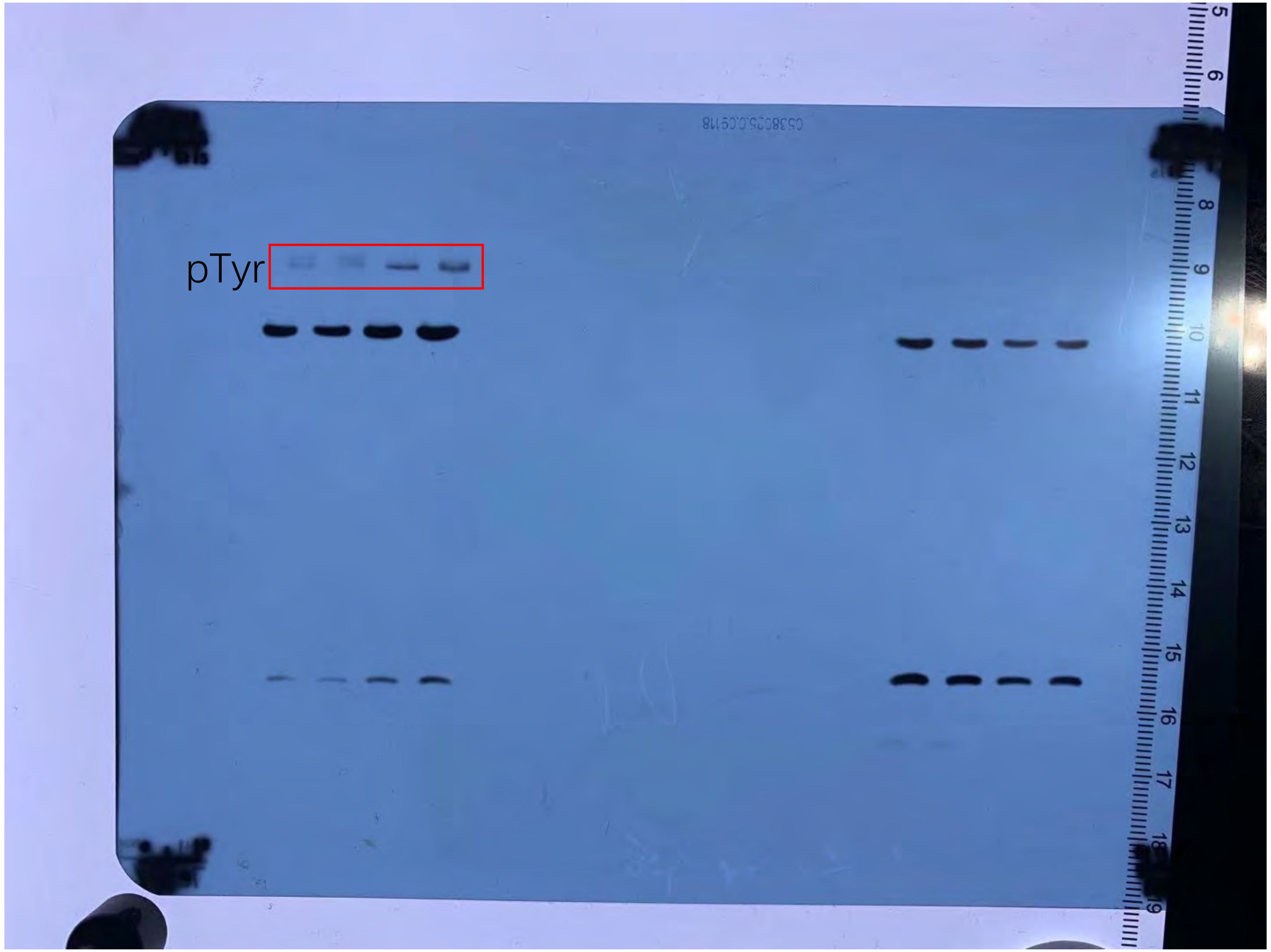

Fig. S2a

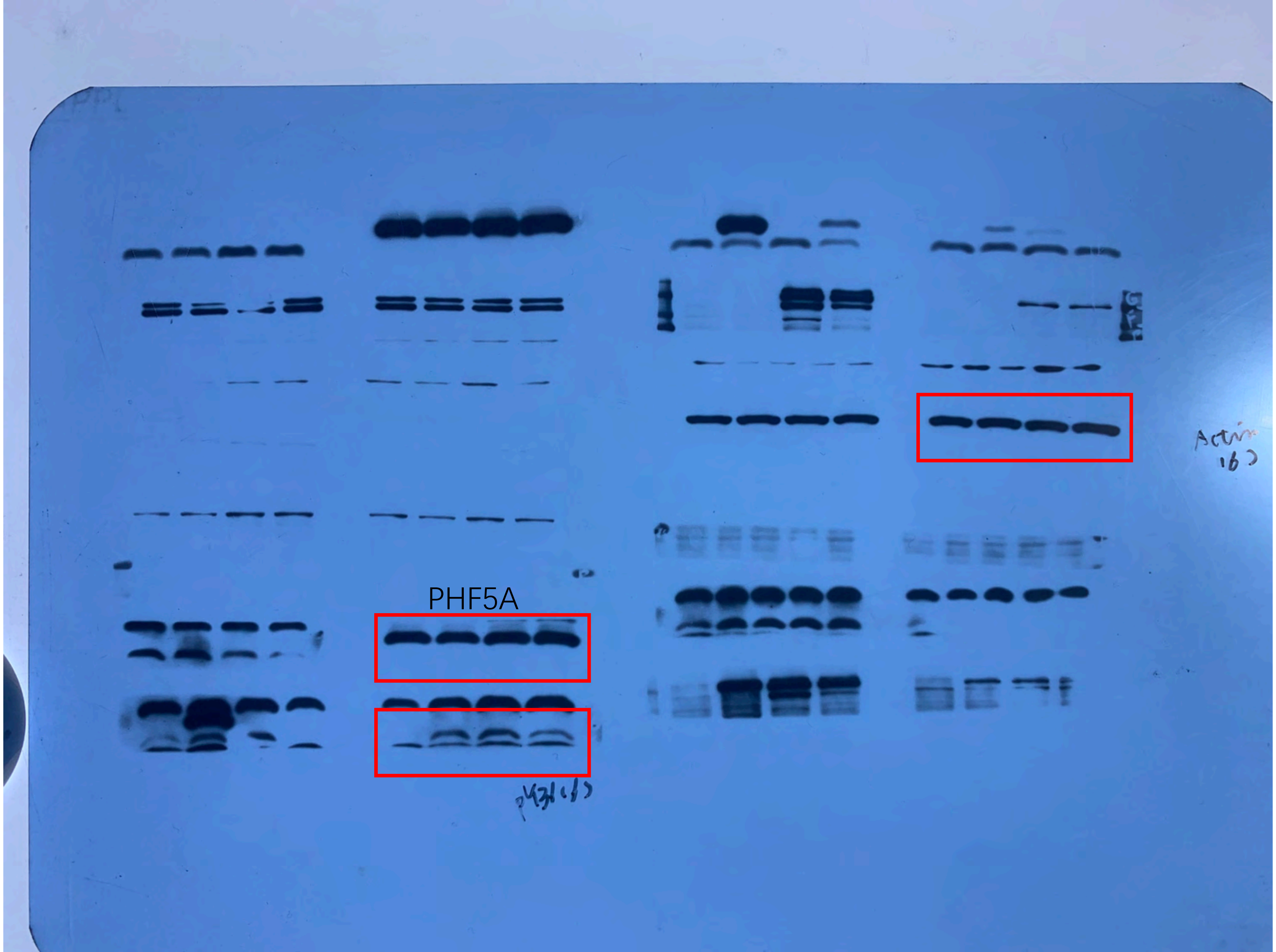

Fig. S2b

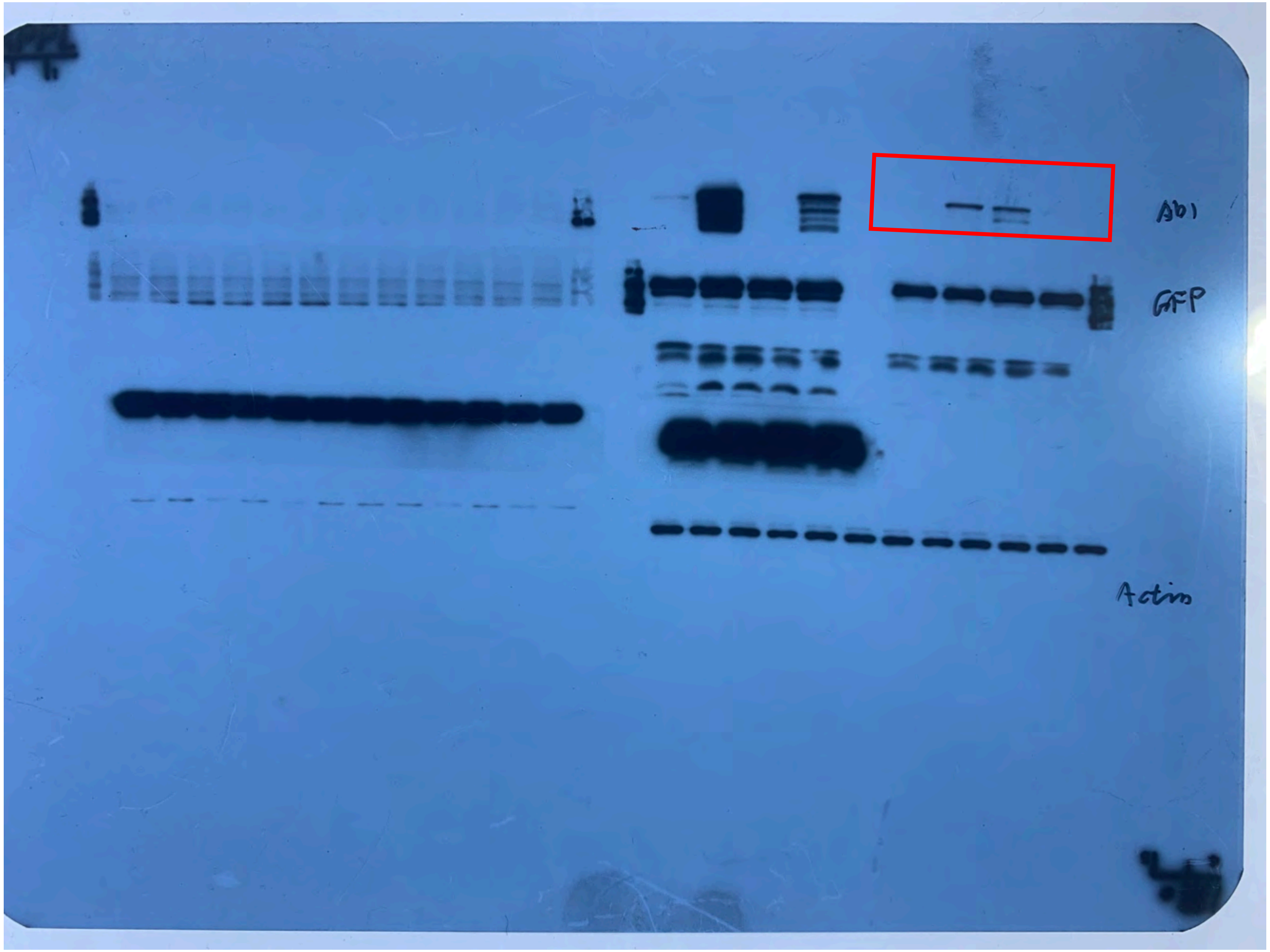

Fig. S2b

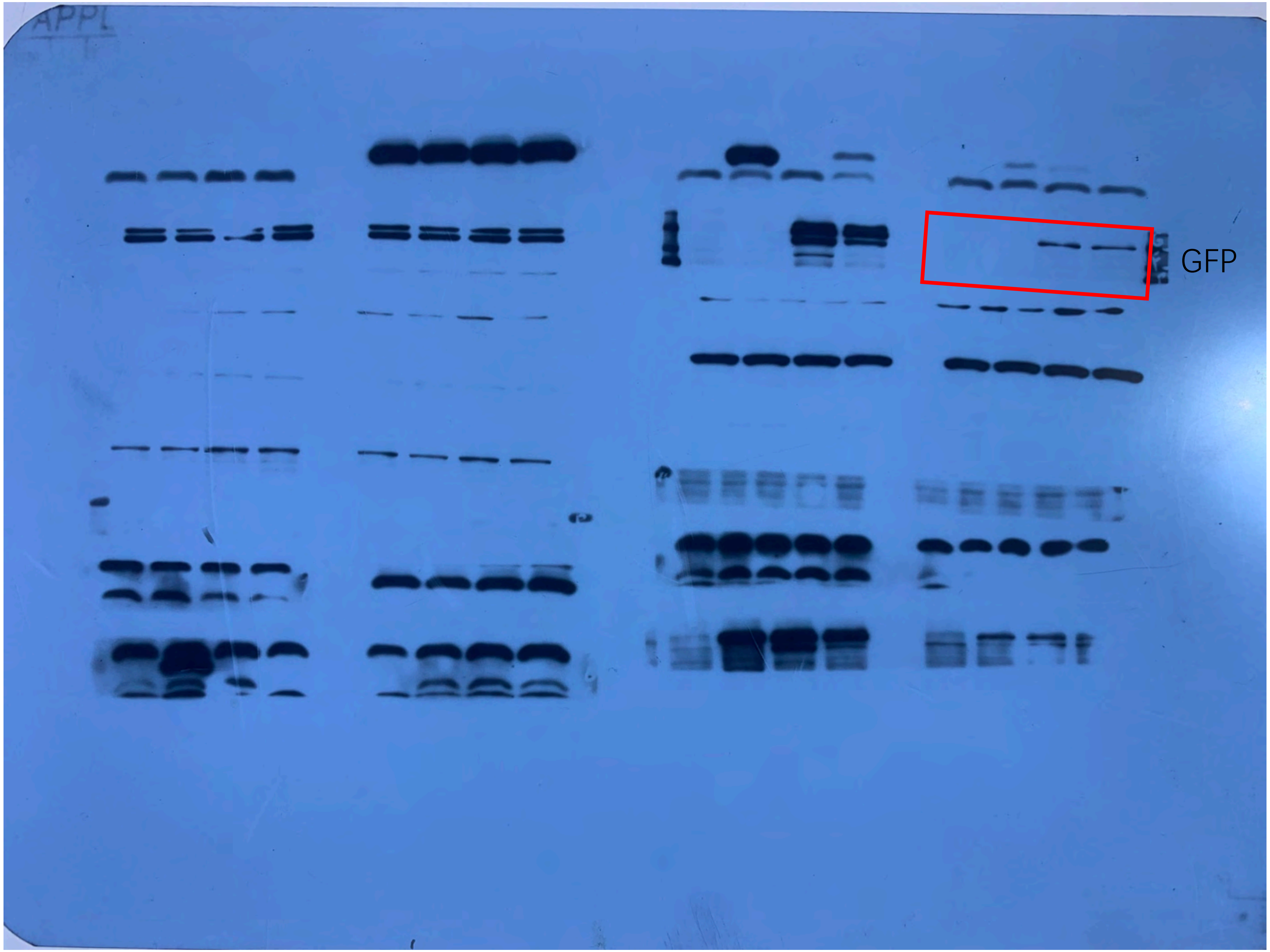

Fig. S2b

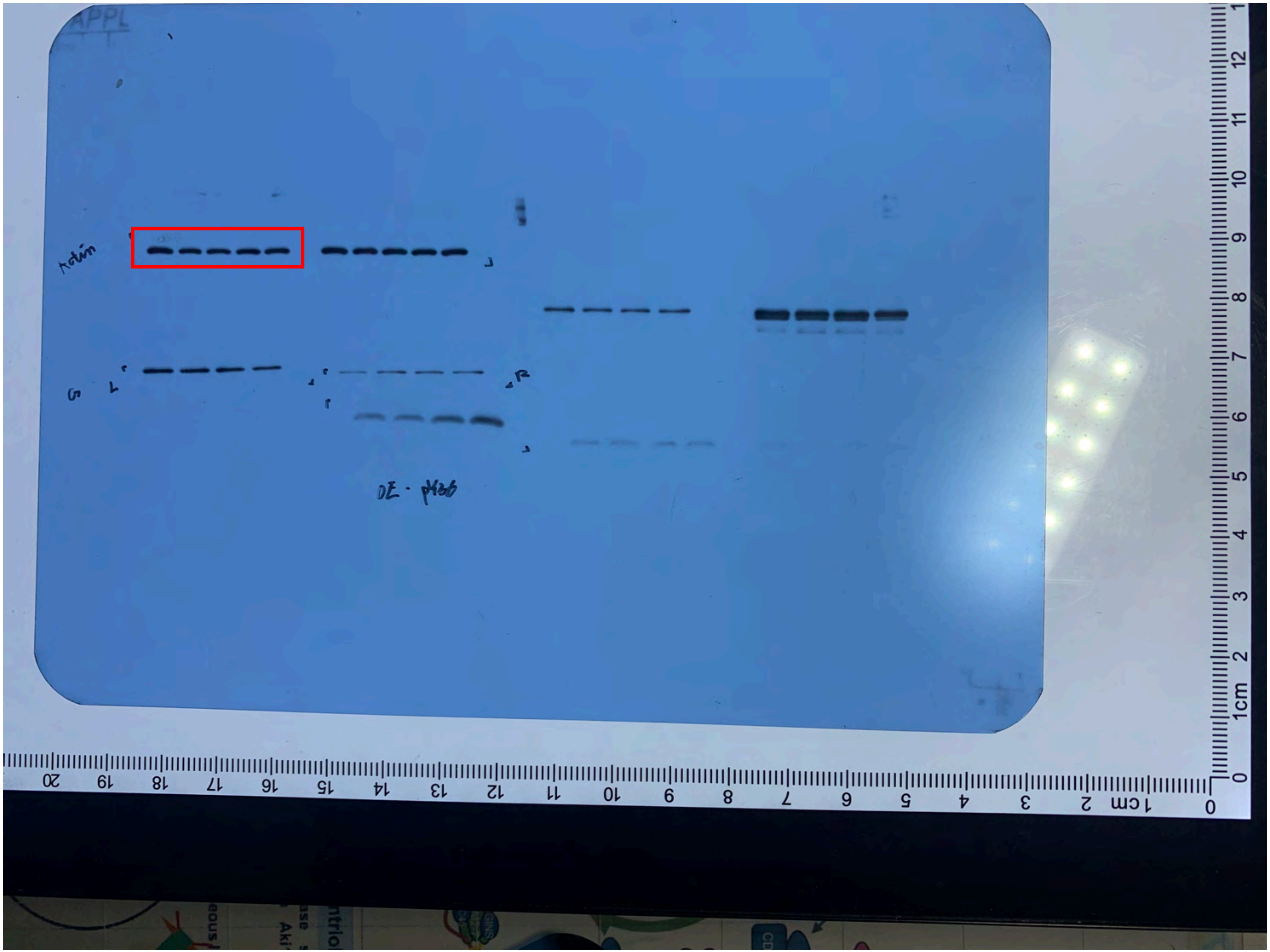

Fig. S2c

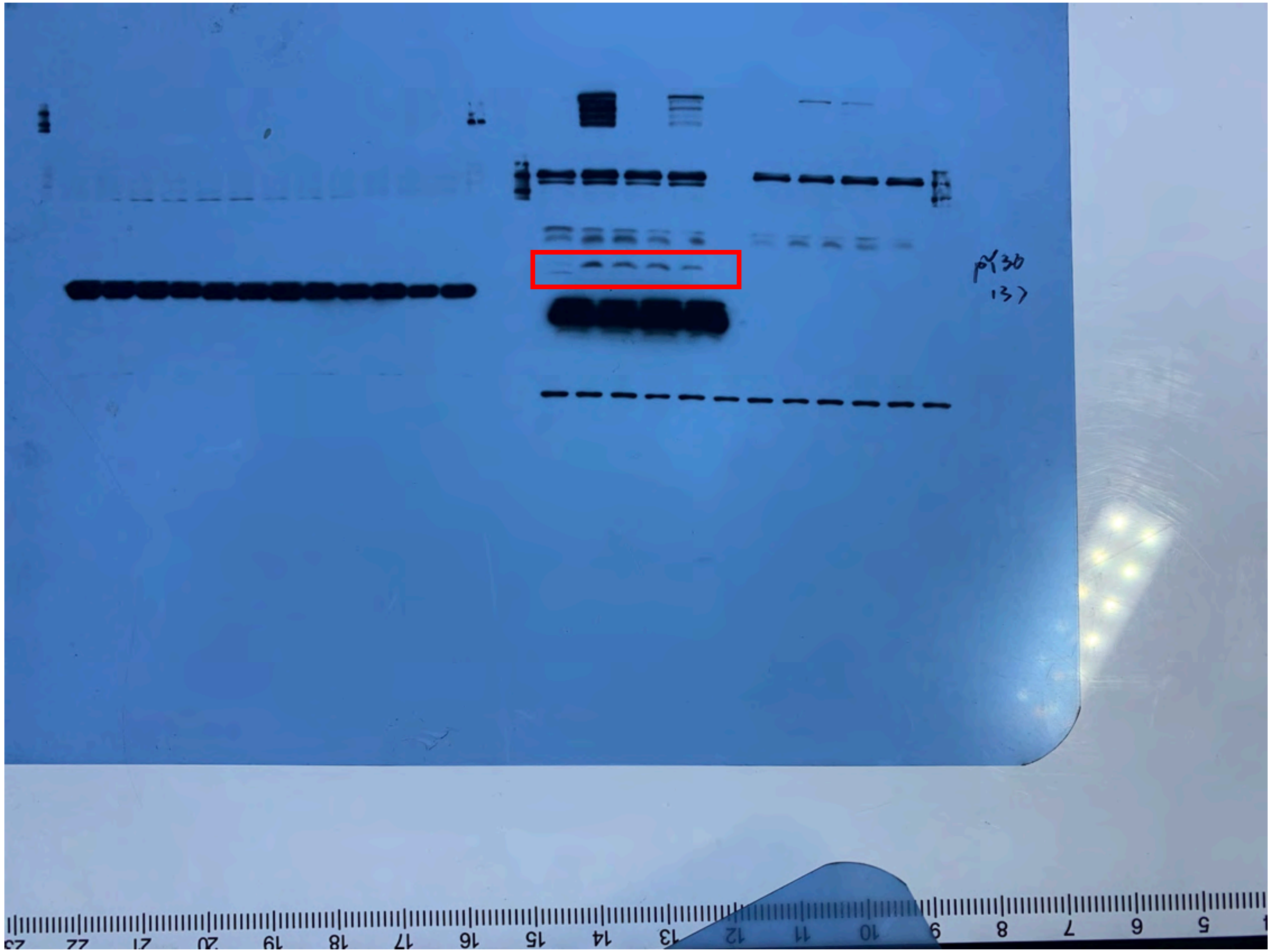

Fig. S2c

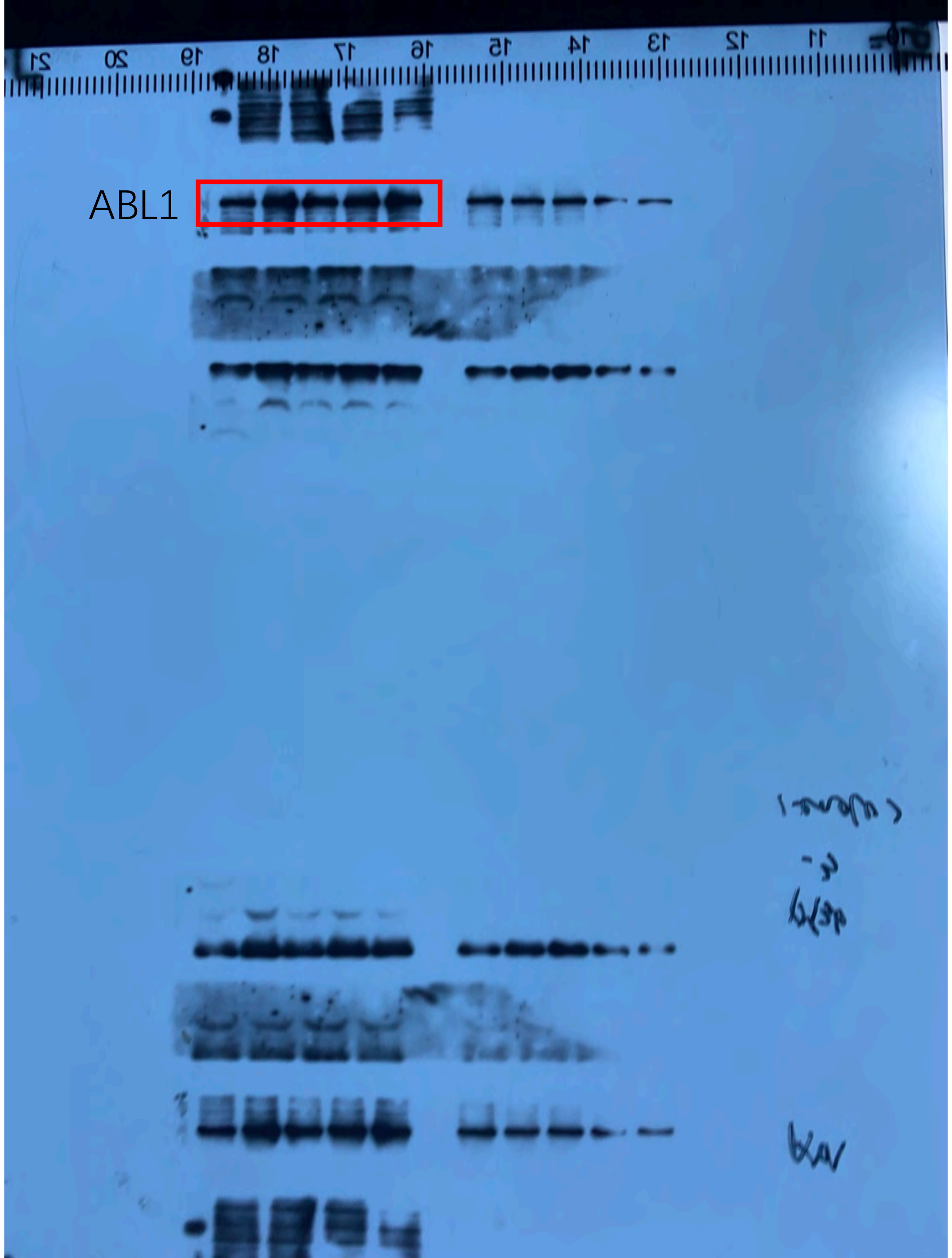

Fig. S2c

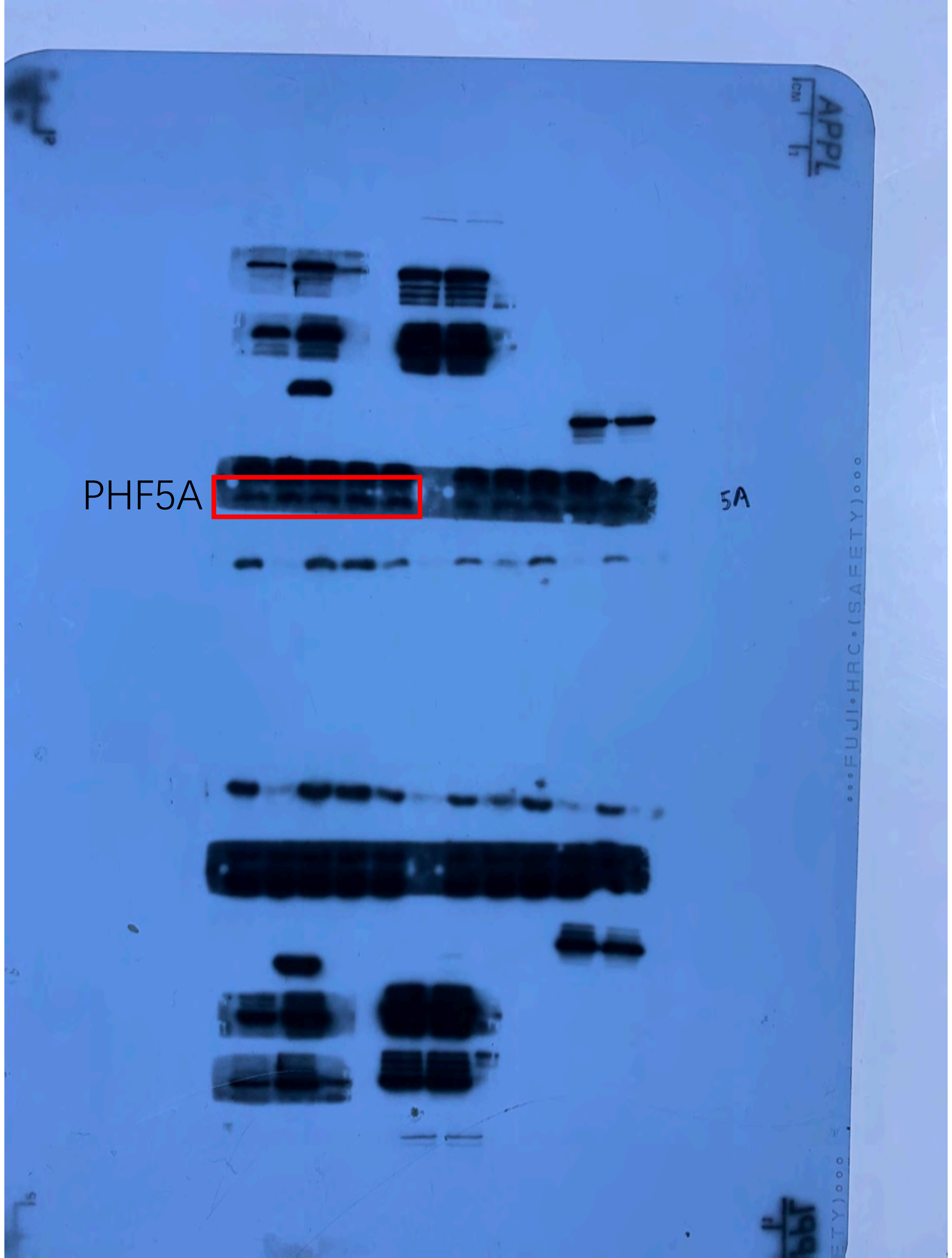

Fig. S2c

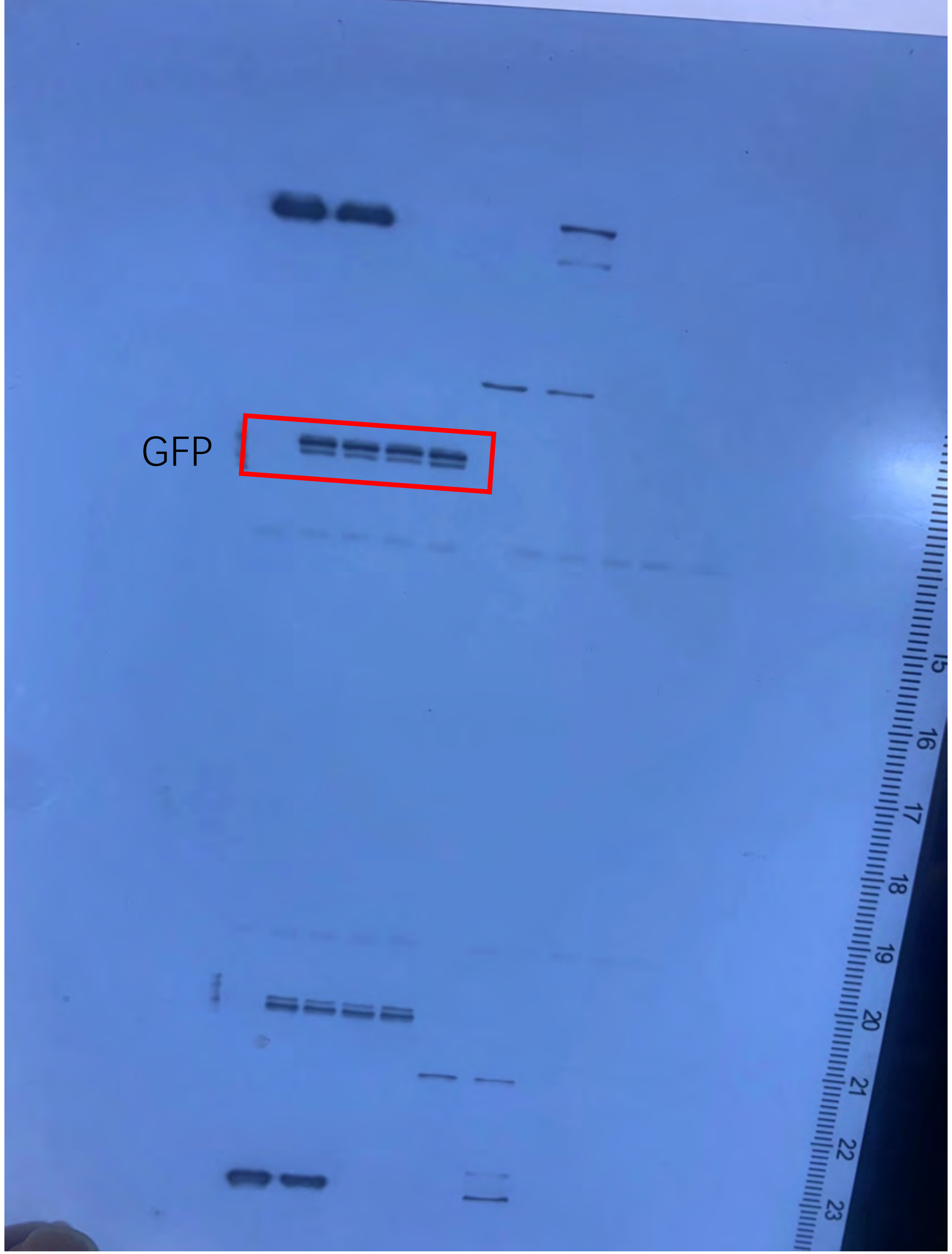

Fig. S2c

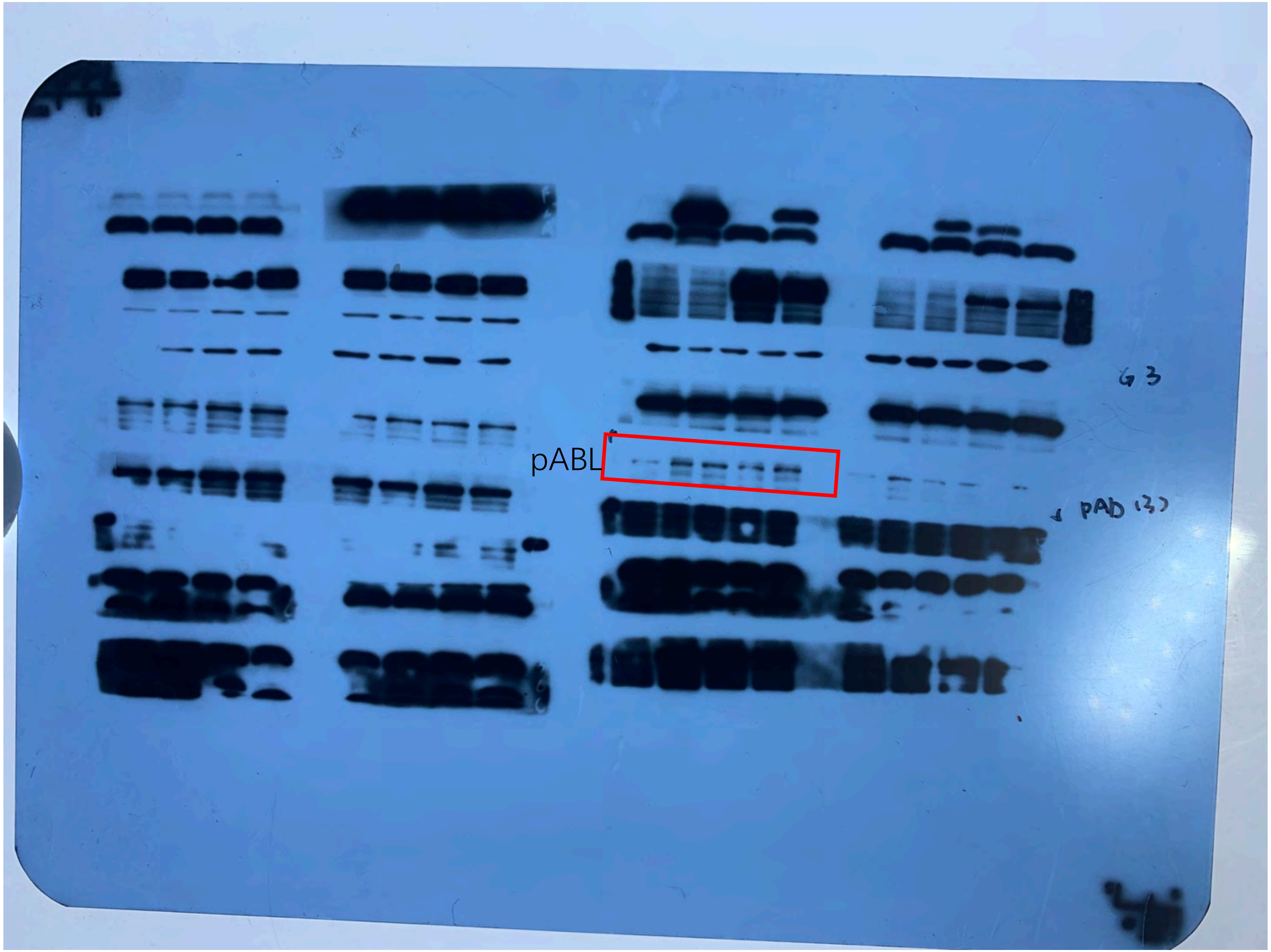

Fig. S2c

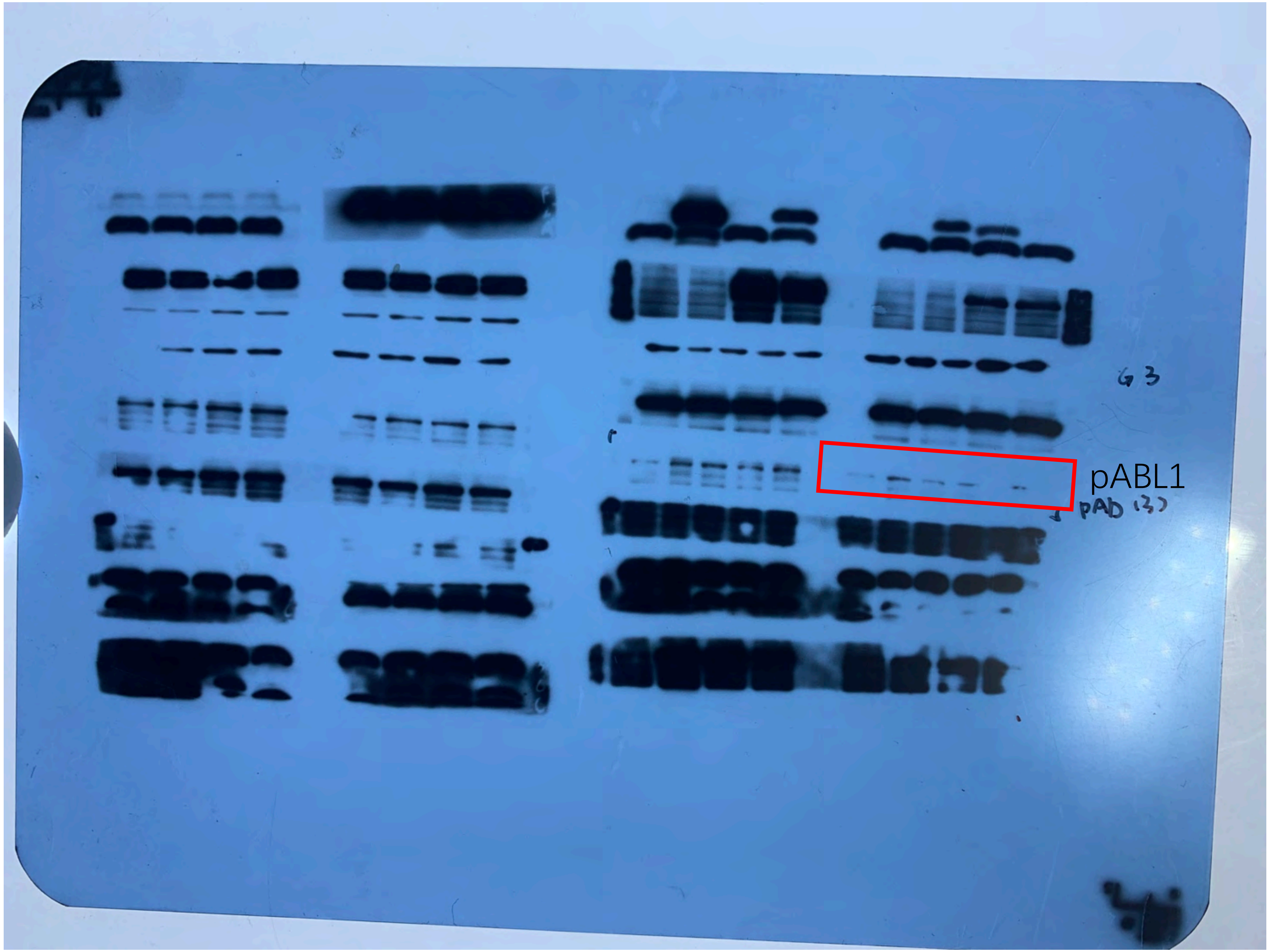

Fig. S2d

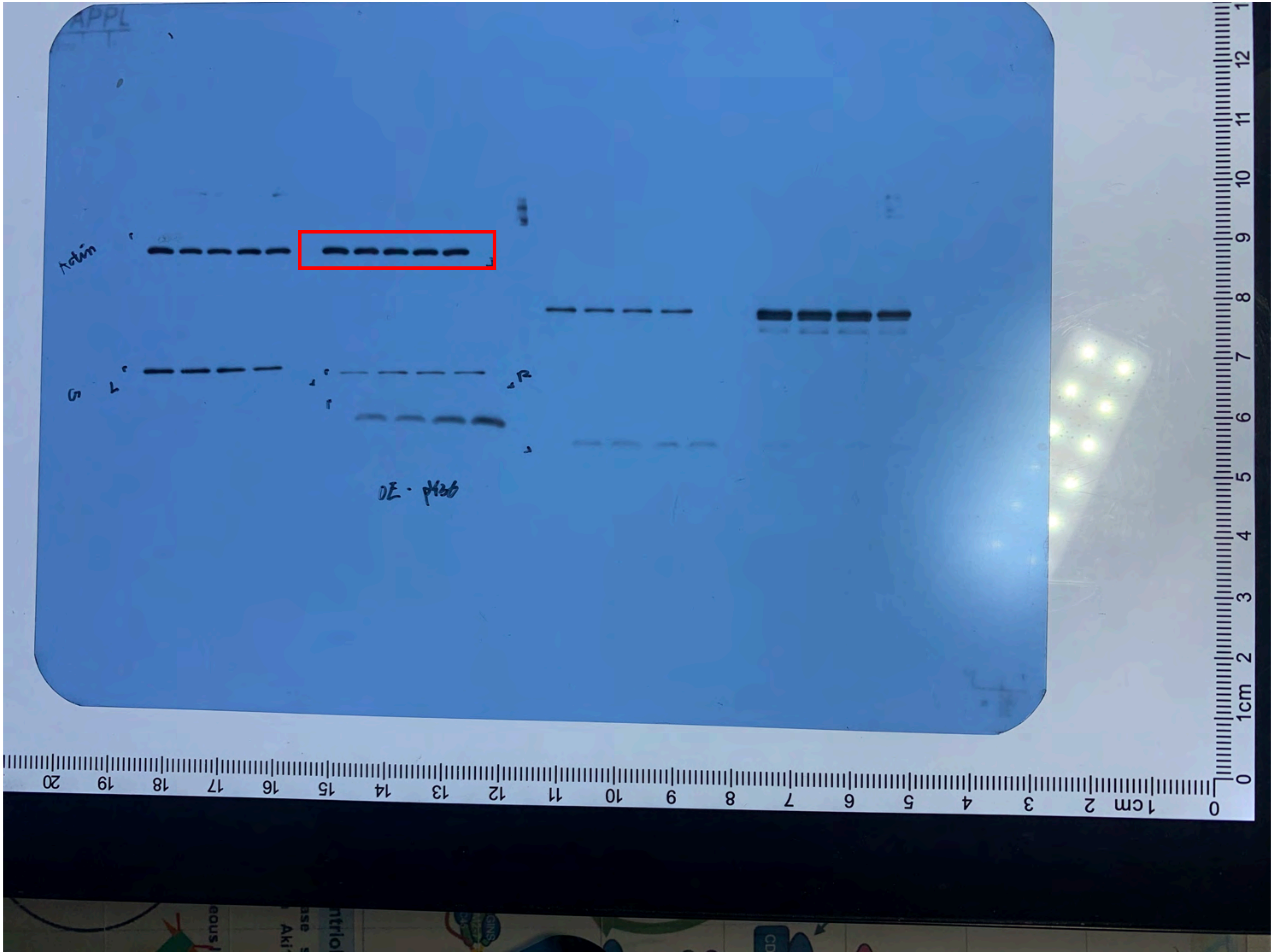

Fig. S2d

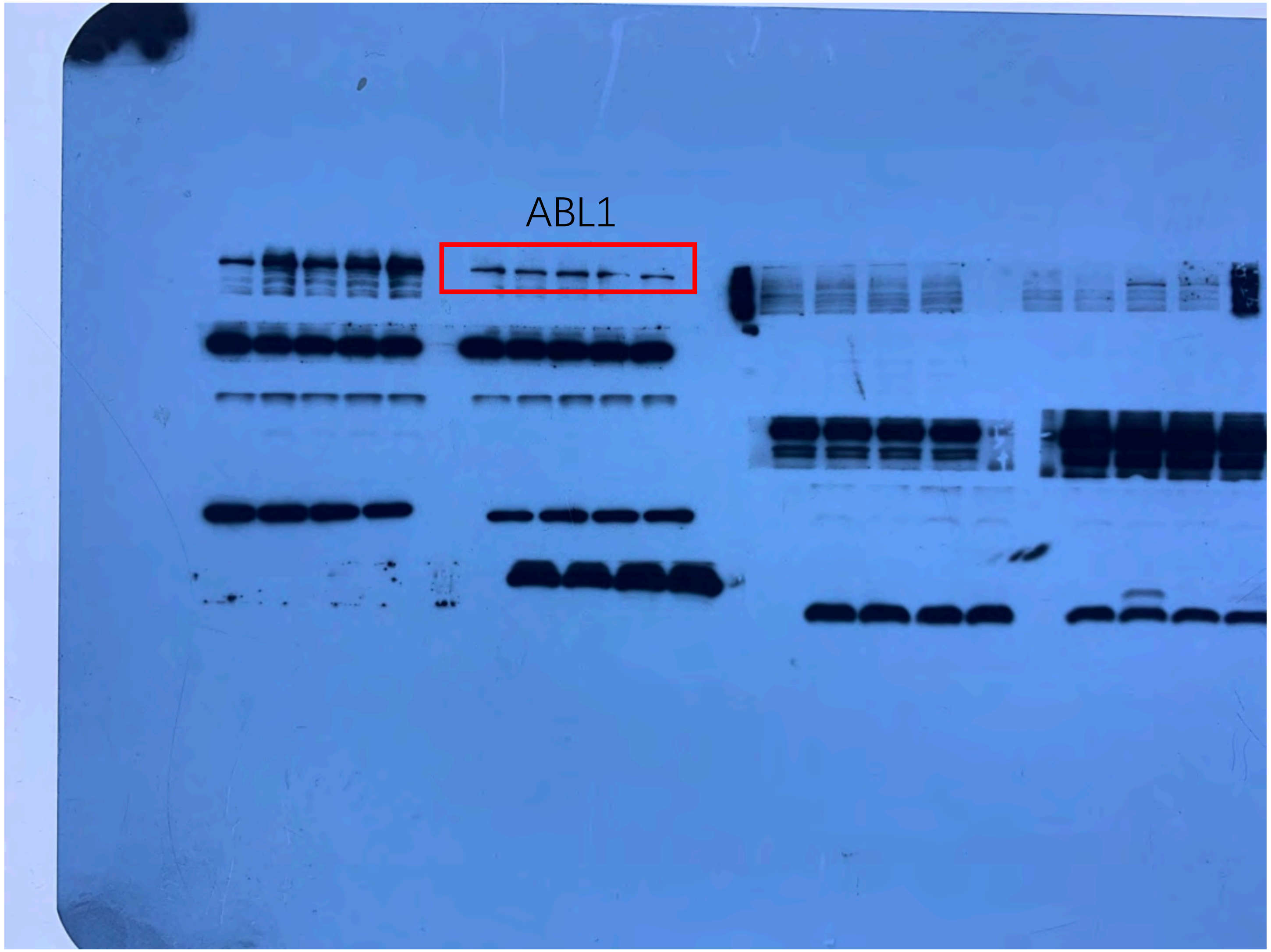

Fig. S2d

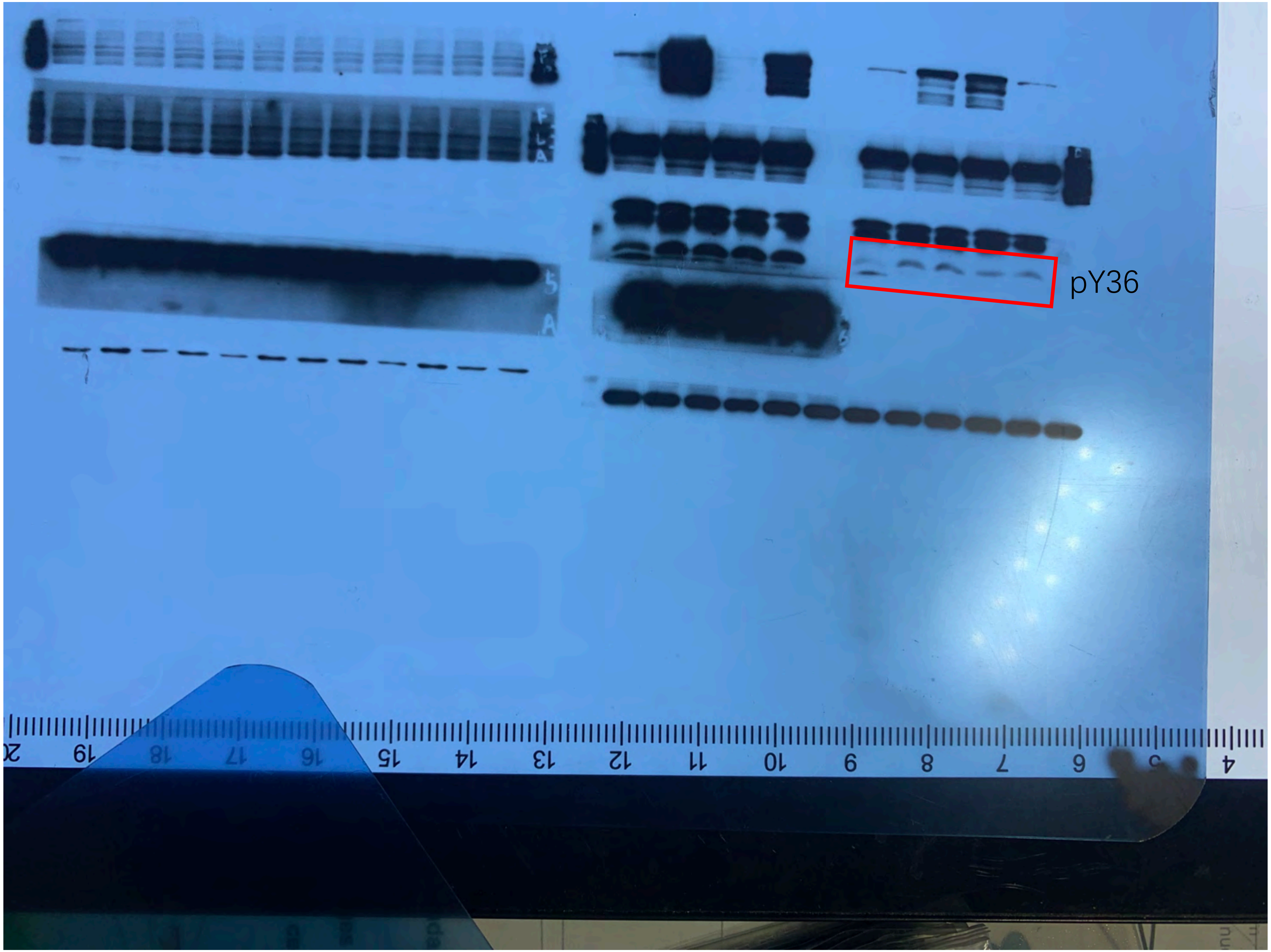

Fig. S2d

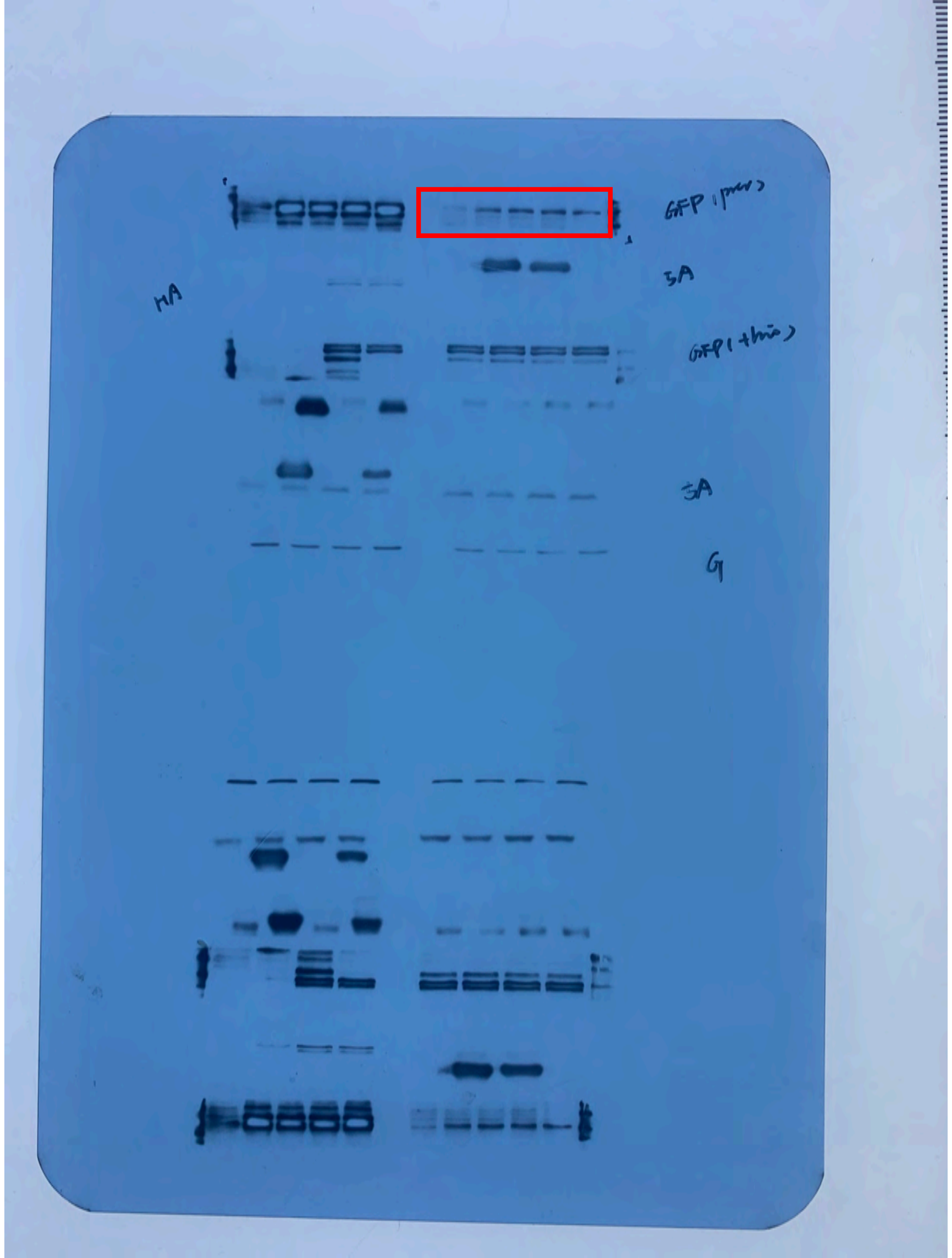

Fig. S2d

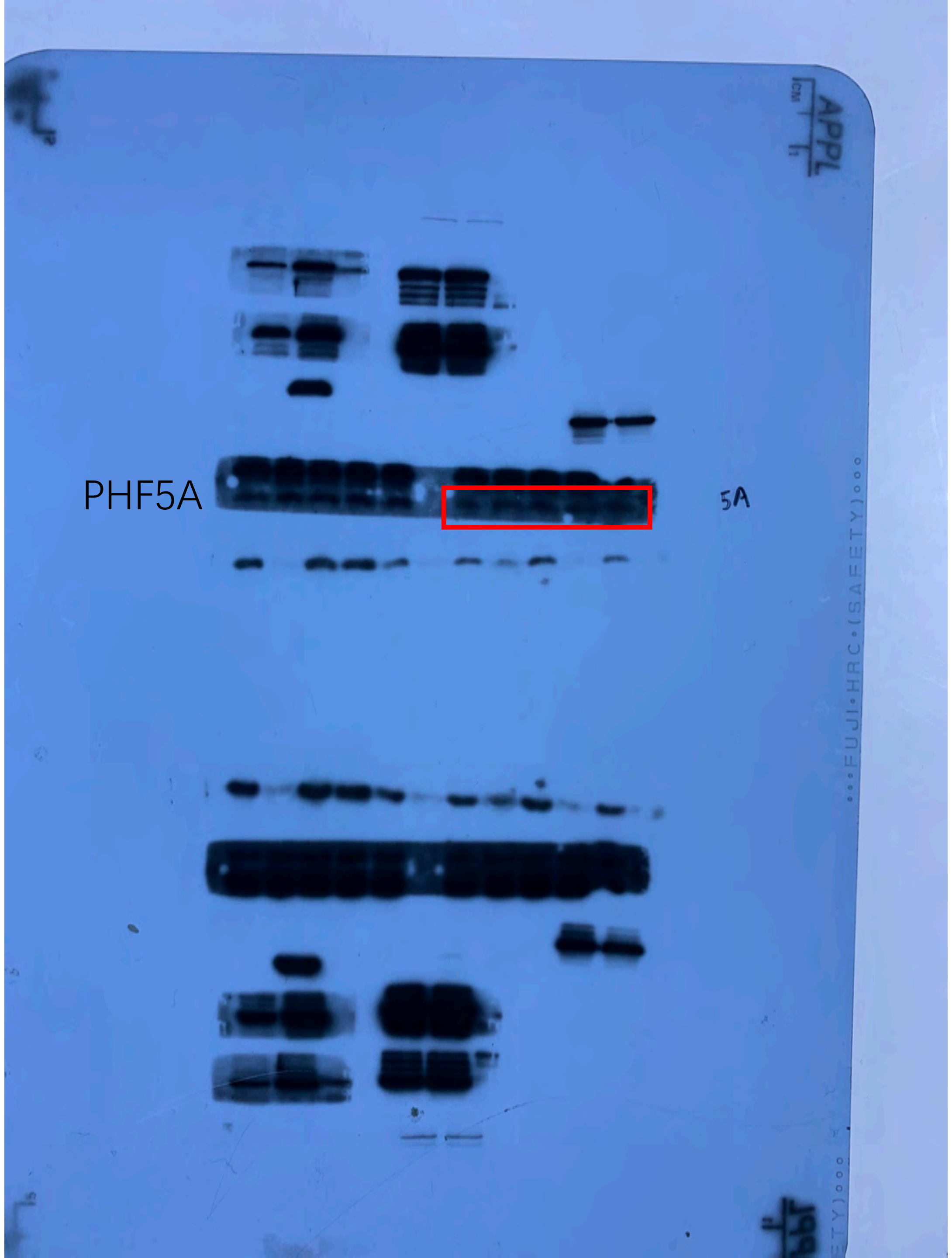

Fig. S2d

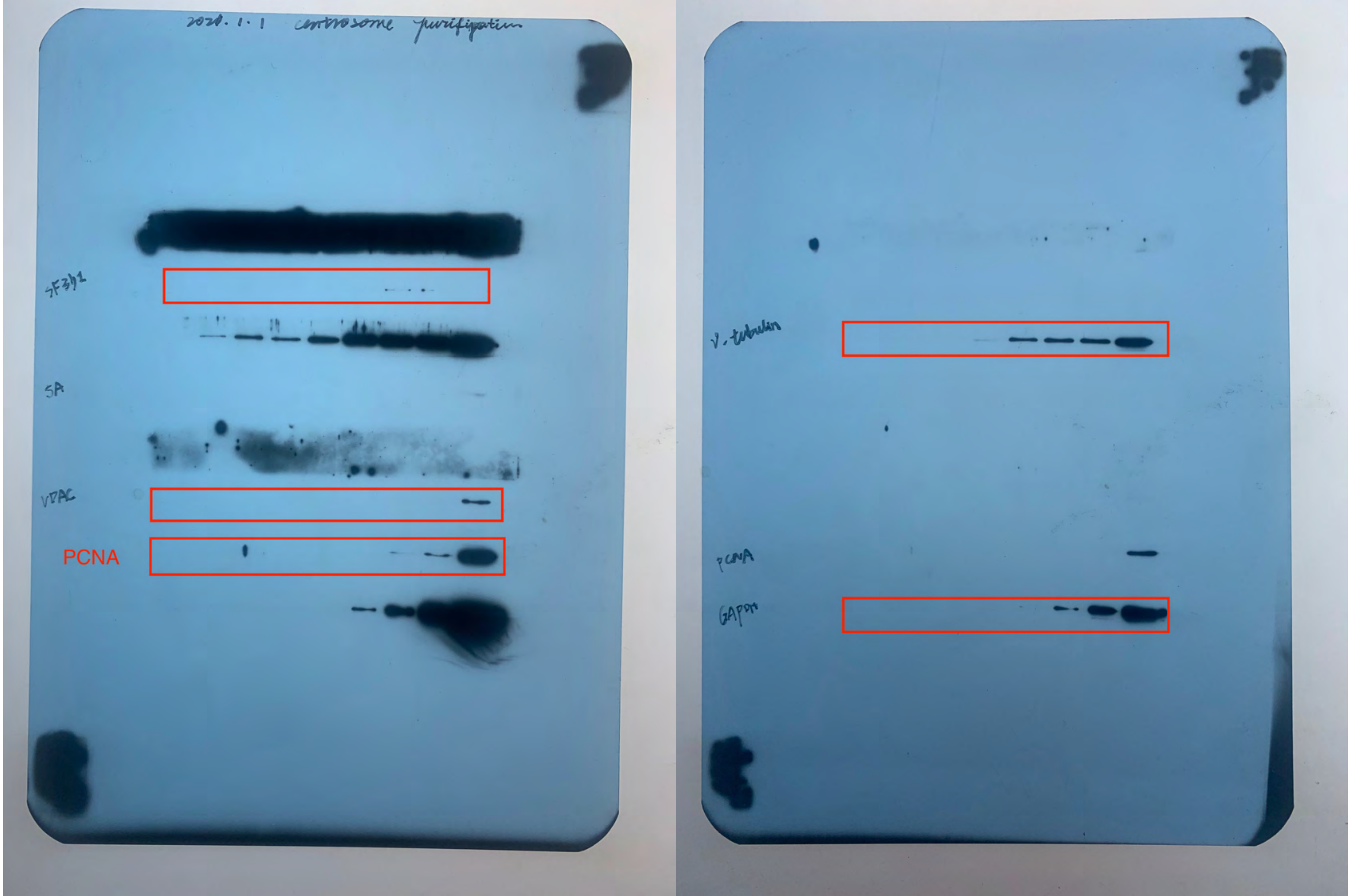

Fig. S3C

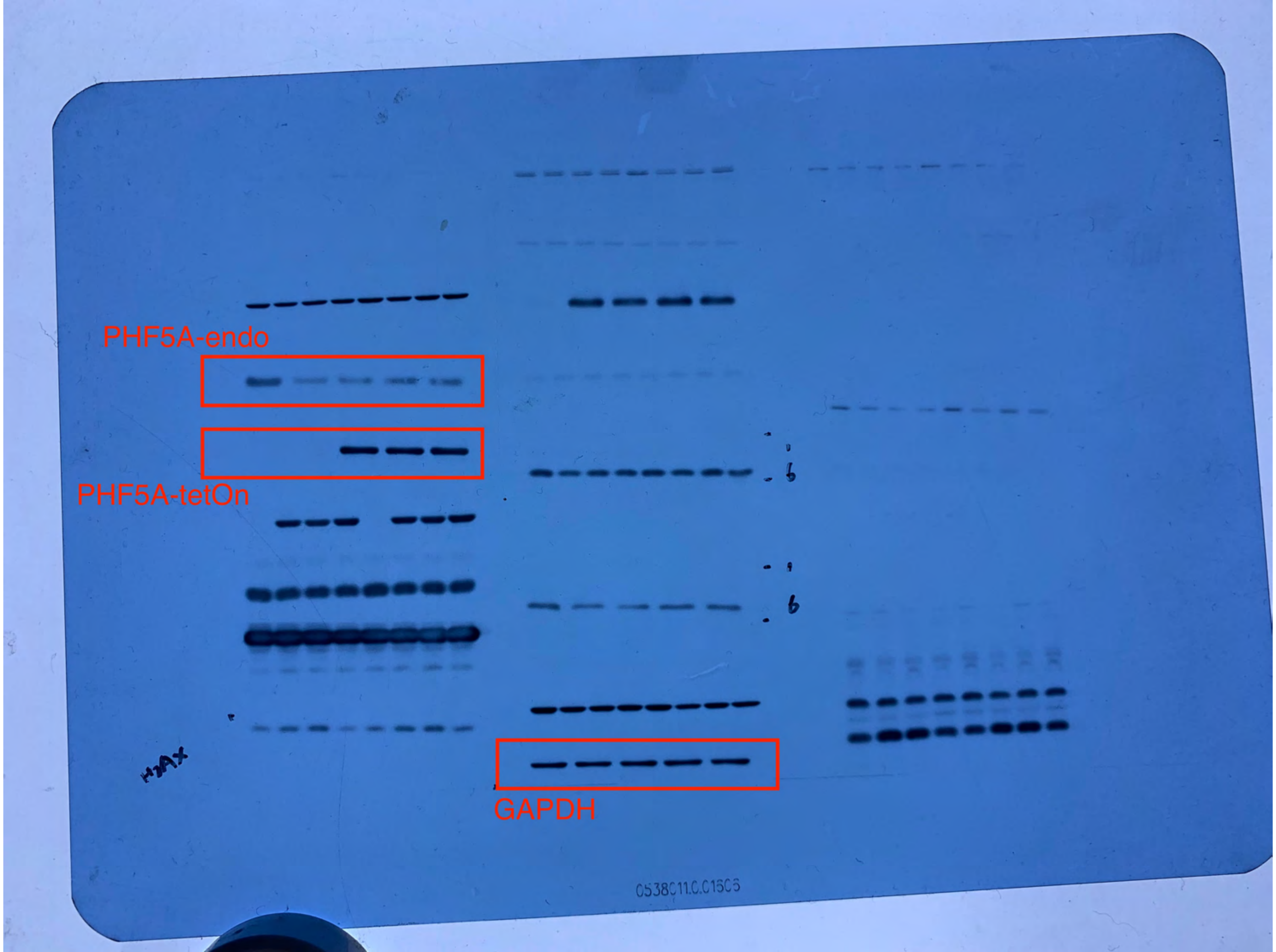

Fig. S6B

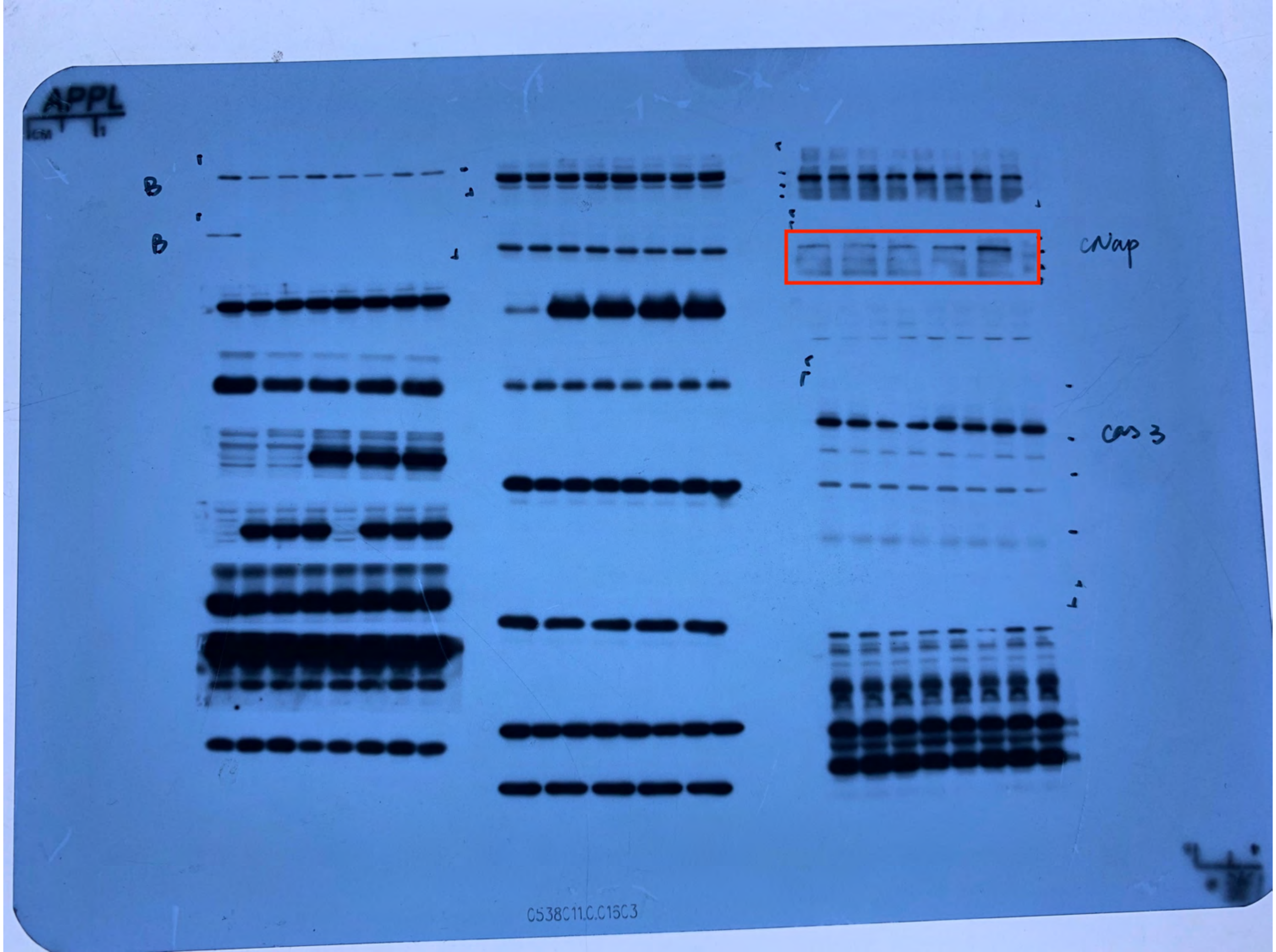

Fig.S6B
